# Author Correction: Macrophage and neutrophil heterogeneity at single-cell spatial resolution in human inflammatory bowel disease

**DOI:** 10.1038/s41467-024-45212-3

**Published:** 2024-01-29

**Authors:** Alba Garrido-Trigo, Ana M. Corraliza, Marisol Veny, Isabella Dotti, Elisa Melón-Ardanaz, Aina Rill, Helena L. Crowell, Ángel Corbí, Victoria Gudiño, Miriam Esteller, Iris Álvarez-Teubel, Daniel Aguilar, M. Carme Masamunt, Emily Killingbeck, Youngmi Kim, Michael Leon, Sudha Visvanathan, Domenica Marchese, Ginevra Caratù, Albert Martin-Cardona, Maria Esteve, Ingrid Ordás, Julian Panés, Elena Ricart, Elisabetta Mereu, Holger Heyn, Azucena Salas

**Affiliations:** 1 Inflammatory Bowel Disease Unit, Institut d’Investigacions Biomèdiques August Pi I Sunyer (IDIBAPS), Hospital Clínic, Barcelona, Spain; 2 Centro de Investigación Biomédica en Red de Enfermedades Hepáticas y Digestivas (CIBEREHD), Barcelona, Spain; 3 Josep Carreras Leukaemia Research Institute (IJC), Badalona, Spain; 4 Department of Molecular Life Sciences, University of Zurich, Switzerland. SIB Swiss Institute of Bioinformatics, Zurich, Switzerland; 5 Centro de Investigaciones Biológicas, Consejo Superior de Investigaciones Científicas (CSIC), Madrid, Spain; 6 NanoString Technologies, Seattle, WA USA; 7 Translational Medicine and Clinical Pharmacology, Boehringer-Ingelheim Pharmaceuticals Inc, Ridgefield, CT USA; 8 CNAG-CRG, Centre for Genomic Regulation (CRG), Barcelona Institute of Science and Technology (BIST), Barcelona, Spain; 9 Department of Gastroenterology, Hospital Universitari Mútua Terrassa, Universitat de Barcelona, Terrassa, Spain; 10 Universitat Pompeu Fabra (UPF), Barcelona, Spain

**Keywords:** Mucosal immunology, Inflammatory bowel disease, Imaging the immune system, Single-cell imaging

Correction to: *Nature Communications* 10.1038/s41467-023-40156-6, published online 26 July 2023

The original version of this Article omitted Ingrid Ordás from the author list as the 22nd author, who is affiliated to [1] Inflammatory Bowel Disease Unit, Institut d’Investigacions Biomèdiques August Pi I Sunyer (IDIBAPS), Hospital Clínic, Barcelona, Spain, and [2] Centro de Investigación Biomédica en Red de Enfermedades Hepáticas y Digestivas (CIBEREHD), Barcelona, Spain.

Additionally, the following was amended in the Author Contributions: ‘AM-C, MaE, IO, MCM and ER provided human samples and clinical information.’

This has been corrected in both the PDF and HTML versions of the Article.

