# Supplementary material for: Macrophage and neutrophil heterogeneity at single-cell spatial resolution in human inflammatory bowel disease
Source: Nat Commun. 2023 Jul 26;14:4506. doi: 10.1038/s41467-023-40156-6 (PMC10372067; doi:10.1038/s41467-023-40156-6)
Supplement: Supplementary file 1 — Supplementary Information [file 41467_2023_40156_MOESM1_ESM.pdf]

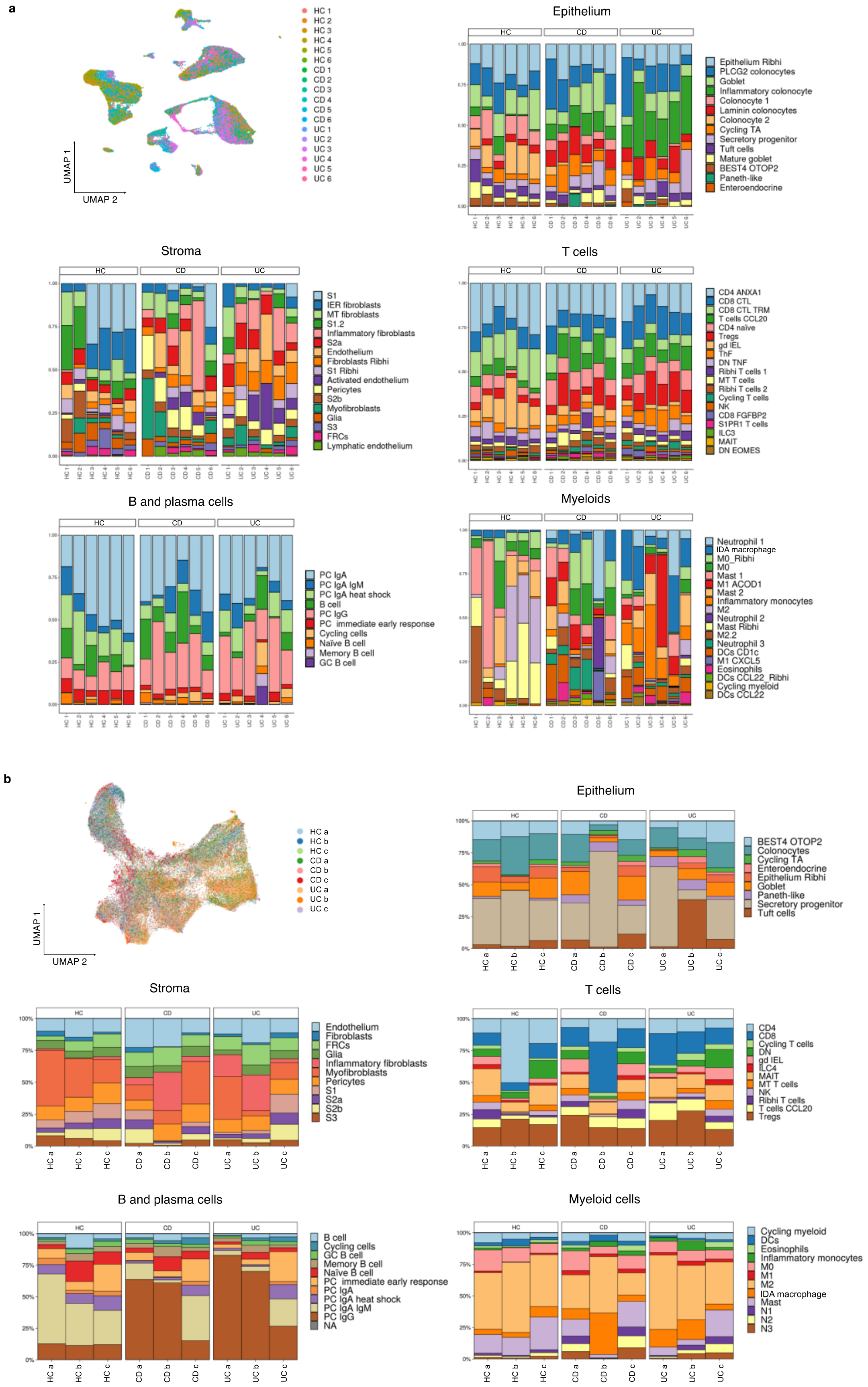
a
HC 1
HC 2
HC 3
HC 4
HC 5
HC 6
CD 1
CD 2
CD 3
CD 4
CD 5
CD 6
UC 1
UC 2
UC 3
UC 4
UC 5
UC 6
UMAP 1
UMAP 2
Epithelium
Epithelium Ribhi
PLCG2 colonocytes
Goblet
Inflammatory colonocyte
Colonocyte 1
Laminin colonocytes
Colonocyte 2
Cycling TA
Secretory progenitor
Tuft cells
Mature goblet
BEST4 OTOP2
Paneth-like
Enteroendocrine
Stroma
S1
IER fibroblasts
MT fibroblasts
S1.2
Inflammatory fibroblasts
S2a
Endothelium
Fibroblasts Ribhi
S1 Ribhi
Activated endothelium
Pericytes
S2b
Myofibroblasts
Glia
S3
FRCs
Lymphatic endothelium
T cells
CD4 ANXA1
CD8 CTL
CD8 CTL TRM
T cells CCL20
CD4 naïve
Tregs
gd IEL
ThF
DN TNF
Ribhi T cells 1
MT T cells
Ribhi T cells 2
Cycling T cells
NK
CD8 FGFBP2
S1PR1 T cells
ILC3
MAIT
DN EOMES
B and plasma cells
PC IgA
PC IgA IgM
PC IgA heat shock
B cell
PC IgG
PC immediate early response
Cycling cells
Naïve B cell
Memory B cell
GC B cell
Myeloids
Neutrophil 1
IDA macrophage
M0_Ribhi
M0
Mast 1
M1 ACOD1
Mast 2
Inflammatory monocytes
M2
Neutrophil 2
Mast Ribhi
M2.2
Neutrophil 3
DCs CD1c
M1 CXCL5
Eosinophils
DCs CCL22_Ribhi
Cycling myeloid
DCs CCL22
b
HC a
HC b
HC c
CD a
CD b
CD c
UC a
UC b
UC c
UMAP 1
UMAP 2
Epithelium
BEST4 OTOP2
Colonocytes
Cycling TA
Enteroendocrine
Epithelium Ribhi
Goblet
Paneth-like
Secretory progenitor
Tuft cells
Stroma
Endothelium
Fibroblasts
FRCs
Glia
Inflammatory fibroblasts
Myofibroblasts
Pericytes
S1
S2a
S2b
S3
T cells
CD4
CD8
Cycling T cells
DN
gd IEL
ILC4
MAIT
MT T cells
NK
Ribhi T cells
T cells CCL20
Tregs
B and plasma cells
B cell
Cycling cells
GC B cell
Memory B cell
Naïve B cell
PC immediate early response
PC IgA
PC IgA heat shock
PC IgA IgM
PC IgG
NA
Myeloid cells
Cycling myeloid
DCs
Eosinophils
Inflammatory monocytes
M0
M1
M2
IDA macrophage
Mast
N1
N2
N3
100%
75%
50%
25%
0%

c

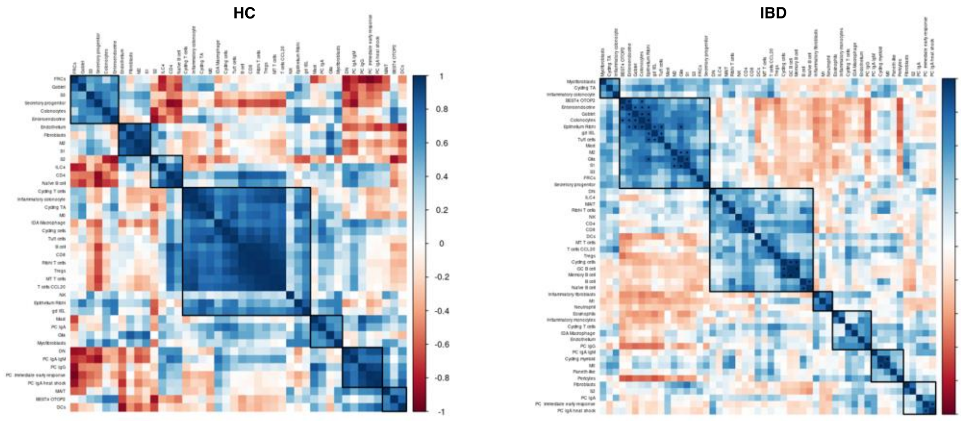
HC
IBD
1
0.8
0.6
0.4
0.2
0
-0.2
-0.4
-0.6
-0.8
-1

d

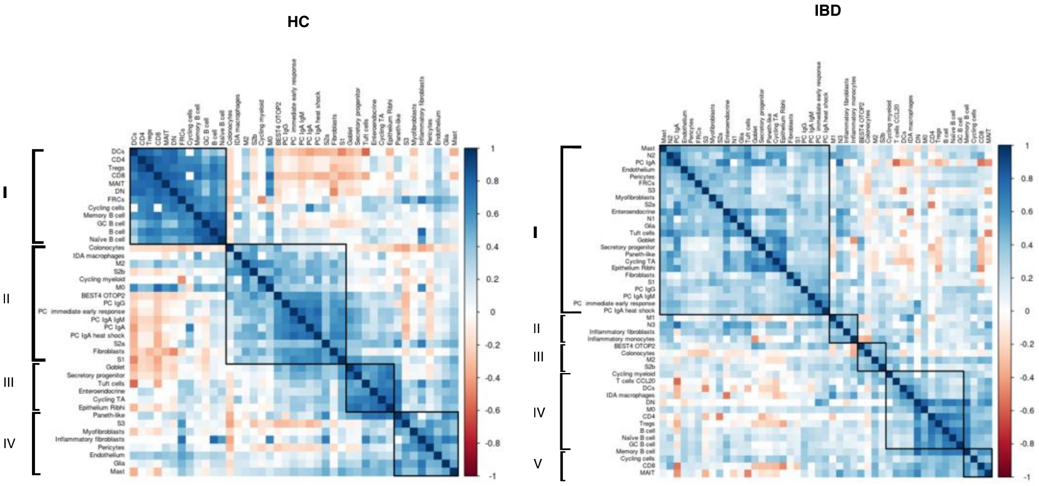
HC
IBD
I
II
III
IV
V
DCs
CD4
Tregs
CD8
MAIT
DN
FRCs
Cycling cells
Memory B cell
GC B cell
B cell
Naïve B cell
Colonocytes
IDA macrophages
M2
S2b
Cycling myeloid
M0
BEST4 OTOP2
PC IgG
PC immediate early response
PC IgA IgM
PC IgA
PC IgA heat shock
S2a
Fibroblasts
S1
Goblet
Secretory progenitor
Tuft cells
Enteroendocrine
Cycling TA
Epithelium Ribhi
Paneth-like
S3
Myofibroblasts
Inflammatory fibroblasts
Pericytes
Endothelium
Glia
Mast
N2
N1
M1
N3
Inflammatory monocytes
T cells CCL20
1
0.8
0.6
0.4
0.2
0
-0.2
-0.4
-0.6
-0.8
-1

Supplementary Figure 1. Single-cell RNA-seq (scRNA-seq) and CosMx™ Spatial Molecular Imaging (SMI) cell clusters. a, UMAP representation of a sample (n=18) distribution across subsets analyzed by scRNA-seq. Barplots describe the proportions of each cell type within each cell subset (epithelium, stroma, B cells and plasma cells, T cells and myeloid cells) in healthy controls (HC, n=6), active CD (n=6) and active UC (n=6) patients using scRNA-seq data. **b**, UMAP representation of samples analyzed by CosMx™ SMI (n=9). Cells in CosMx™ SMI were annotated by label-transfer of scRNA-seq differentially expressed genes per cell cluster. Barplots describe the proportions of each cell type within each cell subset (epithelium, stroma, B cells and plasma cells, T cells and myeloid cells) in HC (n=3), active CD (n=3) and UC (n=3) patients using CosMx™ SMI data. **c**, Heatmap showing the correlation of abundances between cell populations present in the scRNAseq data in HC and IBD. *p<0.05 adjusted for multiple comparison using the false discovery rate. **d**, Heatmap showing the spatial correlation of abundances in CosMx SMI data in HC and IBD. Brackets and numbers show neighborhoods. *p<0.05 adjusted for multiple comparison using the false discovery rate. Source data are provided as a Source Data file.

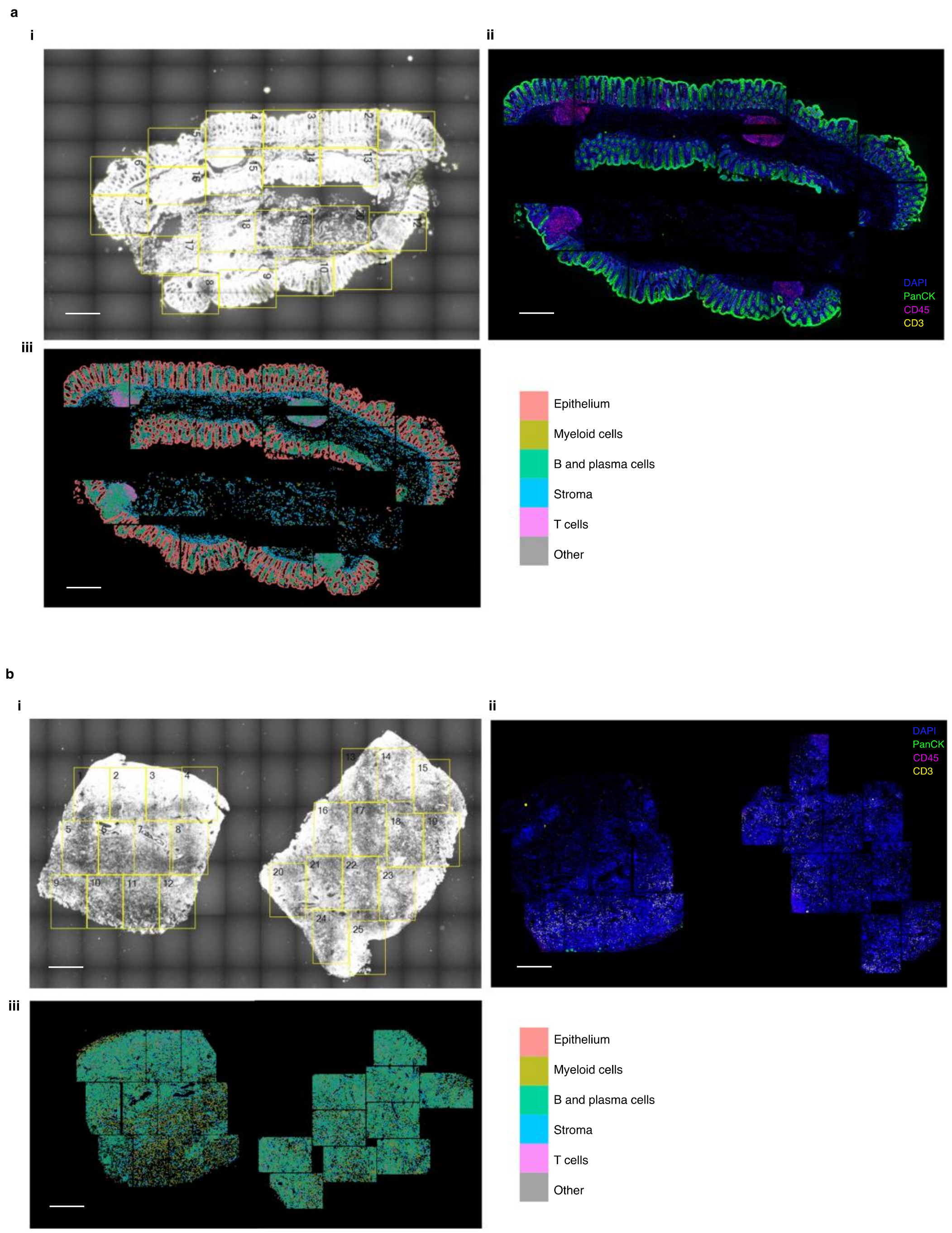

Supplementary Figure 2. CosMx^{TM} Spatial Molecular Imaging (SMI) of healthy and inflammatory bowel disease (IBD) colonic tissue. Panels show tissue scanner (**i**); nuclei (blue) and protein staining for cytokeratin (green), CD45 (magenta) and CD3 (yellow) (**ii**); and cell type annotation based on scRNA-seq data (**iii**) of one representative (**a**), healthy control and (**b**), ulcerative colitis patient, respectively. Scale bar = 500 µm. Source data are provided as a Source Data file.

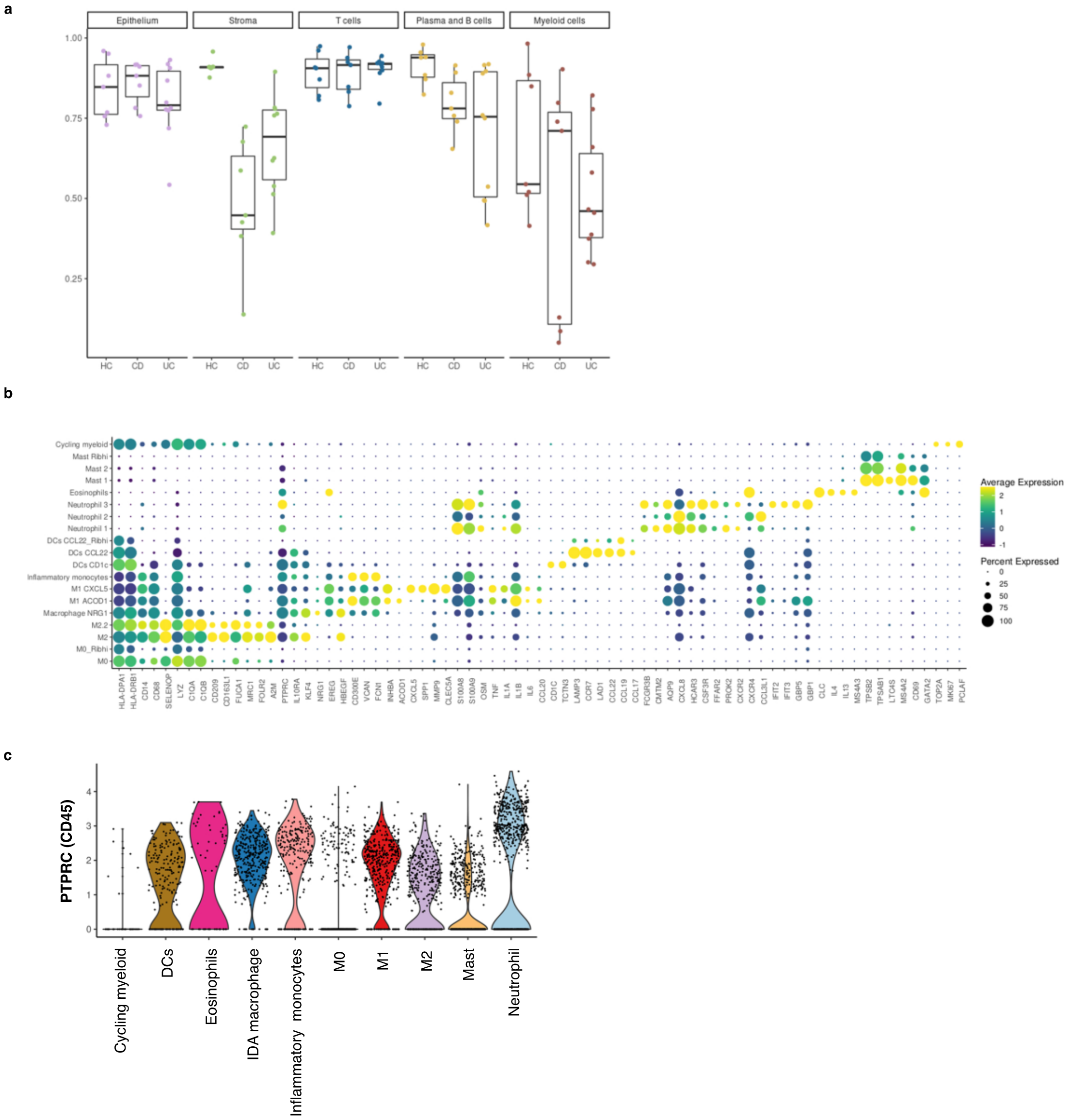

Supplementary Figure 3. Myeloid characterization. **a**, Morisita-Index analysis of dispersion between samples within each group (HC, CD, UC) for each cell subset (epithelium, stroma, B cells and plasma cells, T cells and myeloid cells) based on scRNA-seq data (HC, n=6), active CD (n=6) and active UC (n=6) patients). The upper whisker extends from the hinge to the largest value no further than 1.5 * IQR from the hinge (where IQR is the inter-quartile range, or distance between the first and third quartiles). The lower whisker extends from the hinge to the smallest value at most 1.5 * IQR of the hinge. The lower and upper hinges correspond to the first and third quartiles (the 25th and 75th percentiles) **b**, Myeloid cell subsets and their top gene markers. Dot plot shows the fraction of expressing cells (size of the dot) and mean expression levels (dot color). **c**, Violin plot visualization of PTPRC (CD45) expression in myeloid populations by scRNA-seq pooled data of (HC, n=6), active CD (n=6) and active UC (n=6) patients). Source data are provided as a Source Data file.

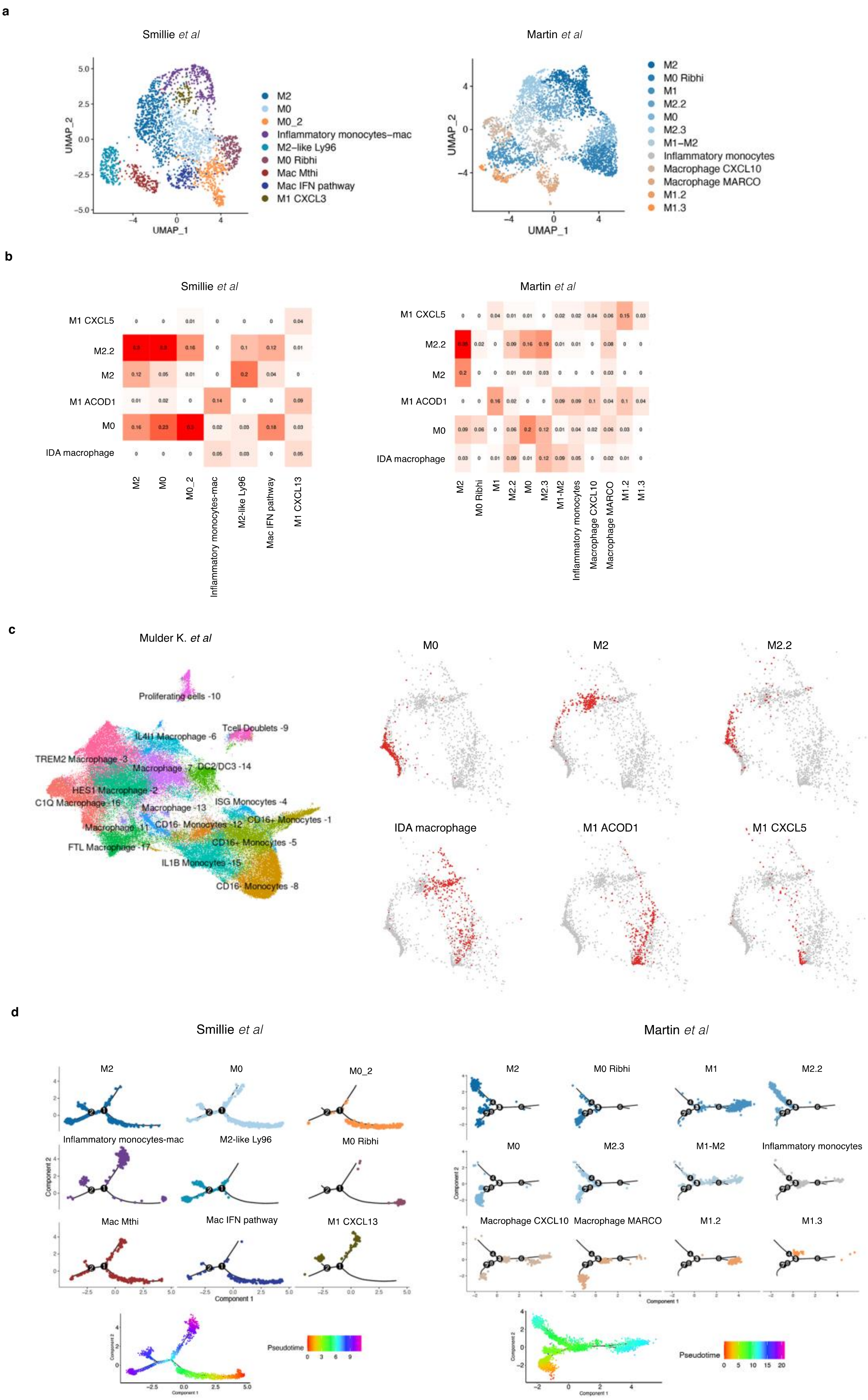
a
Smillie et al
Martin et al
UMAP_1
UMAP_2
M2
M0
M0_2
Inflammatory monocytes−mac
M2−like Ly96
M0 Ribhi
Mac Mthi
Mac IFN pathway
M1 CXCL3
M2
M0 Ribhi
M1
M2.2
M0
M2.3
M1−M2
Inflammatory monocytes
Macrophage CXCL10
Macrophage MARCO
M1.2
M1.3
b
Smillie et al
Martin et al
M1 CXCL5
M2.2
M2
M1 ACOD1
M0
IDA macrophage
c
Mulder K. et al
Proliferating cells -10
Tcell Doublets -9
IL4I1 Macrophage -6
TREM2 Macrophage -3
Macrophage -7
DC2/DC3 -14
HES1 Macrophage -2
C1Q Macrophage -16
ISG Monocytes -4
Macrophage -13
CD16- Monocytes -12
CD16+ Monocytes -1
Macrophage -11
CD16+ Monocytes -5
FTL Macrophage -17
IL1B Monocytes -15
CD16- Monocytes -8
M0
M2
M2.2
IDA macrophage
M1 ACOD1
M1 CXCL5
d
Smillie et al
Martin et al
M2
M0
M0_2
Inflammatory monocytes-mac
M2-like Ly96
M0 Ribhi
Mac Mthi
Mac IFN pathway
M1 CXCL13
M2
M0 Ribhi
M1
M2.2
M0
M2.3
M1-M2
Inflammatory monocytes
Macrophage CXCL10
Macrophage MARCO
M1.2
M1.3
Component 1
Component 2
Pseudotime

Supplementary Figure 4. Intestinal Macrophages across studies. a, UMAP representation of Smillie *et al-* and Martin *et al-* macrophage subsets isolated *in silico*. **b**, Jaccard similarity index between scRNA-seq data of Smillie *et al-* and Martin *et al-* macrophages and our scRNA-seq macrophage populations. **c**, Projection of our intestinal macrophage clusters found in our study into the MoMAc-VERSE UMAP data (Mulder *et al.2021*). **d**, Pseudo-time trajectory analysis of Smillie *et al-* and Martin *et al- in silico* isolated colonic and ileal macrophages, respectively. Source data are provided as a Source Data file.

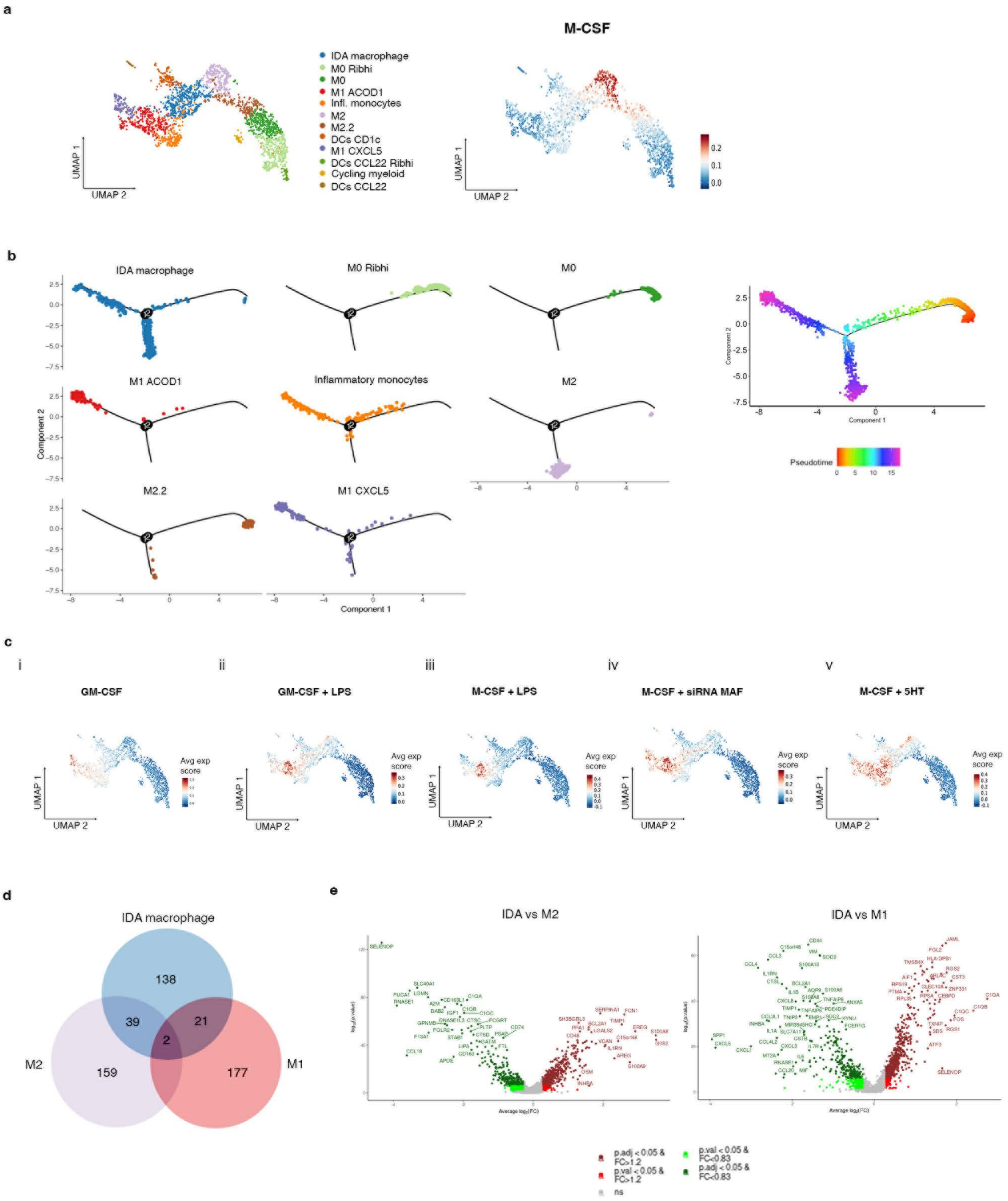

a
M-CSF
IDA macrophage
M0 Ribhi
M0
M1 ACOD1
Infl. monocytes
M2
M2.2
DCs CD1c
M1 CXCL5
DCs CCL22 Ribhi
Cycling myeloid
DCs CCL22
UMAP 1
UMAP 2
b
IDA macrophage
M0 Ribhi
M0
M1 ACOD1
Inflammatory monocytes
M2
M2.2
M1 CXCL5
Component 1
Component 2
Pseudotime
c
i
ii
iii
iv
v
GM-CSF
GM-CSF + LPS
M-CSF + LPS
M-CSF + siRNA MAF
M-CSF + 5HT
Avg exp score
d
IDA macrophage
M2
M1
138
39
21
2
159
177
e
IDA vs M2
IDA vs M1
p.adj < 0.05 & FC>1.2
p.val < 0.05 & FC>1.2
ns
p.val < 0.05 & FC<0.83
p.adj < 0.05 & FC<0.83

Supplementary Figure 5. Inflammation-dependent alternative (IDA) macrophages show a unique signature compared to M2 and M1 macrophages. a, UMAP showing monocyte, macrophage and dendritic cell clusters identified using scRNA-seq analysis of HC, UC and CD colonic biopsies (left panel). Representation of overlapping signatures (average expression score) of upregulated genes in in vitro M-CSF derived macrophages (Cuevas VD et al., 2022) on our macrophage cell UMAP (right panel). **b**, Pseudo-time trajectory analysis of monocytes and macrophage populations in our scRNA-seq data. **c**, Representation of overlapping signatures (average expression score) of in vitro i) GM-CSF-derived macrophages (Cuevas VD et al., 2022), ii) GM-CSF-derived macrophages stimulated with LPS (Cuevas VD et al., 2022), iii) M-CSF-derived macrophages stimulated with LPS (Cuevas VD et al., 2022), iv) upregulated genes of M-CSF-derived macrophages inhibited with MAF siRNA (Vega MA et al, 2020) and v) upregulated genes of M-CSF-derived macrophages stimulated with 5-HT (serotonin) (Nieto C et al, 2020; Domínguez-Soto Á et al, 2017) in our macrophage cell UMAP. **d**, Venn diagram showing the overlap between the top 200 markers of each macrophage population (IDA, M2 (M2 & M2.2) and M1 (M1 ACOD1 & M1 CXCL5) macrophages in the scRNA-seq cohort. **e**, Volcano plot showing differentially expressed genes (DEGs) by scRNA-seq between IDA and M2 or M1 macrophages. A two-sided Wilcoxon rank sum test was applied. Genes upregulated in IDA macrophages are shown in dark (false discovery rate adjusted p value<0.05, FC>1.2) or light red (nominal p value<0.05, FC>1.2). Genes downregulated in IDA are shown in dark (false discovery rate adjusted p value<0.05, FC<0.83) or light green (nominal p value<0.05, FC<0.83). For each gene, the fold-change (FC) and -log10 (p value) are shown. Source data are provided as a Source Data file.

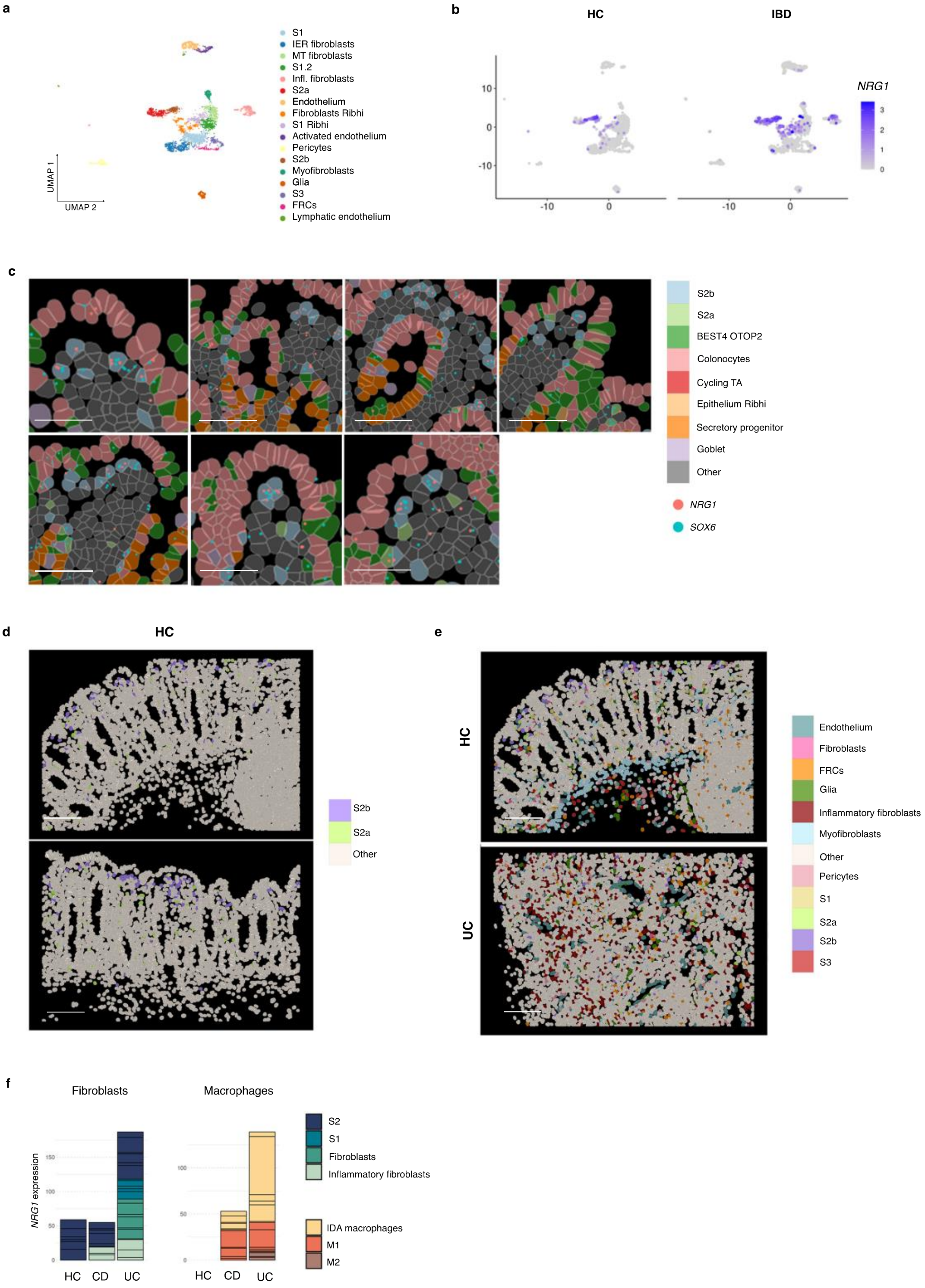

Supplementary Figure 6. Stromal cell populations analyzed by single-cell RNA-seq (scRNA-seq) and Spatial Molecular Imaging (SMI). a, UMAP of stromal clusters observed by scRNA-seq cohort samples. **b,** NRG1 expression in healthy and IBD stromal subsets in scRNA-seq data. c, CosMxTM SMI spatial analysis of S2a and S2b pericryptal fibroblasts showing expression of NRG1 and SOX6 (S2 marker) in representative images of a healthy colon. S2b fibroblasts localize at the most apical area. Scale bar = 500 µm. **d,** S2a and S2b differential spatial distribution showed by CosMxTM SMI in 2 representative FoVs from healthy colon. Scale bar = 500 µm. **e,** Spatial

localization of stromal cells observed by CosMxTM SMI in representative healthy and inflamed UC colonic mucosa. Scale bar = 500 µm**. f,** NRG1 expression in the fibroblast and macrophage compartments in HC and IBD colonic tissues according to scRNA-seq data. Source data are provided as a Source Data file.

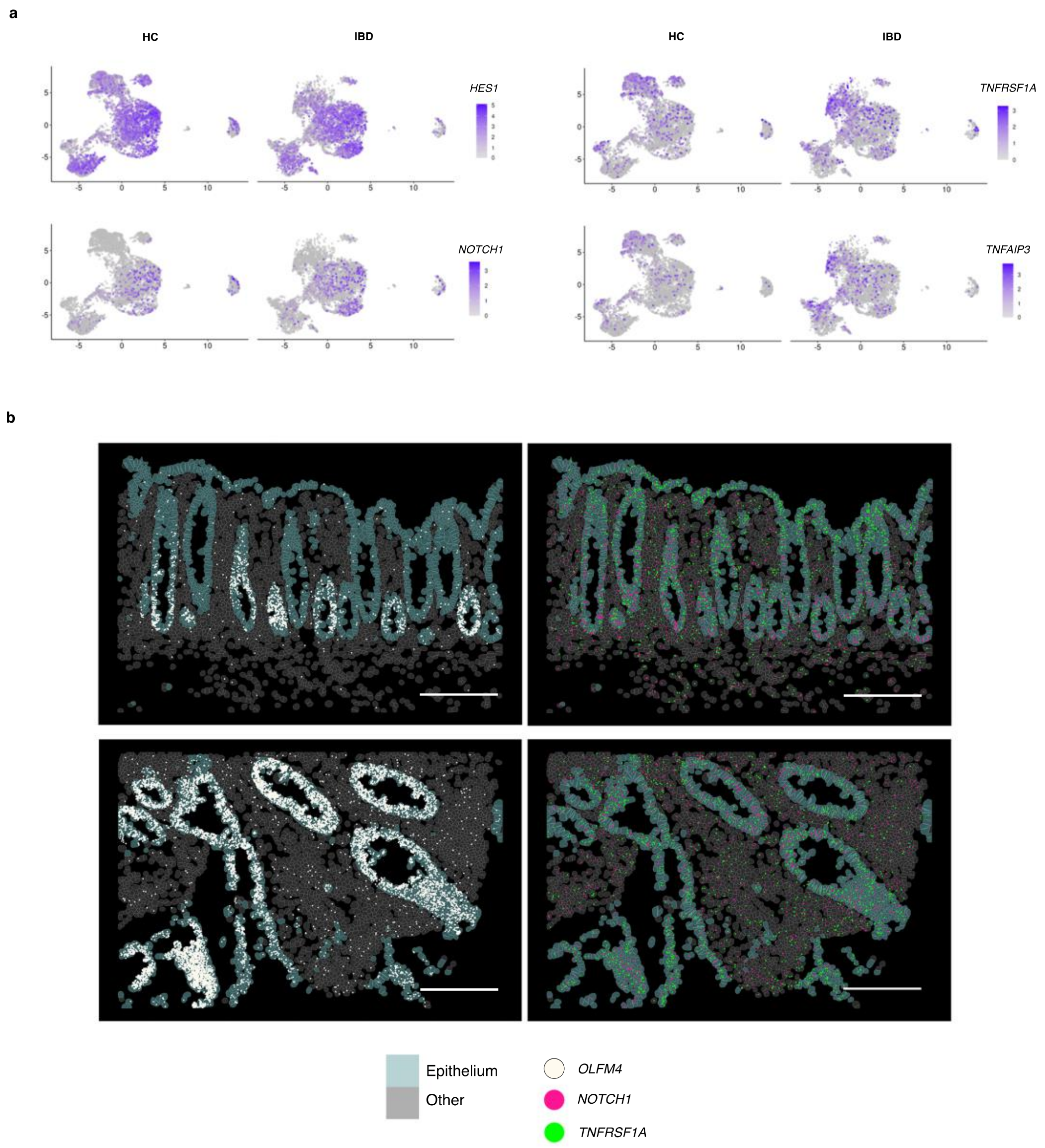

Supplementary Figure 7. **a**, *HES1, NOTCH1, TNFRSF1A, TNFAIP3* expression in healthy and IBD epithelial subsets in scRNA-seq data. **b**, Expression of *OLFM4 (white)*, *NOTCH1(pink)*, and *TNFRSF1A (green)* within epithelium of representative FoVs of a HC and an UC patient analyzed by CosMx^{TM} SMI. Representative of 9 independent biological replicates. Dots represent mRNA molecules. Scale bar = 200 µm. Source data are provided as a Source Data file.

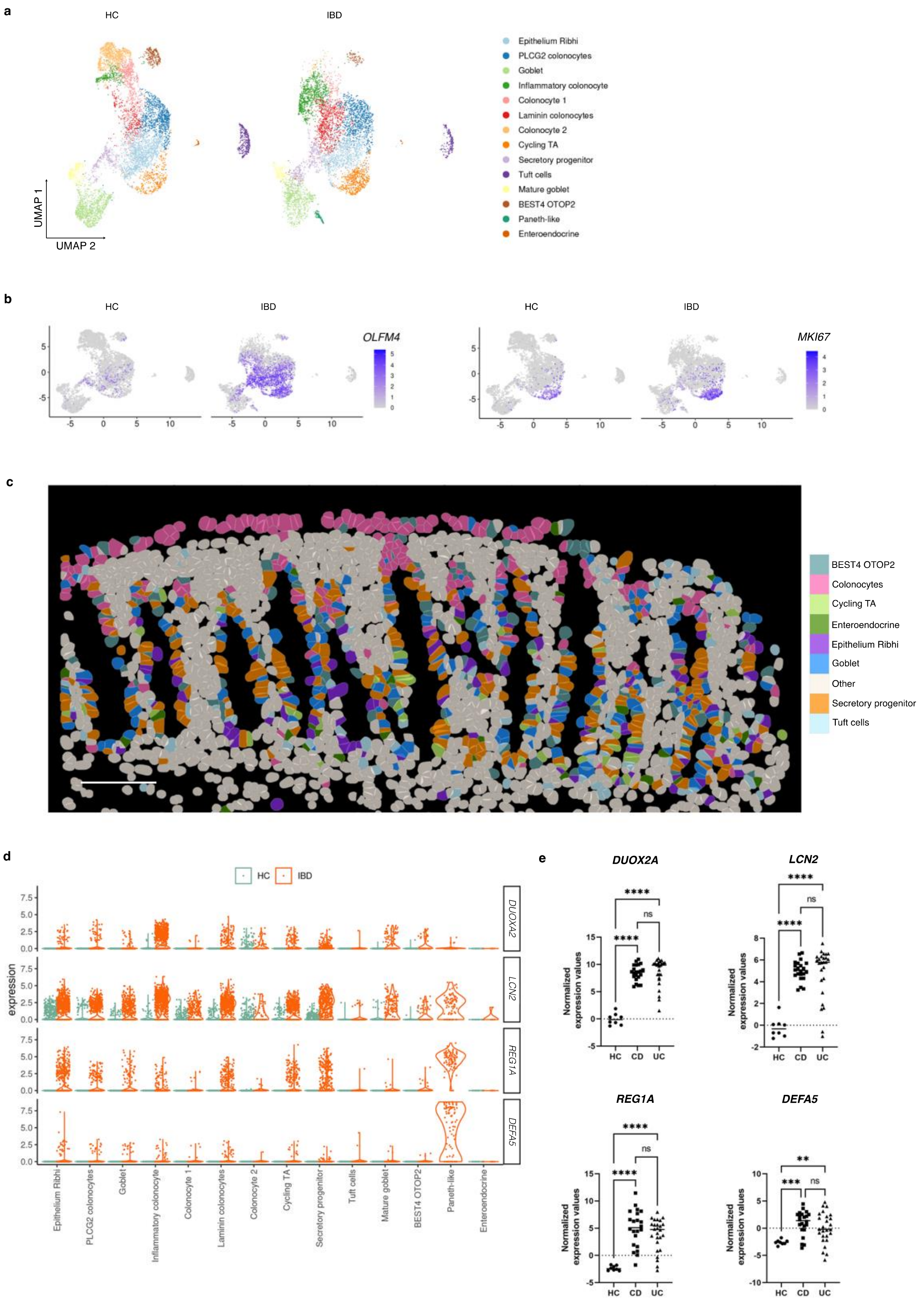

a
HC
IBD
Epithelium Ribhi
PLCG2 colonocytes
Goblet
Inflammatory colonocyte
Colonocyte 1
Laminin colonocytes
Colonocyte 2
Cycling TA
Secretory progenitor
Tuft cells
Mature goblet
BEST4 OTOP2
Paneth-like
Enteroendocrine
UMAP 1
UMAP 2
b
OLFM4
MKI67
c
BEST4 OTOP2
Colonocytes
Cycling TA
Enteroendocrine
Epithelium Ribhi
Goblet
Other
Secretory progenitor
Tuft cells
d
HC
IBD
expression
DUOXA2
LCN2
REG1A
DEFA5
Epithelium Ribhi
PLCG2 colonocytes
Goblet
Inflammatory colonocyte
Colonocyte 1
Laminin colonocytes
Colonocyte 2
Cycling TA
Secretory progenitor
Tuft cells
Mature goblet
BEST4 OTOP2
Paneth-like
Enteroendocrine
e
DUOX2A
LCN2
REG1A
DEFA5
Normalized expression values
HC
CD
UC
****
***
**
ns

Supplementary Figure 8. **Epithelial cell populations analyzed by single-cell RNA-seq (scRNA-seq) and Spatial Molecular Imaging (SMI). a**, UMAP of colonic epithelial clusters observed by scRNA-seq in healthy and IBD mucosa (cohort 1). **b**, UMAP showing the expression of *OLFM4* and *MKI67* in the epithelial compartment from healthy control (HC) and IBD tissues (UC and CD) by scRNA-seq. **c**, CosMx^{TM} SMI image of a representative Field of View (FoV) of an HC from 3 independent biological replicates showing epithelial subsets annotated based on scRNA-seq data. Scale bar = 100 μm. **d**, Violin plots showing the expression (y-axis) of inflammatory markers in epithelial clusters (x-axis) from HC (n=6) and IBD colons (n=12). **e**, Expression of specific inflammatory epithelial cell markers in HC (n=8), active CD (n=22) and active UC (n=26) patients using bulk biopsy RNA-seq data. Ordinary one-way ANOVA test correcting for multiple comparisons (Benjamini-Yekutieli) was applied. Each sample is represented as a dot and the median value of gene expression per patient group is shown as a line. p<0,01 (**), p<0,001(***), p<0,0001(****), ns: not significant. Source data are provided as a Source Data file.

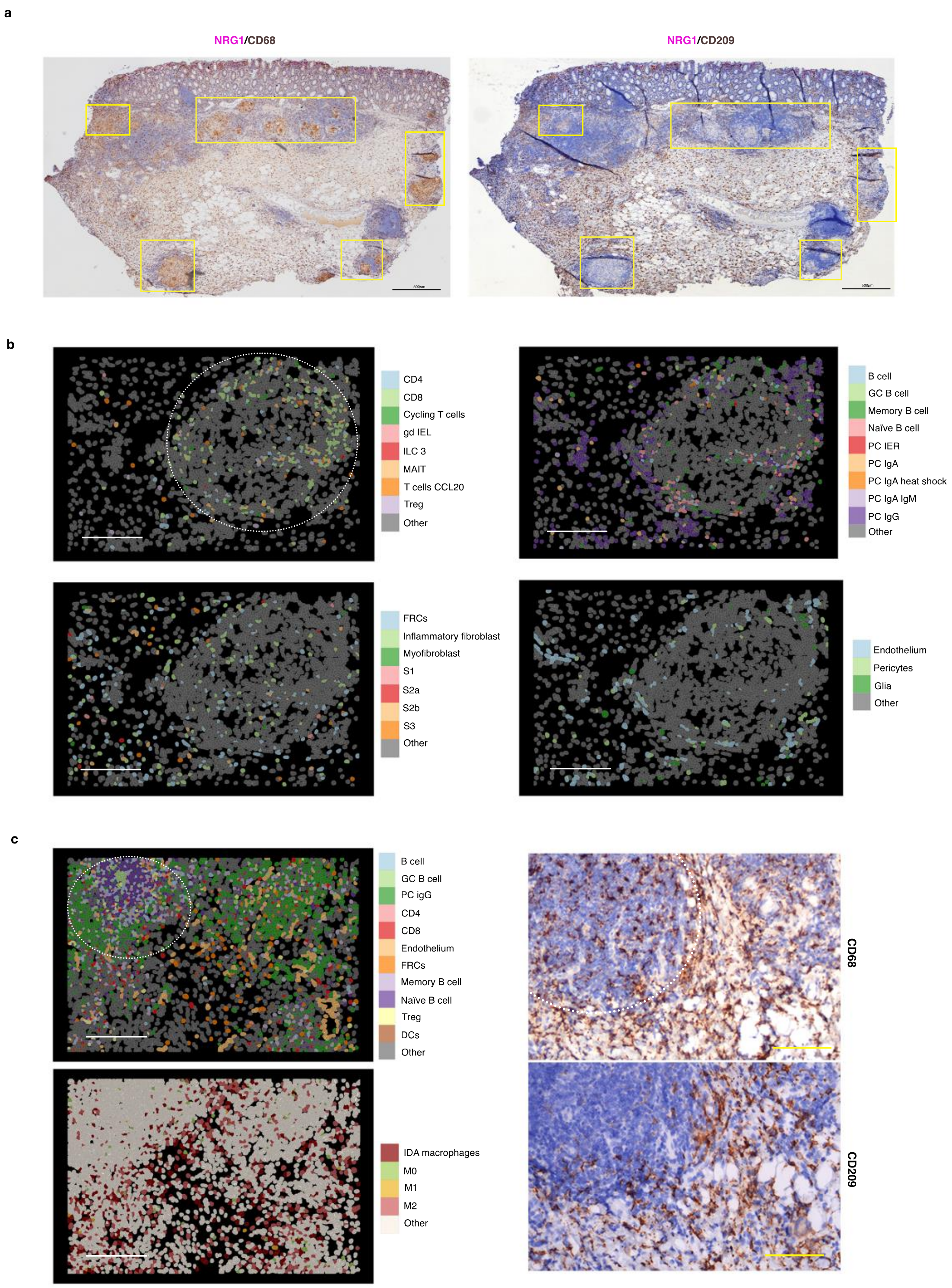

Supplementary Figure 9. Cellular annotation of all cell types present in Crohn's disease (CD)-associated granuloma. a, Colonic tissue of a CD patient (CD b) containing multiple granulomas indicated by yellow squares. Tissue is stained by *in situ* hybridization for *NRG1* combined with immunostaining for CD68 or CD209. Representative of 3 biological replicates. **b,** CosMx^{TM} SMI images showing diverse cell types (stroma cells, T cells, B and plasma cells) present in the granulomas and surrounding area of the analyzed sample. Granuloma is shown by a dotted circle. Scale bar = 200 µm **c,** Left panels show cell labelling by CosMx^{TM} SMI of a section containing a lymphoid aggregate from the same CD patient. Scale bar = 200 µm The right panels show expression of CD68 and CD209 by immunohistochemistry in sequential sections.

Scale bar= 100 µm. The lymphoid aggregate is shown by dotted circle and is mainly constituted by B cells, abundant plasma cells and some macrophages (M0, M2, M1 and IDA macrophages) are found within the granuloma and more abundantly in the surrounding area. Source data are provided as a Source Data file.

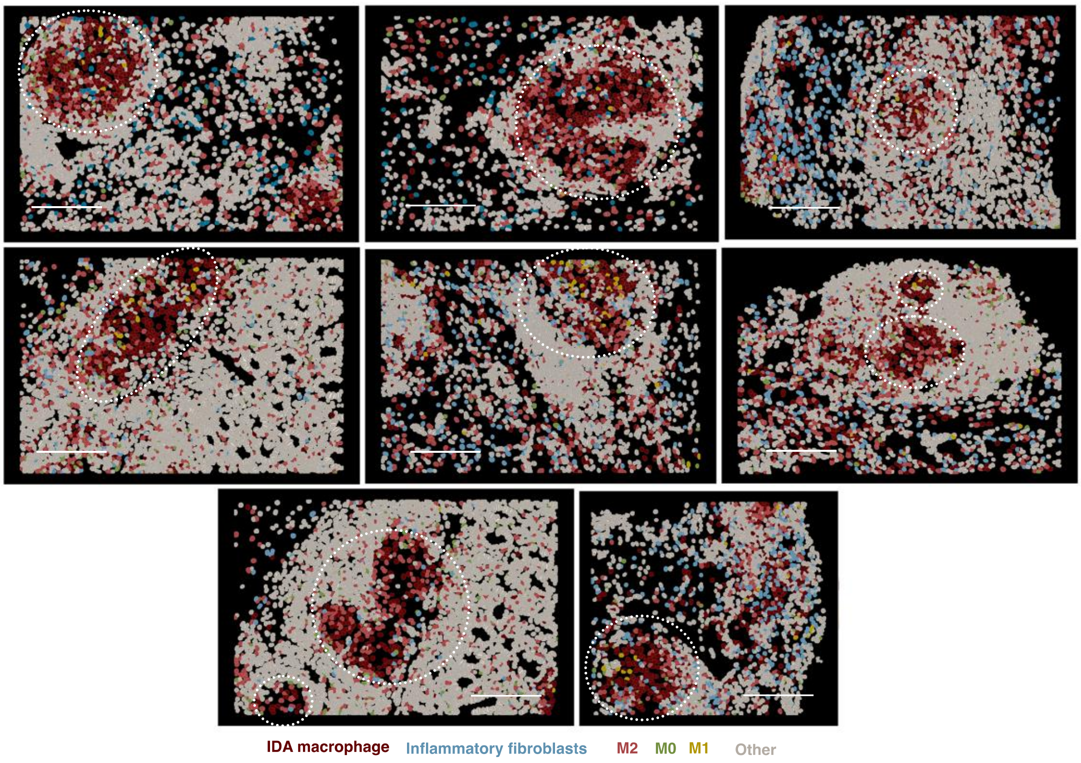

Supplementary Figure 10. Spatial visualization of inflammatory fibroblasts in inflammatory bowel disease (IBD). CosMx SMI visualization of inflammatory fibroblasts within granulomas and the surrounding areas in the CD patient that contained multiple granulomas (CD b). Granulomas are indicated by dotted circles. As shown in Figure 4, granulomas contain abundant IDA macrophages. Inflammatory fibroblasts (light blue) can be found within granulomas and in other adjacent areas. Scale bar = 200 µm. Source data are provided as a Source Data file

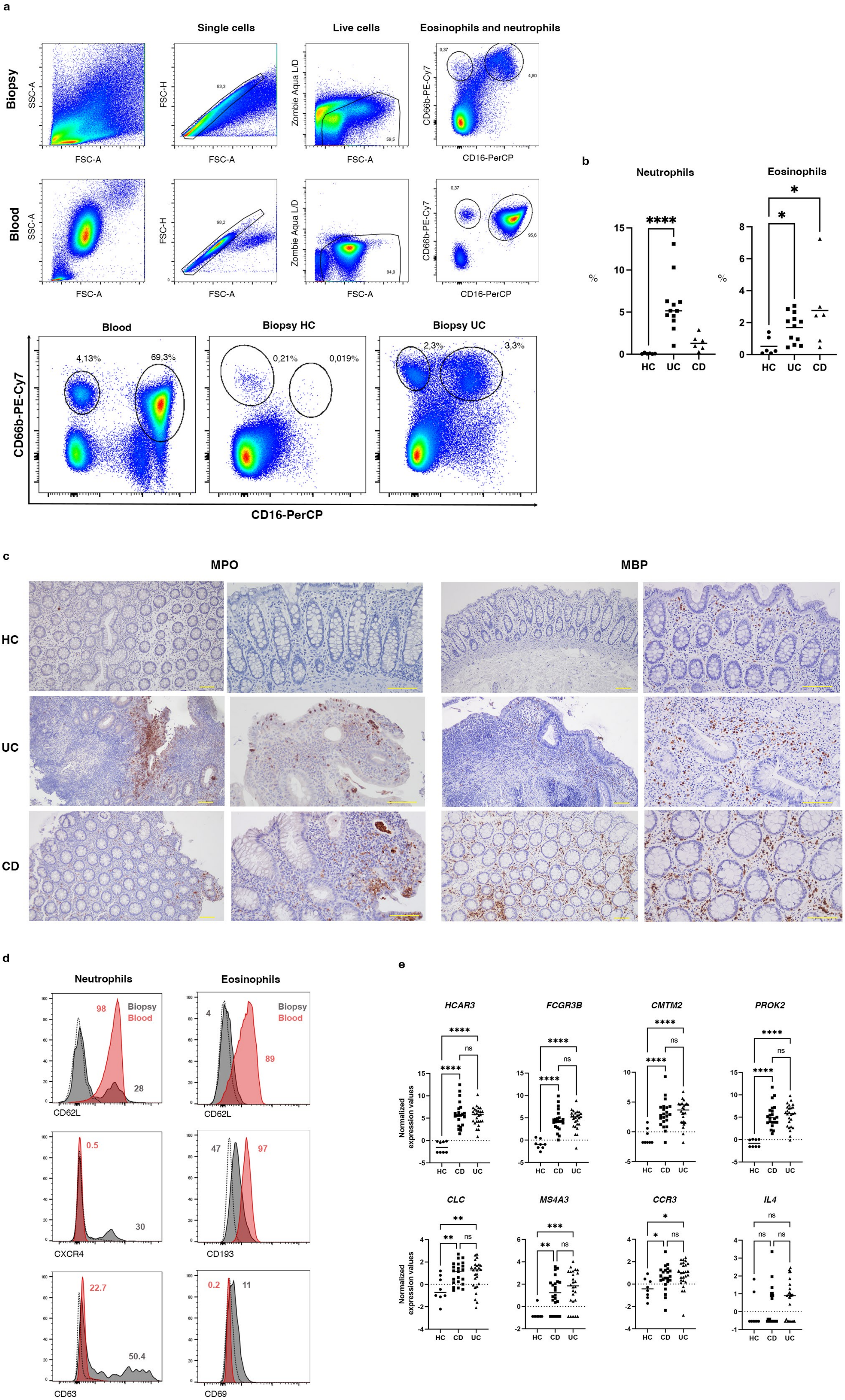
a
Single cells
Live cells
Eosinophils and neutrophils
Biopsy
Blood
SSC-A
FSC-A
FSC-H
Zombie Aqua L/D
CD66b-PE-Cy7
CD16-PerCP
83,3
59,5
0,37
4,80
98,2
94,9
95,6
Blood
Biopsy HC
Biopsy UC
4,13%
69,3%
0,21%
0,019%
2,3%
3,3%
b
Neutrophils
Eosinophils
%
****
*
HC
UC
CD
c
MPO
MBP
HC
UC
CD
d
Neutrophils
Eosinophils
Biopsy
Blood
CD62L
CXCR4
CD63
CD193
CD69
98
28
4
89
0.5
30
47
97
22.7
50.4
0.2
11
e
HCAR3
FCGR3B
CMTM2
PROK2
CLC
MS4A3
CCR3
IL4
Normalized expression values
****
***
**
*
ns
HC
CD
UC

Supplementary Figure 11. **Analysis of neutrophils and eosinophils in inflammatory bowel disease (IBD). a**, Flow cytometry gating strategy to detect eosinophils and neutrophils in IBD blood and colonic biopsies. Numbers represent the percentages of neutrophils ($CD66b^{+}$ $CD16b^{+}$) and eosinophils ($CD66b^{+}$ $CD16b^{-}$) in the sample displayed as representative of all samples analyzed. **b**, Percentage of neutrophils ($CD66b^{+}$ $CD16b^{+}$) and eosinophils ($CD66b^{+}$ $CD16b^{-}$) in colonic biopsies from healthy controls (HC, n=6), active CD (n=6) and active UC (n=12) colonic samples analyzed by flow cytometry. Kruskal–Wallis test and correcting for multiple comparisons by controlling the false discovery rate (Benjamini-Yekutieli). Each sample is represented as a dot and the median value as a line. $p<0.0001$ (****), $p=0.0233$ for HC vs UC and $p=0.0212$ for HC vs CD comparative for eosinophils **c**, Immunostaining for myeloperoxidase (MPO, marker neutrophils) and myelin basic protein (MBP, marker of eosinophils) in representative HC, (of 9 biological replicates), active UC (representative of 6 biological replicates) and active CD (representative of 5 biological replicates) colonic tissues (scale bar = 100 µm). **d**, Protein expression detected by flow cytometry in neutrophils (one representative sample is shown from12 colonic and 6 blood samples) and eosinophils (one representative sample shown from 15 colonic and 5 blood samples) from blood and biopsies of IBD patients. Numbers show the percentage of positive cells for the protein in blood (red) and biopsy (grey)Histogram for the corresponding isotype control in the biopsy sample is shown as a dashed line. **e**, Bulk colonic biopsy RNA-seq expression of neutrophil and eosinophil-specific markers in HC (n=8), active CD (n=22) and UC (n=26) patients. Ordinary one-way ANOVA test correcting for multiple comparisons by controlling the false discovery rate (Benjamini-Yekutieli). Each sample is represented as a dot and the median value as a line. $p<0,05$(*), $p<0,01$ (**), $p<0,001$(***), $p<0,0001$(****), ns: not significant. Source data are provided as a Source Data file.

Supplementary Table 1. Clinical and demographic information of individuals included in the study.

Endoscopic biopsies (cohort 1) or surgical resection pieces (cohort 2) were processed for single cell RNAseq or spatial molecular imaging (SMI), respectively. Na: non applicable. Nd: no data.
* Score could not be calculated due to incomplete colonoscopy preparation.

	Age	Sex	Disease	Biopsy/surgical resection area	Disease location/Extension	Disease duration (years)	Treatment	Total CDEIS	Partial CDEIS	Endoscopic Mayo score
**Cohort 1. scRNA-seq analysis (scRNA-seq)**										
**HC 1**	61	Male	na	Sigmoid colon	na	na	na	na	na	na
**HC 2**	63	Male	na	Sigmoid colon	na	na	na	na	na	na
**HC 3**	68	Female	na	Sigmoid colon	na	na	na	na	na	na
**HC 4**	65	Male	na	Sigmoid colon	na	na	na	na	na	na
**HC 5**	51	Male	na	Sigmoid colon	na	na	na	na	na	na
**HC 6**	66	Female	na	Sigmoid colon	na	na	na	na	na	na
**CD 1**	49	Female	CD	Ascending colon	Ileocolonic	15	Oral prednisone	16.61	15.5	na
**CD 2**	45	Male	CD	Sigmoid colon	Ileocolonic	14	Adalimumab	24.24	12.1	na
**CD 3**	23	Female	CD	Descending colon	Ileocolonic	6	na	6.6	12	na
**CD 4**	56	Male	CD	Sigmoid colon	Colonic	5	Mesalazine, tacrolimus	25.4	31	na
**CD 5**	21	Male	CD	Sigmoid colon	Ileocolonic	5	Vedolizumab	13	20	na
**CD 6**	22	Male	CD	Sigmoid colon	Ileocolonic	10	Ustekinumab	4.4	9.5	na
**UC 1**	48	Male	UC	Sigmoid colon	Descending colon	3	Oral prednisone, ciprofloxacin, metronidazole	na	na	3
**UC 2**	43	Male	UC	Transvers colon	Descending colon	25	Mesalazine	na	na	3
**UC 3**	33	Female	UC	Rectum	Rectum	9	Azathioprine, Prednisone	na	na	3
**UC 4**	46	Female	UC	Sigmoid colon	Pancolitis	5	Vedolizumab	na	na	3
**UC 5**	56	Male	UC	Rectum	Rectum	6	Vedolizumab, mesalazine	na	na	3
**UC 6**	51	Female	UC	Sigmoid colon	Descending colon	14	Mesalazine	na	na	3
**Cohort 2. Spatial molecular Imagining (SMI CosMx, Nanostring)**										
**HC a**	59	Female	na	Sigmoid colon	na	na	na	na	na	na

**HC b**	61	Female	na	Descending colon	na	na	na	na	na	na
**HC c**	69	Male	na	Sigmoid colon	na	na	na	na	na	na
**CD a**	45	Male	CD	Sigmoid colon	Ileocolonic	25	Ustekinumab	*	*	na
**CD b**	42	Female	CD	Transverse colon	Colonic	28	Cyclosporine	0	8	na
**CD c**	38	Female	CD	Transverse colon	Ileocolonic	19	Ciprofloxacin, metronidazole	14	0	na
**UC a**	69	Male	UC	Transverse colon	Pancolitis	21	Infliximab	na	na	3
**UC b**	44	Female	UC	Sigmoid colon	Pancolitis	5	Tofacitinib	na	na	3
**UC c**	40	Male	UC	Sigmoid colon	Pancolitis	5	Tofacitinib	na	na	3
**Cohort 3. Bulk RNA-seq**										
**Bulk 1**	52	Female	HC	Sigmoid colon	na	na	na	na	na	na
**Bulk 2**	51	Male	HC	Sigmoid colon	na	na	na	na	na	na
**Bulk 3**	68	Male	HC	Sigmoid colon	na	na	na	na	na	na
**Bulk 4**	51	Female	HC	Sigmoid colon	na	na	na	na	na	na
**Bulk 5**	27	Male	HC	Sigmoid colon	na	na	na	na	na	na
**Bulk 6**	49	Female	HC	Sigmoid colon	na	na	na	na	na	na
**Bulk 7**	32	Female	HC	Sigmoid colon	na	na	na	na	na	na
**Bulk 8**	52	Female	HC	Sigmoid colon	na	na	na	na	na	na
**Bulk 9**	33	Male	CD	Sigmoid colon	Colonic	13	Immunosupressor	10.4	16	na
**Bulk 10**	47	Male	CD	Descending colon	Colonic	24	Immunosupressor	19	20	na
**Bulk 11**	53	Female	CD	Rectum	Ileocolonic	28	No treatment	20.5	34	na
**Bulk 12**	39	Female	CD	Descending colon	Ileocolonic	23	Immunosupressor	6.2	31	na
**Bulk 13**	67	Male	CD	Sigmoid colon	Colonic	7	Steroids	12	30	na
**Bulk 14**	35	Female	CD	Descending colon	Ileocolonic	7	No treatment	17.75	20.5	na
**Bulk 15**	64	Female	CD	Sigmoid colon	Ileocolonic	7	Steroids	19.75	31	na
**Bulk 16**	35	Male	CD	Ascending colon	Ileocolonic	27	Immunosupressor	12.6	12	na
**Bulk 17**	31	Female	CD	Descending colon	Colonic	6	No treatment	13.5	19	na
**Bulk 18**	33	Male	CD	Sigmoid colon	Colonic	21	Immunosupressor	6.8	17	na
**Bulk 19**	56	Female	CD	Sigmoid colon	Colonic	14	ND	7.88	9.5	na
**Bulk 20**	22	Male	CD	Descending colon	Colonic	9	Immunosupressor	22.6	32	na
**Bulk 21**	42	Male	CD	Transverse colon	Ileocolonic	25	ND	4.67	5	na
**Bulk 22**	56	Female	CD	Rectum	Ileocolonic	29	ND	15.88	29.5	na
**Bulk 23**	44	Female	CD	Rectum	Colonic	12	Immunosupressor	10.4	26	na

**Bulk 24**	37	Male	CD	Ascending colon	Ileocolonic	23	Immunosupressor	24	30	na
**Bulk 25**	20	Male	CD	Rectum	Colonic	14	Immunosupressor	12.8	32	na
**Bulk 26**	24	Female	CD	Sigmoid colon	Colonic	9	Steroids	5.6	10	na
**Bulk 27**	60	Female	CD	Transverse colon	Colonic	33	Immunosupressor	4.67	14	na
**Bulk 28**	29	Male	CD	Ascending colon	Colonic	11	Immunosupressor	19.6	17	na
**Bulk 29**	48	Male	CD	Sigmoid colon	Ileocolonic	32	Immunosupressor	35.5	34	na
**Bulk 30**	41	Male	CD	Rectum	Colonic	16	Immunosupressor	8.25	16.5	na
**Bulk 31**	57	Female	UC	Sigmoid colon	Pancolitis	11	Immunosupressor	na	na	3
**Bulk 32**	43	Male	UC	Sigmoid colon	Pancolitis	30	Immunosupressor, Steroids	na	na	2
**Bulk 33**	56	Male	UC	Sigmoid colon	Pancolitis	25	Immunosupressor	na	na	3
**Bulk 34**	61	Female	UC	Descending colon	Left-sided colitis	38	Immunosupressor	na	na	3
**Bulk 35**	31	Male	UC	Sigmoid colon	Pancolitis	9	Immunosupressor, Mesalazine	na	na	3
**Bulk 36**	62	Male	UC	Sigmoid colon	Left-sided colitis	13	Steroids	na	na	2
**Bulk 37**	44	Male	UC	Sigmoid colon	Pancolitis	24	Immunosupressor	na	na	3
**Bulk 38**	28	Male	UC	Sigmoid colon	Pancolitis	7	Immunosupressor	na	na	3
**Bulk 39**	29	Female	UC	Sigmoid colon	Pancolitis	10	Immunosupressor, Steroids	na	na	3
**Bulk 40**	55	Male	UC	Sigmoid colon	Pancolitis	30	Immunosupressor	na	na	3
**Bulk 41**	48	Male	UC	Rectum	Proctitis	37	Steroids	na	na	2
**Bulk 42**	37	Female	UC	Rectum	proctitis	25	ND	na	na	2
**Bulk 43**	19	Male	UC	Ascending colon	Pancolitis	9	ND	na	na	2
**Bulk 44**	31	Male	UC	Sigmoid colon	Pancolitis	11	Immunosupressor	na	na	3
**Bulk 45**	37	Female	UC	Rectum	Left-sided colitis	14	Immunosupressor, Steroids	na	na	3
**Bulk 46**	35	Male	UC	Sigmoid colon	Left-sided colitis	14	Steroids, Mesalazine	na	na	3
**Bulk 47**	69	Male	UC	Sigmoid colon	Left-sided colitis	12	Immunosupressor	na	na	3
**Bulk 48**	25	Female	UC	Sigmoid colon	Pancolitis	10	Immunosupressor	na	na	2
**Bulk 49**	38	Male	UC	Sigmoid colon	Left-sided colitis	14	Immunosupressor	na	na	3
**Bulk 50**	50	Male	UC	Sigmoid colon	Pancolitis	29	Mesalazine	na	na	3
**Bulk 51**	41	Male	UC	Sigmoid colon	proctitis	14	No treatment	na	na	3
**Bulk 52**	39	Female	UC	Sigmoid colon	Left-sided colitis	22	No treatment	na	na	3
**Bulk 53**	50	Male	UC	Sigmoid colon	na	29	No treatment	na	na	na

**Bulk 54**	40	Female	UC	Sigmoid colon	na	22	Stop anti-TNF (Golimumab)	na	na	na
**Bulk 55**	59	Female	UC	Rectum	Ileocolonic	31	Immunosupressor	na	na	na
**Bulk 56**	27	Female	UC	Sigmoid colon	Colonic	7	Immunosupressor	na	na	na

Supplementary Table 2. DEGs genes between macrophage populations in scRNA-seq data.Two-sided Wilcoxon rank sum test was performed.

p_val	avg_log2FC	pct.1	pct.2	p_val_adj	gene	sign	comp
2,87E-102	3,36895329	0,66	0,03	9,50E-98	FOSB	UPP	M2_vs_M0
8,70E-88	3,27418749	0,653	0,06	2,88E-83	FOS	UPP	M2_vs_M0
1,09E-92	3,17864465	0,57	0,01	3,60E-88	NR4A2	UPP	M2_vs_M0
5,05E-107	2,91914496	0,69	0,04	1,67E-102	DUSP1	UPP	M2_vs_M0
3,60E-54	2,90968851	0,451	0,05	1,19E-49	CXCL8	UPP	M2_vs_M0
1,07E-101	2,89235151	0,703	0,06	3,56E-97	NEAT1	UPP	M2_vs_M0
5,90E-95	2,72104268	0,639	0,04	1,95E-90	GPR183	UPP	M2_vs_M0
2,82E-86	2,71665588	0,626	0,05	9,35E-82	CHMP1B	UPP	M2_vs_M0
2,35E-77	2,70808414	0,623	0,08	7,79E-73	LMNA	UPP	M2_vs_M0
1,61E-93	2,67691602	0,599	0,02	5,34E-89	ZNF331	UPP	M2_vs_M0
8,93E-98	2,51870537	0,647	0,03	2,96E-93	RGS1	UPP	M2_vs_M0
1,20E-80	2,50393164	0,56	0,03	3,97E-76	CD83	UPP	M2_vs_M0
3,50E-82	2,48404697	0,668	0,08	1,16E-77	RNASE1	UPP	M2_vs_M0
5,26E-101	2,47788536	0,732	0,07	1,74E-96	ARL4C	UPP	M2_vs_M0
1,06E-93	2,45091631	0,735	0,09	3,50E-89	ZFP36L1	UPP	M2_vs_M0
3,50E-110	2,4265556	0,894	0,15	1,16E-105	LGMN	UPP	M2_vs_M0
2,77E-65	2,38237961	0,488	0,03	9,17E-61	TNFAIP3	UPP	M2_vs_M0
6,68E-71	2,37344686	0,472	0,02	2,21E-66	NR4A3	UPP	M2_vs_M0
2,13E-83	2,35968557	0,692	0,09	7,05E-79	JUNB	UPP	M2_vs_M0
1,32E-80	2,35233589	0,57	0,03	4,37E-76	IER3	UPP	M2_vs_M0
3,30E-83	2,32609181	0,586	0,04	1,09E-78	PLAUR	UPP	M2_vs_M0
6,53E-90	2,29995424	0,653	0,05	2,16E-85	KLF6	UPP	M2_vs_M0
2,60E-62	2,29121061	0,403	0,01	8,63E-58	CXCL2	UPP	M2_vs_M0
6,52E-54	2,29036367	0,464	0,05	2,16E-49	CCL3	UPP	M2_vs_M0
1,10E-66	2,25087415	0,48	0,03	3,66E-62	ATF3	UPP	M2_vs_M0
3,89E-77	2,17159145	0,477	0,01	1,29E-72	KLF4	UPP	M2_vs_M0
2,00E-78	2,15356156	0,517	0,02	6,64E-74	INSIG1	UPP	M2_vs_M0
3,56E-37	2,15281011	0,355	0,05	1,18E-32	CCL4	UPP	M2_vs_M0
1,09E-76	2,14395918	0,849	0,29	3,62E-72	SRGN	UPP	M2_vs_M0
3,62E-91	2,13323155	0,679	0,06	1,20E-86	ZFP36	UPP	M2_vs_M0
1,38E-104	2,10616294	0,806	0,09	4,57E-100	STAB1	UPP	M2_vs_M0
1,83E-89	2,08852655	0,703	0,08	6,05E-85	MCL1	UPP	M2_vs_M0
6,12E-81	2,0814556	0,615	0,05	2,03E-76	F13A1	UPP	M2_vs_M0
2,40E-90	2,04529055	0,607	0,03	7,96E-86	ICAM1	UPP	M2_vs_M0
1,36E-82	1,97340164	0,607	0,04	4,51E-78	RGS2	UPP	M2_vs_M0
9,14E-74	1,90814067	0,459	0,01	3,03E-69	TIPARP	UPP	M2_vs_M0
4,02E-32	1,89900319	0,3	0,04	1,33E-27	CCL4L2	UPP	M2_vs_M0
1,89E-65	1,86838029	0,586	0,07	6,25E-61	NFKBIA	UPP	M2_vs_M0
2,33E-80	1,85210647	0,509	0,01	7,70E-76	C5AR1	UPP	M2_vs_M0
3,12E-56	1,84723375	0,355	0	1,03E-51	PTGS2	UPP	M2_vs_M0
1,10E-75	1,84427002	0,557	0,04	3,64E-71	SGK1	UPP	M2_vs_M0
4,75E-31	1,84225097	0,345	0,06	1,57E-26	IL1B	UPP	M2_vs_M0
2,09E-34	1,84015847	0,353	0,05	6,91E-30	CCL18	UPP	M2_vs_M0
8,08E-74	1,81910017	0,488	0,02	2,68E-69	FOSL2	UPP	M2_vs_M0
4,19E-79	1,80512298	0,653	0,07	1,39E-74	HERPUD1	UPP	M2_vs_M0
1,60E-67	1,77864594	0,599	0,08	5,29E-63	ATP1B3	UPP	M2_vs_M0

1,16E-70	1,77556836	0,523	0,03	3,83E-66	GADD45B	UPP	M2_vs_M0
2,42E-68	1,7579445	0,544	0,05	8,02E-64	TFRC	UPP	M2_vs_M0
2,95E-51	1,75721374	0,419	0,04	9,79E-47	JUN	UPP	M2_vs_M0
1,46E-67	1,726245	0,438	0,01	4,83E-63	SLC2A3	UPP	M2_vs_M0
3,66E-67	1,72556592	0,594	0,07	1,21E-62	CREM	UPP	M2_vs_M0
3,69E-49	1,72344071	0,326	0,01	1,22E-44	CXCL3	UPP	M2_vs_M0
9,87E-26	1,70703927	0,207	0,01	3,27E-21	CCL3L1	UPP	M2_vs_M0
2,85E-88	1,6960717	0,782	0,11	9,43E-84	A2M	UPP	M2_vs_M0
7,79E-66	1,68412506	0,43	0,01	2,58E-61	HBEGF	UPP	M2_vs_M0
5,05E-35	1,67880519	0,247	0,01	1,67E-30	THBS1	UPP	M2_vs_M0
1,37E-59	1,67386151	0,711	0,16	4,53E-55	JUND	UPP	M2_vs_M0
3,82E-74	1,64857904	0,875	0,23	1,26E-69	MS4A7	UPP	M2_vs_M0
5,72E-80	1,61704465	0,984	0,44	1,89E-75	PSAP	UPP	M2_vs_M0
1,46E-86	1,61037594	0,639	0,05	4,82E-82	PRNP	UPP	M2_vs_M0
1,04E-84	1,6029408	0,605	0,04	3,45E-80	LILRB5	UPP	M2_vs_M0
3,95E-68	1,588576	0,671	0,11	1,31E-63	PNRC1	UPP	M2_vs_M0
1,40E-62	1,5860119	0,684	0,14	4,65E-58	NAMPT	UPP	M2_vs_M0
6,59E-72	1,57956618	0,955	0,38	2,18E-67	CTSB	UPP	M2_vs_M0
3,76E-90	1,56266073	0,623	0,03	1,25E-85	TNFRSF1B	UPP	M2_vs_M0
4,55E-71	1,54904097	0,509	0,03	1,51E-66	MIDN	UPP	M2_vs_M0
1,01E-59	1,5153818	0,923	0,35	3,34E-55	SLC40A1	UPP	M2_vs_M0
1,02E-63	1,49325553	0,408	0,01	3,38E-59	VEGFA	UPP	M2_vs_M0
8,17E-77	1,48831289	0,658	0,07	2,71E-72	MAFB	UPP	M2_vs_M0
2,95E-79	1,48514825	0,647	0,06	9,78E-75	ZEB2	UPP	M2_vs_M0
7,47E-79	1,48234974	0,687	0,08	2,47E-74	CD163	UPP	M2_vs_M0
1,46E-43	1,45967147	0,422	0,06	4,83E-39	SDS	UPP	M2_vs_M0
4,55E-63	1,45871204	0,493	0,04	1,51E-58	IER2	UPP	M2_vs_M0
8,84E-66	1,45832225	0,918	0,32	2,93E-61	CTSZ	UPP	M2_vs_M0
1,26E-72	1,4544899	0,997	0,53	4,17E-68	SELENOP	UPP	M2_vs_M0
5,89E-71	1,451787	0,602	0,06	1,95E-66	ZFP36L2	UPP	M2_vs_M0
8,23E-91	1,4513529	0,719	0,07	2,73E-86	CD163L1	UPP	M2_vs_M0
3,27E-70	1,44727511	0,942	0,33	1,08E-65	FGL2	UPP	M2_vs_M0
5,41E-77	1,4457747	0,737	0,11	1,79E-72	MRC1	UPP	M2_vs_M0
4,62E-78	1,42555457	0,751	0,11	1,53E-73	MAF	UPP	M2_vs_M0
1,02E-53	1,42436391	0,83	0,41	3,37E-49	MALAT1	UPP	M2_vs_M0
7,53E-60	1,42335098	0,401	0,01	2,49E-55	NFKBIZ	UPP	M2_vs_M0
1,87E-60	1,4209435	0,467	0,03	6,19E-56	NFKB1	UPP	M2_vs_M0
1,72E-61	1,41931054	0,419	0,01	5,69E-57	NR4A1	UPP	M2_vs_M0
9,55E-64	1,41581591	0,517	0,05	3,16E-59	REL	UPP	M2_vs_M0
5,36E-53	1,41038334	0,692	0,16	1,77E-48	GPNMB	UPP	M2_vs_M0
2,92E-75	1,39480745	0,485	0,01	9,68E-71	OTUD1	UPP	M2_vs_M0
3,44E-85	1,3864984	0,7	0,07	1,14E-80	TPP1	UPP	M2_vs_M0
2,90E-67	1,38276901	0,446	0,01	9,61E-63	RHOB	UPP	M2_vs_M0
1,92E-67	1,37550226	0,814	0,19	6,37E-63	MPEG1	UPP	M2_vs_M0
4,47E-79	1,37196697	0,623	0,05	1,48E-74	CD209	UPP	M2_vs_M0
8,56E-76	1,36547746	0,578	0,04	2,84E-71	ABCA1	UPP	M2_vs_M0
2,37E-79	1,36072538	0,491	0,01	7,85E-75	CCNL1	UPP	M2_vs_M0
2,47E-80	1,35963476	0,573	0,03	8,17E-76	CEBPB	UPP	M2_vs_M0
6,00E-49	1,35392113	0,509	0,08	1,99E-44	EZR	UPP	M2_vs_M0
6,84E-35	1,35183444	0,236	0,01	2,26E-30	EGR1	UPP	M2_vs_M0

1,14E-73	1,35117145	0,515	0,02	3,78E-69	AHNAK	UPP	M2_vs_M0
8,07E-76	1,34488953	0,546	0,03	2,67E-71	NRP1	UPP	M2_vs_M0
8,57E-86	1,33334163	0,629	0,04	2,84E-81	IQGAP1	UPP	M2_vs_M0
1,78E-66	1,31473147	0,788	0,16	5,88E-62	LIPA	UPP	M2_vs_M0
3,92E-69	1,31315192	0,721	0,12	1,30E-64	CD81	UPP	M2_vs_M0
2,16E-55	1,30461064	0,695	0,15	7,16E-51	HSPA5	UPP	M2_vs_M0
5,37E-84	1,29931256	0,695	0,07	1,78E-79	SLCO2B1	UPP	M2_vs_M0
1,84E-79	1,29270869	0,7	0,08	6,09E-75	FPR3	UPP	M2_vs_M0
2,21E-65	1,2907878	0,475	0,03	7,31E-61	PPP1R15A	UPP	M2_vs_M0
2,34E-65	1,28774667	0,581	0,07	7,75E-61	BTG1	UPP	M2_vs_M0
4,20E-63	1,28762664	0,408	0,01	1,39E-58	PPP1R15B	UPP	M2_vs_M0
3,03E-63	1,27820277	0,406	0,01	1,00E-58	KDM6B	UPP	M2_vs_M0
1,26E-68	1,27330193	0,488	0,02	4,18E-64	PHACTR1	UPP	M2_vs_M0
1,89E-61	1,26761137	0,538	0,06	6,28E-57	YBX3	UPP	M2_vs_M0
5,73E-61	1,26619307	0,472	0,03	1,90E-56	CDKN1A	UPP	M2_vs_M0
1,17E-61	1,26260782	0,44	0,02	3,86E-57	PDE4B	UPP	M2_vs_M0
1,07E-72	1,25940433	0,496	0,02	3,56E-68	TSC22D3	UPP	M2_vs_M0
5,04E-62	1,25358886	0,822	0,19	1,67E-57	DAB2	UPP	M2_vs_M0
5,25E-78	1,24789204	0,642	0,06	1,74E-73	AHR	UPP	M2_vs_M0
4,63E-42	1,24605843	0,305	0,01	1,54E-37	HES1	UPP	M2_vs_M0
4,12E-45	1,23146712	0,342	0,02	1,37E-40	DUSP2	UPP	M2_vs_M0
1,30E-61	1,22528063	0,48	0,04	4,30E-57	DUSP6	UPP	M2_vs_M0
6,20E-52	1,2191801	0,353	0,01	2,06E-47	MXD1	UPP	M2_vs_M0
5,87E-60	1,21836561	0,467	0,03	1,94E-55	RANBP2	UPP	M2_vs_M0
7,66E-67	1,21778829	0,655	0,09	2,54E-62	IGF1	UPP	M2_vs_M0
2,47E-43	1,21640953	0,292	0,01	8,18E-39	MMP19	UPP	M2_vs_M0
5,15E-61	1,20660966	0,79	0,18	1,70E-56	MARCKS	UPP	M2_vs_M0
5,21E-68	1,20625835	0,533	0,04	1,73E-63	MAT2A	UPP	M2_vs_M0
1,95E-56	1,19839983	0,695	0,14	6,44E-52	HSPA1A	UPP	M2_vs_M0
1,95E-64	1,19544617	0,57	0,06	6,47E-60	HIF1A	UPP	M2_vs_M0
7,96E-76	1,19163806	0,737	0,1	2,64E-71	CD4	UPP	M2_vs_M0
1,03E-54	1,19032203	0,523	0,07	3,40E-50	ID2	UPP	M2_vs_M0
3,09E-57	1,18201579	0,48	0,04	1,02E-52	BTG2	UPP	M2_vs_M0
1,09E-64	1,17567344	0,552	0,05	3,61E-60	PLTP	UPP	M2_vs_M0
2,62E-49	1,17432564	0,398	0,03	8,69E-45	CXCR4	UPP	M2_vs_M0
3,40E-57	1,17405932	0,361	0	1,13E-52	ABL2	UPP	M2_vs_M0
2,05E-56	1,17062857	0,679	0,14	6,80E-52	HNRNPU	UPP	M2_vs_M0
7,66E-74	1,15040277	0,581	0,05	2,54E-69	GATM	UPP	M2_vs_M0
1,20E-58	1,14633091	0,637	0,11	3,96E-54	FOLR2	UPP	M2_vs_M0
1,15E-68	1,14621853	0,626	0,08	3,80E-64	CMTM6	UPP	M2_vs_M0
2,01E-63	1,14615718	0,395	0	6,65E-59	PTGER4	UPP	M2_vs_M0
1,15E-75	1,14448233	0,631	0,06	3,80E-71	RTN4	UPP	M2_vs_M0
4,31E-70	1,14194971	0,501	0,03	1,43E-65	CD93	UPP	M2_vs_M0
1,50E-59	1,14113208	0,401	0,01	4,98E-55	RASGEF1B	UPP	M2_vs_M0
6,19E-42	1,14082895	0,711	0,22	2,05E-37	SOD2	UPP	M2_vs_M0
5,61E-65	1,13969679	0,684	0,11	1,86E-60	CSF1R	UPP	M2_vs_M0
5,54E-22	1,13760284	0,183	0,01	1,84E-17	CCL2	UPP	M2_vs_M0
1,60E-78	1,13304395	0,69	0,08	5,31E-74	RNF130	UPP	M2_vs_M0
7,60E-63	1,1323296	0,745	0,15	2,52E-58	TGFBI	UPP	M2_vs_M0
1,01E-60	1,12741479	0,432	0,02	3,33E-56	IER5	UPP	M2_vs_M0

9,86E-72	1,1232049	0,475	0,01	3,27E-67	ATP1B1	UPP	M2_vs_M0
8,76E-59	1,12055129	0,411	0,02	2,90E-54	CXCL12	UPP	M2_vs_M0
1,37E-71	1,11549116	0,573	0,05	4,53E-67	TACC1	UPP	M2_vs_M0
2,46E-45	1,11355238	0,313	0,01	8,16E-41	FILIP1L	UPP	M2_vs_M0
6,51E-46	1,11280492	0,313	0,01	2,16E-41	MAFF	UPP	M2_vs_M0
5,98E-43	1,11179518	0,408	0,05	1,98E-38	BIRC3	UPP	M2_vs_M0
1,94E-55	1,10524554	0,613	0,1	6,42E-51	PPP1CB	UPP	M2_vs_M0
2,26E-68	1,10152455	0,538	0,04	7,48E-64	GNA13	UPP	M2_vs_M0
1,37E-71	1,09800956	0,557	0,04	4,53E-67	ITPR2	UPP	M2_vs_M0
7,57E-68	1,09607336	0,485	0,02	2,51E-63	LPAR6	UPP	M2_vs_M0
3,08E-74	1,09587695	0,605	0,05	1,02E-69	AXL	UPP	M2_vs_M0
2,43E-74	1,09526751	0,531	0,03	8,06E-70	FRMD4B	UPP	M2_vs_M0
5,47E-70	1,09484493	0,483	0,02	1,81E-65	SIGLEC1	UPP	M2_vs_M0
2,47E-74	1,09405121	0,666	0,08	8,17E-70	QKI	UPP	M2_vs_M0
1,18E-64	1,09306836	0,525	0,04	3,90E-60	CEBPD	UPP	M2_vs_M0
4,15E-67	1,09013868	0,448	0,01	1,38E-62	KIF1B	UPP	M2_vs_M0
1,07E-75	1,07761808	0,682	0,08	3,55E-71	APLP2	UPP	M2_vs_M0
3,78E-49	1,07452019	0,955	0,43	1,25E-44	ITM2B	UPP	M2_vs_M0
4,50E-60	1,0713693	0,764	0,16	1,49E-55	RAC1	UPP	M2_vs_M0
6,86E-70	1,07129385	0,515	0,03	2,27E-65	IL10RA	UPP	M2_vs_M0
3,92E-47	1,06871735	0,804	0,24	1,30E-42	CD14	UPP	M2_vs_M0
1,74E-59	1,06715093	0,501	0,05	5,75E-55	DYNC1H1	UPP	M2_vs_M0
2,05E-52	1,06548226	0,836	0,24	6,79E-48	HLA-DMB	UPP	M2_vs_M0
2,48E-60	1,06122577	0,385	0,01	8,21E-56	LDLR	UPP	M2_vs_M0
2,91E-37	1,06014201	0,581	0,15	9,63E-33	HMOX1	UPP	M2_vs_M0
5,20E-61	1,05899757	0,703	0,12	1,72E-56	CREG1	UPP	M2_vs_M0
2,63E-55	1,05679745	0,377	0,01	8,71E-51	MYADM	UPP	M2_vs_M0
1,47E-58	1,05473875	0,374	0,01	4,88E-54	CSRNP1	UPP	M2_vs_M0
4,26E-44	1,05338861	0,851	0,29	1,41E-39	FUCA1	UPP	M2_vs_M0
6,04E-40	1,04912021	0,936	0,4	2,00E-35	CTSD	UPP	M2_vs_M0
2,00E-62	1,0486886	0,66	0,11	6,62E-58	ADA2	UPP	M2_vs_M0
1,51E-68	1,04662812	0,557	0,05	4,99E-64	ZFAND5	UPP	M2_vs_M0
2,17E-47	1,04520104	0,326	0,01	7,19E-43	ARL5B	UPP	M2_vs_M0
3,61E-70	1,04073402	0,525	0,03	1,20E-65	IQGAP2	UPP	M2_vs_M0
1,10E-62	1,0389806	0,454	0,02	3,63E-58	SOCS3	UPP	M2_vs_M0
3,50E-66	1,03894761	0,634	0,08	1,16E-61	CLTC	UPP	M2_vs_M0
4,28E-70	1,0380906	0,605	0,06	1,42E-65	MFSD1	UPP	M2_vs_M0
1,20E-63	1,02833773	0,605	0,08	3,96E-59	LAMP1	UPP	M2_vs_M0
1,96E-72	1,0264185	0,475	0,01	6,49E-68	AKAP9	UPP	M2_vs_M0
7,22E-70	1,02563166	0,597	0,06	2,39E-65	C6orf62	UPP	M2_vs_M0
1,54E-63	1,0234328	0,737	0,13	5,09E-59	RNASE6	UPP	M2_vs_M0
1,11E-67	1,0191135	0,507	0,03	3,66E-63	PDIA4	UPP	M2_vs_M0
1,21E-57	1,01589629	0,448	0,03	4,02E-53	SDC3	UPP	M2_vs_M0
6,59E-47	1,01359749	0,907	0,33	2,18E-42	MS4A6A	UPP	M2_vs_M0
4,21E-38	1,00887572	0,973	0,47	1,39E-33	CTSC	UPP	M2_vs_M0
5,94E-70	1,00345675	0,456	0,01	1,97E-65	MACF1	UPP	M2_vs_M0
6,71E-66	0,9960312	0,456	0,02	2,22E-61	SLC7A8	UPP	M2_vs_M0
4,98E-62	0,99269935	0,552	0,06	1,65E-57	GNS	UPP	M2_vs_M0
2,51E-58	0,98269991	0,995	0,86	8,32E-54	CD74	UPP	M2_vs_M0
1,79E-52	0,98241175	0,366	0,01	5,91E-48	KLF10	UPP	M2_vs_M0

2,40E-29	0,98021115	0,857	0,48	7,95E-25	H3F3B	UPP	M2_vs_M0
2,23E-41	0,97939131	0,907	0,35	7,39E-37	GRN	UPP	M2_vs_M0
6,97E-44	0,9760025	0,334	0,02	2,31E-39	MAN1A1	UPP	M2_vs_M0
8,80E-43	0,97476977	0,981	0,79	2,91E-38	MT-ND4	UPP	M2_vs_M0
1,52E-60	0,97367713	0,411	0,01	5,02E-56	CLN8	UPP	M2_vs_M0
5,24E-63	0,97123126	0,621	0,08	1,74E-58	CFLAR	UPP	M2_vs_M0
4,05E-51	0,97072155	0,358	0,01	1,34E-46	ARRDC3	UPP	M2_vs_M0
1,57E-62	0,97071133	0,491	0,04	5,19E-58	ATP2B1	UPP	M2_vs_M0
1,31E-45	0,96925925	0,416	0,05	4,34E-41	VMP1	UPP	M2_vs_M0
9,02E-58	0,96924217	0,424	0,02	2,99E-53	B4GALT1	UPP	M2_vs_M0
7,84E-55	0,96843138	0,379	0,01	2,60E-50	FOXO3	UPP	M2_vs_M0
1,07E-62	0,96838962	0,438	0,02	3,53E-58	MYO5A	UPP	M2_vs_M0
6,89E-65	0,96407332	0,493	0,03	2,28E-60	TRA2B	UPP	M2_vs_M0
3,04E-44	0,96306913	0,324	0,01	1,01E-39	DDX3Y	UPP	M2_vs_M0
4,55E-44	0,9626233	0,78	0,24	1,51E-39	DDX5	UPP	M2_vs_M0
2,78E-47	0,96077397	0,31	0,01	9,20E-43	GRASP	UPP	M2_vs_M0
6,74E-67	0,95812025	0,549	0,05	2,23E-62	RNF13	UPP	M2_vs_M0
2,24E-57	0,9537666	0,438	0,03	7,43E-53	SPRED1	UPP	M2_vs_M0
4,86E-65	0,95326954	0,615	0,08	1,61E-60	MGAT1	UPP	M2_vs_M0
1,06E-49	0,95308608	0,34	0,01	3,53E-45	ELL2	UPP	M2_vs_M0
1,18E-57	0,95116905	0,684	0,12	3,92E-53	FCGR2A	UPP	M2_vs_M0
1,23E-71	0,94893494	0,554	0,04	4,08E-67	RBM47	UPP	M2_vs_M0
2,14E-66	0,94835444	0,523	0,04	7,09E-62	SERINC1	UPP	M2_vs_M0
9,39E-49	0,9473562	0,401	0,03	3,11E-44	WDR45B	UPP	M2_vs_M0
1,10E-57	0,94495764	0,467	0,04	3,65E-53	DMXL2	UPP	M2_vs_M0
1,37E-65	0,94038046	0,528	0,04	4,55E-61	ANKRD12	UPP	M2_vs_M0
3,82E-45	0,93805171	0,923	0,37	1,27E-40	FCGRT	UPP	M2_vs_M0
1,16E-64	0,93750943	0,504	0,04	3,84E-60	C3AR1	UPP	M2_vs_M0
1,03E-68	0,93638635	0,605	0,06	3,41E-64	CD164	UPP	M2_vs_M0
9,27E-64	0,93425289	0,568	0,06	3,07E-59	WSB1	UPP	M2_vs_M0
8,21E-55	0,93314617	0,385	0,01	2,72E-50	MAP3K8	UPP	M2_vs_M0
1,19E-42	0,92915663	0,324	0,02	3,95E-38	HSPA1B	UPP	M2_vs_M0
4,45E-58	0,9264996	0,408	0,02	1,47E-53	ANKH	UPP	M2_vs_M0
4,11E-50	0,925886	0,347	0,01	1,36E-45	ETV3	UPP	M2_vs_M0
1,91E-41	0,91911847	0,297	0,01	6,33E-37	THBD	UPP	M2_vs_M0
3,96E-57	0,914103	0,398	0,01	1,31E-52	ITGAV	UPP	M2_vs_M0
1,09E-70	0,91383458	0,552	0,04	3,61E-66	UTRN	UPP	M2_vs_M0
8,77E-39	0,91252346	0,979	0,9	2,91E-34	MT-CO1	UPP	M2_vs_M0
1,08E-52	0,90827951	0,382	0,02	3,57E-48	PFKFB3	UPP	M2_vs_M0
8,22E-60	0,90725851	0,411	0,01	2,72E-55	ATP13A3	UPP	M2_vs_M0
5,26E-65	0,90713484	0,631	0,08	1,74E-60	DDX17	UPP	M2_vs_M0
7,41E-58	0,90543331	0,456	0,03	2,45E-53	JMJD1C	UPP	M2_vs_M0
1,25E-62	0,90443292	0,467	0,03	4,13E-58	NABP1	UPP	M2_vs_M0
2,99E-71	0,9032623	0,607	0,06	9,90E-67	ARL6IP1	UPP	M2_vs_M0
8,37E-51	0,9023072	0,377	0,02	2,77E-46	NRP2	UPP	M2_vs_M0
2,59E-44	0,8977497	0,679	0,16	8,58E-40	CTSL	UPP	M2_vs_M0
3,29E-43	0,89536578	0,995	0,69	1,09E-38	C1QA	UPP	M2_vs_M0
2,34E-58	0,89308928	0,395	0,01	7,75E-54	CLK1	UPP	M2_vs_M0
2,71E-30	0,89073454	0,942	0,69	8,97E-26	MT-ND2	UPP	M2_vs_M0
1,32E-55	0,88180141	0,369	0,01	4,36E-51	NFKBID	UPP	M2_vs_M0

6,78E-44	0,88039061	0,448	0,06	2,25E-39	YPEL5	UPP	M2_vs_M0
1,51E-41	0,87961496	0,34	0,03	5,00E-37	ITGAX	UPP	M2_vs_M0
6,04E-45	0,87869584	0,313	0,01	2,00E-40	PRDM1	UPP	M2_vs_M0
8,16E-59	0,87863104	0,491	0,04	2,70E-54	BRD2	UPP	M2_vs_M0
8,15E-58	0,87775786	0,401	0,01	2,70E-53	YTHDF3	UPP	M2_vs_M0
1,80E-55	0,87717149	0,416	0,02	5,97E-51	PLXDC2	UPP	M2_vs_M0
1,59E-65	0,87685817	0,48	0,03	5,28E-61	SGPL1	UPP	M2_vs_M0
3,07E-50	0,87606314	0,374	0,02	1,02E-45	ZC3HAV1	UPP	M2_vs_M0
9,35E-65	0,86900067	0,549	0,05	3,10E-60	ELF1	UPP	M2_vs_M0
1,11E-51	0,8684504	0,74	0,17	3,69E-47	CYBB	UPP	M2_vs_M0
2,90E-63	0,86557722	0,576	0,06	9,62E-59	TIMP2	UPP	M2_vs_M0
9,81E-48	0,86361893	0,371	0,02	3,25E-43	MAP2K3	UPP	M2_vs_M0
3,67E-30	0,86259954	0,963	0,76	1,22E-25	MT-ND1	UPP	M2_vs_M0
5,68E-45	0,8598374	0,302	0,01	1,88E-40	PELI1	UPP	M2_vs_M0
7,21E-68	0,85684915	0,531	0,04	2,39E-63	SH2B3	UPP	M2_vs_M0
7,42E-50	0,85665114	0,361	0,02	2,46E-45	PURB	UPP	M2_vs_M0
1,52E-39	0,85477174	0,565	0,13	5,02E-35	SELENOK	UPP	M2_vs_M0
1,24E-36	0,85381602	0,268	0,01	4,12E-32	KLF2	UPP	M2_vs_M0
2,77E-61	0,85228809	0,573	0,07	9,19E-57	ADAP2	UPP	M2_vs_M0
6,97E-63	0,84943002	0,435	0,02	2,31E-58	CELF1	UPP	M2_vs_M0
7,77E-60	0,84845987	0,499	0,04	2,57E-55	WWP1	UPP	M2_vs_M0
1,56E-50	0,84817292	0,512	0,07	5,17E-46	SCARB2	UPP	M2_vs_M0
5,00E-61	0,8454804	0,663	0,11	1,66E-56	M6PR	UPP	M2_vs_M0
8,55E-38	0,84509778	0,889	0,37	2,83E-33	LAPTM5	UPP	M2_vs_M0
1,75E-62	0,84391691	0,639	0,09	5,79E-58	RPN2	UPP	M2_vs_M0
8,46E-47	0,83463828	0,708	0,17	2,80E-42	PLD3	UPP	M2_vs_M0
2,72E-57	0,83355057	0,615	0,1	9,01E-53	DDX3X	UPP	M2_vs_M0
5,96E-60	0,83323967	0,562	0,07	1,97E-55	PMP22	UPP	M2_vs_M0
2,64E-40	0,83124185	0,801	0,26	8,74E-36	HSP90B1	UPP	M2_vs_M0
8,43E-53	0,83110284	0,422	0,03	2,79E-48	SKIL	UPP	M2_vs_M0
5,04E-26	0,82298358	0,17	0	1,67E-21	XIST	UPP	M2_vs_M0
3,58E-33	0,8209717	0,241	0,01	1,19E-28	RGCC	UPP	M2_vs_M0
1,28E-44	0,82021671	0,759	0,21	4,24E-40	GNAS	UPP	M2_vs_M0
6,33E-57	0,81709655	0,464	0,04	2,10E-52	ZFYVE16	UPP	M2_vs_M0
1,30E-56	0,81680433	0,623	0,1	4,32E-52	TMEM123	UPP	M2_vs_M0
2,95E-30	0,81411809	0,281	0,03	9,76E-26	ID3	UPP	M2_vs_M0
3,90E-31	0,81314447	0,223	0,01	1,29E-26	DUSP4	UPP	M2_vs_M0
1,66E-49	0,81181622	0,34	0,01	5,49E-45	BHLHE40	UPP	M2_vs_M0
1,92E-52	0,81113777	0,366	0,01	6,36E-48	CHD2	UPP	M2_vs_M0
4,95E-44	0,81071145	0,286	0	1,64E-39	NLRP3	UPP	M2_vs_M0
4,42E-55	0,8091244	0,419	0,03	1,46E-50	DSE	UPP	M2_vs_M0
1,41E-30	0,80659338	0,997	0,66	4,68E-26	C1QB	UPP	M2_vs_M0
2,51E-47	0,80644026	0,836	0,24	8,32E-43	ASAH1	UPP	M2_vs_M0
6,27E-53	0,80574628	0,363	0,01	2,08E-48	IL6R	UPP	M2_vs_M0
9,16E-52	0,80121577	0,594	0,1	3,04E-47	NFE2L2	UPP	M2_vs_M0
9,56E-61	0,79907701	0,544	0,06	3,17E-56	SLC15A3	UPP	M2_vs_M0
1,65E-56	0,79862939	0,493	0,05	5,45E-52	RGL1	UPP	M2_vs_M0
2,89E-52	0,79790439	0,422	0,03	9,56E-48	STX11	UPP	M2_vs_M0
2,96E-59	0,7971294	0,48	0,04	9,81E-55	ENTPD1	UPP	M2_vs_M0
6,69E-58	0,79703423	0,387	0,01	2,21E-53	ITGB5	UPP	M2_vs_M0

1,62E-61	0,7961295	0,613	0,08	5,37E-57	LACC1	UPP	M2_vs_M0
1,61E-56	0,78950237	0,706	0,13	5,34E-52	MS4A4A	UPP	M2_vs_M0
1,12E-53	0,78898057	0,602	0,1	3,72E-49	GM2A	UPP	M2_vs_M0
4,28E-59	0,78512209	0,544	0,06	1,42E-54	CTNNB1	UPP	M2_vs_M0
1,20E-41	0,78449269	0,83	0,26	3,96E-37	TMBIM6	UPP	M2_vs_M0
9,68E-54	0,78415258	0,39	0,02	3,21E-49	PLXNC1	UPP	M2_vs_M0
1,14E-46	0,78335668	0,337	0,01	3,79E-42	ARL4A	UPP	M2_vs_M0
1,10E-55	0,78089087	0,507	0,05	3,64E-51	VOPP1	UPP	M2_vs_M0
5,42E-64	0,77860428	0,562	0,06	1,80E-59	DDX24	UPP	M2_vs_M0
2,19E-52	0,77788674	0,446	0,04	7,24E-48	CD84	UPP	M2_vs_M0
1,52E-55	0,77700311	0,451	0,04	5,04E-51	ATP1A1	UPP	M2_vs_M0
1,98E-52	0,77689461	0,414	0,03	6,57E-48	STK17B	UPP	M2_vs_M0
6,55E-31	0,77342166	0,865	0,39	2,17E-26	PABPC1	UPP	M2_vs_M0
3,33E-54	0,7718085	0,491	0,05	1,10E-49	PABPC4	UPP	M2_vs_M0
5,95E-30	0,77170541	0,942	0,5	1,97E-25	CTSS	UPP	M2_vs_M0
6,72E-56	0,76621653	0,538	0,07	2,23E-51	TTYH3	UPP	M2_vs_M0
2,41E-43	0,76525233	0,308	0,01	7,98E-39	PAPSS2	UPP	M2_vs_M0
4,38E-56	0,76412664	0,499	0,05	1,45E-51	HEXB	UPP	M2_vs_M0
2,80E-48	0,76375518	0,541	0,09	9,29E-44	CD44	UPP	M2_vs_M0
6,53E-50	0,75969383	0,366	0,02	2,16E-45	CHD1	UPP	M2_vs_M0
4,87E-49	0,75782693	0,398	0,03	1,61E-44	SH3BP5	UPP	M2_vs_M0
6,63E-52	0,75706563	0,379	0,02	2,20E-47	PLEKHO2	UPP	M2_vs_M0
1,18E-53	0,75676755	0,43	0,03	3,90E-49	ST8SIA4	UPP	M2_vs_M0
1,29E-54	0,755347	0,363	0,01	4,26E-50	GPR155	UPP	M2_vs_M0
6,23E-56	0,75237735	0,52	0,06	2,06E-51	CD36	UPP	M2_vs_M0
4,95E-48	0,75144281	0,546	0,09	1,64E-43	EMP3	UPP	M2_vs_M0
9,49E-53	0,74880829	0,448	0,04	3,14E-48	ATRX	UPP	M2_vs_M0
7,31E-55	0,74078164	0,528	0,07	2,42E-50	HEXA	UPP	M2_vs_M0
8,72E-47	0,7389862	0,44	0,05	2,89E-42	CD59	UPP	M2_vs_M0
8,80E-45	0,73892762	0,374	0,03	2,92E-40	ERBIN	UPP	M2_vs_M0
1,85E-53	0,73666059	0,584	0,09	6,12E-49	DPP7	UPP	M2_vs_M0
1,12E-57	0,7356876	0,464	0,04	3,70E-53	RNF149	UPP	M2_vs_M0
6,36E-50	0,7352008	0,342	0,01	2,11E-45	EMB	UPP	M2_vs_M0
2,79E-52	0,73486898	0,714	0,15	9,26E-48	KCTD12	UPP	M2_vs_M0
2,32E-58	0,73446143	0,385	0,01	7,69E-54	AFF4	UPP	M2_vs_M0
1,33E-40	0,73429105	0,292	0,01	4,41E-36	SLC38A2	UPP	M2_vs_M0
3,67E-50	0,7342875	0,531	0,08	1,21E-45	HSPH1	UPP	M2_vs_M0
9,48E-23	0,73350029	0,175	0,01	3,14E-18	CXCL1	UPP	M2_vs_M0
1,79E-32	0,73296868	0,91	0,41	5,94E-28	HLA-E	UPP	M2_vs_M0
1,72E-37	0,73167726	0,316	0,03	5,68E-33	PLAU	UPP	M2_vs_M0
3,55E-54	0,72966888	0,536	0,07	1,18E-49	SFPQ	UPP	M2_vs_M0
9,29E-54	0,72849368	0,512	0,06	3,08E-49	LAIR1	UPP	M2_vs_M0
2,76E-46	0,72779	0,35	0,02	9,15E-42	C16orf72	UPP	M2_vs_M0
5,59E-57	0,72736221	0,488	0,04	1,85E-52	TTC3	UPP	M2_vs_M0
1,36E-40	0,72701647	0,302	0,01	4,51E-36	LHFPL2	UPP	M2_vs_M0
1,02E-50	0,72678789	0,382	0,02	3,37E-46	ADAM9	UPP	M2_vs_M0
1,35E-49	0,72672319	0,43	0,04	4,47E-45	ADGRE5	UPP	M2_vs_M0
2,29E-46	0,72469366	0,337	0,01	7,60E-42	GFRA2	UPP	M2_vs_M0
4,65E-48	0,72426853	0,379	0,03	1,54E-43	ZNF267	UPP	M2_vs_M0
8,35E-39	0,72267868	0,26	0,01	2,77E-34	PHLDA1	UPP	M2_vs_M0

7,60E-46	0,72230339	0,302	0,01	2,52E-41	ZMYM2	UPP	M2_vs_M0
2,71E-46	0,72200409	0,326	0,01	8,99E-42	TGIF1	UPP	M2_vs_M0
3,06E-55	0,72154825	0,517	0,06	1,01E-50	TRAM1	UPP	M2_vs_M0
5,49E-52	0,71997339	0,57	0,09	1,82E-47	SAMSN1	UPP	M2_vs_M0
6,37E-41	0,71815272	0,289	0,01	2,11E-36	TNFRSF21	UPP	M2_vs_M0
1,90E-50	0,71805263	0,334	0,01	6,29E-46	TGFBR1	UPP	M2_vs_M0
6,06E-48	0,71636781	0,361	0,02	2,01E-43	ATM	UPP	M2_vs_M0
2,76E-44	0,71492764	0,623	0,13	9,15E-40	ATP6AP2	UPP	M2_vs_M0
2,41E-42	0,71193962	0,451	0,07	7,97E-38	VSIG4	UPP	M2_vs_M0
8,20E-57	0,71128828	0,451	0,03	2,72E-52	PTTG1IP	UPP	M2_vs_M0
4,06E-56	0,7106441	0,562	0,07	1,34E-51	SYNGR2	UPP	M2_vs_M0
9,80E-55	0,70752493	0,395	0,02	3,25E-50	ZFHX3	UPP	M2_vs_M0
9,31E-31	0,70597347	0,782	0,29	3,08E-26	ACP5	UPP	M2_vs_M0
8,03E-52	0,70574297	0,398	0,02	2,66E-47	NUFIP2	UPP	M2_vs_M0
3,33E-42	0,7035207	0,496	0,08	1,10E-37	C2	UPP	M2_vs_M0
1,03E-51	0,70220014	0,515	0,07	3,42E-47	GPR34	UPP	M2_vs_M0
8,10E-49	0,70204124	0,531	0,08	2,68E-44	TMED10	UPP	M2_vs_M0
1,23E-47	0,70115954	0,496	0,07	4,08E-43	SSR1	UPP	M2_vs_M0
2,40E-46	0,70103617	0,305	0,01	7,94E-42	DST	UPP	M2_vs_M0
7,40E-58	0,70079826	0,629	0,1	2,45E-53	LAMP2	UPP	M2_vs_M0
1,20E-51	0,70000653	0,387	0,02	3,99E-47	NEU1	UPP	M2_vs_M0
4,21E-58	0,69765063	0,454	0,03	1,39E-53	SASH1	UPP	M2_vs_M0
1,49E-51	0,69705284	0,605	0,11	4,93E-47	AP1B1	UPP	M2_vs_M0
1,83E-45	0,69641992	0,395	0,04	6,05E-41	SLC43A2	UPP	M2_vs_M0
5,12E-44	0,69621333	0,297	0,01	1,70E-39	IFRD1	UPP	M2_vs_M0
3,05E-24	0,69069989	0,958	0,58	1,01E-19	C1QC	UPP	M2_vs_M0
4,22E-47	0,68835062	0,446	0,05	1,40E-42	BLOC1S6	UPP	M2_vs_M0
9,87E-51	0,68826711	0,44	0,04	3,27E-46	IDS	UPP	M2_vs_M0
1,50E-56	0,6880852	0,52	0,06	4,96E-52	HNRNPH1	UPP	M2_vs_M0
1,11E-35	0,68608549	0,231	0	3,68E-31	SLC25A44	UPP	M2_vs_M0
5,87E-45	0,6853839	0,308	0,01	1,94E-40	FAM53C	UPP	M2_vs_M0
6,32E-28	0,68480224	0,3	0,04	2,09E-23	PDK4	UPP	M2_vs_M0
1,45E-39	0,68336482	0,305	0,02	4,80E-35	SEMA4A	UPP	M2_vs_M0
3,69E-42	0,68237099	0,326	0,02	1,22E-37	SEMA6B	UPP	M2_vs_M0
4,69E-52	0,68235872	0,427	0,03	1,55E-47	SIRPA	UPP	M2_vs_M0
2,35E-48	0,67921298	0,398	0,03	7,79E-44	PRCP	UPP	M2_vs_M0
1,25E-46	0,67907464	0,353	0,02	4,14E-42	IL6ST	UPP	M2_vs_M0
2,57E-38	0,67715519	0,268	0,01	8,50E-34	B3GNT5	UPP	M2_vs_M0
9,62E-51	0,6769012	0,549	0,08	3,19E-46	FUS	UPP	M2_vs_M0
1,05E-56	0,67616213	0,557	0,07	3,46E-52	EIF5	UPP	M2_vs_M0
6,72E-33	0,67477963	0,504	0,12	2,22E-28	FCGR3A	UPP	M2_vs_M0
3,23E-47	0,67419442	0,321	0,01	1,07E-42	ZSWIM6	UPP	M2_vs_M0
3,08E-47	0,67180963	0,316	0,01	1,02E-42	TET2	UPP	M2_vs_M0
1,24E-56	0,67166614	0,401	0,02	4,11E-52	GALNT1	UPP	M2_vs_M0
3,81E-54	0,67048358	0,416	0,03	1,26E-49	TNFRSF1A	UPP	M2_vs_M0
9,32E-41	0,66977657	0,302	0,01	3,09E-36	EDEM1	UPP	M2_vs_M0
2,94E-51	0,66884818	0,446	0,04	9,72E-47	MGAT4A	UPP	M2_vs_M0
5,88E-45	0,66867941	0,66	0,15	1,95E-40	CANX	UPP	M2_vs_M0
6,49E-53	0,6674287	0,621	0,11	2,15E-48	MBNL1	UPP	M2_vs_M0
1,98E-55	0,66695492	0,589	0,09	6,55E-51	LAPTM4A	UPP	M2_vs_M0

3,02E-50	0,66527093	0,435	0,04	1,00E-45	COLGALT1	UPP	M2_vs_M0
3,41E-32	0,66525249	0,225	0,01	1,13E-27	THAP2	UPP	M2_vs_M0
4,80E-55	0,66511162	0,382	0,01	1,59E-50	SERINC3	UPP	M2_vs_M0
8,27E-49	0,66437431	0,438	0,04	2,74E-44	RASSF2	UPP	M2_vs_M0
3,02E-26	0,66434065	0,955	0,72	1,00E-21	SAT1	UPP	M2_vs_M0
3,47E-25	0,66391679	0,976	0,65	1,15E-20	HLA-B	UPP	M2_vs_M0
6,83E-51	0,66387806	0,674	0,14	2,26E-46	SARAF	UPP	M2_vs_M0
3,56E-46	0,663135	0,35	0,02	1,18E-41	PTPRJ	UPP	M2_vs_M0
4,60E-52	0,66244597	0,549	0,08	1,52E-47	HLA-DOA	UPP	M2_vs_M0
1,20E-53	0,66180797	0,554	0,08	3,96E-49	PRRC2C	UPP	M2_vs_M0
2,77E-43	0,66136919	0,308	0,01	9,19E-39	USP36	UPP	M2_vs_M0
2,24E-56	0,66125888	0,552	0,07	7,41E-52	PTPRE	UPP	M2_vs_M0
9,76E-50	0,65969021	0,607	0,11	3,23E-45	TAPBP	UPP	M2_vs_M0
1,13E-54	0,65917307	0,485	0,05	3,76E-50	WNK1	UPP	M2_vs_M0
1,08E-42	0,65892286	0,385	0,04	3,58E-38	APOL6	UPP	M2_vs_M0
2,45E-39	0,65870624	0,273	0,01	8,13E-35	PRMT9	UPP	M2_vs_M0
1,87E-46	0,65844882	0,477	0,07	6,19E-42	ETF1	UPP	M2_vs_M0
7,25E-43	0,65723323	0,34	0,02	2,40E-38	SCD	UPP	M2_vs_M0
5,09E-42	0,65705509	0,52	0,09	1,68E-37	SCPEP1	UPP	M2_vs_M0
5,50E-54	0,65642468	0,507	0,06	1,82E-49	MTDH	UPP	M2_vs_M0
1,04E-39	0,65573085	0,297	0,01	3,44E-35	MALT1	UPP	M2_vs_M0
2,16E-47	0,651983	0,568	0,1	7,16E-43	RAB31	UPP	M2_vs_M0
7,36E-51	0,65096973	0,371	0,02	2,44E-46	IFI30	UPP	M2_vs_M0
7,72E-45	0,65013864	0,687	0,16	2,56E-40	SQSTM1	UPP	M2_vs_M0
7,08E-24	0,6494631	0,151	0	2,35E-19	IL10	UPP	M2_vs_M0
1,03E-38	0,64866781	0,255	0	3,41E-34	VPS37B	UPP	M2_vs_M0
2,58E-46	0,64768602	0,379	0,03	8,56E-42	LILRB2	UPP	M2_vs_M0
1,18E-53	0,64748796	0,523	0,07	3,92E-49	NAIP	UPP	M2_vs_M0
1,35E-40	0,64646096	0,321	0,02	4,47E-36	PPP1R10	UPP	M2_vs_M0
1,74E-45	0,64589772	0,345	0,02	5,76E-41	POLR2A	UPP	M2_vs_M0
1,23E-35	0,64571205	0,236	0	4,08E-31	AC020916.	UPP	M2_vs_M0
1,16E-38	0,64519374	0,265	0,01	3,84E-34	MDM4	UPP	M2_vs_M0
3,81E-45	0,64440272	0,671	0,15	1,26E-40	PTPRC	UPP	M2_vs_M0
2,09E-51	0,64406437	0,475	0,05	6,92E-47	TBXAS1	UPP	M2_vs_M0
1,54E-50	0,64399677	0,528	0,07	5,10E-46	TGOLN2	UPP	M2_vs_M0
4,07E-22	0,64393596	0,987	0,6	1,35E-17	HLA-A	UPP	M2_vs_M0
1,50E-49	0,64304837	0,416	0,04	4,96E-45	ATP2A2	UPP	M2_vs_M0
3,37E-43	0,64266563	0,276	0	1,12E-38	ARIH1	UPP	M2_vs_M0
4,29E-43	0,64207657	0,302	0,01	1,42E-38	LPCAT1	UPP	M2_vs_M0
2,02E-51	0,64148571	0,501	0,06	6,68E-47	GRINA	UPP	M2_vs_M0
1,93E-49	0,64131442	0,411	0,03	6,40E-45	STOM	UPP	M2_vs_M0
1,81E-36	0,64079999	0,252	0,01	6,01E-32	ZC3H12A	UPP	M2_vs_M0
2,99E-53	0,64071658	0,469	0,05	9,91E-49	CD99	UPP	M2_vs_M0
7,31E-39	0,63860185	0,286	0,01	2,42E-34	BCL3	UPP	M2_vs_M0
4,42E-36	0,63838756	0,244	0,01	1,46E-31	TPRA1	UPP	M2_vs_M0
1,21E-40	0,63835061	0,286	0,01	4,01E-36	IL4R	UPP	M2_vs_M0
6,89E-41	0,63671256	0,279	0,01	2,28E-36	SERINC5	UPP	M2_vs_M0
6,89E-34	0,63574247	0,326	0,04	2,28E-29	TCN2	UPP	M2_vs_M0
9,18E-39	0,63497542	0,286	0,01	3,04E-34	DUSP3	UPP	M2_vs_M0
4,05E-44	0,63400886	0,342	0,02	1,34E-39	KCTD20	UPP	M2_vs_M0

2,45E-50	0,63342642	0,44	0,04	8,11E-46	RHOQ	UPP	M2_vs_M0
2,10E-47	0,63323392	0,43	0,04	6,96E-43	ARL8B	UPP	M2_vs_M0
9,27E-45	0,63303735	0,523	0,09	3,07E-40	AOAH	UPP	M2_vs_M0
7,63E-48	0,63199833	0,403	0,03	2,53E-43	GOLGA4	UPP	M2_vs_M0
1,79E-55	0,63169807	0,618	0,1	5,93E-51	BCAP31	UPP	M2_vs_M0
5,06E-35	0,62864096	0,263	0,01	1,68E-30	IRS2	UPP	M2_vs_M0
4,27E-47	0,62666567	0,369	0,02	1,42E-42	SLC4A7	UPP	M2_vs_M0
2,41E-50	0,62628547	0,459	0,05	7,98E-46	MYO9B	UPP	M2_vs_M0
7,71E-48	0,62435367	0,531	0,08	2,56E-43	AKAP13	UPP	M2_vs_M0
2,89E-44	0,62384793	0,371	0,03	9,58E-40	ABI1	UPP	M2_vs_M0
1,96E-50	0,62320634	0,456	0,05	6,51E-46	AMD1	UPP	M2_vs_M0
1,91E-40	0,62239426	0,464	0,08	6,33E-36	RILPL2	UPP	M2_vs_M0
5,90E-50	0,62173499	0,393	0,03	1,95E-45	PNISR	UPP	M2_vs_M0
5,51E-49	0,62162736	0,342	0,01	1,83E-44	FNIP2	UPP	M2_vs_M0
3,87E-47	0,62137692	0,411	0,04	1,28E-42	CHD4	UPP	M2_vs_M0
3,57E-35	0,62122228	0,249	0,01	1,18E-30	GZF1	UPP	M2_vs_M0
6,14E-56	0,62086533	0,446	0,03	2,03E-51	RPN1	UPP	M2_vs_M0
4,44E-33	0,62067666	0,329	0,04	1,47E-28	GLA	UPP	M2_vs_M0
8,03E-45	0,61988823	0,387	0,04	2,66E-40	RSRP1	UPP	M2_vs_M0
2,35E-41	0,61921747	0,416	0,05	7,79E-37	GAA	UPP	M2_vs_M0
5,08E-41	0,6191512	0,305	0,01	1,68E-36	TNS3	UPP	M2_vs_M0
1,19E-42	0,61765849	0,289	0,01	3,95E-38	SGMS1	UPP	M2_vs_M0
3,53E-35	0,6174506	0,244	0,01	1,17E-30	TSC22D2	UPP	M2_vs_M0
4,41E-30	0,61717432	0,212	0,01	1,46E-25	OLR1	UPP	M2_vs_M0
2,12E-53	0,61421425	0,422	0,03	7,03E-49	SNX9	UPP	M2_vs_M0
1,90E-43	0,61421154	0,451	0,06	6,31E-39	VSIR	UPP	M2_vs_M0
1,62E-23	0,61355462	0,984	0,87	5,36E-19	MT-ND3	UPP	M2_vs_M0
1,53E-36	0,61025355	0,456	0,08	5,06E-32	SERPINF1	UPP	M2_vs_M0
6,39E-45	0,61016608	0,318	0,01	2,12E-40	BTBD7	UPP	M2_vs_M0
6,67E-45	0,60922558	0,326	0,01	2,21E-40	BAIAP2	UPP	M2_vs_M0
9,67E-49	0,60691375	0,411	0,04	3,20E-44	VPS13C	UPP	M2_vs_M0
8,00E-27	0,60685306	0,191	0,01	2,65E-22	PMAIP1	UPP	M2_vs_M0
9,95E-43	0,60500895	0,393	0,04	3,29E-38	CRTAP	UPP	M2_vs_M0
5,12E-40	0,60418098	0,289	0,01	1,70E-35	EPHX1	UPP	M2_vs_M0
7,74E-42	0,60401683	0,305	0,01	2,57E-37	PLXND1	UPP	M2_vs_M0
9,92E-43	0,60354835	0,639	0,15	3,29E-38	CD53	UPP	M2_vs_M0
2,34E-39	0,60313805	0,432	0,07	7,75E-35	LGALS3BP	UPP	M2_vs_M0
5,62E-41	0,60125249	0,329	0,02	1,86E-36	FAM198B	UPP	M2_vs_M0
4,48E-41	0,60115694	0,305	0,01	1,48E-36	EIF3B	UPP	M2_vs_M0
1,73E-36	0,59731624	0,257	0,01	5,73E-32	WLS	UPP	M2_vs_M0
2,17E-40	0,59678923	0,31	0,02	7,20E-36	NUP58	UPP	M2_vs_M0
1,33E-23	0,59674822	0,875	0,39	4,40E-19	HLA-DMA	UPP	M2_vs_M0
5,78E-44	0,59671482	0,342	0,02	1,91E-39	P4HA1	UPP	M2_vs_M0
2,90E-49	0,5957987	0,374	0,02	9,60E-45	ADAM28	UPP	M2_vs_M0
1,38E-47	0,59454873	0,408	0,04	4,57E-43	YME1L1	UPP	M2_vs_M0
4,37E-40	0,59365092	0,321	0,02	1,45E-35	GABARAPL	UPP	M2_vs_M0
5,00E-50	0,59200601	0,48	0,06	1,66E-45	PPIG	UPP	M2_vs_M0
5,79E-48	0,59199481	0,385	0,03	1,92E-43	MMP14	UPP	M2_vs_M0
9,77E-37	0,59136633	0,366	0,05	3,24E-32	BCAS2	UPP	M2_vs_M0
1,12E-46	0,59055424	0,329	0,01	3,70E-42	PER1	UPP	M2_vs_M0

7,14E-43	0,58934462	0,321	0,02	2,36E-38	SEC63	UPP	M2_vs_M0
4,12E-47	0,58917055	0,366	0,02	1,36E-42	TM9SF3	UPP	M2_vs_M0
9,90E-35	0,58862202	0,236	0,01	3,28E-30	DDIT4	UPP	M2_vs_M0
4,59E-42	0,58755193	0,321	0,02	1,52E-37	TMEM248	UPP	M2_vs_M0
1,64E-46	0,5851461	0,39	0,03	5,44E-42	NFIC	UPP	M2_vs_M0
1,05E-44	0,58488598	0,634	0,14	3,47E-40	ARL6IP5	UPP	M2_vs_M0
1,54E-39	0,58456786	0,31	0,02	5,11E-35	EPS15	UPP	M2_vs_M0
8,07E-55	0,58443531	0,435	0,03	2,67E-50	EMILIN2	UPP	M2_vs_M0
8,33E-29	0,58380013	0,194	0	2,76E-24	RHOU	UPP	M2_vs_M0
3,12E-53	0,58315381	0,371	0,01	1,03E-48	GOLGB1	UPP	M2_vs_M0
3,01E-47	0,58214246	0,347	0,02	9,96E-43	CREBRF	UPP	M2_vs_M0
4,49E-42	0,58165153	0,398	0,04	1,49E-37	CMKLR1	UPP	M2_vs_M0
6,22E-28	0,58157578	0,886	0,37	2,06E-23	CD68	UPP	M2_vs_M0
3,79E-40	0,5811477	0,289	0,01	1,26E-35	YIPF4	UPP	M2_vs_M0
1,72E-41	0,58097058	0,313	0,02	5,71E-37	GLMP	UPP	M2_vs_M0
2,90E-47	0,579355	0,342	0,01	9,59E-43	ATP6V0A1	UPP	M2_vs_M0
3,86E-36	0,57762337	0,302	0,02	1,28E-31	PPP4R3A	UPP	M2_vs_M0
4,08E-38	0,57542652	0,31	0,02	1,35E-33	MKNK2	UPP	M2_vs_M0
2,15E-41	0,57536932	0,316	0,02	7,12E-37	KCNK6	UPP	M2_vs_M0
7,39E-38	0,57478496	0,244	0	2,45E-33	MIR22HG	UPP	M2_vs_M0
3,13E-33	0,57466887	0,78	0,25	1,04E-28	RNASET2	UPP	M2_vs_M0
1,70E-37	0,57464962	0,406	0,06	5,64E-33	SERPINB9	UPP	M2_vs_M0
3,67E-32	0,57343838	0,764	0,25	1,21E-27	CALR	UPP	M2_vs_M0
3,37E-39	0,57291606	0,321	0,02	1,12E-34	PIM3	UPP	M2_vs_M0
1,54E-47	0,5708787	0,382	0,03	5,09E-43	MYCBP2	UPP	M2_vs_M0
7,64E-52	0,57031341	0,485	0,05	2,53E-47	OS9	UPP	M2_vs_M0
1,78E-43	0,57004087	0,393	0,04	5,89E-39	MARCH1	UPP	M2_vs_M0
6,96E-37	0,56945651	0,321	0,03	2,31E-32	EFR3A	UPP	M2_vs_M0
1,58E-46	0,56887202	0,43	0,05	5,22E-42	SCAMP2	UPP	M2_vs_M0
1,56E-44	0,56806869	0,363	0,03	5,15E-40	AHCYL1	UPP	M2_vs_M0
1,64E-33	0,56788727	0,218	0	5,42E-29	JMY	UPP	M2_vs_M0
1,09E-36	0,56755092	0,273	0,01	3,61E-32	IGF2R	UPP	M2_vs_M0
5,05E-39	0,56741103	0,302	0,02	1,67E-34	RYBP	UPP	M2_vs_M0
2,84E-47	0,56676412	0,361	0,02	9,42E-43	CIITA	UPP	M2_vs_M0
1,38E-47	0,56659894	0,393	0,03	4,57E-43	KPNA4	UPP	M2_vs_M0
3,90E-49	0,56578262	0,353	0,01	1,29E-44	SEL1L	UPP	M2_vs_M0
2,16E-41	0,56557602	0,332	0,02	7,16E-37	MAGT1	UPP	M2_vs_M0
3,64E-26	0,56553047	0,207	0,01	1,21E-21	ABHD5	UPP	M2_vs_M0
1,61E-39	0,56480147	0,271	0,01	5,32E-35	TOB2	UPP	M2_vs_M0
9,30E-32	0,56462	0,228	0,01	3,08E-27	IGFBP4	UPP	M2_vs_M0
1,15E-38	0,56437973	0,265	0,01	3,80E-34	MAN2A1	UPP	M2_vs_M0
1,89E-47	0,56335094	0,401	0,03	6,28E-43	GNAQ	UPP	M2_vs_M0
5,31E-40	0,56150072	0,279	0,01	1,76E-35	ZBTB1	UPP	M2_vs_M0
4,15E-49	0,5608292	0,584	0,1	1,38E-44	RBM39	UPP	M2_vs_M0
1,48E-48	0,56006049	0,401	0,03	4,89E-44	SPAG9	UPP	M2_vs_M0
2,37E-42	0,5590395	0,35	0,03	7,85E-38	LEPROT	UPP	M2_vs_M0
2,37E-23	0,5589744	0,926	0,48	7,84E-19	FCER1G	UPP	M2_vs_M0
2,40E-38	0,5588129	0,308	0,02	7,94E-34	HERC1	UPP	M2_vs_M0
1,88E-46	0,55555605	0,379	0,03	6,24E-42	NISCH	UPP	M2_vs_M0
2,97E-32	0,55519516	0,382	0,07	9,82E-28	ENPP2	UPP	M2_vs_M0

6,04E-33	0,55516038	0,469	0,1	2,00E-28	SERPING1	UPP	M2_vs_M0
3,06E-38	0,55484526	0,302	0,02	1,01E-33	PPP2CB	UPP	M2_vs_M0
5,83E-46	0,55467013	0,363	0,02	1,93E-41	SFT2D2	UPP	M2_vs_M0
7,71E-47	0,55462546	0,477	0,06	2,56E-42	LCP2	UPP	M2_vs_M0
1,77E-41	0,55403157	0,419	0,05	5,87E-37	RAB20	UPP	M2_vs_M0
1,69E-42	0,55401144	0,342	0,02	5,60E-38	HSD17B12	UPP	M2_vs_M0
1,59E-46	0,55378002	0,44	0,05	5,27E-42	HNRNPUL1	UPP	M2_vs_M0
2,23E-37	0,55237618	0,263	0,01	7,40E-33	ST3GAL1	UPP	M2_vs_M0
9,16E-45	0,55224003	0,337	0,02	3,04E-40	CEP350	UPP	M2_vs_M0
5,33E-50	0,55204506	0,406	0,03	1,76E-45	PECAM1	UPP	M2_vs_M0
1,65E-33	0,55198682	0,361	0,05	5,46E-29	DNAJB1	UPP	M2_vs_M0
4,52E-32	0,55071214	0,273	0,02	1,50E-27	CITED2	UPP	M2_vs_M0
1,31E-44	0,54999255	0,321	0,01	4,34E-40	HELZ	UPP	M2_vs_M0
5,18E-45	0,54896777	0,454	0,06	1,71E-40	MLEC	UPP	M2_vs_M0
6,82E-48	0,54787466	0,443	0,05	2,26E-43	MSR1	UPP	M2_vs_M0
5,39E-49	0,54742394	0,52	0,07	1,79E-44	ITGB1	UPP	M2_vs_M0
4,25E-39	0,54730788	0,639	0,16	1,41E-34	YWHAZ	UPP	M2_vs_M0
7,43E-42	0,54708576	0,353	0,03	2,46E-37	TMEM30A	UPP	M2_vs_M0
3,01E-38	0,54644234	0,302	0,02	9,99E-34	KHSRP	UPP	M2_vs_M0
1,66E-45	0,54501725	0,385	0,03	5,49E-41	GNPTAB	UPP	M2_vs_M0
1,03E-39	0,54394952	0,353	0,03	3,40E-35	UBE2Z	UPP	M2_vs_M0
6,90E-52	0,54370663	0,459	0,04	2,29E-47	NDFIP1	UPP	M2_vs_M0
5,71E-36	0,54324701	0,297	0,02	1,89E-31	TNPO1	UPP	M2_vs_M0
3,94E-44	0,54238274	0,493	0,08	1,31E-39	THEMIS2	UPP	M2_vs_M0
2,54E-41	0,5416423	0,292	0,01	8,40E-37	LRRC8C	UPP	M2_vs_M0
7,64E-44	0,54130579	0,374	0,03	2,53E-39	TLR4	UPP	M2_vs_M0
1,25E-46	0,54124421	0,427	0,04	4,14E-42	RTN3	UPP	M2_vs_M0
4,07E-24	0,5403549	0,719	0,28	1,35E-19	ANXA1	UPP	M2_vs_M0
1,96E-35	0,54027005	0,26	0,01	6,49E-31	GPCPD1	UPP	M2_vs_M0
2,69E-36	0,53952657	0,313	0,03	8,93E-32	FAM91A1	UPP	M2_vs_M0
8,54E-43	0,53890996	0,345	0,02	2,83E-38	MANBA	UPP	M2_vs_M0
6,60E-39	0,538573	0,355	0,04	2,19E-34	TECR	UPP	M2_vs_M0
6,82E-43	0,53818574	0,363	0,03	2,26E-38	ADIPOR1	UPP	M2_vs_M0
3,32E-42	0,53813234	0,292	0,01	1,10E-37	ARID1B	UPP	M2_vs_M0
4,01E-24	0,53811254	0,891	0,5	1,33E-19	MT-ND5	UPP	M2_vs_M0
1,83E-51	0,53778149	0,477	0,05	6,05E-47	TCF4	UPP	M2_vs_M0
6,78E-36	0,53774219	0,377	0,05	2,25E-31	PPIF	UPP	M2_vs_M0
2,92E-47	0,53732644	0,422	0,04	9,67E-43	KHDRBS1	UPP	M2_vs_M0
2,32E-42	0,53708162	0,35	0,03	7,70E-38	EFCAB14	UPP	M2_vs_M0
5,23E-41	0,53647797	0,48	0,08	1,73E-36	TNFAIP2	UPP	M2_vs_M0
2,00E-43	0,53530733	0,358	0,03	6,62E-39	PLEKHA2	UPP	M2_vs_M0
2,42E-38	0,53474568	0,279	0,01	8,01E-34	FBXO11	UPP	M2_vs_M0
4,96E-41	0,53458565	0,3	0,01	1,64E-36	SMG1	UPP	M2_vs_M0
1,95E-35	0,53454309	0,271	0,01	6,45E-31	NIN	UPP	M2_vs_M0
2,39E-36	0,53401293	0,281	0,02	7,93E-32	CMTM3	UPP	M2_vs_M0
1,35E-42	0,53354563	0,334	0,02	4,48E-38	BACH1	UPP	M2_vs_M0
8,99E-36	0,53291201	0,292	0,02	2,98E-31	CPM	UPP	M2_vs_M0
4,11E-25	0,53212689	0,21	0,02	1,36E-20	MTRNR2L1	UPP	M2_vs_M0
1,38E-30	0,52984925	0,379	0,07	4,58E-26	SLAMF7	UPP	M2_vs_M0
2,11E-47	0,52944326	0,435	0,05	7,00E-43	STAT3	UPP	M2_vs_M0

1,17E-32	0,52898637	0,233	0,01	3,87E-28	RIN2	UPP	M2_vs_M0
1,24E-42	0,526208	0,347	0,03	4,12E-38	PNPLA8	UPP	M2_vs_M0
2,78E-47	0,52596612	0,414	0,04	9,20E-43	RAB21	UPP	M2_vs_M0
6,43E-29	0,52391639	0,215	0,01	2,13E-24	CPED1	UPP	M2_vs_M0
3,55E-44	0,52315489	0,334	0,02	1,18E-39	GLS	UPP	M2_vs_M0
1,56E-30	0,52265942	0,215	0,01	5,18E-26	SESN2	UPP	M2_vs_M0
2,94E-37	0,52264893	0,292	0,02	9,73E-33	METRNL	UPP	M2_vs_M0
9,67E-39	0,52232484	0,286	0,01	3,20E-34	FOXP1	UPP	M2_vs_M0
1,61E-29	0,52211914	0,268	0,03	5,33E-25	UNC93B1	UPP	M2_vs_M0
8,95E-32	0,52142342	0,223	0,01	2,97E-27	RRP12	UPP	M2_vs_M0
1,04E-49	0,52126514	0,424	0,04	3,46E-45	MAPKAPK2	UPP	M2_vs_M0
1,04E-32	0,52041028	0,302	0,03	3,45E-28	PTPN1	UPP	M2_vs_M0
1,94E-44	0,51976227	0,345	0,02	6,43E-40	PUM1	UPP	M2_vs_M0
1,17E-48	0,51915771	0,411	0,04	3,89E-44	MAP3K2	UPP	M2_vs_M0
5,83E-48	0,51879414	0,493	0,07	1,93E-43	ZNF106	UPP	M2_vs_M0
3,80E-16	0,51806145	0,117	0,01	1,26E-11	AREG	UPP	M2_vs_M0
1,48E-40	0,51751673	0,297	0,01	4,90E-36	EP300	UPP	M2_vs_M0
8,03E-47	0,51684647	0,411	0,04	2,66E-42	XBP1	UPP	M2_vs_M0
4,84E-43	0,51593798	0,369	0,03	1,60E-38	NR3C1	UPP	M2_vs_M0
9,20E-17	0,51565542	0,928	0,55	3,05E-12	NPC2	UPP	M2_vs_M0
6,58E-46	0,51501321	0,34	0,02	2,18E-41	RBM5	UPP	M2_vs_M0
9,82E-32	0,51472627	0,284	0,03	3,25E-27	GOLIM4	UPP	M2_vs_M0
1,32E-39	0,51423081	0,286	0,01	4,37E-35	RALGDS	UPP	M2_vs_M0
7,83E-38	0,5141446	0,271	0,01	2,59E-33	SAMD4B	UPP	M2_vs_M0
1,90E-31	0,5141109	0,225	0,01	6,31E-27	DENND4A	UPP	M2_vs_M0
7,06E-39	0,51405495	0,334	0,03	2,34E-34	SOAT1	UPP	M2_vs_M0
6,21E-40	0,51396565	0,565	0,12	2,06E-35	ARHGDIA	UPP	M2_vs_M0
1,83E-46	0,51316365	0,416	0,04	6,05E-42	CELF2	UPP	M2_vs_M0
1,38E-38	0,51311727	0,3	0,02	4,58E-34	TBL1XR1	UPP	M2_vs_M0
4,32E-42	0,5130747	0,39	0,04	1,43E-37	IL13RA1	UPP	M2_vs_M0
1,31E-31	0,51267243	0,26	0,02	4,34E-27	SLC20A1	UPP	M2_vs_M0
1,32E-39	0,51255412	0,427	0,06	4,39E-35	EPB41L3	UPP	M2_vs_M0
4,32E-40	0,51188024	0,342	0,03	1,43E-35	PDCD6IP	UPP	M2_vs_M0
2,34E-43	0,51174237	0,573	0,11	7,76E-39	SEC62	UPP	M2_vs_M0
2,47E-38	0,51137263	0,321	0,03	8,17E-34	XIAP	UPP	M2_vs_M0
2,12E-40	0,50965023	0,34	0,03	7,03E-36	PAFAH1B2	UPP	M2_vs_M0
9,12E-48	0,5096035	0,398	0,03	3,02E-43	PHF20	UPP	M2_vs_M0
4,15E-36	0,50891305	0,233	0	1,37E-31	TENT4B	UPP	M2_vs_M0
3,80E-49	0,50869531	0,438	0,04	1,26E-44	BCLAF1	UPP	M2_vs_M0
7,77E-39	0,50839072	0,292	0,01	2,57E-34	PDE4A	UPP	M2_vs_M0
2,31E-29	0,50837166	0,212	0,01	7,64E-25	TRIB1	UPP	M2_vs_M0
1,25E-43	0,50820764	0,321	0,01	4,15E-39	ATF6	UPP	M2_vs_M0
3,17E-27	0,50818524	0,204	0,01	1,05E-22	FADS1	UPP	M2_vs_M0
4,42E-45	0,50764625	0,363	0,03	1,47E-40	PCM1	UPP	M2_vs_M0
6,65E-32	0,50730625	0,244	0,01	2,20E-27	TLNRD1	UPP	M2_vs_M0
6,88E-34	0,50497659	0,236	0,01	2,28E-29	ABCG1	UPP	M2_vs_M0
2,01E-44	0,50413859	0,305	0,01	6,67E-40	MERTK	UPP	M2_vs_M0
1,74E-38	0,50401691	0,308	0,02	5,76E-34	SEC61A1	UPP	M2_vs_M0
3,53E-43	0,50338264	0,366	0,03	1,17E-38	USP9X	UPP	M2_vs_M0
7,28E-42	0,50330441	0,533	0,1	2,41E-37	SRSF5	UPP	M2_vs_M0

1,86E-41	0,5022025	0,39	0,04	6,17E-37	WTAP	UPP	M2_vs_M0
3,39E-36	0,50168333	0,271	0,01	1,12E-31	RNF144B	UPP	M2_vs_M0
6,22E-34	0,50109666	0,231	0,01	2,06E-29	AC016831.	UPP	M2_vs_M0
2,13E-49	0,50106318	0,43	0,04	7,04E-45	PARP14	UPP	M2_vs_M0
2,80E-44	0,50054761	0,504	0,08	9,27E-40	LYN	UPP	M2_vs_M0
1,51E-23	0,50041268	0,212	0,02	5,00E-19	ITGB7	UPP	M2_vs_M0
3,11E-39	0,49996446	0,366	0,04	1,03E-34	CD46	UPP	M2_vs_M0
5,51E-39	0,49926776	0,302	0,02	1,83E-34	KIDINS220	UPP	M2_vs_M0
2,76E-43	0,49870366	0,653	0,15	9,15E-39	DNAJB6	UPP	M2_vs_M0
1,21E-24	0,49812957	0,162	0	4,02E-20	KCNQ1OT1	UPP	M2_vs_M0
1,34E-39	0,49783581	0,374	0,04	4,43E-35	CALU	UPP	M2_vs_M0
7,02E-45	0,49707679	0,544	0,09	2,33E-40	BNIP3L	UPP	M2_vs_M0
6,39E-18	0,49693213	0,143	0,01	2,12E-13	TNF	UPP	M2_vs_M0
1,39E-37	0,4963554	0,294	0,02	4,62E-33	TAGAP	UPP	M2_vs_M0
8,72E-43	0,49620167	0,552	0,1	2,89E-38	BRI3	UPP	M2_vs_M0
3,01E-38	0,49620047	0,263	0,01	9,98E-34	HGSNAT	UPP	M2_vs_M0
3,91E-42	0,49609122	0,39	0,04	1,29E-37	IL18BP	UPP	M2_vs_M0
4,28E-24	0,49560752	0,753	0,3	1,42E-19	LITAF	UPP	M2_vs_M0
3,95E-46	0,49513419	0,424	0,04	1,31E-41	DAPK1	UPP	M2_vs_M0
5,64E-39	0,49476776	0,361	0,04	1,87E-34	FAM133B	UPP	M2_vs_M0
5,23E-41	0,49440163	0,31	0,02	1,73E-36	FNDC3B	UPP	M2_vs_M0
4,99E-43	0,49405552	0,401	0,04	1,65E-38	TRIM14	UPP	M2_vs_M0
9,02E-42	0,49239032	0,387	0,04	2,99E-37	SLC8A1	UPP	M2_vs_M0
4,27E-42	0,4923282	0,326	0,02	1,42E-37	NRIP1	UPP	M2_vs_M0
1,67E-38	0,49197782	0,294	0,02	5,54E-34	ARID1A	UPP	M2_vs_M0
5,48E-22	0,49128729	0,188	0,02	1,82E-17	TSC22D1	UPP	M2_vs_M0
1,86E-40	0,49124158	0,366	0,04	6,16E-36	LMAN1	UPP	M2_vs_M0
1,55E-54	0,49110757	0,523	0,06	5,14E-50	ATP6AP1	UPP	M2_vs_M0
1,75E-34	0,49013009	0,332	0,04	5,81E-30	EVI2A	UPP	M2_vs_M0
8,92E-37	0,48994886	0,355	0,04	2,96E-32	ITGA4	UPP	M2_vs_M0
1,21E-32	0,4896906	0,308	0,03	3,99E-28	FAM213A	UPP	M2_vs_M0
5,68E-44	0,48917175	0,568	0,11	1,88E-39	RBPJ	UPP	M2_vs_M0
2,53E-39	0,48902507	0,337	0,03	8,38E-35	TBC1D9	UPP	M2_vs_M0
3,01E-33	0,48874573	0,745	0,23	9,98E-29	ATP6V0B	UPP	M2_vs_M0
2,96E-40	0,48868034	0,334	0,03	9,82E-36	RRAGC	UPP	M2_vs_M0
4,74E-51	0,48850002	0,525	0,07	1,57E-46	SRSF2	UPP	M2_vs_M0
2,10E-31	0,48734422	0,225	0,01	6,96E-27	TPST2	UPP	M2_vs_M0
6,81E-43	0,48679403	0,39	0,04	2,26E-38	ENG	UPP	M2_vs_M0
1,08E-40	0,48662692	0,438	0,06	3,57E-36	TMED9	UPP	M2_vs_M0
7,61E-32	0,48661058	0,31	0,04	2,52E-27	SUPT5H	UPP	M2_vs_M0
7,06E-46	0,48531156	0,586	0,11	2,34E-41	ARHGAP18	UPP	M2_vs_M0
6,56E-40	0,48495629	0,358	0,04	2,17E-35	GLUD1	UPP	M2_vs_M0
1,34E-08	0,4845392	0,324	0,15	0,000444	MMP12	UPP	M2_vs_M0
1,26E-42	0,48412701	0,472	0,07	4,17E-38	GNAI3	UPP	M2_vs_M0
3,86E-44	0,48357649	0,39	0,04	1,28E-39	TMED5	UPP	M2_vs_M0
6,47E-24	0,48338346	0,382	0,09	2,14E-19	CFD	UPP	M2_vs_M0
1,73E-25	0,48302197	0,167	0	5,72E-21	C5AR2	UPP	M2_vs_M0
1,37E-38	0,48244919	0,294	0,02	4,55E-34	PCF11	UPP	M2_vs_M0
3,12E-44	0,48222734	0,34	0,02	1,03E-39	DOCK4	UPP	M2_vs_M0
6,91E-38	0,48217789	0,337	0,03	2,29E-33	AIG1	UPP	M2_vs_M0

1,97E-31	0,48103843	0,199	0	6,52E-27	SRGAP1	UPP	M2_vs_M0
1,10E-44	0,48013751	0,342	0,02	3,64E-40	GPRIN3	UPP	M2_vs_M0
1,01E-17	0,47999633	0,122	0	3,34E-13	EGR3	UPP	M2_vs_M0
8,73E-27	0,4798524	0,17	0	2,89E-22	ADGRG6	UPP	M2_vs_M0
9,41E-32	0,47919276	0,218	0,01	3,12E-27	FOXO1	UPP	M2_vs_M0
3,53E-22	0,47903116	0,146	0	1,17E-17	OSM	UPP	M2_vs_M0
2,24E-28	0,47824654	0,191	0	7,43E-24	EPHB2	UPP	M2_vs_M0
1,82E-37	0,47771434	0,459	0,08	6,02E-33	LMAN2	UPP	M2_vs_M0
1,02E-32	0,47762656	0,207	0	3,39E-28	PLK3	UPP	M2_vs_M0
6,97E-41	0,47751675	0,507	0,09	2,31E-36	CAST	UPP	M2_vs_M0
9,85E-52	0,47713848	0,546	0,08	3,26E-47	SF3B1	UPP	M2_vs_M0
2,11E-41	0,47616773	0,308	0,01	6,98E-37	NCEH1	UPP	M2_vs_M0
8,58E-42	0,47513875	0,56	0,11	2,84E-37	P4HB	UPP	M2_vs_M0
3,47E-43	0,47507871	0,528	0,09	1,15E-38	SCAF11	UPP	M2_vs_M0
9,15E-45	0,47476848	0,462	0,06	3,03E-40	TSPAN4	UPP	M2_vs_M0
1,24E-29	0,47454138	0,21	0,01	4,11E-25	EDEM3	UPP	M2_vs_M0
6,67E-37	0,47330496	0,294	0,02	2,21E-32	RAP2A	UPP	M2_vs_M0
1,09E-43	0,47292585	0,355	0,03	3,61E-39	ASAP1	UPP	M2_vs_M0
3,36E-28	0,47291749	0,22	0,01	1,11E-23	CD55	UPP	M2_vs_M0
1,91E-44	0,47150693	0,408	0,04	6,34E-40	GTF2I	UPP	M2_vs_M0
3,66E-44	0,47143328	0,39	0,04	1,21E-39	CMIP	UPP	M2_vs_M0
1,39E-43	0,4710351	0,34	0,02	4,60E-39	ANKRD11	UPP	M2_vs_M0
1,74E-33	0,4706423	0,268	0,02	5,76E-29	GGA1	UPP	M2_vs_M0
2,32E-28	0,47042473	0,191	0	7,68E-24	SLC7A5	UPP	M2_vs_M0
5,44E-41	0,4699642	0,324	0,02	1,80E-36	METTL7A	UPP	M2_vs_M0
1,10E-32	0,46992906	0,244	0,01	3,63E-28	SFMBT2	UPP	M2_vs_M0
2,94E-31	0,46940434	0,249	0,02	9,74E-27	SLC35F6	UPP	M2_vs_M0
1,84E-16	0,46884945	0,984	0,8	6,08E-12	MT-CYB	UPP	M2_vs_M0
3,42E-36	0,4686445	0,271	0,01	1,13E-31	SPEN	UPP	M2_vs_M0
7,07E-38	0,46859373	0,379	0,05	2,34E-33	SPPL2A	UPP	M2_vs_M0
8,85E-47	0,46779858	0,34	0,01	2,93E-42	BAZ2B	UPP	M2_vs_M0
3,48E-43	0,46771043	0,435	0,06	1,15E-38	AP2A2	UPP	M2_vs_M0
1,01E-36	0,46752472	0,393	0,06	3,36E-32	MEF2C	UPP	M2_vs_M0
8,14E-41	0,46640406	0,411	0,05	2,70E-36	IVNS1ABP	UPP	M2_vs_M0
8,92E-39	0,46631788	0,329	0,03	2,95E-34	APMAP	UPP	M2_vs_M0
9,88E-17	0,46600503	0,13	0,01	3,27E-12	EGR2	UPP	M2_vs_M0
7,01E-43	0,4655193	0,326	0,02	2,32E-38	TAOK1	UPP	M2_vs_M0
1,02E-33	0,46516156	0,26	0,01	3,39E-29	LMBRD1	UPP	M2_vs_M0
1,30E-43	0,46458531	0,618	0,13	4,31E-39	CTSA	UPP	M2_vs_M0
3,53E-32	0,46440668	0,225	0,01	1,17E-27	CYBRD1	UPP	M2_vs_M0
4,03E-39	0,46353367	0,279	0,01	1,34E-34	ZNF644	UPP	M2_vs_M0
2,87E-29	0,46347837	0,218	0,01	9,51E-25	INTS6	UPP	M2_vs_M0
2,67E-35	0,46277194	0,666	0,18	8,83E-31	CCNI	UPP	M2_vs_M0
4,08E-33	0,46195453	0,265	0,02	1,35E-28	EPC1	UPP	M2_vs_M0
1,65E-33	0,46167358	0,244	0,01	5,46E-29	SGPP1	UPP	M2_vs_M0
2,48E-17	0,46115763	0,987	0,91	8,20E-13	MT-CO3	UPP	M2_vs_M0
2,36E-34	0,46053658	0,255	0,01	7,81E-30	ADAM17	UPP	M2_vs_M0
1,25E-45	0,45981068	0,615	0,12	4,15E-41	EIF3A	UPP	M2_vs_M0
4,32E-39	0,45863008	0,279	0,01	1,43E-34	ASH1L	UPP	M2_vs_M0
1,35E-30	0,45861644	0,225	0,01	4,47E-26	KMT2A	UPP	M2_vs_M0

2,44E-44	0,45843254	0,491	0,07	8,08E-40	PTBP3	UPP	M2_vs_M0
4,42E-35	0,4582195	0,31	0,03	1,46E-30	SERTAD1	UPP	M2_vs_M0
3,64E-27	0,45810652	0,204	0,01	1,21E-22	EPAS1	UPP	M2_vs_M0
7,47E-42	0,4576519	0,334	0,02	2,47E-37	ACER3	UPP	M2_vs_M0
1,80E-36	0,45677244	0,247	0,01	5,97E-32	GATAD1	UPP	M2_vs_M0
1,10E-43	0,45669262	0,536	0,09	3,65E-39	CXCL16	UPP	M2_vs_M0
5,40E-37	0,45611461	0,353	0,04	1,79E-32	SEC31A	UPP	M2_vs_M0
1,09E-26	0,4558156	0,186	0,01	3,62E-22	C3orf58	UPP	M2_vs_M0
3,80E-40	0,45548456	0,347	0,03	1,26E-35	PDE4DIP	UPP	M2_vs_M0
1,41E-39	0,45508706	0,366	0,04	4,67E-35	PHACTR2	UPP	M2_vs_M0
3,60E-33	0,45488672	0,247	0,01	1,19E-28	TCF12	UPP	M2_vs_M0
3,46E-41	0,45477559	0,379	0,04	1,15E-36	CYTH4	UPP	M2_vs_M0
5,26E-40	0,45450937	0,273	0,01	1,74E-35	C9orf72	UPP	M2_vs_M0
1,08E-40	0,45450518	0,337	0,03	3,57E-36	CYTH1	UPP	M2_vs_M0
1,03E-35	0,45406324	0,347	0,04	3,41E-31	MAPK1IP1I	UPP	M2_vs_M0
5,73E-31	0,45397495	0,228	0,01	1,90E-26	SLC38A6	UPP	M2_vs_M0
3,56E-43	0,45378383	0,318	0,01	1,18E-38	SUSD6	UPP	M2_vs_M0
1,94E-43	0,45366354	0,385	0,04	6,42E-39	KMT2E	UPP	M2_vs_M0
2,21E-31	0,45350172	0,231	0,01	7,33E-27	AAED1	UPP	M2_vs_M0
2,62E-42	0,45336311	0,39	0,04	8,69E-38	LPP	UPP	M2_vs_M0
1,65E-27	0,45315247	0,191	0,01	5,47E-23	BRAF	UPP	M2_vs_M0
4,22E-31	0,45305522	0,313	0,04	1,40E-26	GYPC	UPP	M2_vs_M0
1,31E-14	0,45286812	0,934	0,6	4,33E-10	HLA-DQB1	UPP	M2_vs_M0
3,45E-36	0,45278397	0,313	0,03	1,14E-31	MAPK1	UPP	M2_vs_M0
1,98E-36	0,45247033	0,679	0,19	6,55E-32	UBE2D3	UPP	M2_vs_M0
3,50E-40	0,45192735	0,493	0,09	1,16E-35	DDX21	UPP	M2_vs_M0
9,02E-33	0,45190665	0,268	0,02	2,99E-28	ABR	UPP	M2_vs_M0
2,33E-52	0,45185104	0,477	0,05	7,71E-48	KTN1	UPP	M2_vs_M0
5,98E-15	0,45164673	0,989	0,79	1,98E-10	HLA-DRB1	UPP	M2_vs_M0
8,82E-40	0,4512928	0,358	0,04	2,92E-35	ME1	UPP	M2_vs_M0
4,96E-37	0,45122691	0,326	0,03	1,64E-32	PPP2CA	UPP	M2_vs_M0
8,12E-29	0,45062802	0,204	0,01	2,69E-24	KLF9	UPP	M2_vs_M0
1,05E-32	0,44921929	0,268	0,02	3,49E-28	HECA	UPP	M2_vs_M0
1,38E-45	0,44866713	0,403	0,04	4,56E-41	NORAD	UPP	M2_vs_M0
3,11E-44	0,44815067	0,408	0,04	1,03E-39	PRPF38B	UPP	M2_vs_M0
1,53E-38	0,44717644	0,424	0,06	5,06E-34	SFT2D1	UPP	M2_vs_M0
1,77E-42	0,4460628	0,629	0,14	5,85E-38	PCBP2	UPP	M2_vs_M0
9,13E-35	0,44605493	0,371	0,05	3,02E-30	OTULINL	UPP	M2_vs_M0
5,53E-38	0,44601646	0,292	0,02	1,83E-33	SLC12A7	UPP	M2_vs_M0
8,78E-35	0,44599381	0,247	0,01	2,91E-30	RBM6	UPP	M2_vs_M0
2,02E-37	0,44548432	0,302	0,02	6,69E-33	SNX29	UPP	M2_vs_M0
1,14E-36	0,44426813	0,358	0,04	3,77E-32	IDI1	UPP	M2_vs_M0
3,19E-32	0,44416759	0,231	0,01	1,06E-27	SPTLC1	UPP	M2_vs_M0
1,18E-39	0,44393895	0,347	0,03	3,91E-35	LAT2	UPP	M2_vs_M0
2,54E-42	0,44342329	0,361	0,03	8,43E-38	RBMS1	UPP	M2_vs_M0
5,53E-33	0,44236199	0,501	0,11	1,83E-28	CLEC7A	UPP	M2_vs_M0
4,41E-25	0,4420372	0,186	0,01	1,46E-20	NOTCH1	UPP	M2_vs_M0
6,95E-33	0,44203152	0,292	0,03	2,30E-28	SLC31A2	UPP	M2_vs_M0
1,63E-48	0,44132705	0,43	0,04	5,38E-44	FOXN3	UPP	M2_vs_M0
1,14E-41	0,44116216	0,374	0,04	3,76E-37	INPP5D	UPP	M2_vs_M0

3,40E-43	0,44046731	0,398	0,04	1,13E-38	SETX	UPP	M2_vs_M0
4,79E-25	0,44041874	0,18	0,01	1,59E-20	ZYG11B	UPP	M2_vs_M0
3,25E-21	0,44021008	0,629	0,24	1,08E-16	CYCS	UPP	M2_vs_M0
3,73E-39	0,43985968	0,576	0,12	1,24E-34	NINJ1	UPP	M2_vs_M0
4,55E-36	0,43963234	0,26	0,01	1,51E-31	KDSR	UPP	M2_vs_M0
2,12E-41	0,4395759	0,308	0,01	7,01E-37	MFSD12	UPP	M2_vs_M0
5,48E-41	0,43919644	0,374	0,04	1,82E-36	OGFRL1	UPP	M2_vs_M0
1,12E-47	0,43894172	0,509	0,07	3,72E-43	CCDC88A	UPP	M2_vs_M0
7,84E-25	0,43855844	0,194	0,01	2,60E-20	ARSA	UPP	M2_vs_M0
5,90E-34	0,43808953	0,276	0,02	1,95E-29	GLG1	UPP	M2_vs_M0
1,60E-25	0,43770255	0,183	0,01	5,31E-21	FOXK2	UPP	M2_vs_M0
9,39E-36	0,43739827	0,297	0,02	3,11E-31	PHKB	UPP	M2_vs_M0
1,52E-27	0,43734698	0,225	0,02	5,03E-23	NENF	UPP	M2_vs_M0
7,53E-14	0,43731088	0,939	0,56	2,50E-09	HLA-C	UPP	M2_vs_M0
2,62E-31	0,43727314	0,215	0,01	8,68E-27	NFIL3	UPP	M2_vs_M0
6,50E-33	0,43697782	0,26	0,02	2,15E-28	LNPEP	UPP	M2_vs_M0
5,81E-36	0,43642346	0,284	0,02	1,93E-31	USP7	UPP	M2_vs_M0
5,08E-35	0,43612462	0,31	0,03	1,68E-30	HPS3	UPP	M2_vs_M0
8,19E-35	0,43606948	0,286	0,02	2,71E-30	ZMIZ1	UPP	M2_vs_M0
1,23E-32	0,43457675	0,308	0,03	4,07E-28	ZNF330	UPP	M2_vs_M0
5,69E-35	0,43422762	0,292	0,02	1,89E-30	NPTN	UPP	M2_vs_M0
1,38E-32	0,4340432	0,382	0,06	4,56E-28	SYAP1	UPP	M2_vs_M0
5,74E-31	0,43366998	0,202	0	1,90E-26	ELMSAN1	UPP	M2_vs_M0
3,88E-30	0,43338218	0,212	0,01	1,29E-25	NDEL1	UPP	M2_vs_M0
2,28E-41	0,43301856	0,345	0,03	7,55E-37	DDX6	UPP	M2_vs_M0
1,24E-37	0,43253872	0,345	0,04	4,10E-33	USP15	UPP	M2_vs_M0
1,24E-42	0,43219616	0,462	0,07	4,12E-38	ATF4	UPP	M2_vs_M0
2,29E-31	0,43203353	0,21	0	7,57E-27	AC004687.	UPP	M2_vs_M0
2,50E-41	0,43193191	0,377	0,04	8,28E-37	DICER1	UPP	M2_vs_M0
1,03E-19	0,43168409	0,135	0	3,41E-15	RCAN1	UPP	M2_vs_M0
6,69E-26	0,43163702	0,191	0,01	2,22E-21	BMPR2	UPP	M2_vs_M0
2,50E-15	0,43163647	1	0,94	8,27E-11	B2M	UPP	M2_vs_M0
5,23E-31	0,43147614	0,313	0,04	1,73E-26	SIGLEC10	UPP	M2_vs_M0
2,46E-38	0,43078068	0,308	0,02	8,14E-34	ITPRIPL2	UPP	M2_vs_M0
5,56E-30	0,43057394	0,236	0,01	1,84E-25	MYLIP	UPP	M2_vs_M0
6,68E-30	0,43040267	0,236	0,01	2,21E-25	GBA	UPP	M2_vs_M0
9,35E-13	0,42953172	0,894	0,62	3,10E-08	UBC	UPP	M2_vs_M0
2,95E-36	0,4295189	0,321	0,03	9,78E-32	EPB41L2	UPP	M2_vs_M0
4,39E-39	0,42907462	0,411	0,06	1,46E-34	BSG	UPP	M2_vs_M0
8,25E-42	0,42857882	0,358	0,03	2,73E-37	FRMD4A	UPP	M2_vs_M0
5,07E-38	0,42815672	0,451	0,08	1,68E-33	SELENOS	UPP	M2_vs_M0
5,46E-36	0,42667001	0,308	0,03	1,81E-31	HECTD1	UPP	M2_vs_M0
4,60E-31	0,42583939	0,271	0,02	1,52E-26	RP2	UPP	M2_vs_M0
1,18E-41	0,42453314	0,464	0,07	3,92E-37	RAB1A	UPP	M2_vs_M0
3,31E-30	0,42410938	0,228	0,01	1,10E-25	PRDM2	UPP	M2_vs_M0
2,34E-35	0,42404738	0,294	0,02	7,74E-31	ARHGAP21	UPP	M2_vs_M0
5,08E-40	0,42374332	0,294	0,01	1,68E-35	SLC9A9	UPP	M2_vs_M0
7,57E-37	0,42322807	0,308	0,02	2,51E-32	SLC31A1	UPP	M2_vs_M0
4,68E-32	0,42307044	0,273	0,02	1,55E-27	BDP1	UPP	M2_vs_M0
1,06E-32	0,42205782	0,435	0,09	3,50E-28	MMP9	UPP	M2_vs_M0

5,23E-33	0,42173592	0,297	0,03	1,73E-28	HDGF	UPP	M2_vs_M0
1,42E-20	0,42137903	0,151	0,01	4,71E-16	SLC15A4	UPP	M2_vs_M0
2,38E-28	0,4212612	0,196	0,01	7,87E-24	ABCD4	UPP	M2_vs_M0
1,44E-38	0,42114764	0,371	0,04	4,77E-34	SLC16A3	UPP	M2_vs_M0
1,84E-25	0,42096647	0,172	0	6,10E-21	PPTC7	UPP	M2_vs_M0
1,84E-42	0,42060285	0,477	0,07	6,08E-38	SYK	UPP	M2_vs_M0
2,63E-31	0,42053042	0,215	0,01	8,73E-27	SLC11A2	UPP	M2_vs_M0
1,35E-36	0,42013709	0,302	0,02	4,46E-32	MED13L	UPP	M2_vs_M0
4,41E-42	0,41899219	0,451	0,07	1,46E-37	RBM25	UPP	M2_vs_M0
1,99E-46	0,41880662	0,509	0,08	6,59E-42	CSNK1A1	UPP	M2_vs_M0
3,04E-29	0,41872344	0,191	0	1,01E-24	RLF	UPP	M2_vs_M0
1,18E-29	0,4187171	0,199	0	3,91E-25	SRGAP3	UPP	M2_vs_M0
7,47E-35	0,417973	0,321	0,03	2,47E-30	LILRB1	UPP	M2_vs_M0
5,37E-32	0,41696089	0,345	0,05	1,78E-27	CYB561A3	UPP	M2_vs_M0
1,79E-44	0,41631726	0,355	0,02	5,93E-40	WAC	UPP	M2_vs_M0
6,87E-37	0,41623791	0,687	0,18	2,28E-32	PCBP1	UPP	M2_vs_M0
1,63E-34	0,41615031	0,406	0,07	5,39E-30	CDC42SE1	UPP	M2_vs_M0
4,74E-37	0,41554752	0,318	0,03	1,57E-32	TGFBR2	UPP	M2_vs_M0
1,77E-33	0,41551056	0,273	0,02	5,87E-29	WIPI2	UPP	M2_vs_M0
6,00E-35	0,41516282	0,318	0,03	1,99E-30	SH3BP2	UPP	M2_vs_M0
4,02E-27	0,41507037	0,194	0,01	1,33E-22	USP37	UPP	M2_vs_M0
2,39E-35	0,41503925	0,467	0,09	7,93E-31	COMT	UPP	M2_vs_M0
4,47E-34	0,41460429	0,443	0,08	1,48E-29	IDH1	UPP	M2_vs_M0
4,48E-41	0,41437281	0,446	0,07	1,48E-36	ADAR	UPP	M2_vs_M0
8,15E-38	0,41367758	0,276	0,01	2,70E-33	SP3	UPP	M2_vs_M0
1,91E-33	0,41362674	0,239	0,01	6,31E-29	DOCK11	UPP	M2_vs_M0
3,34E-46	0,41276391	0,403	0,04	1,11E-41	RAPGEF1	UPP	M2_vs_M0
9,33E-35	0,41259408	0,472	0,09	3,09E-30	YWHAH	UPP	M2_vs_M0
1,77E-32	0,41186752	0,257	0,02	5,87E-28	ACSL3	UPP	M2_vs_M0
1,59E-35	0,41168755	0,281	0,02	5,26E-31	IBTK	UPP	M2_vs_M0
3,48E-32	0,41165836	0,231	0,01	1,15E-27	TARDBP	UPP	M2_vs_M0
6,97E-30	0,41136352	0,276	0,03	2,31E-25	FKBP15	UPP	M2_vs_M0
3,62E-46	0,4113283	0,454	0,06	1,20E-41	FAM120A	UPP	M2_vs_M0
4,66E-30	0,41130795	0,218	0,01	1,54E-25	HRH1	UPP	M2_vs_M0
1,91E-33	0,41119252	0,268	0,02	6,32E-29	TMEM51	UPP	M2_vs_M0
2,88E-34	0,41077318	0,615	0,16	9,54E-30	PDIA3	UPP	M2_vs_M0
7,17E-36	0,41053965	0,337	0,04	2,38E-31	FAM177A1	UPP	M2_vs_M0
3,57E-35	0,41050817	0,249	0,01	1,18E-30	MUC20-OT	UPP	M2_vs_M0
9,11E-31	0,4104962	0,236	0,01	3,02E-26	CASC4	UPP	M2_vs_M0
1,18E-30	0,41035983	0,231	0,01	3,92E-26	POGZ	UPP	M2_vs_M0
5,80E-30	0,40987509	0,241	0,02	1,92E-25	CLCN7	UPP	M2_vs_M0
2,76E-36	0,40924429	0,408	0,06	9,15E-32	CD302	UPP	M2_vs_M0
4,69E-36	0,40861433	0,26	0,01	1,56E-31	ITGA9	UPP	M2_vs_M0
5,10E-26	0,40859593	0,22	0,02	1,69E-21	SNX14	UPP	M2_vs_M0
1,76E-40	0,40852263	0,544	0,11	5,83E-36	PLEKHB2	UPP	M2_vs_M0
1,47E-44	0,40821345	0,531	0,09	4,85E-40	PAPOLA	UPP	M2_vs_M0
1,91E-25	0,4069297	0,207	0,01	6,34E-21	SETD7	UPP	M2_vs_M0
9,85E-35	0,40618486	0,241	0,01	3,26E-30	OGA	UPP	M2_vs_M0
1,08E-26	0,40561862	0,22	0,02	3,59E-22	SMAD2	UPP	M2_vs_M0
2,76E-40	0,40500003	0,393	0,05	9,15E-36	CSF2RA	UPP	M2_vs_M0

6,27E-28	0,40361146	0,223	0,01	2,08E-23	ARHGEF12	UPP	M2_vs_M0
4,15E-13	0,40301429	0,971	0,68	1,38E-08	CST3	UPP	M2_vs_M0
1,39E-20	0,40301317	0,135	0	4,62E-16	CEMIP2	UPP	M2_vs_M0
2,10E-26	0,40282284	0,199	0,01	6,97E-22	KIAA1109	UPP	M2_vs_M0
1,77E-40	0,40267511	0,599	0,13	5,87E-36	SON	UPP	M2_vs_M0
8,16E-34	0,40151664	0,271	0,02	2,70E-29	PHACTR4	UPP	M2_vs_M0
9,30E-39	0,40081873	0,507	0,1	3,08E-34	VCP	UPP	M2_vs_M0
2,18E-32	0,40046166	0,318	0,04	7,22E-28	ARHGEF2	UPP	M2_vs_M0
3,63E-36	0,39978518	0,308	0,03	1,20E-31	EMC10	UPP	M2_vs_M0
1,61E-40	0,39977136	0,332	0,02	5,34E-36	BPTF	UPP	M2_vs_M0
6,54E-31	0,39941027	0,223	0,01	2,17E-26	RHBDF2	UPP	M2_vs_M0
4,21E-40	0,39911098	0,592	0,13	1,40E-35	PPT1	UPP	M2_vs_M0
3,32E-24	0,39900547	0,175	0,01	1,10E-19	GPR132	UPP	M2_vs_M0
1,02E-39	0,39898073	0,326	0,02	3,36E-35	ADPGK	UPP	M2_vs_M0
7,00E-32	0,39895716	0,263	0,02	2,32E-27	RCC2	UPP	M2_vs_M0
2,26E-37	0,39875439	0,273	0,01	7,50E-33	LENG8	UPP	M2_vs_M0
3,69E-31	0,39832542	0,276	0,03	1,22E-26	ADAM10	UPP	M2_vs_M0
6,77E-31	0,39798654	0,305	0,04	2,24E-26	PLBD1	UPP	M2_vs_M0
1,20E-29	0,39795205	0,215	0,01	3,96E-25	TLR7	UPP	M2_vs_M0
4,16E-32	0,39712489	0,26	0,02	1,38E-27	MTMR6	UPP	M2_vs_M0
5,86E-29	0,39682717	0,225	0,01	1,94E-24	EVI5	UPP	M2_vs_M0
2,64E-36	0,39632027	0,34	0,04	8,75E-32	ARAP1	UPP	M2_vs_M0
1,59E-27	0,39627106	0,191	0,01	5,27E-23	SIRT1	UPP	M2_vs_M0
1,31E-29	0,39596148	0,21	0,01	4,34E-25	MFHAS1	UPP	M2_vs_M0
4,08E-38	0,39563306	0,353	0,04	1,35E-33	ACP2	UPP	M2_vs_M0
3,43E-22	0,39529027	0,162	0,01	1,14E-17	HPCAL1	UPP	M2_vs_M0
2,28E-28	0,39528435	0,191	0	7,55E-24	EAF1	UPP	M2_vs_M0
9,29E-30	0,39453017	0,249	0,02	3,08E-25	CAB39	UPP	M2_vs_M0
3,94E-35	0,39441722	0,284	0,02	1,31E-30	MAPK6	UPP	M2_vs_M0
6,37E-37	0,39415276	0,265	0,01	2,11E-32	CLIP1	UPP	M2_vs_M0
5,16E-40	0,39406358	0,369	0,04	1,71E-35	HOOK3	UPP	M2_vs_M0
1,91E-25	0,39337453	0,172	0	6,34E-21	DOCK7	UPP	M2_vs_M0
2,47E-35	0,39285816	0,308	0,03	8,17E-31	WDR82	UPP	M2_vs_M0
1,75E-35	0,39259651	0,3	0,02	5,79E-31	ZNFX1	UPP	M2_vs_M0
1,07E-34	0,39255584	0,313	0,03	3,53E-30	EIF4G1	UPP	M2_vs_M0
9,76E-23	0,39248886	0,175	0,01	3,23E-18	STON2	UPP	M2_vs_M0
1,42E-36	0,39194072	0,263	0,01	4,69E-32	POLR2J3.1	UPP	M2_vs_M0
2,51E-34	0,39183633	0,249	0,01	8,33E-30	EMD	UPP	M2_vs_M0
4,91E-32	0,39174661	0,255	0,02	1,63E-27	SPTY2D1	UPP	M2_vs_M0
4,34E-25	0,39168078	0,186	0,01	1,44E-20	PCYOX1	UPP	M2_vs_M0
2,63E-34	0,39116035	0,239	0,01	8,70E-30	MAP3K3	UPP	M2_vs_M0
2,61E-24	0,39105046	0,186	0,01	8,65E-20	DIP2B	UPP	M2_vs_M0
8,31E-24	0,39060289	0,806	0,32	2,75E-19	TMEM176I	UPP	M2_vs_M0
6,74E-37	0,39010542	0,294	0,02	2,23E-32	ZBTB4	UPP	M2_vs_M0
6,87E-36	0,38970306	0,302	0,02	2,28E-31	LPCAT2	UPP	M2_vs_M0
1,56E-35	0,38956675	0,286	0,02	5,18E-31	CCPG1	UPP	M2_vs_M0
1,10E-28	0,38944041	0,289	0,04	3,64E-24	ME2	UPP	M2_vs_M0
4,70E-30	0,38939335	0,202	0	1,56E-25	CCNT1	UPP	M2_vs_M0
3,46E-29	0,38886635	0,202	0,01	1,15E-24	DYRK1A	UPP	M2_vs_M0
5,29E-22	0,38873959	0,175	0,01	1,75E-17	ZNRF2	UPP	M2_vs_M0

2,45E-38	0,38870513	0,326	0,03	8,12E-34	PHIP	UPP	M2_vs_M0
5,14E-38	0,38842817	0,517	0,1	1,70E-33	PTP4A2	UPP	M2_vs_M0
1,08E-34	0,38790988	0,308	0,03	3,59E-30	RNMT	UPP	M2_vs_M0
1,55E-33	0,38741865	0,292	0,03	5,15E-29	PRKDC	UPP	M2_vs_M0
6,52E-32	0,38739425	0,268	0,02	2,16E-27	TNKS2	UPP	M2_vs_M0
2,05E-34	0,38670327	0,292	0,02	6,80E-30	MOB3A	UPP	M2_vs_M0
1,80E-25	0,38652258	0,22	0,02	5,95E-21	GCNT1	UPP	M2_vs_M0
7,12E-39	0,38610558	0,353	0,04	2,36E-34	PNN	UPP	M2_vs_M0
5,13E-25	0,38609066	0,231	0,02	1,70E-20	TMEM138	UPP	M2_vs_M0
1,58E-33	0,38601078	0,249	0,01	5,24E-29	KDM7A	UPP	M2_vs_M0
7,15E-39	0,38563461	0,355	0,04	2,37E-34	NIPBL	UPP	M2_vs_M0
4,02E-36	0,38506767	0,43	0,07	1,33E-31	DPYSL2	UPP	M2_vs_M0
2,14E-41	0,38491922	0,355	0,03	7,10E-37	OSBPL1A	UPP	M2_vs_M0
1,26E-35	0,38462605	0,236	0	4,17E-31	WDFY3	UPP	M2_vs_M0
2,86E-27	0,38448977	0,21	0,01	9,48E-23	FRYL	UPP	M2_vs_M0
3,73E-30	0,3844036	0,196	0	1,24E-25	NCOA2	UPP	M2_vs_M0
1,68E-28	0,38412278	0,218	0,01	5,55E-24	PDGFC	UPP	M2_vs_M0
3,46E-32	0,38381825	0,231	0,01	1,15E-27	MKLN1	UPP	M2_vs_M0
4,47E-32	0,38346245	0,255	0,02	1,48E-27	CMTM7	UPP	M2_vs_M0
2,27E-38	0,38336606	0,371	0,04	7,51E-34	ETS2	UPP	M2_vs_M0
7,07E-23	0,38332669	0,199	0,02	2,34E-18	HSD17B14	UPP	M2_vs_M0
3,80E-32	0,38277755	0,305	0,03	1,26E-27	GGA2	UPP	M2_vs_M0
3,04E-44	0,3827734	0,34	0,02	1,01E-39	KMT2C	UPP	M2_vs_M0
3,06E-35	0,38258618	0,26	0,01	1,01E-30	TPCN1	UPP	M2_vs_M0
1,77E-26	0,38233773	0,204	0,01	5,88E-22	ANO6	UPP	M2_vs_M0
1,50E-31	0,38176673	0,247	0,01	4,97E-27	MCOLN1	UPP	M2_vs_M0
1,25E-19	0,38172405	0,159	0,01	4,14E-15	MFN2	UPP	M2_vs_M0
8,81E-32	0,38082376	0,233	0,01	2,92E-27	ZBTB20	UPP	M2_vs_M0
4,17E-33	0,38059076	0,247	0,01	1,38E-28	ADCY7	UPP	M2_vs_M0
6,89E-37	0,37990556	0,446	0,08	2,28E-32	RAP2B	UPP	M2_vs_M0
3,80E-30	0,37969597	0,252	0,02	1,26E-25	STARD3NL	UPP	M2_vs_M0
3,11E-36	0,37946898	0,615	0,15	1,03E-31	DAZAP2	UPP	M2_vs_M0
1,98E-31	0,37891619	0,279	0,03	6,55E-27	TMEM37	UPP	M2_vs_M0
1,69E-39	0,37883052	0,411	0,06	5,61E-35	CCDC47	UPP	M2_vs_M0
3,38E-35	0,37857293	0,316	0,03	1,12E-30	ARID4B	UPP	M2_vs_M0
1,62E-18	0,37846812	0,122	0	5,36E-14	LATS1	UPP	M2_vs_M0
4,84E-35	0,37844324	0,279	0,02	1,60E-30	EEA1	UPP	M2_vs_M0
2,06E-21	0,37768028	0,135	0	6,83E-17	FGFR1	UPP	M2_vs_M0
2,40E-28	0,37761594	0,263	0,03	7,94E-24	TCIRG1	UPP	M2_vs_M0
2,21E-27	0,37758204	0,247	0,02	7,32E-23	GAPVD1	UPP	M2_vs_M0
5,96E-34	0,37677526	0,337	0,04	1,97E-29	ACAP2	UPP	M2_vs_M0
2,31E-28	0,37656558	0,191	0	7,64E-24	TNRC6A	UPP	M2_vs_M0
2,45E-21	0,37638949	0,146	0	8,12E-17	HS3ST1	UPP	M2_vs_M0
2,11E-41	0,37634163	0,398	0,05	6,98E-37	STAT2	UPP	M2_vs_M0
7,23E-43	0,37578582	0,395	0,04	2,40E-38	COPA	UPP	M2_vs_M0
2,37E-28	0,37563256	0,202	0,01	7,85E-24	SLC37A2	UPP	M2_vs_M0
4,56E-23	0,37536	0,785	0,31	1,51E-18	MT-ND4L	UPP	M2_vs_M0
2,32E-29	0,37507984	0,223	0,01	7,69E-25	PANK3	UPP	M2_vs_M0
2,56E-42	0,37462022	0,43	0,06	8,50E-38	SF1	UPP	M2_vs_M0
1,16E-39	0,37422605	0,358	0,04	3,85E-35	EWSR1	UPP	M2_vs_M0

1,55E-36	0,37386622	0,594	0,14	5,12E-32	WASF2	UPP	M2_vs_M0
3,33E-27	0,37360439	0,172	0	1,10E-22	KLHL21	UPP	M2_vs_M0
6,45E-32	0,37332124	0,244	0,01	2,14E-27	HYOU1	UPP	M2_vs_M0
7,28E-33	0,37289044	0,255	0,01	2,41E-28	TMEM106/	UPP	M2_vs_M0
5,94E-28	0,37277445	0,194	0,01	1,97E-23	MB21D2	UPP	M2_vs_M0
5,14E-19	0,37271094	0,141	0,01	1,70E-14	GPX3	UPP	M2_vs_M0
1,00E-24	0,37253658	0,188	0,01	3,32E-20	RAPGEF2	UPP	M2_vs_M0
5,98E-37	0,37247314	0,3	0,02	1,98E-32	CRK	UPP	M2_vs_M0
1,55E-32	0,37210029	0,316	0,04	5,13E-28	SBDS	UPP	M2_vs_M0
1,27E-22	0,37209275	0,17	0,01	4,20E-18	OLFML2B	UPP	M2_vs_M0
1,67E-30	0,37164192	0,249	0,02	5,55E-26	UBR4	UPP	M2_vs_M0
4,91E-33	0,37139557	0,236	0,01	1,63E-28	DDHD1	UPP	M2_vs_M0
1,00E-26	0,37137383	0,175	0	3,32E-22	TMCC3	UPP	M2_vs_M0
4,37E-23	0,37131161	0,172	0,01	1,45E-18	SCAMP4	UPP	M2_vs_M0
1,91E-25	0,37121222	0,178	0,01	6,33E-21	SLC18B1	UPP	M2_vs_M0
4,51E-41	0,37111358	0,353	0,03	1,50E-36	STT3B	UPP	M2_vs_M0
3,98E-40	0,37090593	0,411	0,05	1,32E-35	IRF2BP2	UPP	M2_vs_M0
7,52E-24	0,37052614	0,178	0,01	2,49E-19	SNHG15	UPP	M2_vs_M0
2,85E-25	0,37038567	0,202	0,01	9,43E-21	TSPAN3	UPP	M2_vs_M0
3,34E-29	0,3702938	0,207	0,01	1,11E-24	TMX3	UPP	M2_vs_M0
6,76E-26	0,37005532	0,215	0,02	2,24E-21	ZFP91	UPP	M2_vs_M0
5,21E-33	0,36996787	0,225	0,01	1,73E-28	PTGS1	UPP	M2_vs_M0
3,14E-24	0,36988624	0,159	0	1,04E-19	PCNX4	UPP	M2_vs_M0
5,15E-27	0,36979892	0,218	0,01	1,71E-22	NCSTN	UPP	M2_vs_M0
3,20E-25	0,36973918	0,215	0,02	1,06E-20	NFATC2	UPP	M2_vs_M0
1,35E-27	0,3695176	0,207	0,01	4,47E-23	TRIP11	UPP	M2_vs_M0
1,05E-33	0,36843455	0,385	0,06	3,49E-29	SLC3A2	UPP	M2_vs_M0
1,58E-27	0,36839564	0,191	0,01	5,24E-23	ago-03	UPP	M2_vs_M0
5,50E-22	0,36819155	0,175	0,01	1,82E-17	PPARD	UPP	M2_vs_M0
2,70E-29	0,36776611	0,218	0,01	8,94E-25	SFXN3	UPP	M2_vs_M0
8,48E-36	0,36774983	0,35	0,04	2,81E-31	TRIP12	UPP	M2_vs_M0
8,95E-22	0,36709369	0,159	0,01	2,96E-17	ARFGEF1	UPP	M2_vs_M0
7,24E-33	0,36706563	0,255	0,01	2,40E-28	ZKSCAN1	UPP	M2_vs_M0
3,76E-16	0,36688114	0,117	0,01	1,24E-11	ICMT	UPP	M2_vs_M0
1,92E-25	0,36687963	0,172	0	6,37E-21	KIAA0319L	UPP	M2_vs_M0
3,17E-12	0,3665992	0,989	0,91	1,05E-07	MT-ATP6	UPP	M2_vs_M0
6,37E-27	0,36632718	0,231	0,02	2,11E-22	RUNX1	UPP	M2_vs_M0
1,39E-22	0,36614492	0,159	0,01	4,61E-18	TP53INP2	UPP	M2_vs_M0
3,76E-36	0,3660417	0,271	0,01	1,24E-31	ABCC5	UPP	M2_vs_M0
6,34E-42	0,36590019	0,371	0,04	2,10E-37	ITSN2	UPP	M2_vs_M0
7,30E-23	0,36578614	0,199	0,02	2,42E-18	VCPIP1	UPP	M2_vs_M0
6,27E-32	0,36567855	0,302	0,03	2,08E-27	FAM107B	UPP	M2_vs_M0
2,35E-44	0,36558848	0,483	0,07	7,80E-40	JAK1	UPP	M2_vs_M0
1,10E-28	0,36558646	0,247	0,02	3,63E-24	NFKB2	UPP	M2_vs_M0
7,29E-26	0,36508774	0,18	0,01	2,41E-21	IKBKB	UPP	M2_vs_M0
1,36E-22	0,36503047	0,164	0,01	4,50E-18	CYP20A1	UPP	M2_vs_M0
7,08E-24	0,36465955	0,151	0	2,35E-19	ZBTB21	UPP	M2_vs_M0
5,66E-19	0,36460755	0,159	0,01	1,87E-14	LAG3	UPP	M2_vs_M0
2,64E-30	0,36407856	0,69	0,21	8,73E-26	EIF4G2	UPP	M2_vs_M0
2,36E-31	0,36371704	0,231	0,01	7,81E-27	UBAP2L	UPP	M2_vs_M0

4,36E-28	0,36318535	0,239	0,02	1,44E-23	BCAT1	UPP	M2_vs_M0
1,06E-30	0,36283069	0,286	0,03	3,52E-26	RNF19B	UPP	M2_vs_M0
1,22E-22	0,36276524	0,143	0	4,03E-18	SLC36A1	UPP	M2_vs_M0
1,87E-33	0,36273807	0,239	0,01	6,18E-29	CDK13	UPP	M2_vs_M0
2,18E-23	0,36259727	0,207	0,02	7,23E-19	RRAGD	UPP	M2_vs_M0
5,02E-30	0,36163185	0,212	0,01	1,66E-25	ELL	UPP	M2_vs_M0
4,58E-26	0,36153086	0,202	0,01	1,52E-21	SORL1	UPP	M2_vs_M0
1,53E-20	0,36140116	0,135	0	5,07E-16	LMBR1L	UPP	M2_vs_M0
6,02E-34	0,3609483	0,276	0,02	1,99E-29	JOSD1	UPP	M2_vs_M0
8,13E-24	0,36080624	0,156	0	2,69E-19	TSPYL2	UPP	M2_vs_M0
8,72E-30	0,35993727	0,276	0,03	2,89E-25	PIK3R1	UPP	M2_vs_M0
1,67E-40	0,35984778	0,483	0,08	5,53E-36	FNBP1	UPP	M2_vs_M0
1,89E-31	0,35956498	0,279	0,03	6,25E-27	AKAP11	UPP	M2_vs_M0
3,57E-27	0,35955064	0,204	0,01	1,18E-22	SLC46A3	UPP	M2_vs_M0
2,92E-41	0,35953931	0,35	0,03	9,68E-37	LARP1	UPP	M2_vs_M0
5,11E-25	0,35939395	0,26	0,04	1,69E-20	MTMR14	UPP	M2_vs_M0
4,89E-28	0,35932827	0,247	0,02	1,62E-23	MCFD2	UPP	M2_vs_M0
1,15E-43	0,35898381	0,451	0,06	3,81E-39	SRSF11	UPP	M2_vs_M0
3,94E-18	0,35846364	0,125	0	1,31E-13	DUSP5	UPP	M2_vs_M0
1,93E-33	0,35831406	0,228	0,01	6,40E-29	SLC38A7	UPP	M2_vs_M0
9,59E-26	0,3582856	0,204	0,01	3,18E-21	MARK3	UPP	M2_vs_M0
2,46E-19	0,35810492	0,133	0	8,14E-15	ST3GAL6	UPP	M2_vs_M0
6,89E-44	0,35749491	0,52	0,09	2,28E-39	CYFIP1	UPP	M2_vs_M0
8,59E-35	0,35736174	0,3	0,03	2,84E-30	SLFN11	UPP	M2_vs_M0
1,03E-25	0,35733751	0,265	0,04	3,40E-21	VMO1	UPP	M2_vs_M0
1,67E-33	0,35725219	0,3	0,03	5,54E-29	GDE1	UPP	M2_vs_M0
1,60E-34	0,35654553	0,406	0,07	5,32E-30	CREBL2	UPP	M2_vs_M0
4,49E-36	0,35651556	0,265	0,01	1,49E-31	MAP3K1	UPP	M2_vs_M0
2,53E-42	0,3562981	0,401	0,04	8,38E-38	NAGA	UPP	M2_vs_M0
3,44E-39	0,35607545	0,464	0,08	1,14E-34	TUBA1C	UPP	M2_vs_M0
1,64E-24	0,3560561	0,756	0,28	5,44E-20	GNAI2	UPP	M2_vs_M0
6,31E-38	0,35600405	0,515	0,1	2,09E-33	TMBIM4	UPP	M2_vs_M0
9,01E-27	0,35584686	0,276	0,04	2,98E-22	PRKCB	UPP	M2_vs_M0
1,44E-31	0,35566455	0,265	0,02	4,75E-27	RBBP6	UPP	M2_vs_M0
1,99E-34	0,35561945	0,316	0,03	6,59E-30	HAVCR2	UPP	M2_vs_M0
9,16E-29	0,35552182	0,366	0,07	3,03E-24	EIF4A3	UPP	M2_vs_M0
1,37E-17	0,35535026	0,141	0,01	4,55E-13	GADD45A	UPP	M2_vs_M0
9,92E-31	0,35514066	0,241	0,01	3,29E-26	CUL3	UPP	M2_vs_M0
9,75E-28	0,35480109	0,212	0,01	3,23E-23	ITGAM	UPP	M2_vs_M0
7,81E-32	0,35467984	0,239	0,01	2,59E-27	WBP1L	UPP	M2_vs_M0
1,86E-39	0,35447937	0,347	0,03	6,16E-35	PBRM1	UPP	M2_vs_M0
1,46E-22	0,35384866	0,154	0	4,84E-18	PARP8	UPP	M2_vs_M0
3,04E-30	0,35384576	0,724	0,23	1,01E-25	TAGLN2	UPP	M2_vs_M0
3,97E-31	0,35375201	0,276	0,03	1,32E-26	CCNH	UPP	M2_vs_M0
1,15E-37	0,35360065	0,3	0,02	3,82E-33	WDR26	UPP	M2_vs_M0
3,20E-28	0,35334978	0,225	0,01	1,06E-23	FEM1B	UPP	M2_vs_M0
5,40E-32	0,35314929	0,363	0,06	1,79E-27	FEZ2	UPP	M2_vs_M0
8,15E-29	0,35274514	0,215	0,01	2,70E-24	FAM13A	UPP	M2_vs_M0
2,80E-39	0,35271206	0,363	0,04	9,27E-35	CYLD	UPP	M2_vs_M0
6,29E-32	0,35259803	0,263	0,02	2,08E-27	UBE2G2	UPP	M2_vs_M0

4,99E-30	0,35244463	0,207	0,01	1,65E-25	PIKFYVE	UPP	M2_vs_M0
4,33E-36	0,35241411	0,411	0,07	1,43E-31	KDELR2	UPP	M2_vs_M0
6,32E-21	0,35228385	0,143	0	2,09E-16	PLXDC1	UPP	M2_vs_M0
1,38E-32	0,35211478	0,233	0,01	4,56E-28	ZZEF1	UPP	M2_vs_M0
1,48E-32	0,35188093	0,324	0,04	4,89E-28	CHD9	UPP	M2_vs_M0
2,51E-30	0,35184128	0,263	0,02	8,33E-26	DRAM1	UPP	M2_vs_M0
2,10E-35	0,35181525	0,347	0,04	6,95E-31	SURF4	UPP	M2_vs_M0
4,31E-42	0,35101544	0,546	0,1	1,43E-37	LRRFIP1	UPP	M2_vs_M0
1,92E-26	0,35093851	0,199	0,01	6,36E-22	ago-02	UPP	M2_vs_M0
3,27E-34	0,35044933	0,318	0,03	1,08E-29	CD47	UPP	M2_vs_M0
1,88E-28	0,35022821	0,236	0,02	6,21E-24	LBR	UPP	M2_vs_M0
2,89E-29	0,35021257	0,191	0	9,56E-25	RNF139	UPP	M2_vs_M0
2,89E-30	0,34988552	0,239	0,01	9,57E-26	REEP3	UPP	M2_vs_M0
1,06E-38	0,34914547	0,554	0,12	3,53E-34	RNF213	UPP	M2_vs_M0
2,56E-32	0,34909537	0,247	0,01	8,48E-28	RB1CC1	UPP	M2_vs_M0
4,84E-33	0,34908448	0,387	0,06	1,60E-28	P2RY13	UPP	M2_vs_M0
3,45E-37	0,3489466	0,342	0,04	1,14E-32	FNDC3A	UPP	M2_vs_M0
1,00E-35	0,3488991	0,374	0,05	3,31E-31	TMEM147	UPP	M2_vs_M0
7,42E-32	0,34841981	0,263	0,02	2,46E-27	YTHDC1	UPP	M2_vs_M0
4,07E-22	0,34832901	0,194	0,02	1,35E-17	WDR36	UPP	M2_vs_M0
6,87E-17	0,34832171	0,141	0,01	2,28E-12	GAS6	UPP	M2_vs_M0
7,23E-30	0,34809086	0,236	0,01	2,40E-25	LTBR	UPP	M2_vs_M0
6,69E-40	0,34787996	0,35	0,03	2,22E-35	SGK3	UPP	M2_vs_M0
9,61E-29	0,34777137	0,247	0,02	3,18E-24	NHLRC3	UPP	M2_vs_M0
2,91E-26	0,34762456	0,326	0,06	9,63E-22	PLA2G16	UPP	M2_vs_M0
5,23E-32	0,34746786	0,565	0,15	1,73E-27	PDIA6	UPP	M2_vs_M0
5,60E-35	0,34723967	0,531	0,12	1,85E-30	HSPB1	UPP	M2_vs_M0
2,96E-26	0,34711792	0,183	0,01	9,79E-22	ZRANB1	UPP	M2_vs_M0
1,03E-33	0,34695051	0,369	0,05	3,40E-29	KIAA0930	UPP	M2_vs_M0
3,33E-29	0,34628224	0,196	0	1,10E-24	DIP2A	UPP	M2_vs_M0
1,10E-28	0,34628174	0,26	0,03	3,66E-24	PDXDC1	UPP	M2_vs_M0
3,74E-29	0,34613999	0,236	0,02	1,24E-24	DNAJB9	UPP	M2_vs_M0
8,70E-42	0,34597534	0,406	0,05	2,88E-37	N4BP2L2	UPP	M2_vs_M0
1,09E-39	0,34565884	0,401	0,05	3,60E-35	PNRC2	UPP	M2_vs_M0
5,81E-34	0,34544241	0,257	0,01	1,92E-29	NCOA3	UPP	M2_vs_M0
3,88E-27	0,34520093	0,199	0,01	1,29E-22	AGAP3	UPP	M2_vs_M0
3,74E-30	0,34513041	0,228	0,01	1,24E-25	CSGALNAC	UPP	M2_vs_M0
3,80E-30	0,34478758	0,223	0,01	1,26E-25	SPPL3	UPP	M2_vs_M0
3,82E-26	0,34423074	0,212	0,01	1,26E-21	DDIT3	UPP	M2_vs_M0
4,12E-27	0,34340728	0,236	0,02	1,36E-22	SLC39A7	UPP	M2_vs_M0
3,77E-14	0,34311639	0,92	0,52	1,25E-09	CD63	UPP	M2_vs_M0
2,45E-21	0,34291601	0,151	0,01	8,13E-17	SLC5A3	UPP	M2_vs_M0
2,25E-38	0,3428617	0,507	0,1	7,47E-34	SPCS3	UPP	M2_vs_M0
6,68E-32	0,34275476	0,281	0,03	2,21E-27	HNRNPUL2	UPP	M2_vs_M0
9,75E-32	0,34264073	0,228	0,01	3,23E-27	MEF2D	UPP	M2_vs_M0
4,94E-36	0,342056	0,395	0,06	1,64E-31	G3BP2	UPP	M2_vs_M0
1,90E-26	0,34166089	0,265	0,03	6,31E-22	WAPL	UPP	M2_vs_M0
6,73E-24	0,34155042	0,183	0,01	2,23E-19	HPSE	UPP	M2_vs_M0
7,83E-42	0,34146044	0,459	0,07	2,59E-37	CDV3	UPP	M2_vs_M0
7,65E-37	0,34142034	0,289	0,02	2,53E-32	CACUL1	UPP	M2_vs_M0

2,38E-36	0,34128041	0,318	0,03	7,88E-32	TMF1	UPP	M2_vs_M0
4,72E-23	0,34085036	0,146	0	1,57E-18	UBP1	UPP	M2_vs_M0
2,75E-32	0,34034964	0,241	0,01	9,10E-28	THUMPD3-	UPP	M2_vs_M0
1,24E-30	0,33988962	0,286	0,03	4,09E-26	BIRC2	UPP	M2_vs_M0
1,56E-23	0,3392593	0,18	0,01	5,17E-19	IPMK	UPP	M2_vs_M0
4,00E-29	0,33911526	0,236	0,02	1,33E-24	KLF3	UPP	M2_vs_M0
4,78E-33	0,33909578	0,289	0,03	1,58E-28	DHRS3	UPP	M2_vs_M0
6,86E-35	0,33901019	0,268	0,01	2,27E-30	CYB5R1	UPP	M2_vs_M0
1,85E-25	0,33876095	0,207	0,01	6,13E-21	RARA	UPP	M2_vs_M0
2,98E-37	0,33854087	0,355	0,04	9,88E-33	RAD21	UPP	M2_vs_M0
3,32E-27	0,33852584	0,204	0,01	1,10E-22	BRI3BP	UPP	M2_vs_M0
1,74E-31	0,33842285	0,334	0,05	5,76E-27	ITM2C	UPP	M2_vs_M0
5,03E-21	0,33758721	0,159	0,01	1,67E-16	MARF1	UPP	M2_vs_M0
4,43E-28	0,33758336	0,297	0,04	1,47E-23	ALDH9A1	UPP	M2_vs_M0
6,94E-27	0,3375256	0,212	0,01	2,30E-22	S100A13	UPP	M2_vs_M0
3,96E-13	0,33721419	0,127	0,02	1,31E-08	C1orf56	UPP	M2_vs_M0
3,37E-30	0,3370758	0,228	0,01	1,12E-25	EI24	UPP	M2_vs_M0
3,57E-27	0,336999	0,204	0,01	1,18E-22	PAG1	UPP	M2_vs_M0
2,91E-32	0,33665628	0,305	0,03	9,64E-28	AKIRIN1	UPP	M2_vs_M0
2,17E-34	0,33616138	0,337	0,04	7,19E-30	CCR1	UPP	M2_vs_M0
7,56E-26	0,33615625	0,18	0,01	2,51E-21	NFAT5	UPP	M2_vs_M0
6,97E-32	0,33610065	0,281	0,03	2,31E-27	ABHD2	UPP	M2_vs_M0
9,91E-38	0,33607015	0,44	0,07	3,28E-33	ROCK1	UPP	M2_vs_M0
9,01E-27	0,33574182	0,202	0,01	2,98E-22	DMXL1	UPP	M2_vs_M0
1,56E-33	0,33566245	0,443	0,08	5,17E-29	ALDH1A1	UPP	M2_vs_M0
8,11E-35	0,3354896	0,371	0,05	2,69E-30	VEGFB	UPP	M2_vs_M0
5,71E-29	0,33546383	0,244	0,02	1,89E-24	SEC24D	UPP	M2_vs_M0
6,97E-24	0,33532308	0,178	0,01	2,31E-19	MILR1	UPP	M2_vs_M0
9,18E-29	0,33496628	0,199	0,01	3,04E-24	SIAH1	UPP	M2_vs_M0
2,23E-28	0,33469753	0,207	0,01	7,37E-24	SLC2A8	UPP	M2_vs_M0
3,08E-33	0,33459004	0,321	0,04	1,02E-28	AZIN1	UPP	M2_vs_M0
5,72E-26	0,33448861	0,22	0,02	1,90E-21	TAB2	UPP	M2_vs_M0
1,18E-33	0,33438114	0,483	0,1	3,91E-29	G3BP1	UPP	M2_vs_M0
1,00E-37	0,33421275	0,324	0,03	3,32E-33	AAK1	UPP	M2_vs_M0
1,12E-29	0,33381532	0,244	0,02	3,73E-25	TBC1D14	UPP	M2_vs_M0
1,42E-29	0,33378288	0,271	0,03	4,71E-25	VPS41	UPP	M2_vs_M0
4,42E-40	0,33351981	0,382	0,04	1,46E-35	BRD4	UPP	M2_vs_M0
5,24E-32	0,33295218	0,255	0,02	1,73E-27	RXRA	UPP	M2_vs_M0
2,00E-35	0,33290627	0,3	0,02	6,62E-31	UBAC2	UPP	M2_vs_M0
3,82E-21	0,33204493	0,204	0,02	1,26E-16	UBTF	UPP	M2_vs_M0
1,29E-24	0,33200818	0,172	0,01	4,27E-20	REV3L	UPP	M2_vs_M0
6,91E-29	0,3319304	0,22	0,01	2,29E-24	SLC8B1	UPP	M2_vs_M0
2,61E-24	0,33184657	0,186	0,01	8,65E-20	C6orf89	UPP	M2_vs_M0
2,50E-32	0,33107915	0,34	0,05	8,28E-28	RUNX3	UPP	M2_vs_M0
2,65E-27	0,33105186	0,233	0,02	8,77E-23	SETD3	UPP	M2_vs_M0
4,87E-41	0,33080182	0,493	0,08	1,61E-36	REEP5	UPP	M2_vs_M0
2,74E-17	0,33067637	0,143	0,01	9,09E-13	KMO	UPP	M2_vs_M0
4,34E-27	0,33039301	0,188	0,01	1,44E-22	NXF1	UPP	M2_vs_M0
2,51E-19	0,33000217	0,127	0	8,30E-15	MYO7A	UPP	M2_vs_M0
1,32E-24	0,32981927	0,172	0,01	4,38E-20	CTSF	UPP	M2_vs_M0

6,03E-34	0,32912859	0,308	0,03	2,00E-29	ADD1	UPP	M2_vs_M0
4,08E-28	0,32895562	0,265	0,03	1,35E-23	STK24	UPP	M2_vs_M0
2,64E-26	0,3288892	0,172	0	8,74E-22	IL1RAP	UPP	M2_vs_M0
9,47E-29	0,32821318	0,366	0,07	3,14E-24	LILRB4	UPP	M2_vs_M0
1,36E-34	0,32806806	0,321	0,03	4,51E-30	DPP9	UPP	M2_vs_M0
4,20E-32	0,32799916	0,358	0,05	1,39E-27	NAAA	UPP	M2_vs_M0
5,93E-38	0,32750133	0,406	0,06	1,96E-33	LRP1	UPP	M2_vs_M0
6,20E-28	0,32722189	0,194	0,01	2,06E-23	CD58	UPP	M2_vs_M0
4,86E-25	0,32712614	0,18	0,01	1,61E-20	ZBTB43	UPP	M2_vs_M0
3,20E-24	0,32709458	0,175	0,01	1,06E-19	CD276	UPP	M2_vs_M0
1,81E-28	0,32681596	0,218	0,01	5,99E-24	SLK	UPP	M2_vs_M0
6,30E-28	0,32660537	0,194	0,01	2,09E-23	PDZD8	UPP	M2_vs_M0
5,20E-32	0,32643364	0,353	0,05	1,72E-27	HPS1	UPP	M2_vs_M0
4,24E-41	0,32603908	0,379	0,04	1,40E-36	GPBP1	UPP	M2_vs_M0
1,11E-18	0,32558918	0,634	0,25	3,68E-14	TXNIP	UPP	M2_vs_M0
3,35E-24	0,3249551	0,175	0,01	1,11E-19	GCLM	UPP	M2_vs_M0
3,62E-20	0,32495136	0,143	0,01	1,20E-15	SLC30A1	UPP	M2_vs_M0
1,04E-29	0,32449745	0,225	0,01	3,45E-25	MAFG	UPP	M2_vs_M0
7,58E-39	0,3244929	0,52	0,1	2,51E-34	WIPF1	UPP	M2_vs_M0
4,19E-24	0,32449246	0,194	0,01	1,39E-19	RTL8C	UPP	M2_vs_M0
2,89E-26	0,32313677	0,183	0,01	9,58E-22	RHOT1	UPP	M2_vs_M0
3,18E-24	0,32281731	0,175	0,01	1,05E-19	TTC17	UPP	M2_vs_M0
1,73E-24	0,32276224	0,196	0,01	5,75E-20	GALNT6	UPP	M2_vs_M0
3,14E-24	0,32252767	0,159	0	1,04E-19	PLEC	UPP	M2_vs_M0
5,42E-33	0,32233423	0,284	0,02	1,80E-28	MED29	UPP	M2_vs_M0
3,12E-37	0,32212969	0,297	0,02	1,03E-32	CPEB4	UPP	M2_vs_M0
7,54E-32	0,32202869	0,316	0,04	2,50E-27	PRKACB	UPP	M2_vs_M0
2,23E-19	0,32199742	0,122	0	7,38E-15	KBTBD8	UPP	M2_vs_M0
6,91E-26	0,32181661	0,18	0,01	2,29E-21	SLC35E1	UPP	M2_vs_M0
9,21E-36	0,32160151	0,297	0,02	3,05E-31	DCTN4	UPP	M2_vs_M0
2,59E-29	0,32141207	0,273	0,03	8,56E-25	HIPK1	UPP	M2_vs_M0
3,55E-30	0,32126545	0,228	0,01	1,18E-25	TLR2	UPP	M2_vs_M0
9,10E-30	0,32125161	0,263	0,02	3,02E-25	CD300LF	UPP	M2_vs_M0
1,39E-32	0,32111213	0,268	0,02	4,61E-28	UBE3A	UPP	M2_vs_M0
9,68E-29	0,32030475	0,286	0,04	3,21E-24	EMC3	UPP	M2_vs_M0
4,63E-19	0,32006103	0,202	0,03	1,53E-14	AMDHD2	UPP	M2_vs_M0
1,60E-20	0,31987111	0,141	0	5,31E-16	ZBTB10	UPP	M2_vs_M0
1,76E-25	0,31985986	0,212	0,02	5,84E-21	PLCB2	UPP	M2_vs_M0
1,34E-22	0,31953494	0,159	0,01	4,44E-18	FBXO34	UPP	M2_vs_M0
1,74E-21	0,31924543	0,167	0,01	5,78E-17	BEX3	UPP	M2_vs_M0
3,43E-20	0,31890145	0,149	0,01	1,14E-15	USP38	UPP	M2_vs_M0
1,95E-25	0,31859371	0,178	0,01	6,47E-21	SATB1	UPP	M2_vs_M0
3,18E-30	0,31855808	0,252	0,02	1,05E-25	MEF2A	UPP	M2_vs_M0
2,59E-24	0,31842229	0,231	0,03	8,59E-20	FLI1	UPP	M2_vs_M0
7,33E-26	0,31813136	0,215	0,02	2,43E-21	ANTXR2	UPP	M2_vs_M0
1,36E-22	0,31789674	0,149	0	4,50E-18	RYK	UPP	M2_vs_M0
7,34E-25	0,31781189	0,363	0,08	2,43E-20	DNASE2	UPP	M2_vs_M0
1,05E-27	0,31759059	0,218	0,01	3,47E-23	TUT7	UPP	M2_vs_M0
4,71E-36	0,31749862	0,377	0,05	1,56E-31	SPG21	UPP	M2_vs_M0
4,16E-31	0,31709936	0,297	0,03	1,38E-26	CYBC1	UPP	M2_vs_M0

1,32E-22	0,31706741	0,21	0,02	4,36E-18	NFATC2IP	UPP	M2_vs_M0
2,64E-26	0,31701927	0,172	0	8,74E-22	B4GALT5	UPP	M2_vs_M0
3,84E-27	0,31683496	0,199	0,01	1,27E-22	SECISBP2L	UPP	M2_vs_M0
5,91E-28	0,31672988	0,204	0,01	1,96E-23	SH3TC1	UPP	M2_vs_M0
1,20E-27	0,3165246	0,212	0,01	3,99E-23	FMNL2	UPP	M2_vs_M0
5,17E-21	0,31639527	0,78	0,32	1,71E-16	GLUL	UPP	M2_vs_M0
3,51E-40	0,31637785	0,515	0,09	1,16E-35	GNB1	UPP	M2_vs_M0
5,60E-27	0,31622826	0,218	0,01	1,85E-22	PI4K2A	UPP	M2_vs_M0
4,36E-36	0,31620582	0,313	0,03	1,44E-31	IST1	UPP	M2_vs_M0
2,35E-21	0,31612844	0,279	0,05	7,79E-17	CCND1	UPP	M2_vs_M0
3,29E-39	0,31610837	0,488	0,09	1,09E-34	CD86	UPP	M2_vs_M0
2,78E-20	0,31603076	0,154	0,01	9,20E-16	RASGRP3	UPP	M2_vs_M0
2,92E-32	0,31569612	0,31	0,04	9,67E-28	MBP	UPP	M2_vs_M0
1,00E-35	0,31507373	0,491	0,1	3,32E-31	KPNB1	UPP	M2_vs_M0
1,12E-24	0,31475272	0,183	0,01	3,71E-20	ARMCX1	UPP	M2_vs_M0
2,16E-08	0,314396	0,936	0,63	0,000715	HSP90AA1	UPP	M2_vs_M0
9,86E-20	0,31429744	0,13	0	3,27E-15	SESN1	UPP	M2_vs_M0
6,59E-09	0,31429327	0,761	0,45	0,000218	DNASE1L3	UPP	M2_vs_M0
1,38E-31	0,314284	0,271	0,02	4,57E-27	RASSF5	UPP	M2_vs_M0
3,47E-29	0,31427575	0,255	0,02	1,15E-24	CCNY	UPP	M2_vs_M0
4,00E-18	0,31392571	0,13	0,01	1,33E-13	CRY2	UPP	M2_vs_M0
3,33E-16	0,31387227	0,122	0,01	1,10E-11	MARVELD1	UPP	M2_vs_M0
8,44E-24	0,31387113	0,172	0,01	2,80E-19	CTSO	UPP	M2_vs_M0
1,31E-32	0,31341596	0,268	0,02	4,35E-28	USP22	UPP	M2_vs_M0
8,25E-24	0,31334979	0,162	0	2,73E-19	SAMD8	UPP	M2_vs_M0
3,94E-32	0,3132191	0,313	0,04	1,31E-27	TMX1	UPP	M2_vs_M0
2,26E-32	0,31258417	0,252	0,01	7,48E-28	LCOR	UPP	M2_vs_M0
1,10E-24	0,31245988	0,178	0,01	3,65E-20	ASXL2	UPP	M2_vs_M0
5,10E-23	0,31232095	0,156	0	1,69E-18	TTC19	UPP	M2_vs_M0
1,40E-27	0,31225877	0,196	0,01	4,64E-23	SLC30A7	UPP	M2_vs_M0
9,69E-28	0,31206737	0,263	0,03	3,21E-23	PLEKHM2	UPP	M2_vs_M0
1,09E-35	0,31196611	0,374	0,05	3,60E-31	COPB1	UPP	M2_vs_M0
2,23E-21	0,31195958	0,233	0,04	7,38E-17	DDRGK1	UPP	M2_vs_M0
1,94E-13	0,31191017	0,117	0,01	6,42E-09	AP1AR	UPP	M2_vs_M0
1,18E-26	0,3118898	0,22	0,02	3,91E-22	ADORA3	UPP	M2_vs_M0
1,22E-24	0,31181154	0,178	0,01	4,03E-20	RNF145	UPP	M2_vs_M0
5,40E-23	0,31176096	0,162	0,01	1,79E-18	C20orf194	UPP	M2_vs_M0
1,49E-35	0,31162996	0,382	0,05	4,94E-31	EVL	UPP	M2_vs_M0
7,44E-28	0,31159181	0,241	0,02	2,47E-23	UBA6	UPP	M2_vs_M0
6,36E-26	0,31140526	0,191	0,01	2,11E-21	CLIP2	UPP	M2_vs_M0
2,06E-21	0,31139713	0,135	0	6,83E-17	ERN1	UPP	M2_vs_M0
6,36E-31	0,31082571	0,228	0,01	2,11E-26	NF1	UPP	M2_vs_M0
6,23E-30	0,31074248	0,273	0,03	2,06E-25	GGNBP2	UPP	M2_vs_M0
6,03E-28	0,31053986	0,204	0,01	2,00E-23	GAL3ST4	UPP	M2_vs_M0
8,47E-20	0,31052086	0,459	0,15	2,81E-15	ADAMDEC1	UPP	M2_vs_M0
1,93E-25	0,31034549	0,183	0,01	6,41E-21	SIPA1L2	UPP	M2_vs_M0
1,07E-30	0,31023656	0,273	0,03	3,53E-26	MESD	UPP	M2_vs_M0
1,95E-26	0,31009902	0,204	0,01	6,46E-22	OXSR1	UPP	M2_vs_M0
8,02E-37	0,30978387	0,491	0,09	2,66E-32	EIF1AX	UPP	M2_vs_M0
1,40E-19	0,30946913	0,172	0,02	4,63E-15	SHISA5	UPP	M2_vs_M0

1,23E-35	0,30936544	0,414	0,07	4,06E-31	MKNK1	UPP	M2_vs_M0
3,47E-33	0,30930656	0,294	0,03	1,15E-28	OSTM1	UPP	M2_vs_M0
1,06E-17	0,30907599	0,122	0	3,50E-13	TMEM65	UPP	M2_vs_M0
9,26E-20	0,30878764	0,141	0,01	3,07E-15	ENTPD6	UPP	M2_vs_M0
1,63E-40	0,30860826	0,544	0,11	5,40E-36	TCF25	UPP	M2_vs_M0
3,83E-22	0,30859438	0,151	0	1,27E-17	TMEM245	UPP	M2_vs_M0
4,72E-24	0,30796327	0,194	0,01	1,56E-19	WASHC1	UPP	M2_vs_M0
2,90E-26	0,30794971	0,188	0,01	9,59E-22	PHF10	UPP	M2_vs_M0
9,88E-30	0,30794954	0,249	0,02	3,27E-25	TRPM2	UPP	M2_vs_M0
1,71E-28	0,30792528	0,255	0,02	5,68E-24	HUWE1	UPP	M2_vs_M0
2,54E-27	0,30750119	0,247	0,02	8,42E-23	LRRC58	UPP	M2_vs_M0
2,08E-23	0,30740877	0,17	0,01	6,88E-19	LMF2	UPP	M2_vs_M0
7,89E-20	0,30738528	0,146	0,01	2,61E-15	GPR35	UPP	M2_vs_M0
3,12E-31	0,30730922	0,249	0,02	1,03E-26	IFNAR2	UPP	M2_vs_M0
4,04E-32	0,30712799	0,342	0,05	1,34E-27	GUSB	UPP	M2_vs_M0
3,24E-28	0,30698469	0,244	0,02	1,07E-23	TWF1	UPP	M2_vs_M0
4,76E-38	0,30689678	0,61	0,14	1,58E-33	TMEM59	UPP	M2_vs_M0
2,52E-41	0,3064159	0,43	0,06	8,34E-37	GTF2B	UPP	M2_vs_M0
5,66E-24	0,30626496	0,188	0,01	1,88E-19	KIAA0232	UPP	M2_vs_M0
1,47E-33	0,30621692	0,589	0,15	4,87E-29	LIMS1	UPP	M2_vs_M0
4,31E-34	0,30616127	0,355	0,05	1,43E-29	RRBP1	UPP	M2_vs_M0
1,75E-43	0,30601209	0,467	0,07	5,81E-39	CISD2	UPP	M2_vs_M0
8,74E-25	0,3059587	0,194	0,01	2,90E-20	RALGAPA1	UPP	M2_vs_M0
4,50E-31	0,3056813	0,302	0,04	1,49E-26	CD9	UPP	M2_vs_M0
3,60E-24	0,30560087	0,199	0,01	1,19E-19	CTBP2	UPP	M2_vs_M0
2,67E-24	0,30551814	0,186	0,01	8,83E-20	RELT	UPP	M2_vs_M0
4,00E-35	0,30531701	0,284	0,02	1,33E-30	BIRC6	UPP	M2_vs_M0
1,70E-36	0,30517059	0,438	0,07	5,62E-32	APH1A	UPP	M2_vs_M0
6,14E-19	0,30514708	0,22	0,04	2,03E-14	SECTM1	UPP	M2_vs_M0
7,11E-17	0,30489857	0,141	0,01	2,36E-12	AC092069.	UPP	M2_vs_M0
9,36E-20	0,30478849	0,141	0,01	3,10E-15	TUT4	UPP	M2_vs_M0
7,52E-24	0,30463972	0,178	0,01	2,49E-19	TMEM192	UPP	M2_vs_M0
3,94E-21	0,30446247	0,164	0,01	1,31E-16	SMC5	UPP	M2_vs_M0
3,36E-29	0,30437198	0,207	0,01	1,11E-24	STX16	UPP	M2_vs_M0
4,04E-26	0,3042994	0,26	0,03	1,34E-21	TENT2	UPP	M2_vs_M0
1,06E-26	0,30427793	0,196	0,01	3,52E-22	UGCG	UPP	M2_vs_M0
5,00E-23	0,3038449	0,172	0,01	1,66E-18	SHE	UPP	M2_vs_M0
1,47E-22	0,30332323	0,159	0,01	4,88E-18	TIAM1	UPP	M2_vs_M0
4,65E-36	0,30311971	0,308	0,03	1,54E-31	GCC2	UPP	M2_vs_M0
1,51E-22	0,30271951	0,231	0,03	5,01E-18	SULT1A1	UPP	M2_vs_M0
5,85E-38	0,30231569	0,385	0,05	1,94E-33	PTAFR	UPP	M2_vs_M0
5,66E-19	0,30204385	0,119	0	1,87E-14	BCAM	UPP	M2_vs_M0
1,57E-27	0,30203438	0,257	0,03	5,21E-23	FAM50A	UPP	M2_vs_M0
4,91E-24	0,30192345	0,194	0,01	1,63E-19	TMX2	UPP	M2_vs_M0
3,32E-24	0,30190111	0,175	0,01	1,10E-19	PDGFB	UPP	M2_vs_M0
9,57E-29	0,30187848	0,252	0,02	3,17E-24	ZNF148	UPP	M2_vs_M0
6,82E-28	0,301767	0,247	0,02	2,26E-23	ADGRE2	UPP	M2_vs_M0
1,48E-25	0,30111167	0,194	0,01	4,91E-21	RETREG2	UPP	M2_vs_M0
2,35E-32	0,30089922	0,34	0,05	7,77E-28	GNA15	UPP	M2_vs_M0
8,25E-28	0,30081973	0,241	0,02	2,73E-23	TMEM127	UPP	M2_vs_M0

2,64E-24	0,3003619	0,186	0,01	8,74E-20	WASHC5	UPP	M2_vs_M0
1,83E-21	0,30029455	0,162	0,01	6,07E-17	ZNF609	UPP	M2_vs_M0
2,34E-21	0,30020308	0,141	0	7,76E-17	TMEM268	UPP	M2_vs_M0
4,76E-32	0,29995614	0,69	0,2	1,58E-27	HNRNPC	UPP	M2_vs_M0
3,97E-26	0,29972939	0,43	0,11	1,32E-21	PLIN2	UPP	M2_vs_M0
3,51E-25	0,29859526	0,196	0,01	1,16E-20	SEL1L3	UPP	M2_vs_M0
2,14E-23	0,29856557	0,17	0,01	7,09E-19	AMFR	UPP	M2_vs_M0
3,27E-19	0,29815946	0,156	0,01	1,08E-14	CRYBG3	UPP	M2_vs_M0
7,86E-29	0,2981249	0,252	0,02	2,60E-24	SIL1	UPP	M2_vs_M0
3,03E-24	0,29812404	0,18	0,01	1,00E-19	CTTNBP2	UPP	M2_vs_M0
2,79E-26	0,29802005	0,188	0,01	9,24E-22	ARID3A	UPP	M2_vs_M0
8,37E-28	0,29785974	0,223	0,01	2,77E-23	UBE4A	UPP	M2_vs_M0
3,82E-28	0,29763928	0,239	0,02	1,27E-23	PITPNA	UPP	M2_vs_M0
8,45E-22	0,29744042	0,164	0,01	2,80E-17	ARNT	UPP	M2_vs_M0
6,01E-28	0,29717896	0,199	0,01	1,99E-23	SNX13	UPP	M2_vs_M0
1,11E-26	0,29707479	0,191	0,01	3,67E-22	BTAF1	UPP	M2_vs_M0
3,65E-24	0,29657023	0,199	0,01	1,21E-19	PNPLA6	UPP	M2_vs_M0
1,53E-28	0,29643459	0,223	0,01	5,08E-24	WDR6	UPP	M2_vs_M0
6,80E-19	0,29616912	0,212	0,03	2,25E-14	IL2RG	UPP	M2_vs_M0
2,67E-36	0,2952544	0,406	0,06	8,85E-32	TM9SF2	UPP	M2_vs_M0
5,44E-33	0,29522317	0,31	0,03	1,80E-28	TMEM165	UPP	M2_vs_M0
1,75E-20	0,29515791	0,17	0,01	5,81E-16	IFIH1	UPP	M2_vs_M0
9,08E-37	0,29509422	0,565	0,13	3,01E-32	NCKAP1L	UPP	M2_vs_M0
3,52E-26	0,29496275	0,252	0,03	1,17E-21	PPP3CA	UPP	M2_vs_M0
1,81E-34	0,2947644	0,35	0,04	6,00E-30	DNTTIP2	UPP	M2_vs_M0
5,63E-34	0,2946069	0,294	0,03	1,87E-29	ERGIC1	UPP	M2_vs_M0
6,21E-17	0,29395061	0,122	0,01	2,06E-12	SYNJ2BP	UPP	M2_vs_M0
4,60E-29	0,29363373	0,276	0,03	1,52E-24	PLXNB2	UPP	M2_vs_M0
3,47E-29	0,29359953	0,297	0,04	1,15E-24	TXNDC12	UPP	M2_vs_M0
6,19E-26	0,2935793	0,196	0,01	2,05E-21	GOLPH3	UPP	M2_vs_M0
8,26E-27	0,29341429	0,202	0,01	2,74E-22	SLC39A10	UPP	M2_vs_M0
1,56E-25	0,29314715	0,162	0	5,16E-21	CBLB	UPP	M2_vs_M0
8,79E-26	0,29307969	0,273	0,04	2,91E-21	ALG13	UPP	M2_vs_M0
5,97E-22	0,29298195	0,175	0,01	1,98E-17	PPM1F	UPP	M2_vs_M0
7,39E-26	0,29296303	0,186	0,01	2,45E-21	RLIM	UPP	M2_vs_M0
1,05E-18	0,29280508	0,218	0,04	3,49E-14	FCGR2B	UPP	M2_vs_M0
5,44E-23	0,29279385	0,225	0,03	1,80E-18	SMC1A	UPP	M2_vs_M0
7,51E-26	0,29263288	0,186	0,01	2,49E-21	KLF7	UPP	M2_vs_M0
4,63E-24	0,29258612	0,194	0,01	1,53E-19	RC3H1	UPP	M2_vs_M0
1,77E-23	0,2923143	0,18	0,01	5,85E-19	RNF168	UPP	M2_vs_M0
2,07E-24	0,29226343	0,191	0,01	6,85E-20	FGD4	UPP	M2_vs_M0
2,33E-29	0,29216218	0,228	0,01	7,73E-25	WASHC2A	UPP	M2_vs_M0
8,67E-21	0,29212382	0,167	0,01	2,87E-16	TRIOBP	UPP	M2_vs_M0
1,12E-26	0,29199073	0,191	0,01	3,71E-22	MEPCE	UPP	M2_vs_M0
2,28E-23	0,29197942	0,164	0,01	7,54E-19	ZDHHC14	UPP	M2_vs_M0
3,07E-15	0,29194011	0,212	0,05	1,02E-10	ALOX5AP	UPP	M2_vs_M0
2,55E-19	0,29151635	0,133	0	8,45E-15	HIVEP1	UPP	M2_vs_M0
4,92E-25	0,29139185	0,18	0,01	1,63E-20	DIAPH2	UPP	M2_vs_M0
6,87E-34	0,29133769	0,329	0,04	2,28E-29	GLB1	UPP	M2_vs_M0
1,94E-22	0,29121941	0,196	0,02	6,44E-18	CDC73	UPP	M2_vs_M0

1,06E-26	0,2906776	0,247	0,03	3,50E-22	TMED7	UPP	M2_vs_M0
9,46E-26	0,28996208	0,223	0,02	3,14E-21	SMARCD2	UPP	M2_vs_M0
1,26E-22	0,28977523	0,17	0,01	4,18E-18	CSF3R	UPP	M2_vs_M0
1,15E-38	0,28968519	0,523	0,1	3,81E-34	SRRM2	UPP	M2_vs_M0
1,02E-29	0,28965934	0,249	0,02	3,37E-25	TNRC6B	UPP	M2_vs_M0
1,48E-18	0,28951575	0,133	0,01	4,92E-14	TSC2	UPP	M2_vs_M0
1,62E-28	0,28915863	0,241	0,02	5,38E-24	RREB1	UPP	M2_vs_M0
4,25E-16	0,28911876	0,111	0	1,41E-11	ZFY	UPP	M2_vs_M0
1,82E-23	0,2887559	0,212	0,02	6,01E-19	PPP3R1	UPP	M2_vs_M0
2,98E-27	0,28865156	0,21	0,01	9,87E-23	ZNF451	UPP	M2_vs_M0
9,29E-18	0,28831448	0,133	0,01	3,08E-13	SLF2	UPP	M2_vs_M0
1,24E-22	0,28823742	0,17	0,01	4,09E-18	ATP10D	UPP	M2_vs_M0
5,21E-36	0,28761615	0,395	0,06	1,73E-31	HIST1H4C	UPP	M2_vs_M0
1,23E-28	0,28756526	0,247	0,02	4,06E-24	TLE4	UPP	M2_vs_M0
1,12E-40	0,28729068	0,408	0,05	3,71E-36	IFNAR1	UPP	M2_vs_M0
5,82E-15	0,28713915	0,109	0,01	1,93E-10	MAP3K7	UPP	M2_vs_M0
3,26E-32	0,28712535	0,408	0,07	1,08E-27	SELENOF	UPP	M2_vs_M0
1,72E-34	0,28708666	0,289	0,02	5,70E-30	GANAB	UPP	M2_vs_M0
5,34E-23	0,28707232	0,167	0,01	1,77E-18	KANSL1	UPP	M2_vs_M0
1,44E-33	0,28666139	0,255	0,01	4,77E-29	SETD2	UPP	M2_vs_M0
5,13E-23	0,28663077	0,204	0,02	1,70E-18	RBM12	UPP	M2_vs_M0
6,28E-36	0,28658074	0,454	0,08	2,08E-31	TUBB4B	UPP	M2_vs_M0
3,79E-22	0,28653166	0,151	0	1,25E-17	SAV1	UPP	M2_vs_M0
9,15E-23	0,28621555	0,18	0,01	3,03E-18	MBNL2	UPP	M2_vs_M0
3,14E-20	0,28578343	0,186	0,02	1,04E-15	C5orf15	UPP	M2_vs_M0
3,10E-29	0,28571453	0,218	0,01	1,03E-24	ATP8B4	UPP	M2_vs_M0
2,47E-26	0,28554779	0,244	0,03	8,18E-22	SULF2	UPP	M2_vs_M0
1,82E-33	0,28550176	0,398	0,07	6,03E-29	ZNF207	UPP	M2_vs_M0
1,63E-18	0,28537897	0,122	0	5,39E-14	CHSY1	UPP	M2_vs_M0
4,08E-32	0,28530958	0,279	0,02	1,35E-27	SNX27	UPP	M2_vs_M0
1,16E-24	0,28497193	0,233	0,03	3,85E-20	OSER1	UPP	M2_vs_M0
2,74E-31	0,28482875	0,255	0,02	9,06E-27	XPO6	UPP	M2_vs_M0
2,92E-20	0,28468541	0,186	0,02	9,66E-16	DCAF12	UPP	M2_vs_M0
2,69E-27	0,28455964	0,215	0,01	8,90E-23	SPOP	UPP	M2_vs_M0
5,64E-22	0,28415636	0,793	0,32	1,87E-17	MTRNR2L8	UPP	M2_vs_M0
4,03E-39	0,28378613	0,337	0,03	1,33E-34	KDM5A	UPP	M2_vs_M0
7,52E-26	0,28375562	0,18	0,01	2,49E-21	RICTOR	UPP	M2_vs_M0
1,58E-21	0,28363299	0,199	0,02	5,24E-17	GRAMD1A	UPP	M2_vs_M0
8,41E-37	0,28329672	0,382	0,05	2,78E-32	DCAF7	UPP	M2_vs_M0
6,04E-29	0,28313168	0,225	0,01	2,00E-24	CAMK1D	UPP	M2_vs_M0
2,25E-27	0,28283697	0,35	0,07	7,45E-23	SDHC	UPP	M2_vs_M0
1,58E-40	0,28272629	0,377	0,04	5,23E-36	GAS7	UPP	M2_vs_M0
5,13E-32	0,28265592	0,501	0,11	1,70E-27	GNPDA1	UPP	M2_vs_M0
1,87E-30	0,28237431	0,268	0,02	6,19E-26	SELPLG	UPP	M2_vs_M0
5,91E-30	0,28217449	0,26	0,02	1,96E-25	RNF10	UPP	M2_vs_M0
1,47E-22	0,28117348	0,159	0,01	4,86E-18	CCNT2	UPP	M2_vs_M0
2,80E-18	0,28101815	0,141	0,01	9,26E-14	ACSL1	UPP	M2_vs_M0
2,43E-26	0,28094887	0,223	0,02	8,05E-22	NFAM1	UPP	M2_vs_M0
3,58E-31	0,28086509	0,456	0,1	1,19E-26	MPP1	UPP	M2_vs_M0
1,03E-19	0,2805202	0,178	0,02	3,42E-15	MIA2	UPP	M2_vs_M0

1,63E-35	0,28043609	0,361	0,05	5,39E-31	FCHO2	UPP	M2_vs_M0
1,55E-27	0,28038806	0,244	0,02	5,14E-23	SAFB2	UPP	M2_vs_M0
5,01E-23	0,28012511	0,196	0,02	1,66E-18	H1F0	UPP	M2_vs_M0
2,86E-26	0,28010287	0,188	0,01	9,49E-22	PCSK7	UPP	M2_vs_M0
3,18E-24	0,28006756	0,175	0,01	1,05E-19	CHST15	UPP	M2_vs_M0
4,96E-34	0,27981748	0,263	0,01	1,64E-29	USP34	UPP	M2_vs_M0
1,82E-26	0,2797711	0,204	0,01	6,03E-22	SPG7	UPP	M2_vs_M0
8,84E-21	0,27966444	0,167	0,01	2,93E-16	MTR	UPP	M2_vs_M0
5,37E-23	0,27957199	0,167	0,01	1,78E-18	G6PC3	UPP	M2_vs_M0
6,34E-28	0,27906441	0,194	0,01	2,10E-23	TET3	UPP	M2_vs_M0
1,01E-29	0,27905193	0,305	0,04	3,36E-25	SRSF4	UPP	M2_vs_M0
8,89E-18	0,27871856	0,133	0,01	2,94E-13	KIAA1147	UPP	M2_vs_M0
9,17E-18	0,27865644	0,111	0	3,04E-13	TBC1D12	UPP	M2_vs_M0
9,80E-22	0,27864035	0,149	0	3,25E-17	AC007952.	UPP	M2_vs_M0
9,15E-35	0,2782721	0,305	0,03	3,03E-30	PTP4A1	UPP	M2_vs_M0
4,49E-29	0,27825608	0,284	0,03	1,49E-24	NEMF	UPP	M2_vs_M0
8,96E-25	0,27813919	0,194	0,01	2,97E-20	SDE2	UPP	M2_vs_M0
2,48E-21	0,27811501	0,151	0,01	8,21E-17	KIAA2026	UPP	M2_vs_M0
9,11E-27	0,27801505	0,239	0,02	3,02E-22	TSPAN33	UPP	M2_vs_M0
2,46E-16	0,27787273	0,178	0,03	8,15E-12	CHTF8	UPP	M2_vs_M0
6,17E-26	0,27783947	0,233	0,02	2,05E-21	PIK3C2A	UPP	M2_vs_M0
1,08E-27	0,27781159	0,218	0,01	3,59E-23	PQLC1	UPP	M2_vs_M0
2,27E-27	0,27780341	0,26	0,03	7,51E-23	PIP4K2A	UPP	M2_vs_M0
1,37E-15	0,27766883	0,127	0,01	4,55E-11	TOR2A	UPP	M2_vs_M0
7,47E-23	0,27762375	0,199	0,02	2,47E-18	ERLEC1	UPP	M2_vs_M0
3,21E-29	0,2773934	0,212	0,01	1,06E-24	DIAPH1	UPP	M2_vs_M0
1,01E-19	0,27696733	0,191	0,02	3,34E-15	TCEAL9	UPP	M2_vs_M0
3,74E-32	0,27681862	0,265	0,02	1,24E-27	ZNF655	UPP	M2_vs_M0
2,10E-26	0,27676558	0,223	0,02	6,95E-22	TOR1AIP1	UPP	M2_vs_M0
3,92E-19	0,27664557	0,151	0,01	1,30E-14	ERAP1	UPP	M2_vs_M0
7,36E-26	0,27642499	0,271	0,04	2,44E-21	SYPL1	UPP	M2_vs_M0
1,04E-19	0,2762337	0,135	0	3,44E-15	LIMD1	UPP	M2_vs_M0
2,31E-37	0,27603469	0,496	0,09	7,66E-33	GLIPR1	UPP	M2_vs_M0
2,77E-22	0,27600213	0,172	0,01	9,17E-18	NPLOC4	UPP	M2_vs_M0
3,73E-25	0,27574475	0,721	0,25	1,24E-20	CAP1	UPP	M2_vs_M0
2,91E-26	0,27557067	0,183	0,01	9,63E-22	ANKLE2	UPP	M2_vs_M0
8,66E-24	0,27554274	0,162	0	2,87E-19	TNFRSF10E	UPP	M2_vs_M0
8,09E-29	0,27546956	0,239	0,02	2,68E-24	PSEN1	UPP	M2_vs_M0
9,00E-12	0,27542384	0,125	0,02	2,98E-07	UBE2G1	UPP	M2_vs_M0
1,60E-20	0,27541152	0,146	0,01	5,30E-16	AVPI1	UPP	M2_vs_M0
1,06E-24	0,27526296	0,188	0,01	3,50E-20	SUMF1	UPP	M2_vs_M0
2,53E-19	0,27497702	0,138	0,01	8,37E-15	SCARF1	UPP	M2_vs_M0
4,07E-18	0,27483587	0,13	0,01	1,35E-13	RPS6KA5	UPP	M2_vs_M0
2,17E-22	0,27459175	0,178	0,01	7,20E-18	RERE	UPP	M2_vs_M0
4,15E-34	0,27432323	0,34	0,04	1,37E-29	FUCA2	UPP	M2_vs_M0
4,29E-25	0,27416007	0,186	0,01	1,42E-20	TTC14	UPP	M2_vs_M0
4,43E-26	0,27398955	0,207	0,01	1,47E-21	ARRDC2	UPP	M2_vs_M0
5,08E-31	0,27384516	0,43	0,09	1,68E-26	SLAMF8	UPP	M2_vs_M0
4,13E-25	0,27352077	0,191	0,01	1,37E-20	FURIN	UPP	M2_vs_M0
8,61E-24	0,27350369	0,215	0,02	2,85E-19	CREBBP	UPP	M2_vs_M0

1,04E-24	0,27326128	0,188	0,01	3,45E-20	P2RX4	UPP	M2_vs_M0
1,27E-28	0,27292504	0,228	0,01	4,19E-24	TRPS1	UPP	M2_vs_M0
2,90E-26	0,27279807	0,218	0,02	9,62E-22	PLBD2	UPP	M2_vs_M0
1,56E-36	0,2727643	0,451	0,08	5,16E-32	SYNCRIP	UPP	M2_vs_M0
1,21E-24	0,2725572	0,162	0	4,02E-20	SCAMP1	UPP	M2_vs_M0
3,35E-19	0,27237924	0,156	0,01	1,11E-14	CCRL2	UPP	M2_vs_M0
8,13E-34	0,27229337	0,377	0,06	2,69E-29	STK4	UPP	M2_vs_M0
2,50E-27	0,27222119	0,233	0,02	8,29E-23	ATG7	UPP	M2_vs_M0
1,36E-31	0,2720627	0,284	0,03	4,51E-27	EHD4	UPP	M2_vs_M0
8,66E-28	0,27170748	0,223	0,01	2,87E-23	BICD2	UPP	M2_vs_M0
6,93E-27	0,27149974	0,231	0,02	2,29E-22	ST14	UPP	M2_vs_M0
1,91E-25	0,27123104	0,183	0,01	6,34E-21	MRC2	UPP	M2_vs_M0
4,71E-34	0,27122346	0,446	0,08	1,56E-29	SELENOT	UPP	M2_vs_M0
5,39E-08	0,2710757	0,947	0,68	0,001785	HLA-DQA1	UPP	M2_vs_M0
1,78E-21	0,27059031	0,167	0,01	5,89E-17	MGRN1	UPP	M2_vs_M0
1,65E-20	0,27058707	0,17	0,01	5,47E-16	RDH11	UPP	M2_vs_M0
3,99E-27	0,27051365	0,199	0,01	1,32E-22	FNIP1	UPP	M2_vs_M0
5,90E-34	0,27044789	0,403	0,07	1,96E-29	MORF4L2	UPP	M2_vs_M0
4,81E-22	0,27039492	0,207	0,02	1,59E-17	HTT	UPP	M2_vs_M0
8,68E-35	0,27034842	0,286	0,02	2,88E-30	BOD1L1	UPP	M2_vs_M0
4,07E-18	0,26959374	0,125	0	1,35E-13	ERCC3	UPP	M2_vs_M0
1,35E-23	0,26956144	0,204	0,02	4,48E-19	PGPEP1	UPP	M2_vs_M0
6,69E-17	0,26948027	0,117	0	2,22E-12	TBCD	UPP	M2_vs_M0
1,72E-20	0,26943065	0,204	0,03	5,71E-16	TCEAL3	UPP	M2_vs_M0
1,30E-34	0,2689995	0,411	0,07	4,30E-30	NECAP2	UPP	M2_vs_M0
8,85E-24	0,2689827	0,167	0,01	2,93E-19	ATP11C	UPP	M2_vs_M0
1,52E-20	0,26885801	0,135	0	5,04E-16	LRCH1	UPP	M2_vs_M0
3,20E-22	0,26873869	0,167	0,01	1,06E-17	DNASE1L1	UPP	M2_vs_M0
3,42E-28	0,26872428	0,244	0,02	1,13E-23	NPEPPS	UPP	M2_vs_M0
1,93E-23	0,2683418	0,199	0,02	6,41E-19	NLN	UPP	M2_vs_M0
6,30E-28	0,26782867	0,247	0,02	2,09E-23	AKNA	UPP	M2_vs_M0
3,11E-33	0,26754159	0,427	0,08	1,03E-28	RASSF4	UPP	M2_vs_M0
9,90E-26	0,26734694	0,21	0,01	3,28E-21	NFATC1	UPP	M2_vs_M0
4,62E-30	0,26702168	0,279	0,03	1,53E-25	CHID1	UPP	M2_vs_M0
1,28E-27	0,26699698	0,212	0,01	4,24E-23	NFATC3	UPP	M2_vs_M0
1,26E-18	0,26698451	0,143	0,01	4,18E-14	ZNF562	UPP	M2_vs_M0
2,94E-21	0,26662177	0,175	0,01	9,73E-17	SCYL2	UPP	M2_vs_M0
2,30E-21	0,26644306	0,156	0,01	7,60E-17	ZSWIM8	UPP	M2_vs_M0
6,36E-28	0,26636911	0,233	0,02	2,11E-23	MLXIP	UPP	M2_vs_M0
4,36E-17	0,266176	0,167	0,02	1,44E-12	UBALD2	UPP	M2_vs_M0
5,43E-29	0,26608031	0,263	0,03	1,80E-24	SNRNP200	UPP	M2_vs_M0
5,44E-23	0,26577967	0,156	0	1,80E-18	ZNF430	UPP	M2_vs_M0
6,56E-12	0,26561909	0,114	0,01	2,17E-07	GMPS	UPP	M2_vs_M0
3,97E-20	0,26548431	0,138	0	1,31E-15	POM121	UPP	M2_vs_M0
2,37E-27	0,26546558	0,215	0,01	7,86E-23	SMURF2	UPP	M2_vs_M0
1,01E-19	0,26522336	0,141	0,01	3,35E-15	CPNE2	UPP	M2_vs_M0
5,13E-31	0,26518973	0,31	0,04	1,70E-26	GPR137B	UPP	M2_vs_M0
2,40E-09	0,2650592	1	0,95	7,94E-05	HLA-DRA	UPP	M2_vs_M0
1,98E-14	0,26475395	0,119	0,01	6,55E-10	TMEM259	UPP	M2_vs_M0
2,29E-20	0,26470489	0,164	0,01	7,59E-16	SNHG12	UPP	M2_vs_M0

4,92E-25	0,26469099	0,18	0,01	1,63E-20	FAM102B	UPP	M2_vs_M0
2,64E-19	0,26462194	0,175	0,02	8,74E-15	TK2	UPP	M2_vs_M0
1,69E-31	0,26452468	0,313	0,04	5,59E-27	UBL3	UPP	M2_vs_M0
4,48E-28	0,26440834	0,273	0,03	1,49E-23	DHRS7	UPP	M2_vs_M0
2,26E-16	0,26432015	0,133	0,01	7,50E-12	SRD5A3	UPP	M2_vs_M0
1,04E-17	0,26407334	0,127	0,01	3,43E-13	SLC25A46	UPP	M2_vs_M0
5,97E-28	0,26403078	0,281	0,04	1,98E-23	RAD23B	UPP	M2_vs_M0
1,38E-16	0,26400629	0,125	0,01	4,57E-12	TACC3	UPP	M2_vs_M0
1,33E-24	0,26335601	0,172	0,01	4,41E-20	TOR4A	UPP	M2_vs_M0
1,60E-34	0,2632321	0,424	0,07	5,29E-30	ATP6V1A	UPP	M2_vs_M0
3,05E-35	0,26321286	0,385	0,06	1,01E-30	WASHC4	UPP	M2_vs_M0
1,28E-26	0,26307234	0,241	0,02	4,25E-22	FMNL1	UPP	M2_vs_M0
5,26E-13	-0,2633585	0,369	0,13	1,74E-08	POLR2J	DWW	M2_vs_M0
1,31E-06	-0,2638078	0,149	0,05	0,04323	RAB5IF	DWW	M2_vs_M0
9,93E-13	-0,26447164	0,531	0,21	3,29E-08	EIF3L	DWW	M2_vs_M0
5,98E-11	-0,26466612	0,549	0,24	1,98E-06	H2AFZ	DWW	M2_vs_M0
4,18E-12	-0,2646696	0,3	0,1	1,39E-07	EIF3J	DWW	M2_vs_M0
1,26E-10	-0,26541801	0,239	0,08	4,17E-06	PLIN3	DWW	M2_vs_M0
1,66E-08	-0,26642843	0,183	0,06	0,000551	MLX	DWW	M2_vs_M0
1,33E-07	-0,26674172	0,162	0,05	0,004413	MSRB2	DWW	M2_vs_M0
6,65E-18	-0,26692006	0,318	0,08	2,20E-13	SAT2	DWW	M2_vs_M0
1,33E-07	-0,26697225	0,159	0,05	0,004393	FDX1	DWW	M2_vs_M0
1,85E-14	-0,26800964	0,369	0,12	6,14E-10	TUFM	DWW	M2_vs_M0
1,17E-15	-0,26801076	0,387	0,13	3,87E-11	FKBP8	DWW	M2_vs_M0
7,84E-10	-0,2681235	0,194	0,06	2,60E-05	COA5	DWW	M2_vs_M0
1,24E-11	-0,26833652	0,308	0,11	4,11E-07	CCT2	DWW	M2_vs_M0
2,60E-12	-0,26865292	0,472	0,19	8,62E-08	LDHB	DWW	M2_vs_M0
2,03E-12	-0,26872425	0,313	0,11	6,71E-08	NDUFA5	DWW	M2_vs_M0
3,46E-12	-0,26960603	0,239	0,07	1,15E-07	PSMD12	DWW	M2_vs_M0
3,94E-12	-0,26986731	0,313	0,11	1,30E-07	ATP6V1D	DWW	M2_vs_M0
5,37E-10	-0,27014345	0,279	0,1	1,78E-05	CBR1	DWW	M2_vs_M0
4,80E-16	-0,27020056	0,305	0,08	1,59E-11	LACTB	DWW	M2_vs_M0
3,43E-07	-0,27076296	0,18	0,07	0,011371	RAB34	DWW	M2_vs_M0
8,52E-16	-0,27204749	0,406	0,13	2,82E-11	PCBD1	DWW	M2_vs_M0
2,15E-17	-0,27301149	0,332	0,09	7,14E-13	SRSF10	DWW	M2_vs_M0
1,20E-11	-0,27556158	0,292	0,1	3,97E-07	TOMM22	DWW	M2_vs_M0
6,41E-15	-0,27949199	0,297	0,09	2,12E-10	MRPL27	DWW	M2_vs_M0
2,00E-07	-0,28012101	0,21	0,08	0,006619	STOML2	DWW	M2_vs_M0
2,99E-08	-0,28018354	0,183	0,06	0,00099	GYG1	DWW	M2_vs_M0
5,19E-10	-0,28091103	0,223	0,07	1,72E-05	NIFK	DWW	M2_vs_M0
2,36E-10	-0,2811558	0,199	0,06	7,83E-06	PQBP1	DWW	M2_vs_M0
7,93E-11	-0,28148492	0,289	0,1	2,63E-06	FIBP	DWW	M2_vs_M0
1,15E-15	-0,28200935	0,294	0,08	3,82E-11	ERCC1	DWW	M2_vs_M0
1,24E-12	-0,28223949	0,252	0,07	4,12E-08	KYNU	DWW	M2_vs_M0
2,32E-14	-0,28233789	0,462	0,17	7,69E-10	XRCC5	DWW	M2_vs_M0
4,76E-07	-0,28367679	0,133	0,04	0,01576	MRPL42	DWW	M2_vs_M0
4,77E-10	-0,28569643	0,241	0,08	1,58E-05	PDAP1	DWW	M2_vs_M0
1,64E-14	-0,28617233	0,361	0,12	5,44E-10	ATG3	DWW	M2_vs_M0
4,50E-14	-0,28694795	0,292	0,09	1,49E-09	PSMB5	DWW	M2_vs_M0
1,26E-14	-0,28697722	0,39	0,13	4,17E-10	RTF2	DWW	M2_vs_M0

4,24E-14	-0,28842512	0,345	0,11	1,40E-09	UQCRFS1	DWW	M2_vs_M0
5,99E-11	-0,2886048	0,3	0,11	1,98E-06	NAA20	DWW	M2_vs_M0
6,60E-08	-0,28925164	0,162	0,05	0,002187	TXNDC9	DWW	M2_vs_M0
4,10E-14	-0,28934016	0,538	0,21	1,36E-09	ENY2	DWW	M2_vs_M0
9,84E-15	-0,29049991	0,528	0,2	3,26E-10	ANAPC11	DWW	M2_vs_M0
7,92E-10	-0,29156817	0,236	0,08	2,62E-05	POLR2K	DWW	M2_vs_M0
3,91E-14	-0,29180157	0,424	0,15	1,29E-09	SUMO3	DWW	M2_vs_M0
7,36E-14	-0,29197605	0,531	0,21	2,44E-09	SF3B5	DWW	M2_vs_M0
1,33E-11	-0,29269127	0,401	0,16	4,40E-07	NDUFA13	DWW	M2_vs_M0
1,60E-07	-0,29271149	0,202	0,08	0,005307	RAB4A	DWW	M2_vs_M0
1,75E-13	-0,29313511	0,395	0,14	5,78E-09	MRPL52	DWW	M2_vs_M0
1,81E-10	-0,29401749	0,228	0,07	6,00E-06	POLR2G	DWW	M2_vs_M0
5,72E-12	-0,29518697	0,589	0,24	1,90E-07	OSTF1	DWW	M2_vs_M0
2,80E-09	-0,29616822	0,629	0,29	9,26E-05	ATP6V1G1	DWW	M2_vs_M0
3,61E-11	-0,2967345	0,48	0,2	1,19E-06	SNU13	DWW	M2_vs_M0
1,14E-11	-0,29706997	0,231	0,07	3,78E-07	CTNNBL1	DWW	M2_vs_M0
1,47E-13	-0,2972575	0,528	0,21	4,86E-09	EFHD2	DWW	M2_vs_M0
9,51E-14	-0,29729761	0,464	0,17	3,15E-09	PRDX3	DWW	M2_vs_M0
6,52E-11	-0,29746169	0,239	0,08	2,16E-06	SRP19	DWW	M2_vs_M0
9,91E-08	-0,2986265	0,488	0,24	0,003283	RPL36A	DWW	M2_vs_M0
1,62E-08	-0,29912332	0,228	0,08	0,000535	ANXA6	DWW	M2_vs_M0
1,94E-14	-0,299261	0,451	0,16	6,41E-10	DYNLT1	DWW	M2_vs_M0
3,91E-13	-0,29973649	0,3	0,09	1,30E-08	RRP7A	DWW	M2_vs_M0
1,22E-13	-0,30183581	0,294	0,09	4,04E-09	NDUFV1	DWW	M2_vs_M0
2,36E-11	-0,30194929	0,202	0,06	7,83E-07	DMAC1	DWW	M2_vs_M0
1,43E-12	-0,30341946	0,573	0,23	4,72E-08	SOD1	DWW	M2_vs_M0
8,80E-12	-0,30556281	0,528	0,22	2,92E-07	NDUFB7	DWW	M2_vs_M0
6,77E-09	-0,30575204	0,175	0,05	0,000224	BCKDK	DWW	M2_vs_M0
2,98E-07	-0,30625155	0,21	0,08	0,009871	TMEM141	DWW	M2_vs_M0
1,68E-11	-0,30732903	0,18	0,04	5,57E-07	ZCCHC10	DWW	M2_vs_M0
3,81E-14	-0,30780503	0,321	0,1	1,26E-09	TCP1	DWW	M2_vs_M0
3,52E-10	-0,30867199	0,411	0,17	1,17E-05	PSMD8	DWW	M2_vs_M0
3,78E-11	-0,3096654	0,345	0,13	1,25E-06	CALM3	DWW	M2_vs_M0
1,53E-12	-0,30968372	0,302	0,1	5,08E-08	CKLF	DWW	M2_vs_M0
1,89E-08	-0,30981382	0,162	0,05	0,000626	ARHGAP15	DWW	M2_vs_M0
5,90E-08	-0,30999656	0,236	0,09	0,001955	VTI1B	DWW	M2_vs_M0
3,01E-09	-0,31058148	0,432	0,19	9,98E-05	TRAPPC1	DWW	M2_vs_M0
5,04E-08	-0,3117543	0,316	0,14	0,001671	SYF2	DWW	M2_vs_M0
3,18E-09	-0,31178827	0,594	0,27	0,000105	PKM	DWW	M2_vs_M0
4,35E-14	-0,31181117	0,427	0,15	1,44E-09	MRPL51	DWW	M2_vs_M0
3,04E-07	-0,31242415	0,204	0,08	0,010066	ARF5	DWW	M2_vs_M0
2,66E-12	-0,31337083	0,297	0,1	8,82E-08	POLR2I	DWW	M2_vs_M0
1,22E-09	-0,31387563	0,228	0,08	4,03E-05	OGFR	DWW	M2_vs_M0
4,28E-10	-0,31487772	0,263	0,09	1,42E-05	HAX1	DWW	M2_vs_M0
1,71E-12	-0,31527541	0,374	0,14	5,67E-08	SNRPB2	DWW	M2_vs_M0
2,57E-15	-0,31532033	0,488	0,18	8,52E-11	EIF3D	DWW	M2_vs_M0
1,44E-06	-0,31548486	0,231	0,1	0,047844	COMMD8	DWW	M2_vs_M0
2,91E-10	-0,31548839	0,289	0,11	9,63E-06	PDCD6	DWW	M2_vs_M0
3,56E-16	-0,31729415	0,408	0,13	1,18E-11	CACYBP	DWW	M2_vs_M0
1,90E-09	-0,31793996	0,196	0,06	6,30E-05	MIEN1	DWW	M2_vs_M0

3,81E-09	-0,31874721	0,592	0,27	0,000126	HMGN2	DWW	M2_vs_M0
5,91E-09	-0,31953944	0,194	0,06	0,000196	DCTN6	DWW	M2_vs_M0
1,71E-08	-0,31972429	0,207	0,07	0,000567	CCNDBP1	DWW	M2_vs_M0
3,74E-15	-0,3200729	0,488	0,18	1,24E-10	HMGN3	DWW	M2_vs_M0
1,65E-07	-0,32029912	0,143	0,04	0,005467	LZIC	DWW	M2_vs_M0
2,52E-09	-0,32041893	0,475	0,21	8,34E-05	TKT	DWW	M2_vs_M0
3,69E-10	-0,32080338	0,257	0,09	1,22E-05	AIP	DWW	M2_vs_M0
7,53E-10	-0,32153789	0,515	0,23	2,49E-05	LGALS9	DWW	M2_vs_M0
2,45E-13	-0,32225994	0,358	0,12	8,12E-09	PSMD7	DWW	M2_vs_M0
1,06E-09	-0,3240881	0,252	0,09	3,50E-05	POLE4	DWW	M2_vs_M0
7,79E-14	-0,32417317	0,475	0,18	2,58E-09	CASP1	DWW	M2_vs_M0
9,37E-12	-0,32463075	0,56	0,23	3,10E-07	BRK1	DWW	M2_vs_M0
9,94E-14	-0,32524682	0,305	0,09	3,29E-09	ETFA	DWW	M2_vs_M0
1,92E-08	-0,32528753	0,281	0,11	0,000635	MRPL40	DWW	M2_vs_M0
3,86E-09	-0,32622377	0,244	0,09	0,000128	TIMM10	DWW	M2_vs_M0
1,03E-06	-0,32664117	0,18	0,07	0,033999	ZSCAN16-A	DWW	M2_vs_M0
1,56E-12	-0,32707439	0,313	0,11	5,17E-08	ISG15	DWW	M2_vs_M0
3,25E-08	-0,32777086	0,239	0,09	0,001078	NDUFS3	DWW	M2_vs_M0
1,40E-13	-0,3283817	0,462	0,18	4,63E-09	ROMO1	DWW	M2_vs_M0
3,23E-11	-0,32910065	0,26	0,09	1,07E-06	FRG1	DWW	M2_vs_M0
7,02E-07	-0,32977718	0,191	0,07	0,023247	NUTF2	DWW	M2_vs_M0
1,83E-13	-0,32983854	0,366	0,13	6,07E-09	CHMP4A	DWW	M2_vs_M0
1,09E-12	-0,33034648	0,382	0,14	3,62E-08	PSMB7	DWW	M2_vs_M0
3,02E-08	-0,33048193	0,204	0,07	0,001001	sep-06	DWW	M2_vs_M0
9,33E-15	-0,33064051	0,215	0,05	3,09E-10	ACBD5	DWW	M2_vs_M0
1,15E-08	-0,33138875	0,324	0,14	0,000382	BCL2A1	DWW	M2_vs_M0
2,10E-10	-0,3315836	0,305	0,11	6,96E-06	DECR1	DWW	M2_vs_M0
1,70E-10	-0,33193955	0,228	0,07	5,63E-06	CDC26	DWW	M2_vs_M0
1,17E-10	-0,33252865	0,202	0,06	3,89E-06	PDHA1	DWW	M2_vs_M0
4,81E-08	-0,33272351	0,194	0,07	0,001595	DGUOK	DWW	M2_vs_M0
4,68E-08	-0,33298445	0,199	0,07	0,001552	APBB1IP	DWW	M2_vs_M0
1,66E-11	-0,33644306	0,34	0,12	5,52E-07	EIF4E2	DWW	M2_vs_M0
2,33E-15	-0,33668692	0,281	0,08	7,72E-11	ACP1	DWW	M2_vs_M0
1,07E-17	-0,3369766	0,377	0,11	3,56E-13	C1orf43	DWW	M2_vs_M0
3,51E-08	-0,33807478	0,188	0,07	0,001161	MRPL3	DWW	M2_vs_M0
1,67E-10	-0,33814408	0,403	0,16	5,53E-06	ETFB	DWW	M2_vs_M0
6,63E-14	-0,33841737	0,448	0,16	2,20E-09	SSB	DWW	M2_vs_M0
1,12E-10	-0,33860295	0,292	0,1	3,70E-06	MRPL14	DWW	M2_vs_M0
6,30E-07	-0,33892225	0,18	0,07	0,020865	MRPL15	DWW	M2_vs_M0
3,51E-08	-0,33921363	0,21	0,08	0,001161	PSMB10	DWW	M2_vs_M0
2,35E-12	-0,34135643	0,324	0,11	7,79E-08	PHB	DWW	M2_vs_M0
5,82E-14	-0,34186636	0,408	0,14	1,93E-09	MDH1	DWW	M2_vs_M0
1,07E-08	-0,34377741	0,212	0,07	0,000356	CYC1	DWW	M2_vs_M0
4,94E-08	-0,34459219	0,241	0,09	0,001636	THOC7	DWW	M2_vs_M0
1,08E-11	-0,3452079	0,57	0,24	3,58E-07	HSBP1	DWW	M2_vs_M0
3,64E-07	-0,34526492	0,334	0,15	0,012059	DOK2	DWW	M2_vs_M0
2,66E-11	-0,34930431	0,552	0,23	8,81E-07	FKBP1A	DWW	M2_vs_M0
1,04E-15	-0,35031308	0,408	0,13	3,45E-11	ENSA	DWW	M2_vs_M0
3,46E-12	-0,35076415	0,424	0,16	1,15E-07	BANF1	DWW	M2_vs_M0
6,27E-11	-0,35089289	0,361	0,14	2,08E-06	TMEM230	DWW	M2_vs_M0

1,05E-07	-0,35152157	0,382	0,18	0,00349	RPS4Y1	DWW	M2_vs_M0
2,70E-07	-0,353164	0,202	0,08	0,008933	ZBTB8OS	DWW	M2_vs_M0
6,57E-14	-0,35336897	0,406	0,14	2,18E-09	NDUFS8	DWW	M2_vs_M0
1,15E-09	-0,3543884	0,223	0,07	3,82E-05	PYURF	DWW	M2_vs_M0
2,14E-10	-0,35468963	0,403	0,17	7,08E-06	H2AFJ	DWW	M2_vs_M0
2,48E-07	-0,35544896	0,3	0,13	0,008211	ISCU	DWW	M2_vs_M0
1,30E-07	-0,35665213	0,225	0,09	0,004309	GSDMD	DWW	M2_vs_M0
1,18E-10	-0,35677565	0,199	0,06	3,91E-06	MEA1	DWW	M2_vs_M0
1,33E-12	-0,35726577	0,316	0,11	4,41E-08	SNX17	DWW	M2_vs_M0
5,98E-12	-0,35857223	0,385	0,14	1,98E-07	EIF3I	DWW	M2_vs_M0
1,76E-09	-0,35868065	0,223	0,08	5,83E-05	MT1X	DWW	M2_vs_M0
6,70E-09	-0,35949796	0,271	0,1	0,000222	MRPS15	DWW	M2_vs_M0
1,23E-11	-0,36019908	0,379	0,14	4,09E-07	NAA38	DWW	M2_vs_M0
1,67E-07	-0,360363	0,207	0,08	0,005546	NUBP1	DWW	M2_vs_M0
5,81E-15	-0,36262266	0,438	0,15	1,92E-10	VDAC2	DWW	M2_vs_M0
1,01E-09	-0,36318835	0,347	0,14	3,35E-05	HLA-F	DWW	M2_vs_M0
5,81E-13	-0,36323891	0,268	0,08	1,92E-08	PFDN1	DWW	M2_vs_M0
4,05E-13	-0,36503902	0,35	0,12	1,34E-08	TMEM256	DWW	M2_vs_M0
2,64E-10	-0,36519249	0,361	0,14	8,76E-06	UBE2I	DWW	M2_vs_M0
3,02E-10	-0,3655519	0,284	0,1	9,99E-06	GADD45GI	DWW	M2_vs_M0
1,55E-11	-0,36609291	0,26	0,08	5,13E-07	CHCHD5	DWW	M2_vs_M0
6,75E-12	-0,36631702	0,289	0,1	2,24E-07	ZCRB1	DWW	M2_vs_M0
9,29E-14	-0,36743972	0,456	0,17	3,08E-09	NDUFB5	DWW	M2_vs_M0
5,37E-11	-0,36756481	0,523	0,22	1,78E-06	NDUFA2	DWW	M2_vs_M0
4,20E-13	-0,36844345	0,302	0,1	1,39E-08	NDUFB9	DWW	M2_vs_M0
9,37E-07	-0,36877174	0,31	0,14	0,031043	MPC1	DWW	M2_vs_M0
3,20E-10	-0,37077235	0,316	0,12	1,06E-05	LSM10	DWW	M2_vs_M0
1,95E-08	-0,37116446	0,183	0,06	0,000646	SS18L2	DWW	M2_vs_M0
1,21E-09	-0,37135452	0,276	0,1	4,01E-05	SUCLG1	DWW	M2_vs_M0
1,36E-10	-0,3724267	0,395	0,16	4,51E-06	CASP4	DWW	M2_vs_M0
2,38E-07	-0,37298556	0,194	0,07	0,007894	HIKESHI	DWW	M2_vs_M0
8,61E-12	-0,37350479	0,369	0,14	2,85E-07	EPSTI1	DWW	M2_vs_M0
1,01E-11	-0,37454029	0,422	0,16	3,35E-07	C19orf70	DWW	M2_vs_M0
3,96E-11	-0,37599406	0,369	0,14	1,31E-06	IL18	DWW	M2_vs_M0
2,24E-12	-0,37659267	0,451	0,18	7,43E-08	MRPL20	DWW	M2_vs_M0
8,99E-11	-0,37691634	0,329	0,12	2,98E-06	ZNHIT1	DWW	M2_vs_M0
9,41E-09	-0,37714949	0,634	0,29	0,000312	HIGD2A	DWW	M2_vs_M0
4,38E-12	-0,37913478	0,374	0,14	1,45E-07	SNRPD1	DWW	M2_vs_M0
3,75E-11	-0,3791952	0,263	0,09	1,24E-06	DPY30	DWW	M2_vs_M0
2,35E-11	-0,37994808	0,578	0,24	7,80E-07	LAMTOR1	DWW	M2_vs_M0
1,19E-08	-0,38019498	0,249	0,09	0,000394	TPMT	DWW	M2_vs_M0
4,07E-14	-0,38121118	0,284	0,08	1,35E-09	DNAJA2	DWW	M2_vs_M0
7,11E-12	-0,3822057	0,347	0,13	2,35E-07	DNAJC15	DWW	M2_vs_M0
3,18E-09	-0,38378313	0,257	0,09	0,000105	MPC2	DWW	M2_vs_M0
9,26E-11	-0,38444049	0,464	0,19	3,07E-06	ATP5PB	DWW	M2_vs_M0
5,54E-07	-0,38604776	0,215	0,09	0,018352	IFT20	DWW	M2_vs_M0
1,50E-10	-0,38704173	0,594	0,26	4,97E-06	ATP5PD	DWW	M2_vs_M0
1,03E-15	-0,38760157	0,414	0,14	3,41E-11	TOP1	DWW	M2_vs_M0
1,53E-08	-0,3881005	0,544	0,25	0,000506	NEDD8	DWW	M2_vs_M0
2,49E-11	-0,3902741	0,454	0,18	8,26E-07	C1orf162	DWW	M2_vs_M0

1,24E-09	-0,3902996	0,623	0,27	4,10E-05	SAP18	DWW	M2_vs_M0
3,82E-13	-0,39067476	0,48	0,18	1,27E-08	TOMM20	DWW	M2_vs_M0
3,82E-09	-0,39191749	0,22	0,08	0,000127	DUT	DWW	M2_vs_M0
4,38E-11	-0,39226512	0,512	0,21	1,45E-06	VPS28	DWW	M2_vs_M0
3,01E-11	-0,3938152	0,332	0,12	9,97E-07	TXN2	DWW	M2_vs_M0
7,07E-14	-0,39394816	0,358	0,12	2,34E-09	SUPT4H1	DWW	M2_vs_M0
2,20E-08	-0,3943403	0,241	0,09	0,000728	MRPL21	DWW	M2_vs_M0
4,50E-09	-0,39493142	0,223	0,08	0,000149	IAH1	DWW	M2_vs_M0
1,08E-07	-0,39547298	0,212	0,08	0,003592	WASHC3	DWW	M2_vs_M0
9,65E-13	-0,39584909	0,586	0,23	3,20E-08	SEM1	DWW	M2_vs_M0
2,85E-11	-0,39634037	0,544	0,23	9,44E-07	NAGK	DWW	M2_vs_M0
6,25E-11	-0,39877487	0,615	0,26	2,07E-06	C4orf3	DWW	M2_vs_M0
7,19E-11	-0,39899893	0,363	0,14	2,38E-06	GABARAPL	DWW	M2_vs_M0
3,91E-08	-0,4026226	0,613	0,29	0,001294	NDUFS5	DWW	M2_vs_M0
5,08E-09	-0,403155	0,414	0,18	0,000168	EIF1B	DWW	M2_vs_M0
9,83E-11	-0,40550859	0,393	0,16	3,26E-06	NCF1	DWW	M2_vs_M0
1,00E-12	-0,40684593	0,419	0,16	3,31E-08	CCT3	DWW	M2_vs_M0
1,19E-09	-0,40688114	0,247	0,09	3,93E-05	PPP1R7	DWW	M2_vs_M0
2,16E-11	-0,40956392	0,475	0,19	7,15E-07	CHMP2A	DWW	M2_vs_M0
2,46E-11	-0,41146445	0,406	0,16	8,13E-07	ANP32A	DWW	M2_vs_M0
1,09E-10	-0,41180318	0,308	0,11	3,62E-06	EIF6	DWW	M2_vs_M0
1,35E-07	-0,41182561	0,557	0,27	0,004459	S100A10	DWW	M2_vs_M0
6,57E-13	-0,4128946	0,34	0,12	2,17E-08	PSMD4	DWW	M2_vs_M0
2,80E-12	-0,41293562	0,467	0,18	9,27E-08	RBM8A	DWW	M2_vs_M0
3,97E-09	-0,41319357	0,615	0,28	0,000131	TALDO1	DWW	M2_vs_M0
7,47E-10	-0,41475081	0,289	0,11	2,47E-05	NDUFAF3	DWW	M2_vs_M0
1,84E-08	-0,41499792	0,305	0,13	0,00061	ABI3	DWW	M2_vs_M0
9,57E-08	-0,41598828	0,629	0,3	0,00317	UQCRQ	DWW	M2_vs_M0
1,33E-09	-0,41645007	0,268	0,1	4,41E-05	CPPED1	DWW	M2_vs_M0
2,17E-07	-0,41676993	0,225	0,09	0,007202	SMIM20	DWW	M2_vs_M0
1,69E-08	-0,41711966	0,393	0,17	0,00056	LSM3	DWW	M2_vs_M0
1,36E-09	-0,41913502	0,3	0,12	4,50E-05	BLOC1S2	DWW	M2_vs_M0
2,99E-12	-0,4194761	0,297	0,1	9,90E-08	LSM8	DWW	M2_vs_M0
1,38E-08	-0,4199425	0,225	0,08	0,000458	GLO1	DWW	M2_vs_M0
1,11E-13	-0,42004187	0,467	0,17	3,69E-09	ABRACL	DWW	M2_vs_M0
9,55E-11	-0,42068455	0,34	0,13	3,16E-06	GTF2H5	DWW	M2_vs_M0
6,96E-11	-0,42078362	0,377	0,15	2,31E-06	NDUFS7	DWW	M2_vs_M0
4,41E-08	-0,42182801	0,3	0,13	0,001459	DNPH1	DWW	M2_vs_M0
4,95E-13	-0,42219875	0,379	0,14	1,64E-08	ALDH2	DWW	M2_vs_M0
4,20E-08	-0,4230751	0,958	0,88	0,001391	PTMA	DWW	M2_vs_M0
1,59E-09	-0,42333226	0,212	0,07	5,26E-05	FAM32A	DWW	M2_vs_M0
5,37E-07	-0,4235358	0,164	0,06	0,017805	GMPR2	DWW	M2_vs_M0
1,68E-12	-0,42441167	0,414	0,15	5,56E-08	sep-07	DWW	M2_vs_M0
2,11E-08	-0,42464407	0,265	0,1	0,000698	COA6	DWW	M2_vs_M0
4,98E-10	-0,42480318	0,584	0,26	1,65E-05	TMEM258	DWW	M2_vs_M0
8,61E-10	-0,4250866	0,345	0,14	2,85E-05	NARS	DWW	M2_vs_M0
2,89E-11	-0,42690534	0,477	0,19	9,59E-07	COX14	DWW	M2_vs_M0
7,64E-07	-0,42742631	0,151	0,05	0,025323	CCDC25	DWW	M2_vs_M0
2,24E-12	-0,42769528	0,353	0,12	7,40E-08	RAB8A	DWW	M2_vs_M0
3,96E-11	-0,43030852	0,276	0,09	1,31E-06	CYSTM1	DWW	M2_vs_M0

1,77E-14	-0,43035903	0,398	0,14	5,85E-10	RNF7	DWW	M2_vs_M0
4,47E-07	-0,43037402	0,202	0,08	0,014796	HSPB11	DWW	M2_vs_M0
9,31E-14	-0,43060202	0,446	0,16	3,08E-09	DNAJC8	DWW	M2_vs_M0
1,87E-11	-0,43098317	0,61	0,26	6,21E-07	PTGES3	DWW	M2_vs_M0
8,50E-11	-0,4314202	0,284	0,1	2,81E-06	DCTN3	DWW	M2_vs_M0
5,24E-08	-0,4334129	0,427	0,2	0,001736	PSMA1	DWW	M2_vs_M0
5,69E-12	-0,43611675	0,557	0,23	1,88E-07	EIF3H	DWW	M2_vs_M0
6,00E-07	-0,4368158	0,626	0,31	0,019871	SKP1	DWW	M2_vs_M0
1,22E-09	-0,43683666	0,39	0,16	4,03E-05	SNRPD3	DWW	M2_vs_M0
7,81E-07	-0,43765107	0,676	0,32	0,025877	COX7A2	DWW	M2_vs_M0
4,60E-10	-0,43832016	0,255	0,09	1,52E-05	UFD1	DWW	M2_vs_M0
8,61E-09	-0,43851509	0,406	0,18	0,000285	TRIR	DWW	M2_vs_M0
5,12E-11	-0,43859279	0,369	0,14	1,70E-06	SUMO1	DWW	M2_vs_M0
1,20E-08	-0,43870938	0,44	0,2	0,000396	FAM96B	DWW	M2_vs_M0
2,83E-08	-0,43912241	0,302	0,13	0,000937	BUD31	DWW	M2_vs_M0
8,51E-08	-0,4392475	0,265	0,11	0,00282	MRPL34	DWW	M2_vs_M0
4,20E-12	-0,44055863	0,501	0,2	1,39E-07	GPSM3	DWW	M2_vs_M0
2,89E-08	-0,44271113	0,997	0,99	0,000957	FTL	DWW	M2_vs_M0
3,73E-11	-0,44417999	0,337	0,12	1,24E-06	GTF2A2	DWW	M2_vs_M0
2,77E-12	-0,44547133	0,398	0,15	9,18E-08	NANS	DWW	M2_vs_M0
1,09E-09	-0,44651172	0,477	0,21	3,63E-05	TMEM14C	DWW	M2_vs_M0
1,13E-07	-0,4465461	0,212	0,08	0,003746	TRAPPC3	DWW	M2_vs_M0
6,19E-07	-0,44700229	0,207	0,08	0,020519	DESI1	DWW	M2_vs_M0
2,00E-09	-0,44799798	0,454	0,2	6,64E-05	SSBP1	DWW	M2_vs_M0
3,32E-08	-0,45019793	0,613	0,29	0,001101	AKR1A1	DWW	M2_vs_M0
6,10E-15	-0,45031461	0,39	0,13	2,02E-10	NUDC	DWW	M2_vs_M0
5,44E-08	-0,45130206	0,3	0,13	0,001802	PRMT1	DWW	M2_vs_M0
2,82E-08	-0,4535693	0,289	0,12	0,000934	FAM162A	DWW	M2_vs_M0
2,68E-10	-0,45398079	0,406	0,17	8,86E-06	EIF3F	DWW	M2_vs_M0
8,29E-09	-0,45559461	0,22	0,08	0,000275	FAM204A	DWW	M2_vs_M0
9,90E-09	-0,45668269	0,345	0,15	0,000328	UBE2L6	DWW	M2_vs_M0
1,55E-14	-0,45782163	0,366	0,12	5,14E-10	MT-ND6	DWW	M2_vs_M0
1,01E-06	-0,45928253	0,241	0,1	0,033455	CAMTA1	DWW	M2_vs_M0
4,43E-07	-0,46031027	0,223	0,09	0,014663	PSMC3	DWW	M2_vs_M0
1,25E-07	-0,46077455	0,252	0,1	0,004128	NMI	DWW	M2_vs_M0
4,78E-08	-0,46204964	0,223	0,08	0,001584	SARS	DWW	M2_vs_M0
2,72E-11	-0,46280631	0,374	0,14	9,01E-07	NCF2	DWW	M2_vs_M0
2,82E-11	-0,46322913	0,31	0,11	9,33E-07	TFPT	DWW	M2_vs_M0
6,13E-11	-0,46414083	0,305	0,11	2,03E-06	CLNS1A	DWW	M2_vs_M0
5,32E-08	-0,46665061	0,408	0,18	0,001763	GSTK1	DWW	M2_vs_M0
2,97E-07	-0,46773975	0,321	0,14	0,009849	IDH3G	DWW	M2_vs_M0
1,22E-08	-0,47039955	0,21	0,07	0,000403	C12orf45	DWW	M2_vs_M0
4,51E-08	-0,47299468	0,546	0,25	0,001493	NDUFA3	DWW	M2_vs_M0
3,19E-09	-0,47368562	0,446	0,19	0,000106	NDUFA11	DWW	M2_vs_M0
3,84E-10	-0,4740175	0,552	0,24	1,27E-05	MGST3	DWW	M2_vs_M0
1,76E-08	-0,47490731	0,992	0,92	0,000584	HLA-DPB1	DWW	M2_vs_M0
1,27E-07	-0,47510666	0,592	0,28	0,004219	SEC11A	DWW	M2_vs_M0
1,86E-11	-0,47887703	0,355	0,13	6,18E-07	APEX1	DWW	M2_vs_M0
1,46E-09	-0,47919601	0,443	0,19	4,84E-05	ERH	DWW	M2_vs_M0
2,81E-10	-0,47959786	0,371	0,15	9,29E-06	SRP9	DWW	M2_vs_M0

2,22E-11	-0,48219332	0,332	0,12	7,34E-07	NBDY	DWW	M2_vs_M0
1,24E-07	-0,4838882	0,589	0,28	0,004102	JPT1	DWW	M2_vs_M0
1,46E-06	-0,48748479	0,244	0,11	0,048402	AHSA1	DWW	M2_vs_M0
1,43E-10	-0,4881404	0,48	0,2	4,72E-06	SELENOH	DWW	M2_vs_M0
1,46E-11	-0,48868834	0,454	0,18	4,82E-07	UXT	DWW	M2_vs_M0
2,18E-10	-0,48989505	0,355	0,14	7,23E-06	UQCRC2	DWW	M2_vs_M0
9,40E-09	-0,49062617	0,363	0,15	0,000311	IFI27L2	DWW	M2_vs_M0
2,00E-09	-0,49132006	0,337	0,14	6,63E-05	MRPL57	DWW	M2_vs_M0
6,17E-09	-0,49145851	0,462	0,2	0,000204	SNRPG	DWW	M2_vs_M0
2,45E-11	-0,49225668	0,507	0,21	8,12E-07	UBE2L3	DWW	M2_vs_M0
6,23E-09	-0,49291784	0,239	0,09	0,000206	CIR1	DWW	M2_vs_M0
7,95E-08	-0,49308426	0,424	0,19	0,002635	SNRPB	DWW	M2_vs_M0
1,30E-07	-0,49527306	0,244	0,1	0,0043	MAP3K13	DWW	M2_vs_M0
1,05E-06	-0,49728159	0,215	0,09	0,034785	HSD17B10	DWW	M2_vs_M0
6,52E-08	-0,49883724	0,361	0,16	0,002161	SNRPF	DWW	M2_vs_M0
1,90E-07	-0,50006258	0,241	0,1	0,006295	IGBP1	DWW	M2_vs_M0
7,12E-24	-0,50206548	0,997	0,99	2,36E-19	TPT1	DWW	M2_vs_M0
6,28E-10	-0,50317594	0,382	0,15	2,08E-05	BAX	DWW	M2_vs_M0
9,79E-10	-0,50589564	0,35	0,14	3,24E-05	PHB2	DWW	M2_vs_M0
7,35E-09	-0,50615942	0,297	0,12	0,000244	LSM5	DWW	M2_vs_M0
1,59E-07	-0,51090826	0,416	0,19	0,005283	MNDA	DWW	M2_vs_M0
1,22E-07	-0,51122045	0,212	0,08	0,00403	EIF2A	DWW	M2_vs_M0
8,54E-08	-0,51356113	0,499	0,23	0,00283	NDUFB8	DWW	M2_vs_M0
6,55E-07	-0,51451438	0,653	0,31	0,021697	SH3BGRL	DWW	M2_vs_M0
1,29E-11	-0,51697846	0,393	0,15	4,28E-07	MRPS21	DWW	M2_vs_M0
1,84E-11	-0,51742834	0,456	0,18	6,11E-07	DYNLRB1	DWW	M2_vs_M0
6,54E-07	-0,51844979	0,509	0,25	0,021679	IFITM3	DWW	M2_vs_M0
5,44E-12	-0,51915044	0,318	0,11	1,80E-07	CCT7	DWW	M2_vs_M0
8,14E-08	-0,52100588	0,385	0,17	0,002695	C8orf59	DWW	M2_vs_M0
7,16E-10	-0,52342329	0,332	0,13	2,37E-05	SPAG7	DWW	M2_vs_M0
1,01E-06	-0,52683954	0,326	0,15	0,03333	SDHD	DWW	M2_vs_M0
9,43E-09	-0,53328021	0,401	0,17	0,000312	TXNDC17	DWW	M2_vs_M0
9,89E-10	-0,53448887	0,509	0,22	3,28E-05	GUK1	DWW	M2_vs_M0
1,11E-06	-0,53745686	0,43	0,21	0,036682	VPS29	DWW	M2_vs_M0
1,30E-11	-0,53949914	0,369	0,14	4,30E-07	ELOC	DWW	M2_vs_M0
5,02E-07	-0,54156485	0,329	0,15	0,016639	SDHB	DWW	M2_vs_M0
3,49E-09	-0,5418401	0,353	0,15	0,000116	CHMP5	DWW	M2_vs_M0
1,58E-08	-0,54378807	0,347	0,15	0,000523	POLR1D	DWW	M2_vs_M0
4,12E-13	-0,54812336	0,952	0,85	1,36E-08	OAZ1	DWW	M2_vs_M0
1,29E-09	-0,55048287	0,345	0,14	4,27E-05	BTF3L4	DWW	M2_vs_M0
2,95E-07	-0,5513561	0,39	0,18	0,009758	RNF181	DWW	M2_vs_M0
5,73E-11	-0,55516289	0,398	0,16	1,90E-06	SLIRP	DWW	M2_vs_M0
2,84E-07	-0,55683787	0,576	0,28	0,009407	BLOC1S1	DWW	M2_vs_M0
4,42E-08	-0,5601074	0,268	0,11	0,001464	NDUFS4	DWW	M2_vs_M0
8,08E-08	-0,56209726	0,517	0,24	0,002677	ATP5PO	DWW	M2_vs_M0
6,84E-08	-0,56286912	0,422	0,19	0,002267	GTF3C6	DWW	M2_vs_M0
9,72E-08	-0,56485862	0,358	0,16	0,00322	TSPO	DWW	M2_vs_M0
8,10E-07	-0,56654709	0,305	0,14	0,026843	ALKBH7	DWW	M2_vs_M0
6,77E-07	-0,57165537	0,321	0,15	0,022441	SELENOW	DWW	M2_vs_M0
1,20E-08	-0,57329716	0,382	0,16	0,000396	CCT8	DWW	M2_vs_M0

1,12E-08	-0,57762732	0,504	0,23	0,00037	CIB1	DWW	M2_vs_M0
3,82E-10	-0,58124825	0,342	0,13	1,27E-05	IDH2	DWW	M2_vs_M0
7,29E-07	-0,58237416	0,586	0,28	0,024146	ZFAS1	DWW	M2_vs_M0
1,96E-08	-0,5867453	0,401	0,18	0,00065	MRPL33	DWW	M2_vs_M0
2,52E-10	-0,59095671	0,276	0,1	8,33E-06	COA4	DWW	M2_vs_M0
8,07E-07	-0,59277744	0,305	0,14	0,026733	ECH1	DWW	M2_vs_M0
3,64E-10	-0,59397128	0,345	0,13	1,21E-05	RSL24D1	DWW	M2_vs_M0
7,22E-07	-0,59583904	0,523	0,25	0,023901	PRDX5	DWW	M2_vs_M0
3,97E-08	-0,59666913	0,395	0,18	0,001314	EIF5A	DWW	M2_vs_M0
8,83E-08	-0,59769405	0,565	0,26	0,002926	EIF3E	DWW	M2_vs_M0
6,47E-07	-0,60840498	0,432	0,2	0,021446	PFDN2	DWW	M2_vs_M0
1,23E-07	-0,61140843	0,3	0,13	0,004089	PAK1	DWW	M2_vs_M0
1,63E-08	-0,61364694	0,454	0,2	0,00054	NDUFC1	DWW	M2_vs_M0
1,17E-07	-0,61458927	0,377	0,17	0,003876	COX7A2L	DWW	M2_vs_M0
2,75E-23	-0,61740287	0,989	0,96	9,12E-19	EEF1A1	DWW	M2_vs_M0
3,06E-21	-0,61966078	0,971	0,95	1,01E-16	RPS28	DWW	M2_vs_M0
1,16E-16	-0,61973004	0,944	0,85	3,86E-12	RPLP2	DWW	M2_vs_M0
4,78E-07	-0,62594756	0,265	0,11	0,01582	MRPS36	DWW	M2_vs_M0
2,01E-07	-0,62699938	0,302	0,13	0,006653	PSMG2	DWW	M2_vs_M0
2,30E-10	-0,63145921	0,501	0,21	7,62E-06	TRMT112	DWW	M2_vs_M0
8,32E-07	-0,64335527	0,451	0,21	0,02756	ZNF706	DWW	M2_vs_M0
1,44E-07	-0,64340651	0,443	0,21	0,004763	TIMM8B	DWW	M2_vs_M0
6,54E-14	-0,64533726	0,968	0,84	2,17E-09	AIF1	DWW	M2_vs_M0
1,02E-07	-0,6567184	0,395	0,18	0,003367	NDUFA6	DWW	M2_vs_M0
6,01E-08	-0,66200104	0,44	0,2	0,001992	SNRPD2	DWW	M2_vs_M0
8,35E-45	-0,66580828	0,997	0,99	2,77E-40	TMSB4X	DWW	M2_vs_M0
8,77E-18	-0,67158803	0,928	0,84	2,91E-13	SERF2	DWW	M2_vs_M0
2,63E-18	-0,68173098	0,96	0,88	8,70E-14	RPL21	DWW	M2_vs_M0
4,22E-07	-0,68810211	0,424	0,2	0,013994	SNHG8	DWW	M2_vs_M0
7,57E-20	-0,68843822	0,96	0,88	2,51E-15	ATP5F1E	DWW	M2_vs_M0
1,78E-08	-0,70068876	0,382	0,16	0,00059	SMIM26	DWW	M2_vs_M0
3,69E-20	-0,72319475	0,934	0,84	1,22E-15	RPL13A	DWW	M2_vs_M0
5,49E-09	-0,74736992	0,849	0,69	0,000182	RPLP0	DWW	M2_vs_M0
2,04E-11	-0,75269282	0,899	0,72	6,76E-07	RPL27	DWW	M2_vs_M0
7,69E-12	-0,75854994	0,915	0,79	2,55E-07	S100A11	DWW	M2_vs_M0
4,29E-12	-0,7595008	0,883	0,71	1,42E-07	PFDN5	DWW	M2_vs_M0
1,34E-22	-0,76151005	0,934	0,86	4,44E-18	RPL9	DWW	M2_vs_M0
2,48E-21	-0,76487726	0,907	0,84	8,20E-17	RPS16	DWW	M2_vs_M0
3,09E-23	-0,76516164	0,952	0,89	1,02E-18	RPS13	DWW	M2_vs_M0
7,07E-08	-0,76542201	0,475	0,22	0,002341	COX17	DWW	M2_vs_M0
1,08E-21	-0,78873518	0,95	0,87	3,59E-17	RPS6	DWW	M2_vs_M0
7,66E-13	-0,79837042	0,422	0,17	2,54E-08	S100A9	DWW	M2_vs_M0
1,59E-24	-0,80017296	0,955	0,92	5,28E-20	RPL12	DWW	M2_vs_M0
6,57E-11	-0,82209601	0,369	0,15	2,17E-06	JCHAIN	DWW	M2_vs_M0
6,21E-25	-0,82353218	0,912	0,83	2,06E-20	FAU	DWW	M2_vs_M0
6,51E-49	-0,82769488	0,995	0,99	2,16E-44	RPLP1	DWW	M2_vs_M0
4,58E-30	-0,83479142	0,96	0,94	1,52E-25	RPL39	DWW	M2_vs_M0
1,70E-16	-0,8518916	0,894	0,76	5,62E-12	RPL38	DWW	M2_vs_M0
5,15E-24	-0,86315788	0,939	0,83	1,71E-19	UBA52	DWW	M2_vs_M0
1,72E-24	-0,87419263	0,907	0,84	5,71E-20	RPL35	DWW	M2_vs_M0

1,08E-23	-0,88800081	0,928	0,82	3,59E-19	RPS11	DWW	M2_vs_M0
5,15E-18	-0,89278886	0,897	0,76	1,71E-13	RPL22	DWW	M2_vs_M0
2,55E-29	-0,89601953	0,947	0,9	8,46E-25	RPL34	DWW	M2_vs_M0
6,90E-14	-0,90886687	0,841	0,7	2,28E-09	RPL24	DWW	M2_vs_M0
6,88E-26	-0,91382067	0,923	0,84	2,28E-21	RPL36	DWW	M2_vs_M0
1,35E-13	-0,91823762	0,873	0,72	4,47E-09	RPS21	DWW	M2_vs_M0
8,21E-09	-0,9299788	0,809	0,64	0,000272	RPSA	DWW	M2_vs_M0
3,37E-27	-0,93023423	0,936	0,89	1,12E-22	RPS8	DWW	M2_vs_M0
8,86E-37	-0,9334099	0,966	0,92	2,94E-32	RPS27A	DWW	M2_vs_M0
5,52E-46	-0,93569797	0,981	0,98	1,83E-41	RPL41	DWW	M2_vs_M0
1,43E-32	-0,94438046	0,958	0,93	4,73E-28	RPS12	DWW	M2_vs_M0
6,89E-15	-0,94486715	0,878	0,75	2,28E-10	ACTG1	DWW	M2_vs_M0
2,74E-31	-0,96835859	0,952	0,89	9,07E-27	RPS9	DWW	M2_vs_M0
5,98E-25	-0,98048719	0,897	0,8	1,98E-20	RPS25	DWW	M2_vs_M0
4,37E-31	-0,98133156	0,92	0,86	1,45E-26	RPS15A	DWW	M2_vs_M0
7,70E-19	-0,98505972	0,841	0,72	2,55E-14	ARPC3	DWW	M2_vs_M0
1,89E-18	-1,00508278	0,854	0,73	6,26E-14	RACK1	DWW	M2_vs_M0
4,42E-45	-1,01183032	0,976	0,93	1,46E-40	RPL28	DWW	M2_vs_M0
2,82E-11	-1,01236361	0,804	0,65	9,33E-07	GSTP1	DWW	M2_vs_M0
3,36E-26	-1,01676099	0,875	0,79	1,11E-21	RPL6	DWW	M2_vs_M0
1,15E-29	-1,02484891	0,942	0,87	3,82E-25	RPL27A	DWW	M2_vs_M0
3,65E-23	-1,02747494	0,912	0,81	1,21E-18	RPL35A	DWW	M2_vs_M0
1,20E-40	-1,03195696	0,926	0,89	3,99E-36	RPL15	DWW	M2_vs_M0
6,58E-35	-1,03771647	0,955	0,92	2,18E-30	RPS24	DWW	M2_vs_M0
2,64E-35	-1,03884552	0,944	0,9	8,73E-31	RPS3A	DWW	M2_vs_M0
6,38E-07	-1,04396687	0,798	0,62	0,021137	SH3BGRL3	DWW	M2_vs_M0
2,76E-13	-1,05019424	0,814	0,67	9,13E-09	EEF1B2	DWW	M2_vs_M0
2,29E-26	-1,05827896	0,867	0,78	7,60E-22	NACA	DWW	M2_vs_M0
3,38E-33	-1,0603783	0,955	0,91	1,12E-28	RPS23	DWW	M2_vs_M0
2,41E-36	-1,06058661	0,947	0,9	7,99E-32	RPL30	DWW	M2_vs_M0
1,07E-30	-1,06681187	0,923	0,83	3,54E-26	RPL23A	DWW	M2_vs_M0
4,85E-59	-1,08314145	0,989	0,99	1,61E-54	TMSB10	DWW	M2_vs_M0
2,32E-23	-1,08749019	0,846	0,77	7,67E-19	GAPDH	DWW	M2_vs_M0
3,50E-39	-1,09331884	0,942	0,89	1,16E-34	RPL37A	DWW	M2_vs_M0
5,05E-35	-1,10261824	0,91	0,85	1,67E-30	RPL18	DWW	M2_vs_M0
1,62E-37	-1,10355158	0,931	0,88	5,36E-33	RPL3	DWW	M2_vs_M0
6,06E-43	-1,10840704	0,944	0,91	2,01E-38	RPL32	DWW	M2_vs_M0
2,45E-45	-1,11319125	0,963	0,93	8,12E-41	RPS14	DWW	M2_vs_M0
1,62E-41	-1,12107673	0,926	0,92	5,37E-37	RPL26	DWW	M2_vs_M0
1,50E-36	-1,12822895	0,889	0,86	4,96E-32	RPS7	DWW	M2_vs_M0
1,16E-34	-1,13653671	0,897	0,85	3,83E-30	RPS3	DWW	M2_vs_M0
2,05E-53	-1,13731437	0,952	0,93	6,78E-49	RPL19	DWW	M2_vs_M0
9,56E-59	-1,1450394	0,968	0,97	3,17E-54	RPL10	DWW	M2_vs_M0
9,34E-42	-1,15473605	0,894	0,87	3,09E-37	RPL29	DWW	M2_vs_M0
3,04E-21	-1,1558352	0,958	0,8	1,01E-16	RPL13	DWW	M2_vs_M0
1,80E-50	-1,15758234	0,944	0,93	5,97E-46	RPL37	DWW	M2_vs_M0
1,42E-21	-1,15785432	0,809	0,73	4,70E-17	RPL5	DWW	M2_vs_M0
9,44E-40	-1,16144338	0,91	0,87	3,13E-35	RPL7A	DWW	M2_vs_M0
8,29E-40	-1,16191083	0,902	0,87	2,74E-35	RPS4X	DWW	M2_vs_M0
1,70E-36	-1,16772377	0,894	0,85	5,64E-32	RPS15	DWW	M2_vs_M0

2,27E-18	-1,16882688	0,788	0,7	7,52E-14	RPS5	DWW	M2_vs_M0
2,57E-48	-1,16978352	0,944	0,91	8,52E-44	RPS29	DWW	M2_vs_M0
3,36E-54	-1,17390954	0,968	0,97	1,11E-49	RPS27	DWW	M2_vs_M0
4,48E-27	-1,18118171	0,865	0,78	1,48E-22	RPL8	DWW	M2_vs_M0
4,44E-41	-1,22997065	0,91	0,88	1,47E-36	RPL18A	DWW	M2_vs_M0
7,06E-57	-1,24103321	0,931	0,94	2,34E-52	RPL11	DWW	M2_vs_M0
5,25E-55	-1,24930189	0,928	0,93	1,74E-50	RPS19	DWW	M2_vs_M0
1,76E-28	-1,25217656	0,822	0,76	5,83E-24	RPL10A	DWW	M2_vs_M0
7,40E-33	-1,26595396	0,846	0,79	2,45E-28	RPL14	DWW	M2_vs_M0
8,34E-21	-1,27540992	0,363	0,09	2,76E-16	PLCG2	DWW	M2_vs_M0
1,51E-54	-1,28111841	0,952	0,94	4,99E-50	RPS18	DWW	M2_vs_M0
7,69E-29	-1,49121385	0,915	0,8	2,55E-24	RPS2	DWW	M2_vs_M0
6,18E-11	-1,75813864	0,003	0,11	2,05E-06	IDO1	DWW	M2_vs_M0
7,40E-07	-2,13129808	0,268	0,34	0,024519	LGALS2	DWW	M2_vs_M0

p_val	avg_log2FC	pct.1	pct.2	p_val_adj	gene	sign	comp
3,22E-67	1,92336241	0,796	0,231	1,07E-62	SERPINA1	UPP	IDA_macrophage_vs_M2
2,77E-66	2,61345495	0,582	0	9,18E-62	FCN1	UPP	IDA_macrophage_vs_M2
1,96E-59	1,30380525	0,978	0,798	6,48E-55	SH3BGRL3	UPP	IDA_macrophage_vs_M2
5,34E-58	2,53675897	0,899	0,509	1,77E-53	TIMP1	UPP	IDA_macrophage_vs_M2
1,33E-55	1,5912247	0,853	0,324	4,40E-51	BCL2A1	UPP	IDA_macrophage_vs_M2
1,01E-53	1,5884828	0,807	0,286	3,36E-49	PPA1	UPP	IDA_macrophage_vs_M2
2,84E-52	2,92167841	0,603	0,082	9,42E-48	EREG	UPP	IDA_macrophage_vs_M2
4,07E-52	1,34259857	0,856	0,355	1,35E-47	CD48	UPP	IDA_macrophage_vs_M2
1,93E-51	1,66764066	0,758	0,268	6,38E-47	LGALS2	UPP	IDA_macrophage_vs_M2
6,44E-49	3,5093831	0,505	0,032	2,13E-44	S100A8	UPP	IDA_macrophage_vs_M2
3,49E-45	3,52444935	0,5	0,045	1,16E-40	G0S2	UPP	IDA_macrophage_vs_M2
1,09E-44	1,27076494	0,992	0,703	3,62E-40	NEAT1	UPP	IDA_macrophage_vs_M2
1,24E-44	1,26468529	0,508	0,056	4,10E-40	FPR1	UPP	IDA_macrophage_vs_M2
2,77E-44	1,5285342	0,94	0,581	9,17E-40	BTG1	UPP	IDA_macrophage_vs_M2
5,25E-44	2,43468428	0,647	0,183	1,74E-39	C15orf48	UPP	IDA_macrophage_vs_M2
1,09E-43	1,67916889	0,867	0,438	3,61E-39	SLC2A3	UPP	IDA_macrophage_vs_M2
1,68E-43	0,99628564	0,976	0,751	5,56E-39	LST1	UPP	IDA_macrophage_vs_M2
3,29E-43	1,48171089	0,918	0,57	1,09E-38	S100A6	UPP	IDA_macrophage_vs_M2
9,40E-43	1,27833685	0,538	0,088	3,11E-38	FLNA	UPP	IDA_macrophage_vs_M2
1,89E-42	1,4249665	0,644	0,183	6,25E-38	GK	UPP	IDA_macrophage_vs_M2
5,33E-42	1,79699448	0,671	0,212	1,77E-37	OLR1	UPP	IDA_macrophage_vs_M2
8,45E-41	2,06777241	0,394	0,003	2,80E-36	VCAN	UPP	IDA_macrophage_vs_M2
1,56E-40	1,16370306	0,954	0,655	5,17E-36	COTL1	UPP	IDA_macrophage_vs_M2
2,52E-40	1,1182357	0,704	0,26	8,36E-36	GPCPD1	UPP	IDA_macrophage_vs_M2
4,83E-40	1,22120747	0,981	0,684	1,60E-35	NAMPT	UPP	IDA_macrophage_vs_M2
1,13E-39	2,05118711	0,576	0,143	3,75E-35	IL1RN	UPP	IDA_macrophage_vs_M2
5,48E-39	1,54234321	0,734	0,31	1,82E-34	WARS	UPP	IDA_macrophage_vs_M2
7,71E-39	1,00441116	0,959	0,671	2,55E-34	PTPRC	UPP	IDA_macrophage_vs_M2
1,57E-38	1,60568075	0,894	0,57	5,21E-34	S100A4	UPP	IDA_macrophage_vs_M2
1,99E-38	1,18615308	0,658	0,225	6,60E-34	CYTIP	UPP	IDA_macrophage_vs_M2
4,56E-38	1,51793057	0,552	0,125	1,51E-33	CD52	UPP	IDA_macrophage_vs_M2
6,92E-38	1,15689951	0,796	0,353	2,29E-33	MXD1	UPP	IDA_macrophage_vs_M2
2,19E-37	0,69085396	0,989	0,952	7,25E-33	RPS9	UPP	IDA_macrophage_vs_M2
3,06E-37	1,27165638	0,821	0,406	1,01E-32	SERPINB9	UPP	IDA_macrophage_vs_M2
2,33E-36	1,40909286	0,44	0,05	7,73E-32	CD300E	UPP	IDA_macrophage_vs_M2
6,70E-36	0,83468189	0,995	0,849	2,22E-31	SRGN	UPP	IDA_macrophage_vs_M2
1,02E-35	1,40169021	0,802	0,398	3,38E-31	CXCR4	UPP	IDA_macrophage_vs_M2
1,31E-35	0,95651927	0,812	0,446	4,33E-31	PLSCR1	UPP	IDA_macrophage_vs_M2
3,18E-35	1,27514625	0,375	0,019	1,05E-30	SLC11A1	UPP	IDA_macrophage_vs_M2
1,08E-34	1,21736202	0,462	0,064	3,57E-30	PLAC8	UPP	IDA_macrophage_vs_M2
3,69E-34	1,11971979	0,889	0,541	1,22E-29	CD44	UPP	IDA_macrophage_vs_M2
7,04E-34	1,23826248	0,976	0,711	2,33E-29	SOD2	UPP	IDA_macrophage_vs_M2
9,12E-34	1,16536761	0,758	0,395	3,02E-29	PRELID1	UPP	IDA_macrophage_vs_M2
2,60E-33	0,94125647	0,793	0,416	8,63E-29	MNDA	UPP	IDA_macrophage_vs_M2
4,30E-33	1,09481287	0,951	0,679	1,43E-28	ZFP36	UPP	IDA_macrophage_vs_M2

9,63E-33	0,92831886	0,927	0,687	3,19E-28	LCP1	UPP	IDA_macrophage_vs_M2
9,90E-33	1,09855381	0,736	0,358	3,28E-28	TSPO	UPP	IDA_macrophage_vs_M2
6,56E-32	1,20707112	0,734	0,366	2,17E-27	LDHA	UPP	IDA_macrophage_vs_M2
9,09E-32	1,22120258	1	0,989	3,01E-27	FTH1	UPP	IDA_macrophage_vs_M2
1,60E-31	1,18233373	0,617	0,22	5,31E-27	CD55	UPP	IDA_macrophage_vs_M2
1,64E-31	1,19878149	0,935	0,586	5,42E-27	PLAUR	UPP	IDA_macrophage_vs_M2
2,65E-31	0,86362636	0,883	0,552	8,78E-27	PTPRE	UPP	IDA_macrophage_vs_M2
7,00E-31	0,94333183	0,503	0,125	2,32E-26	RIPK2	UPP	IDA_macrophage_vs_M2
4,08E-30	0,73101194	0,435	0,074	1,35E-25	PID1	UPP	IDA_macrophage_vs_M2
9,08E-30	1,19885798	0,995	0,83	3,01E-25	MALAT1	UPP	IDA_macrophage_vs_M2
1,19E-29	2,32718456	0,476	0,117	3,95E-25	AREG	UPP	IDA_macrophage_vs_M2
1,47E-29	0,65359255	0,995	0,928	4,87E-25	RPS19	UPP	IDA_macrophage_vs_M2
2,54E-29	1,23228343	0,78	0,454	8,41E-25	SOCS3	UPP	IDA_macrophage_vs_M2
2,74E-29	0,90045569	0,728	0,374	9,08E-25	NCF2	UPP	IDA_macrophage_vs_M2
3,51E-29	0,99416029	0,69	0,321	1,16E-24	PIM3	UPP	IDA_macrophage_vs_M2
3,92E-29	1,32532465	0,293	0,003	1,30E-24	IDO1	UPP	IDA_macrophage_vs_M2
4,64E-29	0,86448947	0,723	0,318	1,54E-24	CORO1A	UPP	IDA_macrophage_vs_M2
1,59E-28	0,61742048	0,981	0,91	5,28E-24	RPL18A	UPP	IDA_macrophage_vs_M2
1,63E-28	0,92518969	0,473	0,114	5,39E-24	CFP	UPP	IDA_macrophage_vs_M2
1,97E-28	1,34550748	0,72	0,377	6,54E-24	PPIF	UPP	IDA_macrophage_vs_M2
3,11E-28	0,85070233	0,641	0,271	1,03E-23	RASSF5	UPP	IDA_macrophage_vs_M2
5,65E-28	0,94405193	0,397	0,069	1,87E-23	TREM1	UPP	IDA_macrophage_vs_M2
6,64E-28	0,68787565	0,652	0,271	2,20E-23	CSTA	UPP	IDA_macrophage_vs_M2
1,28E-27	0,86552069	0,69	0,345	4,25E-23	BID	UPP	IDA_macrophage_vs_M2
1,39E-27	0,83524863	0,557	0,191	4,60E-23	GLIPR2	UPP	IDA_macrophage_vs_M2
1,72E-27	0,86917011	0,796	0,406	5,69E-23	KDM6B	UPP	IDA_macrophage_vs_M2
2,34E-27	0,817235	0,367	0,053	7,77E-23	FGR	UPP	IDA_macrophage_vs_M2
4,22E-27	1,31569682	0,535	0,183	1,40E-22	BASP1	UPP	IDA_macrophage_vs_M2
4,43E-27	0,56247323	0,992	0,955	1,47E-22	RPS24	UPP	IDA_macrophage_vs_M2
7,03E-27	0,50397012	0,995	0,976	2,33E-22	RPL28	UPP	IDA_macrophage_vs_M2
9,67E-27	0,80185476	0,764	0,414	3,20E-22	STK17B	UPP	IDA_macrophage_vs_M2
2,04E-26	0,99066821	0,288	0,011	6,75E-22	AQP9	UPP	IDA_macrophage_vs_M2
2,27E-26	2,76964977	0,685	0,422	7,52E-22	S100A9	UPP	IDA_macrophage_vs_M2
2,71E-26	0,77523168	0,897	0,621	8,97E-22	PSME2	UPP	IDA_macrophage_vs_M2
2,97E-26	0,73907978	0,28	0,008	9,83E-22	AC245128.3	UPP	IDA_macrophage_vs_M2
4,94E-26	0,845384	0,652	0,318	1,64E-21	IFITM2	UPP	IDA_macrophage_vs_M2
7,16E-26	1,01948441	0,815	0,509	2,37E-21	IFITM3	UPP	IDA_macrophage_vs_M2
8,91E-26	0,80088291	0,88	0,517	2,95E-21	REL	UPP	IDA_macrophage_vs_M2
1,24E-25	0,95968964	0,454	0,122	4,12E-21	LILRA5	UPP	IDA_macrophage_vs_M2
1,44E-25	0,83205304	0,329	0,034	4,78E-21	NRG1	UPP	IDA_macrophage_vs_M2
1,64E-25	0,94432642	0,524	0,199	5,43E-21	RALA	UPP	IDA_macrophage_vs_M2
1,93E-25	0,79830626	0,408	0,09	6,38E-21	LYST	UPP	IDA_macrophage_vs_M2
4,62E-25	0,80733406	0,579	0,228	1,53E-20	CYTOR	UPP	IDA_macrophage_vs_M2
5,08E-25	1,03321151	0,451	0,127	1,68E-20	ATP2B1-AS1	UPP	IDA_macrophage_vs_M2
7,84E-25	0,91719742	0,851	0,509	2,60E-20	C5AR1	UPP	IDA_macrophage_vs_M2
1,11E-24	0,67467169	0,989	0,894	3,69E-20	H3F3A	UPP	IDA_macrophage_vs_M2

1,49E-24	0,95668587	0,935	0,639	4,93E-20	GPR183	UPP	IDA_macrophage_vs_M2
2,74E-24	0,6786787	0,462	0,138	9,09E-20	MYD88	UPP	IDA_macrophage_vs_M2
3,05E-24	0,7252641	0,527	0,191	1,01E-19	FGD4	UPP	IDA_macrophage_vs_M2
3,39E-24	0,73822113	0,609	0,247	1,12E-19	ADGRE2	UPP	IDA_macrophage_vs_M2
4,16E-24	1,50580679	0,707	0,345	1,38E-19	IL1B	UPP	IDA_macrophage_vs_M2
6,22E-24	0,88290292	0,812	0,538	2,06E-19	SERPINB1	UPP	IDA_macrophage_vs_M2
6,60E-24	0,65347094	0,345	0,05	2,19E-19	VDR	UPP	IDA_macrophage_vs_M2
1,75E-23	1,46852225	0,56	0,236	5,79E-19	DDIT4	UPP	IDA_macrophage_vs_M2
2,58E-23	1,33794712	0,867	0,586	8,53E-19	NFKBIA	UPP	IDA_macrophage_vs_M2
4,92E-23	0,88968022	0,231	0	1,63E-18	S100A12	UPP	IDA_macrophage_vs_M2
7,88E-23	0,99804174	0,832	0,499	2,61E-18	TYMP	UPP	IDA_macrophage_vs_M2
9,84E-23	0,91253412	0,867	0,557	3,26E-18	SGK1	UPP	IDA_macrophage_vs_M2
1,22E-22	0,78496585	0,655	0,316	4,04E-18	TET2	UPP	IDA_macrophage_vs_M2
1,43E-22	0,69934851	0,408	0,111	4,73E-18	PSTPIP2	UPP	IDA_macrophage_vs_M2
1,90E-22	0,64349732	0,736	0,406	6,31E-18	CARD16	UPP	IDA_macrophage_vs_M2
6,83E-22	0,73226639	0,668	0,347	2,26E-17	RPL22L1	UPP	IDA_macrophage_vs_M2
7,12E-22	1,41021722	0,717	0,43	2,36E-17	PLIN2	UPP	IDA_macrophage_vs_M2
8,07E-22	0,75366776	0,807	0,475	2,67E-17	PPP1R15A	UPP	IDA_macrophage_vs_M2
8,78E-22	0,55526595	0,424	0,114	2,91E-17	ASGR1	UPP	IDA_macrophage_vs_M2
9,06E-22	0,77908544	0,802	0,491	3,00E-17	ATP2B1	UPP	IDA_macrophage_vs_M2
9,66E-22	0,52481181	0,753	0,411	3,20E-17	PSMB9	UPP	IDA_macrophage_vs_M2
1,37E-21	1,06095534	0,823	0,557	4,53E-17	S100A10	UPP	IDA_macrophage_vs_M2
1,73E-21	0,92712532	0,663	0,34	5,73E-17	BHLHE40	UPP	IDA_macrophage_vs_M2
1,96E-21	0,9495511	0,668	0,371	6,48E-17	IFI30	UPP	IDA_macrophage_vs_M2
5,95E-21	0,58275145	0,69	0,34	1,97E-16	ITGAX	UPP	IDA_macrophage_vs_M2
9,25E-21	0,81976054	0,764	0,419	3,06E-16	NR4A1	UPP	IDA_macrophage_vs_M2
1,45E-20	0,56322326	0,666	0,361	4,81E-16	ATG3	UPP	IDA_macrophage_vs_M2
1,75E-20	0,94365623	0,685	0,382	5,80E-16	PFKFB3	UPP	IDA_macrophage_vs_M2
1,84E-20	0,63315662	0,37	0,088	6,11E-16	CLEC4E	UPP	IDA_macrophage_vs_M2
2,19E-20	0,62255098	0,772	0,464	7,25E-16	RNF149	UPP	IDA_macrophage_vs_M2
2,41E-20	0,71873209	0,658	0,334	7,99E-16	BACH1	UPP	IDA_macrophage_vs_M2
3,69E-20	0,73615895	0,766	0,416	1,22E-15	VMP1	UPP	IDA_macrophage_vs_M2
4,56E-20	0,63652358	0,682	0,366	1,51E-15	CHD1	UPP	IDA_macrophage_vs_M2
5,11E-20	0,77668312	0,312	0,056	1,69E-15	ANPEP	UPP	IDA_macrophage_vs_M2
5,58E-20	0,65801783	0,666	0,366	1,85E-15	FAM49A	UPP	IDA_macrophage_vs_M2
5,85E-20	0,81896034	0,383	0,106	1,94E-15	SLC25A37	UPP	IDA_macrophage_vs_M2
7,46E-20	0,46150506	0,995	0,968	2,47E-15	RPL10	UPP	IDA_macrophage_vs_M2
9,29E-20	0,64804743	0,543	0,236	3,08E-15	UPP1	UPP	IDA_macrophage_vs_M2
1,07E-19	0,46151618	0,989	0,912	3,54E-15	FAU	UPP	IDA_macrophage_vs_M2
1,12E-19	1,21663532	0,557	0,247	3,72E-15	THBS1	UPP	IDA_macrophage_vs_M2
1,56E-19	0,84200482	0,446	0,156	5,16E-15	CCRL2	UPP	IDA_macrophage_vs_M2
1,60E-19	0,6302863	0,332	0,074	5,32E-15	MYO1G	UPP	IDA_macrophage_vs_M2
1,78E-19	0,47974013	0,755	0,451	5,88E-15	VSIR	UPP	IDA_macrophage_vs_M2
2,12E-19	0,55202151	0,285	0,048	7,03E-15	SPATA13	UPP	IDA_macrophage_vs_M2
2,32E-19	0,60991109	0,976	0,915	7,69E-15	RPS2	UPP	IDA_macrophage_vs_M2
2,80E-19	0,70665788	0,97	0,958	9,27E-15	RPL13	UPP	IDA_macrophage_vs_M2

2,96E-19	0,37877027	0,992	0,939	9,81E-15	UBA52	UPP	IDA_macrophage_vs_M2
3,58E-19	0,53376589	0,924	0,658	1,19E-14	TPI1	UPP	IDA_macrophage_vs_M2
4,03E-19	0,56647201	0,845	0,592	1,33E-14	GNG5	UPP	IDA_macrophage_vs_M2
4,64E-19	1,16622532	0,671	0,342	1,54E-14	DUSP2	UPP	IDA_macrophage_vs_M2
4,69E-19	0,85773287	0,598	0,321	1,55E-14	LILRB1	UPP	IDA_macrophage_vs_M2
5,03E-19	0,45946496	0,693	0,387	1,67E-14	PSMB8	UPP	IDA_macrophage_vs_M2
5,15E-19	0,90363997	0,625	0,324	1,71E-14	GBP1	UPP	IDA_macrophage_vs_M2
7,46E-19	0,90515275	0,614	0,313	2,47E-14	PRDM1	UPP	IDA_macrophage_vs_M2
9,00E-19	0,49898682	0,196	0,003	2,98E-14	RIPOR2	UPP	IDA_macrophage_vs_M2
1,00E-18	0,71467806	0,731	0,43	3,32E-14	ADGRE5	UPP	IDA_macrophage_vs_M2
1,44E-18	0,80481729	0,649	0,371	4,78E-14	ETS2	UPP	IDA_macrophage_vs_M2
1,56E-18	0,41399685	0,986	0,947	5,16E-14	RPL30	UPP	IDA_macrophage_vs_M2
2,01E-18	1,08677569	0,658	0,379	6,65E-14	TUBA1A	UPP	IDA_macrophage_vs_M2
2,15E-18	0,7215574	0,693	0,419	7,11E-14	RAB20	UPP	IDA_macrophage_vs_M2
2,33E-18	0,56407198	0,573	0,276	7,73E-14	GBP2	UPP	IDA_macrophage_vs_M2
3,15E-18	0,5143886	0,293	0,056	1,04E-13	CEACAM4	UPP	IDA_macrophage_vs_M2
3,73E-18	0,69933774	0,535	0,252	1,24E-13	KYNU	UPP	IDA_macrophage_vs_M2
4,48E-18	0,85463786	0,861	0,565	1,49E-13	SELENOK	UPP	IDA_macrophage_vs_M2
5,28E-18	0,57257947	0,391	0,122	1,75E-13	LIMD2	UPP	IDA_macrophage_vs_M2
6,49E-18	0,86938873	0,707	0,401	2,15E-13	RASGEF1B	UPP	IDA_macrophage_vs_M2
6,79E-18	0,53021152	0,554	0,263	2,25E-13	TCIRG1	UPP	IDA_macrophage_vs_M2
1,15E-17	0,53343727	0,992	0,936	3,80E-13	RPS8	UPP	IDA_macrophage_vs_M2
1,22E-17	0,57333802	0,731	0,454	4,03E-13	C1orf162	UPP	IDA_macrophage_vs_M2
1,34E-17	0,63757223	0,78	0,517	4,44E-13	PTP4A2	UPP	IDA_macrophage_vs_M2
1,47E-17	0,66513597	0,622	0,347	4,86E-13	PNPLA8	UPP	IDA_macrophage_vs_M2
1,62E-17	0,62055317	0,514	0,241	5,38E-13	FMNL1	UPP	IDA_macrophage_vs_M2
1,63E-17	0,54008717	0,965	0,822	5,39E-13	RPL10A	UPP	IDA_macrophage_vs_M2
2,02E-17	0,54062632	0,348	0,095	6,69E-13	CD1D	UPP	IDA_macrophage_vs_M2
2,10E-17	0,59228132	0,554	0,276	6,95E-13	UBE2J1	UPP	IDA_macrophage_vs_M2
2,10E-17	0,72526813	0,31	0,072	6,97E-13	SMIM25	UPP	IDA_macrophage_vs_M2
2,92E-17	0,445185	0,973	0,867	9,66E-13	NACA	UPP	IDA_macrophage_vs_M2
2,99E-17	0,80314383	0,84	0,597	9,91E-13	PLEK	UPP	IDA_macrophage_vs_M2
3,11E-17	0,61113409	0,97	0,846	1,03E-12	GAPDH	UPP	IDA_macrophage_vs_M2
3,91E-17	0,7962294	0,81	0,589	1,30E-12	LIMS1	UPP	IDA_macrophage_vs_M2
4,41E-17	0,50748634	0,326	0,085	1,46E-12	THAP9-AS1	UPP	IDA_macrophage_vs_M2
4,70E-17	0,65452429	0,38	0,122	1,56E-12	PIM1	UPP	IDA_macrophage_vs_M2
5,08E-17	0,57725423	0,549	0,265	1,68E-12	GCA	UPP	IDA_macrophage_vs_M2
6,23E-17	0,93458435	0,372	0,122	2,06E-12	SNAI1	UPP	IDA_macrophage_vs_M2
6,62E-17	0,59708288	0,804	0,501	2,19E-12	CLEC7A	UPP	IDA_macrophage_vs_M2
6,68E-17	0,48014197	0,823	0,61	2,21E-12	PSME1	UPP	IDA_macrophage_vs_M2
7,68E-17	0,58473127	0,769	0,44	2,55E-12	PDE4B	UPP	IDA_macrophage_vs_M2
8,54E-17	0,61121667	0,405	0,141	2,83E-12	CDC42EP2	UPP	IDA_macrophage_vs_M2
8,78E-17	0,65670625	0,56	0,294	2,91E-12	STXBP2	UPP	IDA_macrophage_vs_M2
9,58E-17	0,55996717	0,799	0,538	3,17E-12	HNRNPDL	UPP	IDA_macrophage_vs_M2
9,93E-17	0,63049719	0,747	0,493	3,29E-12	DDX21	UPP	IDA_macrophage_vs_M2
1,08E-16	0,60479752	0,383	0,133	3,59E-12	MIR4435-2I	UPP	IDA_macrophage_vs_M2

1,15E-16	0,5718614	0,842	0,549	3,80E-12	CYBA	UPP	IDA_macrophage_vs_M2
1,53E-16	0,40564529	0,549	0,252	5,07E-12	POLE4	UPP	IDA_macrophage_vs_M2
1,92E-16	0,45902681	0,69	0,414	6,36E-12	PTPN6	UPP	IDA_macrophage_vs_M2
2,14E-16	0,59489471	0,207	0,019	7,10E-12	MCEMP1	UPP	IDA_macrophage_vs_M2
2,20E-16	0,65937535	0,693	0,411	7,28E-12	ATP13A3	UPP	IDA_macrophage_vs_M2
2,45E-16	0,44033868	0,859	0,488	8,10E-12	FOSL2	UPP	IDA_macrophage_vs_M2
2,72E-16	0,4897169	0,867	0,589	9,02E-12	GSTO1	UPP	IDA_macrophage_vs_M2
2,94E-16	0,5398917	0,228	0,032	9,75E-12	ADAM8	UPP	IDA_macrophage_vs_M2
3,18E-16	0,53158256	0,576	0,3	1,05E-11	PAK1	UPP	IDA_macrophage_vs_M2
3,34E-16	0,46194634	0,815	0,549	1,11E-11	PSMB3	UPP	IDA_macrophage_vs_M2
3,57E-16	0,51838264	0,478	0,207	1,18E-11	ETV6	UPP	IDA_macrophage_vs_M2
3,87E-16	0,45207299	0,995	0,931	1,28E-11	RPL11	UPP	IDA_macrophage_vs_M2
4,56E-16	0,72366816	0,668	0,39	1,51E-11	WTAP	UPP	IDA_macrophage_vs_M2
4,74E-16	0,56200368	0,989	0,857	1,57E-11	H3F3B	UPP	IDA_macrophage_vs_M2
5,25E-16	0,49795403	0,997	0,973	1,74E-11	ACTB	UPP	IDA_macrophage_vs_M2
5,44E-16	0,67580342	0,66	0,379	1,80E-11	LILRB2	UPP	IDA_macrophage_vs_M2
6,39E-16	0,36130763	0,226	0,029	2,12E-11	LILRA1	UPP	IDA_macrophage_vs_M2
7,69E-16	0,71021464	0,603	0,34	2,55E-11	RUNX3	UPP	IDA_macrophage_vs_M2
9,88E-16	0,5071558	0,745	0,467	3,27E-11	RBM8A	UPP	IDA_macrophage_vs_M2
1,07E-15	0,83322403	0,962	0,772	3,55E-11	VIM	UPP	IDA_macrophage_vs_M2
1,22E-15	0,52591606	0,663	0,385	4,03E-11	KMT2E	UPP	IDA_macrophage_vs_M2
1,49E-15	0,40716827	0,981	0,915	4,94E-11	S100A11	UPP	IDA_macrophage_vs_M2
1,69E-15	0,54891906	0,522	0,268	5,61E-11	CPPED1	UPP	IDA_macrophage_vs_M2
1,76E-15	0,55191958	0,484	0,225	5,84E-11	LILRB3	UPP	IDA_macrophage_vs_M2
1,79E-15	0,52258114	0,818	0,533	5,91E-11	SRSF5	UPP	IDA_macrophage_vs_M2
1,85E-15	0,41485647	0,986	0,907	6,14E-11	RPS16	UPP	IDA_macrophage_vs_M2
1,89E-15	0,57946136	0,804	0,546	6,25E-11	LRRFIP1	UPP	IDA_macrophage_vs_M2
2,20E-15	0,43594297	0,959	0,841	7,29E-11	ARPC3	UPP	IDA_macrophage_vs_M2
2,36E-15	0,50927906	0,459	0,202	7,82E-11	RAC2	UPP	IDA_macrophage_vs_M2
2,59E-15	0,4257352	0,815	0,552	8,57E-11	FKBP1A	UPP	IDA_macrophage_vs_M2
2,60E-15	0,5053706	0,546	0,276	8,62E-11	PRKCB	UPP	IDA_macrophage_vs_M2
2,98E-15	0,45619858	0,889	0,642	9,86E-11	EIF3K	UPP	IDA_macrophage_vs_M2
3,02E-15	0,43172486	0,228	0,034	1,00E-10	ANKRD22	UPP	IDA_macrophage_vs_M2
3,04E-15	0,65370847	0,938	0,692	1,01E-10	JUNB	UPP	IDA_macrophage_vs_M2
3,63E-15	0,34124984	0,864	0,658	1,20E-10	RPS10	UPP	IDA_macrophage_vs_M2
3,68E-15	1,34012058	0,391	0,146	1,22E-10	OSM	UPP	IDA_macrophage_vs_M2
3,70E-15	0,39963697	0,467	0,204	1,22E-10	SMCO4	UPP	IDA_macrophage_vs_M2
4,00E-15	0,54492705	0,288	0,072	1,33E-10	NEDD9	UPP	IDA_macrophage_vs_M2
4,66E-15	0,44234671	0,228	0,037	1,54E-10	CCR5	UPP	IDA_macrophage_vs_M2
5,29E-15	0,40327484	0,989	0,944	1,75E-10	RPL32	UPP	IDA_macrophage_vs_M2
5,34E-15	0,61645655	0,495	0,231	1,77E-10	AC016831.5	UPP	IDA_macrophage_vs_M2
5,84E-15	0,58231418	0,44	0,196	1,93E-10	IRAK3	UPP	IDA_macrophage_vs_M2
6,87E-15	0,59064729	0,679	0,411	2,28E-10	IRF2BP2	UPP	IDA_macrophage_vs_M2
6,98E-15	0,57807055	0,84	0,57	2,31E-10	HIF1A	UPP	IDA_macrophage_vs_M2
7,05E-15	0,43278321	0,777	0,504	2,33E-10	CIB1	UPP	IDA_macrophage_vs_M2
7,35E-15	0,46276955	0,302	0,082	2,43E-10	HRH2	UPP	IDA_macrophage_vs_M2

7,47E-15	0,70883696	0,679	0,408	2,48E-10	VEGFA	UPP	IDA_macrophage_vs_M2
7,52E-15	0,62779898	0,505	0,244	2,49E-10	TNFSF10	UPP	IDA_macrophage_vs_M2
8,21E-15	0,47013225	0,764	0,504	2,72E-10	LYN	UPP	IDA_macrophage_vs_M2
9,85E-15	0,47591476	0,986	0,889	3,26E-10	RPS7	UPP	IDA_macrophage_vs_M2
1,31E-14	0,43405073	0,943	0,775	4,34E-10	PFN1	UPP	IDA_macrophage_vs_M2
1,40E-14	0,51363295	0,967	0,865	4,65E-10	RPL8	UPP	IDA_macrophage_vs_M2
1,45E-14	0,47891265	0,758	0,509	4,79E-10	GUK1	UPP	IDA_macrophage_vs_M2
1,45E-14	0,40074208	0,984	0,91	4,81E-10	RPL18	UPP	IDA_macrophage_vs_M2
1,56E-14	0,37056003	0,609	0,308	5,17E-10	LSP1	UPP	IDA_macrophage_vs_M2
1,80E-14	0,4754861	0,655	0,379	5,95E-10	ZNF267	UPP	IDA_macrophage_vs_M2
1,86E-14	0,48720244	0,375	0,138	6,17E-10	IRF7	UPP	IDA_macrophage_vs_M2
1,94E-14	0,41479631	0,897	0,679	6,42E-10	CHCHD2	UPP	IDA_macrophage_vs_M2
2,09E-14	0,44191174	0,742	0,456	6,93E-10	JMJD1C	UPP	IDA_macrophage_vs_M2
2,12E-14	0,37693529	0,582	0,294	7,02E-10	LSM7	UPP	IDA_macrophage_vs_M2
2,14E-14	0,50086956	0,383	0,146	7,09E-10	CBX6	UPP	IDA_macrophage_vs_M2
2,14E-14	0,45622632	0,821	0,586	7,11E-10	ZFAS1	UPP	IDA_macrophage_vs_M2
2,61E-14	0,50470964	0,565	0,292	8,66E-10	METRNL	UPP	IDA_macrophage_vs_M2
2,97E-14	0,38865525	0,228	0,042	9,84E-10	LMNB1	UPP	IDA_macrophage_vs_M2
3,23E-14	0,41277408	0,66	0,395	1,07E-09	EIF5A	UPP	IDA_macrophage_vs_M2
3,35E-14	0,78152122	0,91	0,653	1,11E-09	KLF6	UPP	IDA_macrophage_vs_M2
3,76E-14	0,3820894	0,989	0,841	1,25E-09	RPL24	UPP	IDA_macrophage_vs_M2
4,12E-14	0,35966721	0,204	0,029	1,36E-09	PRAM1	UPP	IDA_macrophage_vs_M2
4,14E-14	0,44750725	0,747	0,477	1,37E-09	LCP2	UPP	IDA_macrophage_vs_M2
4,51E-14	0,70508035	0,946	0,69	1,49E-09	DUSP1	UPP	IDA_macrophage_vs_M2
5,06E-14	0,42191713	0,959	0,894	1,67E-09	RPS15	UPP	IDA_macrophage_vs_M2
5,09E-14	0,52864899	0,383	0,143	1,68E-09	MARCKSL1	UPP	IDA_macrophage_vs_M2
5,11E-14	0,37825783	0,935	0,7	1,69E-09	SUB1	UPP	IDA_macrophage_vs_M2
5,49E-14	0,27016712	0,997	0,981	1,82E-09	RPL41	UPP	IDA_macrophage_vs_M2
6,33E-14	0,49160769	0,644	0,379	2,10E-09	PHPT1	UPP	IDA_macrophage_vs_M2
6,63E-14	0,43378717	0,962	0,846	2,20E-09	RPL14	UPP	IDA_macrophage_vs_M2
6,74E-14	0,53365406	0,978	0,703	2,23E-09	MCL1	UPP	IDA_macrophage_vs_M2
7,18E-14	0,60197104	0,543	0,289	2,38E-09	AKIRIN2	UPP	IDA_macrophage_vs_M2
7,37E-14	0,46342459	0,766	0,533	2,44E-09	RAP1B	UPP	IDA_macrophage_vs_M2
7,50E-14	0,84641517	0,78	0,57	2,48E-09	IER3	UPP	IDA_macrophage_vs_M2
9,86E-14	0,34795842	0,524	0,265	3,27E-09	PTPN2	UPP	IDA_macrophage_vs_M2
1,01E-13	1,14258291	0,158	0,008	3,36E-09	APOBEC3A	UPP	IDA_macrophage_vs_M2
1,14E-13	0,38224636	0,174	0,016	3,78E-09	GGT5	UPP	IDA_macrophage_vs_M2
1,37E-13	0,55621214	0,81	0,57	4,54E-09	SAMSN1	UPP	IDA_macrophage_vs_M2
1,56E-13	0,40470833	0,978	0,875	5,17E-09	RPL6	UPP	IDA_macrophage_vs_M2
1,74E-13	0,51008236	0,312	0,103	5,76E-09	FBP1	UPP	IDA_macrophage_vs_M2
1,96E-13	0,42017655	0,188	0,024	6,48E-09	FPR2	UPP	IDA_macrophage_vs_M2
2,00E-13	0,41767191	0,255	0,064	6,63E-09	MYOF	UPP	IDA_macrophage_vs_M2
2,13E-13	0,41930903	0,878	0,626	7,07E-09	CMTM6	UPP	IDA_macrophage_vs_M2
2,58E-13	0,61655547	0,443	0,199	8,56E-09	EHD1	UPP	IDA_macrophage_vs_M2
2,67E-13	0,4281871	0,677	0,398	8,85E-09	ZNF207	UPP	IDA_macrophage_vs_M2
2,86E-13	0,35682384	0,264	0,069	9,46E-09	JAK3	UPP	IDA_macrophage_vs_M2

2,98E-13	0,36285356	0,625	0,347	9,88E-09	DDT	UPP	IDA_macrophage_vs_M2
3,12E-13	0,46797364	0,791	0,552	1,03E-08	TPM4	UPP	IDA_macrophage_vs_M2
3,19E-13	0,36957051	0,685	0,424	1,06E-08	SNRPB	UPP	IDA_macrophage_vs_M2
3,75E-13	0,5374059	0,584	0,326	1,24E-08	ARL5B	UPP	IDA_macrophage_vs_M2
3,78E-13	0,44313105	0,364	0,141	1,25E-08	MPHOSPH6	UPP	IDA_macrophage_vs_M2
3,79E-13	0,47126045	0,394	0,17	1,26E-08	CSF3R	UPP	IDA_macrophage_vs_M2
4,29E-13	0,39329056	0,734	0,475	1,42E-08	TKT	UPP	IDA_macrophage_vs_M2
4,72E-13	0,60552208	0,644	0,382	1,56E-08	SYAP1	UPP	IDA_macrophage_vs_M2
5,44E-13	0,4098033	0,348	0,127	1,80E-08	GCH1	UPP	IDA_macrophage_vs_M2
6,04E-13	0,63952043	0,44	0,212	2,00E-08	DDIT3	UPP	IDA_macrophage_vs_M2
6,20E-13	0,5267431	0,372	0,151	2,05E-08	ISG20	UPP	IDA_macrophage_vs_M2
6,67E-13	0,54355339	0,698	0,43	2,21E-08	HBEGF	UPP	IDA_macrophage_vs_M2
7,21E-13	0,92007609	0,141	0,005	2,39E-08	RETN	UPP	IDA_macrophage_vs_M2
7,48E-13	0,53786127	0,671	0,44	2,48E-08	VAMP5	UPP	IDA_macrophage_vs_M2
7,84E-13	0,43438265	0,332	0,117	2,60E-08	CLEC12A	UPP	IDA_macrophage_vs_M2
9,97E-13	0,45386267	0,359	0,143	3,30E-08	TES	UPP	IDA_macrophage_vs_M2
1,08E-12	0,49511138	0,495	0,26	3,59E-08	TAF1D	UPP	IDA_macrophage_vs_M2
1,17E-12	0,33632183	0,152	0,011	3,88E-08	AC005280.2	UPP	IDA_macrophage_vs_M2
1,29E-12	0,47999089	0,408	0,178	4,28E-08	SNHG15	UPP	IDA_macrophage_vs_M2
1,32E-12	0,37242564	0,584	0,329	4,38E-08	ZNHIT1	UPP	IDA_macrophage_vs_M2
1,32E-12	0,33907056	0,995	0,963	4,39E-08	RPS14	UPP	IDA_macrophage_vs_M2
1,34E-12	0,29481197	0,997	0,958	4,45E-08	PTMA	UPP	IDA_macrophage_vs_M2
1,37E-12	0,40963584	0,712	0,454	4,54E-08	VASP	UPP	IDA_macrophage_vs_M2
1,43E-12	0,39184629	0,568	0,324	4,72E-08	PGLS	UPP	IDA_macrophage_vs_M2
1,54E-12	0,52091244	0,595	0,379	5,09E-08	ALDH2	UPP	IDA_macrophage_vs_M2
1,65E-12	0,74229265	0,524	0,294	5,48E-08	TAGAP	UPP	IDA_macrophage_vs_M2
1,84E-12	0,53574343	0,723	0,464	6,08E-08	RILPL2	UPP	IDA_macrophage_vs_M2
2,00E-12	0,43940004	0,696	0,422	6,64E-08	STX11	UPP	IDA_macrophage_vs_M2
2,03E-12	0,42831424	0,693	0,408	6,71E-08	PPP1R15B	UPP	IDA_macrophage_vs_M2
2,07E-12	0,50183369	0,367	0,159	6,84E-08	AMPD2	UPP	IDA_macrophage_vs_M2
2,07E-12	0,68130863	0,402	0,175	6,84E-08	GBP5	UPP	IDA_macrophage_vs_M2
2,47E-12	0,47269782	0,291	0,098	8,18E-08	LRRK2	UPP	IDA_macrophage_vs_M2
2,89E-12	0,33853212	0,799	0,531	9,59E-08	AKAP13	UPP	IDA_macrophage_vs_M2
3,02E-12	0,31542888	0,402	0,17	1,00E-07	TGFB1	UPP	IDA_macrophage_vs_M2
3,07E-12	0,39048383	0,372	0,164	1,02E-07	AGTRAP	UPP	IDA_macrophage_vs_M2
3,73E-12	1,27773877	0,198	0,037	1,24E-07	INHBA	UPP	IDA_macrophage_vs_M2
4,26E-12	0,4363841	0,91	0,706	1,41E-07	HNRNPA2B1	UPP	IDA_macrophage_vs_M2
5,64E-12	0,42307799	0,815	0,613	1,87E-07	ACTR3	UPP	IDA_macrophage_vs_M2
5,93E-12	0,4280712	0,938	0,809	1,96E-07	RPL5	UPP	IDA_macrophage_vs_M2
6,32E-12	0,46294223	0,446	0,212	2,09E-07	NDEL1	UPP	IDA_macrophage_vs_M2
6,39E-12	0,79496481	0,636	0,401	2,12E-07	NFKBIZ	UPP	IDA_macrophage_vs_M2
7,00E-12	0,41208469	0,386	0,17	2,32E-07	CAMK2D	UPP	IDA_macrophage_vs_M2
7,37E-12	0,75933569	0,571	0,297	2,44E-07	MT2A	UPP	IDA_macrophage_vs_M2
8,57E-12	0,37304808	0,628	0,385	2,84E-07	BAZ1A	UPP	IDA_macrophage_vs_M2
8,77E-12	0,30682935	0,701	0,462	2,91E-07	SNRPG	UPP	IDA_macrophage_vs_M2
8,99E-12	0,33516816	0,598	0,361	2,98E-07	WAS	UPP	IDA_macrophage_vs_M2

9,17E-12	0,36524415	0,647	0,414	3,04E-07	sep-07	UPP	IDA_macrophage_vs_M2
9,20E-12	0,42187736	0,739	0,491	3,05E-07	GLRX	UPP	IDA_macrophage_vs_M2
9,78E-12	0,38199225	0,427	0,204	3,24E-07	ARF5	UPP	IDA_macrophage_vs_M2
1,00E-11	0,3244763	0,215	0,048	3,33E-07	HOTAIRM1	UPP	IDA_macrophage_vs_M2
1,06E-11	0,38789611	0,655	0,374	3,51E-07	CSRNP1	UPP	IDA_macrophage_vs_M2
1,10E-11	0,38396179	0,84	0,594	3,64E-07	NFE2L2	UPP	IDA_macrophage_vs_M2
1,23E-11	0,38250513	0,639	0,406	4,06E-07	EIF3F	UPP	IDA_macrophage_vs_M2
1,28E-11	0,39423629	0,56	0,332	4,24E-07	PEA15	UPP	IDA_macrophage_vs_M2
1,28E-11	0,50605795	0,435	0,215	4,25E-07	NFIL3	UPP	IDA_macrophage_vs_M2
1,32E-11	0,4330292	0,889	0,647	4,37E-07	ZEB2	UPP	IDA_macrophage_vs_M2
1,47E-11	0,54969839	0,633	0,419	4,87E-07	DSE	UPP	IDA_macrophage_vs_M2
1,49E-11	0,34177405	0,899	0,676	4,94E-07	SRSF3	UPP	IDA_macrophage_vs_M2
1,51E-11	0,26771102	0,158	0,019	5,00E-07	LILRA2	UPP	IDA_macrophage_vs_M2
1,55E-11	0,45096791	0,592	0,374	5,15E-07	PGAM1	UPP	IDA_macrophage_vs_M2
1,68E-11	0,49451404	0,799	0,568	5,57E-07	WSB1	UPP	IDA_macrophage_vs_M2
1,72E-11	0,46291811	0,562	0,332	5,71E-07	CLEC4A	UPP	IDA_macrophage_vs_M2
1,87E-11	0,56178248	0,755	0,546	6,21E-07	EMP3	UPP	IDA_macrophage_vs_M2
1,95E-11	0,42784779	0,226	0,056	6,47E-07	GPR84	UPP	IDA_macrophage_vs_M2
2,03E-11	0,38672957	0,709	0,483	6,71E-07	PYCARD	UPP	IDA_macrophage_vs_M2
2,06E-11	0,4354944	0,399	0,175	6,82E-07	GPR132	UPP	IDA_macrophage_vs_M2
2,09E-11	0,43301079	0,139	0,011	6,92E-07	CLEC5A	UPP	IDA_macrophage_vs_M2
2,10E-11	0,89910927	0,535	0,313	6,97E-07	ISG15	UPP	IDA_macrophage_vs_M2
2,14E-11	0,39405916	0,541	0,302	7,07E-07	PTPN1	UPP	IDA_macrophage_vs_M2
2,46E-11	0,28768356	0,144	0,013	8,15E-07	CCR2	UPP	IDA_macrophage_vs_M2
2,49E-11	0,56906579	0,391	0,194	8,24E-07	LTA4H	UPP	IDA_macrophage_vs_M2
2,50E-11	0,38442771	0,274	0,09	8,27E-07	ODF3B	UPP	IDA_macrophage_vs_M2
2,58E-11	0,36388604	0,723	0,501	8,54E-07	GPSM3	UPP	IDA_macrophage_vs_M2
2,66E-11	0,5505187	0,56	0,321	8,82E-07	GABARAPL1	UPP	IDA_macrophage_vs_M2
3,07E-11	0,36336048	0,668	0,435	1,02E-06	STAT3	UPP	IDA_macrophage_vs_M2
3,26E-11	0,31740099	1	0,989	1,08E-06	TMSB10	UPP	IDA_macrophage_vs_M2
3,30E-11	0,3974383	0,842	0,639	1,09E-06	HNRNPA3	UPP	IDA_macrophage_vs_M2
3,34E-11	0,56499303	0,875	0,607	1,11E-06	RGS2	UPP	IDA_macrophage_vs_M2
3,77E-11	0,29353971	0,486	0,263	1,25E-06	PARVG	UPP	IDA_macrophage_vs_M2
3,91E-11	0,41911134	0,84	0,639	1,30E-06	FYB1	UPP	IDA_macrophage_vs_M2
4,40E-11	0,47753043	0,334	0,146	1,46E-06	ERICH1	UPP	IDA_macrophage_vs_M2
4,78E-11	0,5144643	0,122	0,005	1,58E-06	FLT1	UPP	IDA_macrophage_vs_M2
4,98E-11	0,36706575	0,981	0,894	1,65E-06	RPL29	UPP	IDA_macrophage_vs_M2
5,07E-11	0,39067653	0,66	0,43	1,68E-06	ARL8B	UPP	IDA_macrophage_vs_M2
6,79E-11	0,27811614	0,902	0,695	2,25E-06	MYL12A	UPP	IDA_macrophage_vs_M2
6,82E-11	0,29855131	0,207	0,05	2,26E-06	SNX20	UPP	IDA_macrophage_vs_M2
7,10E-11	0,3167381	0,147	0,016	2,35E-06	CD1C	UPP	IDA_macrophage_vs_M2
7,44E-11	0,34739955	0,802	0,623	2,47E-06	LAP3	UPP	IDA_macrophage_vs_M2
7,52E-11	0,29932656	0,856	0,621	2,49E-06	HCST	UPP	IDA_macrophage_vs_M2
7,60E-11	0,53603174	0,603	0,379	2,52E-06	SLAMF7	UPP	IDA_macrophage_vs_M2
8,03E-11	0,30826909	0,764	0,528	2,66E-06	EFHD2	UPP	IDA_macrophage_vs_M2
8,65E-11	0,44429597	0,804	0,631	2,87E-06	JAML	UPP	IDA_macrophage_vs_M2

8,67E-11	0,52013743	0,764	0,48	2,87E-06	ATF3	UPP	IDA_macrophage_vs_M2
9,21E-11	0,4126233	0,261	0,088	3,05E-06	STARD4	UPP	IDA_macrophage_vs_M2
9,29E-11	0,33217886	0,734	0,509	3,08E-06	PRR13	UPP	IDA_macrophage_vs_M2
9,49E-11	0,52119507	0,378	0,178	3,14E-06	CCDC107	UPP	IDA_macrophage_vs_M2
9,86E-11	0,31521178	0,707	0,48	3,27E-06	MTPN	UPP	IDA_macrophage_vs_M2
1,06E-10	0,26396141	0,666	0,456	3,50E-06	PSMA4	UPP	IDA_macrophage_vs_M2
1,11E-10	0,74740031	0,296	0,114	3,66E-06	PHLDA2	UPP	IDA_macrophage_vs_M2
1,20E-10	0,59122731	0,541	0,342	3,97E-06	FNIP2	UPP	IDA_macrophage_vs_M2
1,30E-10	0,49798628	0,429	0,241	4,29E-06	NUMB	UPP	IDA_macrophage_vs_M2
1,30E-10	0,31262229	0,174	0,032	4,32E-06	BCL11A	UPP	IDA_macrophage_vs_M2
1,33E-10	0,36706427	0,552	0,353	4,41E-06	RAB8A	UPP	IDA_macrophage_vs_M2
1,35E-10	0,36163833	0,978	0,897	4,46E-06	RPS3	UPP	IDA_macrophage_vs_M2
1,41E-10	0,34042225	0,255	0,085	4,67E-06	ITGAL	UPP	IDA_macrophage_vs_M2
1,42E-10	0,35099648	0,25	0,08	4,70E-06	IL3RA	UPP	IDA_macrophage_vs_M2
1,45E-10	0,5084642	0,245	0,08	4,80E-06	CDC42EP3	UPP	IDA_macrophage_vs_M2
1,47E-10	0,3547983	0,878	0,629	4,85E-06	ATP6V1G1	UPP	IDA_macrophage_vs_M2
1,50E-10	0,5321761	0,726	0,488	4,97E-06	TNFAIP3	UPP	IDA_macrophage_vs_M2
1,62E-10	0,33098151	0,394	0,199	5,37E-06	APBB1IP	UPP	IDA_macrophage_vs_M2
1,63E-10	0,51481739	0,84	0,623	5,41E-06	TNFRSF1B	UPP	IDA_macrophage_vs_M2
1,64E-10	0,38780129	0,769	0,565	5,43E-06	TNFSF13B	UPP	IDA_macrophage_vs_M2
1,68E-10	0,41225537	0,31	0,122	5,57E-06	CHSY1	UPP	IDA_macrophage_vs_M2
1,77E-10	0,50059602	0,473	0,273	5,86E-06	C9orf72	UPP	IDA_macrophage_vs_M2
1,79E-10	0,36276223	0,541	0,321	5,92E-06	SELENOW	UPP	IDA_macrophage_vs_M2
2,26E-10	0,31659954	0,867	0,645	7,47E-06	CTSH	UPP	IDA_macrophage_vs_M2
2,36E-10	0,30900652	0,174	0,034	7,82E-06	TRAF3IP3	UPP	IDA_macrophage_vs_M2
2,43E-10	0,26538559	0,802	0,602	8,06E-06	FAM49B	UPP	IDA_macrophage_vs_M2
2,72E-10	0,40054829	0,856	0,639	9,03E-06	YWHAZ	UPP	IDA_macrophage_vs_M2
2,77E-10	0,36411306	0,459	0,26	9,19E-06	FLOT1	UPP	IDA_macrophage_vs_M2
2,89E-10	0,40830898	0,266	0,095	9,58E-06	TESC	UPP	IDA_macrophage_vs_M2
2,97E-10	0,50234616	0,389	0,196	9,84E-06	GPR65	UPP	IDA_macrophage_vs_M2
3,01E-10	0,28795752	0,234	0,069	9,96E-06	SLC36A4	UPP	IDA_macrophage_vs_M2
3,07E-10	0,37891879	0,677	0,475	1,02E-05	CASP1	UPP	IDA_macrophage_vs_M2
3,21E-10	0,68418321	0,598	0,355	1,06E-05	PTGS2	UPP	IDA_macrophage_vs_M2
3,28E-10	0,4227219	0,269	0,093	1,09E-05	IFI6	UPP	IDA_macrophage_vs_M2
3,62E-10	0,40064791	1	0,955	1,20E-05	SAT1	UPP	IDA_macrophage_vs_M2
3,83E-10	0,41698118	0,41	0,215	1,27E-05	TAP1	UPP	IDA_macrophage_vs_M2
3,91E-10	0,37031827	0,788	0,589	1,29E-05	SERP1	UPP	IDA_macrophage_vs_M2
3,94E-10	0,29744237	0,582	0,382	1,30E-05	EIF3M	UPP	IDA_macrophage_vs_M2
4,37E-10	0,40912808	0,522	0,318	1,45E-05	SH3BP2	UPP	IDA_macrophage_vs_M2
4,51E-10	0,38108481	0,334	0,151	1,49E-05	SCLT1	UPP	IDA_macrophage_vs_M2
4,56E-10	0,30369963	0,429	0,218	1,51E-05	LY6E	UPP	IDA_macrophage_vs_M2
4,79E-10	0,45310606	0,288	0,111	1,59E-05	HCAR2	UPP	IDA_macrophage_vs_M2
4,95E-10	0,39715557	0,785	0,517	1,64E-05	INSIG1	UPP	IDA_macrophage_vs_M2
5,43E-10	0,36583259	0,611	0,398	1,80E-05	RNF7	UPP	IDA_macrophage_vs_M2
5,54E-10	0,36435072	0,965	0,878	1,84E-05	ACTG1	UPP	IDA_macrophage_vs_M2
5,57E-10	0,38613036	0,546	0,358	1,84E-05	PILRA	UPP	IDA_macrophage_vs_M2

5,74E-10	0,28491018	1	0,96	1,90E-05	RPL39	UPP	IDA_macrophage_vs_M2
5,79E-10	0,32994312	0,72	0,499	1,92E-05	APRT	UPP	IDA_macrophage_vs_M2
5,97E-10	0,32823049	0,864	0,658	1,98E-05	MYL12B	UPP	IDA_macrophage_vs_M2
6,00E-10	0,72649001	0,715	0,493	1,99E-05	IER2	UPP	IDA_macrophage_vs_M2
6,05E-10	0,34634405	0,31	0,133	2,00E-05	ZDHHC20	UPP	IDA_macrophage_vs_M2
6,29E-10	0,40016713	0,329	0,149	2,08E-05	RAB5IF	UPP	IDA_macrophage_vs_M2
6,76E-10	0,42436103	0,31	0,127	2,24E-05	IL4I1	UPP	IDA_macrophage_vs_M2
6,85E-10	0,45363216	0,552	0,329	2,27E-05	PER1	UPP	IDA_macrophage_vs_M2
7,76E-10	0,49706096	0,302	0,127	2,57E-05	AC016831.1	UPP	IDA_macrophage_vs_M2
8,34E-10	0,32963119	0,636	0,406	2,76E-05	TRIR	UPP	IDA_macrophage_vs_M2
8,37E-10	0,26900315	0,989	0,952	2,77E-05	RPL19	UPP	IDA_macrophage_vs_M2
8,98E-10	0,3323668	0,91	0,708	2,98E-05	BTF3	UPP	IDA_macrophage_vs_M2
9,31E-10	0,34130933	0,636	0,424	3,09E-05	SNHG8	UPP	IDA_macrophage_vs_M2
9,40E-10	0,26956619	0,576	0,345	3,11E-05	UBE2L6	UPP	IDA_macrophage_vs_M2
9,51E-10	0,29626428	0,932	0,743	3,15E-05	EEF1D	UPP	IDA_macrophage_vs_M2
9,68E-10	0,31998573	0,402	0,199	3,21E-05	FCGR1A	UPP	IDA_macrophage_vs_M2
9,88E-10	0,4223921	0,323	0,146	3,27E-05	AVPI1	UPP	IDA_macrophage_vs_M2
9,89E-10	0,35290121	0,668	0,451	3,28E-05	ZNF706	UPP	IDA_macrophage_vs_M2
1,15E-09	0,27295051	0,579	0,347	3,82E-05	HLA-F	UPP	IDA_macrophage_vs_M2
1,28E-09	0,28257599	0,101	0,003	4,25E-05	MARCO	UPP	IDA_macrophage_vs_M2
1,42E-09	0,30222599	0,788	0,536	4,70E-05	SFPQ	UPP	IDA_macrophage_vs_M2
1,44E-09	0,33468967	0,978	0,91	4,77E-05	RPL7A	UPP	IDA_macrophage_vs_M2
1,74E-09	0,48326603	0,427	0,233	5,76E-05	TENT4B	UPP	IDA_macrophage_vs_M2
1,88E-09	0,26646916	0,139	0,021	6,22E-05	ICAM3	UPP	IDA_macrophage_vs_M2
1,91E-09	0,41678709	0,726	0,491	6,33E-05	CCNL1	UPP	IDA_macrophage_vs_M2
1,97E-09	0,36377253	0,304	0,13	6,52E-05	LDLRAD4	UPP	IDA_macrophage_vs_M2
2,00E-09	0,30286747	0,88	0,711	6,62E-05	CLIC1	UPP	IDA_macrophage_vs_M2
2,46E-09	0,42177645	0,772	0,576	8,15E-05	NINJ1	UPP	IDA_macrophage_vs_M2
2,47E-09	0,26654031	0,978	0,92	8,18E-05	RPS15A	UPP	IDA_macrophage_vs_M2
2,51E-09	0,26775923	0,438	0,241	8,30E-05	CAMTA1	UPP	IDA_macrophage_vs_M2
2,52E-09	0,37208962	0,53	0,326	8,35E-05	ATP5MC1	UPP	IDA_macrophage_vs_M2
2,53E-09	0,39934423	0,505	0,302	8,37E-05	CKLF	UPP	IDA_macrophage_vs_M2
2,55E-09	0,3168412	0,386	0,188	8,44E-05	TBCB	UPP	IDA_macrophage_vs_M2
2,71E-09	0,37704761	0,413	0,223	8,97E-05	HMGA1	UPP	IDA_macrophage_vs_M2
2,76E-09	0,29302963	0,546	0,345	9,14E-05	AURKAIP1	UPP	IDA_macrophage_vs_M2
2,80E-09	0,44612943	0,299	0,13	9,28E-05	NDRG1	UPP	IDA_macrophage_vs_M2
2,81E-09	0,46875707	0,582	0,387	9,31E-05	RSRP1	UPP	IDA_macrophage_vs_M2
2,96E-09	0,33436449	0,16	0,034	9,81E-05	ASGR2	UPP	IDA_macrophage_vs_M2
2,98E-09	0,26729248	0,386	0,196	9,86E-05	AIMP1	UPP	IDA_macrophage_vs_M2
3,07E-09	0,41952567	0,611	0,411	0,0001	XBP1	UPP	IDA_macrophage_vs_M2
3,22E-09	0,30308772	0,997	0,958	0,00011	RPS12	UPP	IDA_macrophage_vs_M2
3,24E-09	0,41140956	0,351	0,18	0,00011	TLE3	UPP	IDA_macrophage_vs_M2
3,28E-09	0,34028881	0,345	0,156	0,00011	NXT1	UPP	IDA_macrophage_vs_M2
3,37E-09	0,27080415	0,981	0,928	0,00011	RPS11	UPP	IDA_macrophage_vs_M2
3,45E-09	0,33530502	0,652	0,435	0,00011	VPS35	UPP	IDA_macrophage_vs_M2
3,48E-09	0,43981144	0,492	0,305	0,00012	PTP4A1	UPP	IDA_macrophage_vs_M2

3,52E-09	0,32582895	0,454	0,263	0,00012	RGS19	UPP	IDA_macrophage_vs_M2
3,77E-09	0,33657145	0,348	0,162	0,00012	METTL26	UPP	IDA_macrophage_vs_M2
3,78E-09	0,34862397	0,31	0,138	0,00013	CREB5	UPP	IDA_macrophage_vs_M2
3,88E-09	0,3381695	0,329	0,156	0,00013	ACAA1	UPP	IDA_macrophage_vs_M2
4,10E-09	0,31641005	0,989	0,952	0,00014	RPS13	UPP	IDA_macrophage_vs_M2
4,58E-09	0,55648425	0,682	0,496	0,00015	TSC22D3	UPP	IDA_macrophage_vs_M2
4,65E-09	0,31264319	0,514	0,297	0,00015	MALT1	UPP	IDA_macrophage_vs_M2
4,92E-09	0,26961326	0,359	0,178	0,00016	MBOAT7	UPP	IDA_macrophage_vs_M2
5,27E-09	0,33190423	0,272	0,111	0,00017	C19orf38	UPP	IDA_macrophage_vs_M2
5,58E-09	0,38773473	0,59	0,379	0,00018	GPBP1	UPP	IDA_macrophage_vs_M2
5,66E-09	0,3090553	0,288	0,122	0,00019	MAD2L2	UPP	IDA_macrophage_vs_M2
5,68E-09	0,31807934	0,375	0,199	0,00019	CSF2RB	UPP	IDA_macrophage_vs_M2
5,86E-09	0,29317902	0,391	0,202	0,00019	SORL1	UPP	IDA_macrophage_vs_M2
6,05E-09	0,62921193	0,625	0,44	0,0002	IRF1	UPP	IDA_macrophage_vs_M2
6,16E-09	0,37150197	0,508	0,31	0,0002	UBE2R2	UPP	IDA_macrophage_vs_M2
6,81E-09	0,37640896	0,188	0,053	0,00023	PDE4D	UPP	IDA_macrophage_vs_M2
7,04E-09	0,56213777	0,337	0,164	0,00023	TWISTNB	UPP	IDA_macrophage_vs_M2
7,07E-09	0,55780042	0,239	0,09	0,00023	HCAR3	UPP	IDA_macrophage_vs_M2
7,60E-09	0,27718434	0,734	0,528	0,00025	MSN	UPP	IDA_macrophage_vs_M2
7,63E-09	0,29889783	0,391	0,204	0,00025	sep-06	UPP	IDA_macrophage_vs_M2
7,78E-09	0,53369358	0,766	0,573	0,00026	CEBPB	UPP	IDA_macrophage_vs_M2
8,68E-09	0,36571986	0,94	0,671	0,00029	PNRC1	UPP	IDA_macrophage_vs_M2
8,99E-09	0,33937837	0,571	0,393	0,0003	PIK3AP1	UPP	IDA_macrophage_vs_M2
9,50E-09	0,33457605	0,315	0,146	0,00031	UBE2S	UPP	IDA_macrophage_vs_M2
9,87E-09	0,27664548	0,16	0,037	0,00033	PGS1	UPP	IDA_macrophage_vs_M2
9,88E-09	0,27140304	0,103	0,008	0,00033	AC015912.3	UPP	IDA_macrophage_vs_M2
1,01E-08	0,42493672	0,372	0,191	0,00033	SLC7A5	UPP	IDA_macrophage_vs_M2
1,02E-08	0,38082699	0,584	0,395	0,00034	SLC43A2	UPP	IDA_macrophage_vs_M2
1,07E-08	0,37422949	0,296	0,13	0,00035	C15orf39	UPP	IDA_macrophage_vs_M2
1,08E-08	0,34296563	0,198	0,061	0,00036	CD300LB	UPP	IDA_macrophage_vs_M2
1,10E-08	0,42760858	0,753	0,538	0,00036	SRSF7	UPP	IDA_macrophage_vs_M2
1,13E-08	0,44555461	0,34	0,172	0,00037	B4GALT5	UPP	IDA_macrophage_vs_M2
1,16E-08	0,35255794	0,478	0,276	0,00039	JOSD1	UPP	IDA_macrophage_vs_M2
1,18E-08	0,32775928	0,473	0,289	0,00039	ILF2	UPP	IDA_macrophage_vs_M2
1,26E-08	0,2667302	0,848	0,623	0,00042	H2AFY	UPP	IDA_macrophage_vs_M2
1,31E-08	0,39089952	0,557	0,366	0,00043	KLF10	UPP	IDA_macrophage_vs_M2
1,38E-08	0,41554648	0,527	0,342	0,00046	CD37	UPP	IDA_macrophage_vs_M2
1,46E-08	0,38042595	0,207	0,069	0,00048	TMEM170B	UPP	IDA_macrophage_vs_M2
1,46E-08	0,38427234	0,236	0,09	0,00048	RHOH	UPP	IDA_macrophage_vs_M2
1,46E-08	0,27167572	0,601	0,395	0,00048	NDUFA6	UPP	IDA_macrophage_vs_M2
1,47E-08	0,26733219	0,418	0,231	0,00049	CALCOCO2	UPP	IDA_macrophage_vs_M2
1,69E-08	0,35543691	0,519	0,316	0,00056	MANF	UPP	IDA_macrophage_vs_M2
1,73E-08	0,2661797	0,209	0,072	0,00057	EEF1E1	UPP	IDA_macrophage_vs_M2
1,94E-08	0,26889284	0,122	0,019	0,00064	CRADD	UPP	IDA_macrophage_vs_M2
1,98E-08	0,33954635	0,375	0,204	0,00065	OXSR1	UPP	IDA_macrophage_vs_M2
2,06E-08	0,38805163	0,359	0,196	0,00068	PPP2R2A	UPP	IDA_macrophage_vs_M2

2,11E-08	0,56535605	0,111	0,013	0,0007	TNFAIP6	UPP	IDA_macrophage_vs_M2
2,14E-08	0,33123248	0,53	0,324	0,00071	SDF2L1	UPP	IDA_macrophage_vs_M2
2,20E-08	0,39392455	0,747	0,523	0,00073	GADD45B	UPP	IDA_macrophage_vs_M2
2,36E-08	0,28850001	0,973	0,926	0,00078	RPL26	UPP	IDA_macrophage_vs_M2
2,44E-08	0,81306342	0,878	0,695	0,00081	HSPA5	UPP	IDA_macrophage_vs_M2
2,58E-08	0,42301382	0,543	0,337	0,00086	ARL4A	UPP	IDA_macrophage_vs_M2
2,65E-08	0,36214984	0,37	0,199	0,00088	ago-02	UPP	IDA_macrophage_vs_M2
3,32E-08	0,44770672	0,457	0,273	0,0011	RAB11FIP1	UPP	IDA_macrophage_vs_M2
4,57E-08	0,35259335	0,299	0,146	0,00151	PTK2B	UPP	IDA_macrophage_vs_M2
4,65E-08	0,26537721	0,394	0,215	0,00154	PHF20L1	UPP	IDA_macrophage_vs_M2
4,70E-08	0,28727203	0,614	0,422	0,00156	RSRC2	UPP	IDA_macrophage_vs_M2
4,72E-08	0,28303151	0,201	0,066	0,00157	GPR157	UPP	IDA_macrophage_vs_M2
4,85E-08	0,31704282	0,908	0,788	0,00161	RPS5	UPP	IDA_macrophage_vs_M2
5,26E-08	0,27072745	0,976	0,912	0,00174	RPL35A	UPP	IDA_macrophage_vs_M2
5,35E-08	0,32907896	0,296	0,143	0,00177	DAPP1	UPP	IDA_macrophage_vs_M2
5,73E-08	0,3332217	0,179	0,056	0,0019	SLC2A6	UPP	IDA_macrophage_vs_M2
5,86E-08	0,4055794	0,405	0,231	0,00194	CDKN1B	UPP	IDA_macrophage_vs_M2
6,00E-08	0,35923943	0,59	0,385	0,00199	MAP3K8	UPP	IDA_macrophage_vs_M2
6,36E-08	0,29821866	0,894	0,687	0,00211	PCBP1	UPP	IDA_macrophage_vs_M2
6,38E-08	0,43084877	0,726	0,544	0,00211	CTNNB1	UPP	IDA_macrophage_vs_M2
6,42E-08	0,29629589	0,677	0,485	0,00213	EIF4A1	UPP	IDA_macrophage_vs_M2
6,49E-08	0,27420382	0,56	0,395	0,00215	MRPL52	UPP	IDA_macrophage_vs_M2
6,85E-08	0,34517219	0,391	0,228	0,00227	POLR2G	UPP	IDA_macrophage_vs_M2
7,02E-08	0,42073364	0,916	0,753	0,00232	LITAF	UPP	IDA_macrophage_vs_M2
7,12E-08	0,28551795	0,353	0,186	0,00236	RELT	UPP	IDA_macrophage_vs_M2
7,23E-08	0,29997567	0,312	0,156	0,0024	CENPX	UPP	IDA_macrophage_vs_M2
7,57E-08	0,28865556	0,562	0,382	0,00251	RPS4Y1	UPP	IDA_macrophage_vs_M2
7,69E-08	0,30672338	0,397	0,225	0,00255	ANKRD44	UPP	IDA_macrophage_vs_M2
8,00E-08	0,35213273	0,592	0,416	0,00265	CELF2	UPP	IDA_macrophage_vs_M2
8,26E-08	0,34386676	0,63	0,422	0,00274	SKIL	UPP	IDA_macrophage_vs_M2
8,81E-08	0,30268978	0,603	0,435	0,00292	EMILIN2	UPP	IDA_macrophage_vs_M2
8,90E-08	0,28371476	0,546	0,363	0,00295	PTEN	UPP	IDA_macrophage_vs_M2
9,06E-08	0,38753602	0,367	0,215	0,003	FAM45A	UPP	IDA_macrophage_vs_M2
9,55E-08	0,49828016	0,16	0,045	0,00316	MIR155HG	UPP	IDA_macrophage_vs_M2
9,86E-08	0,32894491	0,133	0,029	0,00327	AC025164.1	UPP	IDA_macrophage_vs_M2
1,05E-07	0,26442285	0,508	0,318	0,00348	UBE2D2	UPP	IDA_macrophage_vs_M2
1,09E-07	0,39154445	0,405	0,247	0,0036	TRIM38	UPP	IDA_macrophage_vs_M2
1,21E-07	0,31020033	0,736	0,538	0,00401	GNA13	UPP	IDA_macrophage_vs_M2
1,24E-07	0,28509045	0,446	0,276	0,0041	CCNH	UPP	IDA_macrophage_vs_M2
1,32E-07	0,30991808	0,239	0,103	0,00438	MEFV	UPP	IDA_macrophage_vs_M2
1,33E-07	0,3661218	0,353	0,191	0,0044	EAF1	UPP	IDA_macrophage_vs_M2
1,35E-07	0,28582144	0,16	0,045	0,00447	LIMK2	UPP	IDA_macrophage_vs_M2
1,37E-07	0,27607335	0,696	0,493	0,00453	THEMIS2	UPP	IDA_macrophage_vs_M2
1,46E-07	0,26317534	0,418	0,241	0,00484	MRPL21	UPP	IDA_macrophage_vs_M2
1,52E-07	0,26370453	0,269	0,125	0,00504	ADSS	UPP	IDA_macrophage_vs_M2
1,55E-07	0,27300159	0,364	0,196	0,00513	DPH3	UPP	IDA_macrophage_vs_M2

1,70E-07	0,29624014	0,212	0,082	0,00563	TNIP2	UPP	IDA_macrophage_vs_M2
1,72E-07	0,28936285	0,152	0,042	0,00569	CACNA2D3	UPP	IDA_macrophage_vs_M2
1,84E-07	0,34128171	0,383	0,225	0,00609	ERO1A	UPP	IDA_macrophage_vs_M2
1,87E-07	0,26779739	0,285	0,141	0,0062	CARD19	UPP	IDA_macrophage_vs_M2
1,97E-07	0,35257839	0,212	0,08	0,00652	TRAF1	UPP	IDA_macrophage_vs_M2
2,03E-07	0,28704011	0,701	0,509	0,00671	CSNK1A1	UPP	IDA_macrophage_vs_M2
2,15E-07	0,31658103	0,391	0,215	0,00711	KRAS	UPP	IDA_macrophage_vs_M2
2,19E-07	0,32874642	0,679	0,488	0,00726	CD86	UPP	IDA_macrophage_vs_M2
2,23E-07	0,26894924	0,307	0,151	0,00738	SIPA1L1	UPP	IDA_macrophage_vs_M2
2,23E-07	0,29647651	0,253	0,114	0,00739	CAPN2	UPP	IDA_macrophage_vs_M2
2,26E-07	0,2785323	0,326	0,164	0,00749	MIS18BP1	UPP	IDA_macrophage_vs_M2
2,37E-07	0,44646718	0,288	0,143	0,00786	CXorf21	UPP	IDA_macrophage_vs_M2
2,50E-07	0,29883209	0,622	0,451	0,0083	RBM25	UPP	IDA_macrophage_vs_M2
2,51E-07	0,2998707	0,484	0,308	0,00833	RNMT	UPP	IDA_macrophage_vs_M2
2,75E-07	0,35279957	0,41	0,257	0,00911	ARID5A	UPP	IDA_macrophage_vs_M2
2,85E-07	0,28401559	0,378	0,223	0,00945	KARS	UPP	IDA_macrophage_vs_M2
2,91E-07	0,33967848	0,492	0,308	0,00963	SNX10	UPP	IDA_macrophage_vs_M2
3,16E-07	0,36385204	0,505	0,326	0,01047	TGIF1	UPP	IDA_macrophage_vs_M2
3,78E-07	0,29067809	0,546	0,382	0,01254	BAX	UPP	IDA_macrophage_vs_M2
3,84E-07	0,32649347	0,361	0,21	0,01272	JARID2	UPP	IDA_macrophage_vs_M2
4,14E-07	0,27236765	0,37	0,202	0,01372	TRABD	UPP	IDA_macrophage_vs_M2
4,14E-07	0,30144652	0,565	0,401	0,01372	PNRC2	UPP	IDA_macrophage_vs_M2
4,23E-07	0,39734149	0,239	0,109	0,01403	HK2	UPP	IDA_macrophage_vs_M2
4,30E-07	0,48015496	0,571	0,411	0,01424	IVNS1ABP	UPP	IDA_macrophage_vs_M2
4,66E-07	0,32239028	0,462	0,305	0,01542	PTPN12	UPP	IDA_macrophage_vs_M2
4,93E-07	0,28220941	0,337	0,186	0,01633	PDCL3	UPP	IDA_macrophage_vs_M2
5,28E-07	0,35288764	0,37	0,215	0,0175	DNAJB11	UPP	IDA_macrophage_vs_M2
5,33E-07	0,33239852	0,734	0,557	0,01766	EIF5	UPP	IDA_macrophage_vs_M2
5,60E-07	0,28643295	0,848	0,658	0,01856	HNRNPK	UPP	IDA_macrophage_vs_M2
5,63E-07	0,29597449	0,516	0,345	0,01864	UQCRFS1	UPP	IDA_macrophage_vs_M2
5,81E-07	0,3892636	0,503	0,334	0,01924	ATF5	UPP	IDA_macrophage_vs_M2
5,87E-07	0,35671884	0,486	0,31	0,01945	SERTAD1	UPP	IDA_macrophage_vs_M2
6,26E-07	0,30597613	0,554	0,379	0,02073	MTHFD2	UPP	IDA_macrophage_vs_M2
6,40E-07	0,2721704	0,845	0,666	0,02121	CCNI	UPP	IDA_macrophage_vs_M2
7,10E-07	0,28825002	0,413	0,249	0,02352	EMD	UPP	IDA_macrophage_vs_M2
7,27E-07	0,30409326	0,557	0,395	0,02407	COPA	UPP	IDA_macrophage_vs_M2
7,32E-07	0,26945511	0,992	0,915	0,02424	EIF1	UPP	IDA_macrophage_vs_M2
7,55E-07	0,34611833	0,375	0,218	0,02502	TUT7	UPP	IDA_macrophage_vs_M2
7,79E-07	0,45983271	0,28	0,141	0,02579	ACSL1	UPP	IDA_macrophage_vs_M2
7,89E-07	0,4640036	0,568	0,403	0,02614	TNFAIP8	UPP	IDA_macrophage_vs_M2
7,96E-07	0,36241052	0,109	0,021	0,02636	TNIP3	UPP	IDA_macrophage_vs_M2
8,32E-07	0,2765363	0,413	0,249	0,02757	RIOK3	UPP	IDA_macrophage_vs_M2
8,46E-07	0,28844702	0,109	0,021	0,02803	CDA	UPP	IDA_macrophage_vs_M2
8,62E-07	0,27123605	0,207	0,085	0,02855	PLP2	UPP	IDA_macrophage_vs_M2
8,88E-07	0,2687864	0,533	0,358	0,02941	EWSR1	UPP	IDA_macrophage_vs_M2
9,21E-07	0,3090567	0,234	0,109	0,0305	MTF1	UPP	IDA_macrophage_vs_M2

9,34E-07	1,60522984	0,147	0,045	0,03094	CCL20	UPP	IDA_macrophage_vs_M2
9,66E-07	0,27679608	0,951	0,83	0,03201	ARPC2	UPP	IDA_macrophage_vs_M2
9,68E-07	0,2657518	0,353	0,204	0,03206	PAG1	UPP	IDA_macrophage_vs_M2
9,84E-07	0,26983034	0,522	0,371	0,03259	SLC16A3	UPP	IDA_macrophage_vs_M2
9,90E-07	0,27193149	0,59	0,416	0,03279	PLXDC2	UPP	IDA_macrophage_vs_M2
1,03E-06	0,27812173	0,459	0,292	0,03428	CSNK1D	UPP	IDA_macrophage_vs_M2
1,08E-06	0,30950032	0,929	0,809	0,03583	RPSA	UPP	IDA_macrophage_vs_M2
1,19E-06	0,3526323	0,193	0,077	0,03946	ANKRD28	UPP	IDA_macrophage_vs_M2
1,23E-06	0,34592266	0,264	0,133	0,04061	ITGA5	UPP	IDA_macrophage_vs_M2
1,26E-06	0,5020871	0,533	0,366	0,04158	EIF4A3	UPP	IDA_macrophage_vs_M2
1,35E-06	0,69316739	0,364	0,207	0,04467	CCL3L1	UPP	IDA_macrophage_vs_M2
1,47E-06	0,36004054	0,397	0,252	0,0487	ACTN1	UPP	IDA_macrophage_vs_M2
2,24E-126	-4,3345532	0,147	0,997	########	SELENOP	DWW	IDA_macrophage_vs_M2
1,13E-88	-3,3175817	0,375	0,923	3,73E-84	SLC40A1	DWW	IDA_macrophage_vs_M2
2,90E-86	-3,5311283	0,19	0,851	9,62E-82	FUCA1	DWW	IDA_macrophage_vs_M2
8,93E-81	-3,005124	0,427	0,894	2,96E-76	LGMN	DWW	IDA_macrophage_vs_M2
1,26E-78	-2,6146427	0,133	0,782	4,16E-74	A2M	DWW	IDA_macrophage_vs_M2
2,23E-78	-1,8885115	0,595	0,995	7,37E-74	C1QA	DWW	IDA_macrophage_vs_M2
3,28E-75	-2,1740624	0,068	0,719	1,09E-70	CD163L1	DWW	IDA_macrophage_vs_M2
3,50E-74	-2,0573114	0,557	0,997	1,16E-69	C1QB	DWW	IDA_macrophage_vs_M2
1,05E-73	-3,9014886	0,03	0,668	3,48E-69	RNASE1	DWW	IDA_macrophage_vs_M2
1,86E-72	-2,5271141	0,293	0,822	6,16E-68	DAB2	DWW	IDA_macrophage_vs_M2
1,21E-67	-1,9747873	0,535	0,958	4,01E-63	C1QC	DWW	IDA_macrophage_vs_M2
2,28E-64	-2,0935611	0,068	0,655	7,56E-60	IGF1	DWW	IDA_macrophage_vs_M2
2,41E-58	-2,5248877	0,136	0,692	7,97E-54	GPNMB	DWW	IDA_macrophage_vs_M2
3,78E-58	-1,5808912	0,829	0,923	1,25E-53	FCGRT	DWW	IDA_macrophage_vs_M2
2,47E-57	-2,4648025	0,193	0,761	8,19E-53	DNASE1L3	DWW	IDA_macrophage_vs_M2
1,44E-56	-1,7640614	0,864	0,973	4,79E-52	CTSC	DWW	IDA_macrophage_vs_M2
6,88E-55	-1,8549506	0,073	0,605	2,28E-50	LILRB5	DWW	IDA_macrophage_vs_M2
1,90E-53	-2,3140976	0,122	0,637	6,30E-49	FOLR2	DWW	IDA_macrophage_vs_M2
2,55E-53	-2,0417188	0,291	0,751	8,44E-49	MAF	DWW	IDA_macrophage_vs_M2
2,60E-52	-1,7816081	0,041	0,552	8,63E-48	PLTP	DWW	IDA_macrophage_vs_M2
3,99E-51	-2,9919836	0,12	0,615	1,32E-46	F13A1	DWW	IDA_macrophage_vs_M2
1,32E-50	-2,0279357	0,495	0,806	4,38E-46	STAB1	DWW	IDA_macrophage_vs_M2
2,26E-49	-1,7070402	0,81	0,936	7,49E-45	CTSD	DWW	IDA_macrophage_vs_M2
4,95E-47	-1,1617544	0,965	0,984	1,64E-42	PSAP	DWW	IDA_macrophage_vs_M2
7,23E-47	-0,8506196	1	0,995	2,39E-42	CD74	DWW	IDA_macrophage_vs_M2
2,26E-44	-1,6150514	0,269	0,695	7,48E-40	SLCO2B1	DWW	IDA_macrophage_vs_M2
1,01E-43	-1,5303563	0,117	0,581	3,36E-39	GATM	DWW	IDA_macrophage_vs_M2
2,22E-42	-1,7684685	0,486	0,788	7,35E-38	LIPA	DWW	IDA_macrophage_vs_M2
6,97E-40	-1,1740447	0,038	0,456	2,31E-35	SLC7A8	DWW	IDA_macrophage_vs_M2
2,08E-39	-1,5973738	0,326	0,706	6,88E-35	MS4A4A	DWW	IDA_macrophage_vs_M2
2,90E-39	-1,3675213	0,878	0,918	9,60E-35	CTSZ	DWW	IDA_macrophage_vs_M2
8,33E-38	-1,0611408	0,929	0,955	2,76E-33	ITM2B	DWW	IDA_macrophage_vs_M2
8,03E-37	-1,7602279	0,31	0,687	2,66E-32	CD163	DWW	IDA_macrophage_vs_M2
1,66E-36	-0,7478875	1	0,997	5,49E-32	FTL	DWW	IDA_macrophage_vs_M2

1,08E-35	-1,2806967	0,03	0,411	3,58E-31	CXCL12	DWW IDA_macrophage_vs_M2
1,12E-34	-1,4574981	0,144	0,546	3,71E-30	NRP1	DWW IDA_macrophage_vs_M2
2,01E-33	-1,2749981	0,12	0,515	6,65E-29	GPR34	DWW IDA_macrophage_vs_M2
3,56E-33	-1,5848461	0,397	0,708	1,18E-28	PLD3	DWW IDA_macrophage_vs_M2
8,05E-33	-1,1362316	0,87	0,907	2,67E-28	GRN	DWW IDA_macrophage_vs_M2
3,70E-32	-3,614641	0,016	0,353	1,22E-27	CCL18	DWW IDA_macrophage_vs_M2
1,92E-31	-1,0040761	0,845	0,907	6,35E-27	MS4A6A	DWW IDA_macrophage_vs_M2
4,60E-31	-1,2172042	0,234	0,605	1,52E-26	AXL	DWW IDA_macrophage_vs_M2
5,43E-31	-2,2752627	0,087	0,472	1,80E-26	APOE	DWW IDA_macrophage_vs_M2
1,70E-30	-1,37597	0,255	0,623	5,65E-26	CD209	DWW IDA_macrophage_vs_M2
2,25E-30	-0,8389149	0,043	0,387	7,45E-26	ITGB5	DWW IDA_macrophage_vs_M2
9,67E-30	-1,4573294	0,204	0,605	3,20E-25	APOC1	DWW IDA_macrophage_vs_M2
1,41E-29	-0,7638242	1	0,997	4,68E-25	TMSB4X	DWW IDA_macrophage_vs_M2
3,34E-28	-1,2027145	0,109	0,451	1,11E-23	STMN1	DWW IDA_macrophage_vs_M2
3,31E-27	-1,3733711	0,122	0,456	1,10E-22	SERPINF1	DWW IDA_macrophage_vs_M2
3,33E-27	-1,20452	0,467	0,737	1,10E-22	RNASE6	DWW IDA_macrophage_vs_M2
2,81E-26	-1,1651041	0,291	0,613	9,29E-22	LACC1	DWW IDA_macrophage_vs_M2
3,24E-26	-0,7469152	0,005	0,279	1,07E-21	TMEM37	DWW IDA_macrophage_vs_M2
4,36E-26	-0,8776396	0,019	0,302	1,44E-21	QPRT	DWW IDA_macrophage_vs_M2
5,23E-26	-1,4469835	0,158	0,496	1,73E-21	C2	DWW IDA_macrophage_vs_M2
5,07E-25	-1,0966417	0,166	0,499	1,68E-20	WWP1	DWW IDA_macrophage_vs_M2
8,85E-25	-1,0687403	0,859	0,875	2,93E-20	MS4A7	DWW IDA_macrophage_vs_M2
1,19E-24	-1,11702	0,122	0,44	3,93E-20	CD59	DWW IDA_macrophage_vs_M2
1,39E-24	-1,3025013	0,5	0,703	4,59E-20	CREG1	DWW IDA_macrophage_vs_M2
1,48E-24	-0,7282754	0,054	0,358	4,92E-20	FRMD4A	DWW IDA_macrophage_vs_M2
4,20E-24	-0,858773	0,06	0,358	1,39E-19	ME1	DWW IDA_macrophage_vs_M2
4,21E-24	-0,8447467	0,962	0,955	1,39E-19	CTSB	DWW IDA_macrophage_vs_M2
1,22E-23	-1,5294806	0,147	0,459	4,04E-19	ADAMDEC1	DWW IDA_macrophage_vs_M2
5,26E-23	-0,746361	0,954	0,928	1,74E-18	NPC2	DWW IDA_macrophage_vs_M2
1,05E-22	-1,2511508	0,579	0,782	3,49E-18	ACP5	DWW IDA_macrophage_vs_M2
1,24E-22	-1,0586528	0,163	0,483	4,11E-18	SIGLEC1	DWW IDA_macrophage_vs_M2
1,68E-22	-0,8458978	0,057	0,34	5,57E-18	SCD	DWW IDA_macrophage_vs_M2
3,12E-22	-0,9452306	0,024	0,279	1,03E-17	CCND1	DWW IDA_macrophage_vs_M2
5,63E-22	-1,0702181	0,307	0,573	1,86E-17	AKR1B1	DWW IDA_macrophage_vs_M2
6,89E-22	-0,9061154	0,114	0,408	2,28E-17	ANKH	DWW IDA_macrophage_vs_M2
7,46E-22	-0,983974	0,821	0,875	2,47E-17	PRDX1	DWW IDA_macrophage_vs_M2
2,40E-21	-1,0613054	0,144	0,443	7,95E-17	ALDH1A1	DWW IDA_macrophage_vs_M2
5,22E-21	-1,0458204	0,03	0,276	1,73E-16	NUPR1	DWW IDA_macrophage_vs_M2
4,74E-20	-0,6970822	0,024	0,257	1,57E-15	WLS	DWW IDA_macrophage_vs_M2
9,03E-19	-0,8379497	0,158	0,435	2,99E-14	AP2A2	DWW IDA_macrophage_vs_M2
9,55E-19	-1,0822515	0,37	0,605	3,16E-14	AP1B1	DWW IDA_macrophage_vs_M2
1,37E-18	-1,0214582	0,166	0,448	4,55E-14	SDC3	DWW IDA_macrophage_vs_M2
3,04E-18	-0,8547748	0,082	0,334	1,01E-13	ITM2C	DWW IDA_macrophage_vs_M2
3,83E-18	-0,6344466	0,005	0,199	1,27E-13	HSD17B14	DWW IDA_macrophage_vs_M2
1,75E-17	-0,6865189	0,943	0,886	5,81E-13	CD68	DWW IDA_macrophage_vs_M2
1,91E-17	-0,8417815	0,16	0,443	6,33E-13	MSR1	DWW IDA_macrophage_vs_M2

3,84E-17	-0,9088568	0,22	0,477	1,27E-12	TCF4	DWWIDA_macrophage_vs_M2
6,07E-17	-0,6614249	0,06	0,289	2,01E-12	EPHX1	DWWIDA_macrophage_vs_M2
8,04E-17	-0,7895161	0,014	0,204	2,66E-12	ATP6V0D2	DWWIDA_macrophage_vs_M2
8,80E-17	-0,4469972	0	0,172	2,91E-12	SHE	DWWIDA_macrophage_vs_M2
1,07E-16	-0,9785585	0,342	0,573	3,55E-12	ADAP2	DWWIDA_macrophage_vs_M2
1,15E-16	-0,490104	0,019	0,218	3,80E-12	HRH1	DWWIDA_macrophage_vs_M2
1,83E-16	-1,0691905	0,378	0,602	6,05E-12	GM2A	DWWIDA_macrophage_vs_M2
2,02E-16	-0,5590211	0,024	0,22	6,69E-12	ADORA3	DWWIDA_macrophage_vs_M2
2,31E-16	-0,9879335	0,726	0,759	7,64E-12	GNAS	DWWIDA_macrophage_vs_M2
3,76E-16	-0,9278471	0,196	0,451	1,24E-11	NPL	DWWIDA_macrophage_vs_M2
9,81E-16	-0,8194956	0,742	0,79	3,25E-11	MARCKS	DWWIDA_macrophage_vs_M2
1,55E-15	-1,1367185	0,459	0,639	5,12E-11	PRNP	DWWIDA_macrophage_vs_M2
2,19E-15	-0,9240308	0,207	0,443	7,24E-11	IDH1	DWWIDA_macrophage_vs_M2
2,45E-15	-0,4613882	0,052	0,26	8,12E-11	ITGA9	DWWIDA_macrophage_vs_M2
2,94E-15	-1,0850537	0,543	0,737	9,74E-11	MRC1	DWWIDA_macrophage_vs_M2
4,34E-15	-0,6445289	0,035	0,228	1,44E-10	IGFBP4	DWWIDA_macrophage_vs_M2
4,52E-15	-0,4408049	0,024	0,21	1,50E-10	EMP2	DWWIDA_macrophage_vs_M2
9,38E-15	-0,8458187	0,62	0,806	3,11E-10	TMEM176B	DWWIDA_macrophage_vs_M2
1,36E-14	-0,6099577	0,938	0,942	4,52E-10	FGL2	DWWIDA_macrophage_vs_M2
2,69E-14	-0,7555275	0,204	0,438	8,92E-10	MYO5A	DWWIDA_macrophage_vs_M2
2,99E-14	-0,4026826	0,011	0,172	9,92E-10	CTSF	DWWIDA_macrophage_vs_M2
3,70E-14	-0,6977622	0,823	0,814	1,23E-09	MPEG1	DWWIDA_macrophage_vs_M2
4,99E-14	-0,396059	0,027	0,204	1,65E-09	GAL3ST4	DWWIDA_macrophage_vs_M2
7,13E-14	-0,7462677	0,098	0,308	2,36E-09	FAM213A	DWWIDA_macrophage_vs_M2
7,22E-14	-0,886093	0,549	0,674	2,39E-09	NCF4	DWWIDA_macrophage_vs_M2
9,52E-14	-0,4717319	0,082	0,294	3,15E-09	SLC9A9	DWWIDA_macrophage_vs_M2
1,06E-13	-0,8861917	0,212	0,451	3,51E-09	VSIG4	DWWIDA_macrophage_vs_M2
1,15E-13	-0,96553	0,103	0,313	3,81E-09	FILIP1L	DWWIDA_macrophage_vs_M2
1,48E-13	-0,7935963	0,291	0,512	4,90E-09	SMS	DWWIDA_macrophage_vs_M2
1,48E-13	-0,9694548	0,465	0,61	4,90E-09	PEBP1	DWWIDA_macrophage_vs_M2
1,52E-13	-0,5594025	0,133	0,363	5,02E-09	GPR155	DWWIDA_macrophage_vs_M2
1,54E-13	-0,4591302	0,014	0,17	5,10E-09	ADGRG6	DWWIDA_macrophage_vs_M2
1,79E-13	-0,834216	0,304	0,531	5,91E-09	FRMD4B	DWWIDA_macrophage_vs_M2
2,12E-13	-0,5856463	0,049	0,236	7,03E-09	NMRK1	DWWIDA_macrophage_vs_M2
2,18E-13	-0,5412425	0,997	0,984	7,23E-09	MT-ND3	DWWIDA_macrophage_vs_M2
2,23E-13	-0,6543977	0,033	0,207	7,40E-09	OTOA	DWWIDA_macrophage_vs_M2
2,30E-13	-0,8164135	0,283	0,501	7,62E-09	GNPDA1	DWWIDA_macrophage_vs_M2
5,22E-13	-0,7771085	0,239	0,462	1,73E-08	TSPAN4	DWWIDA_macrophage_vs_M2
5,57E-13	-0,4418542	0,073	0,271	1,84E-08	ABCC5	DWWIDA_macrophage_vs_M2
8,35E-13	-0,9510142	0,22	0,432	2,77E-08	LGALS3BP	DWWIDA_macrophage_vs_M2
9,78E-13	-0,6823752	0,09	0,289	3,24E-08	TNFRSF21	DWWIDA_macrophage_vs_M2
1,07E-12	-0,4103357	0,005	0,143	3,54E-08	PLXDC1	DWWIDA_macrophage_vs_M2
1,09E-12	-0,4209925	0,016	0,167	3,61E-08	BEX3	DWWIDA_macrophage_vs_M2
1,10E-12	-0,8402231	0,106	0,324	3,66E-08	MMP12	DWWIDA_macrophage_vs_M2
1,22E-12	-0,533675	0,057	0,241	4,06E-08	VAT1	DWWIDA_macrophage_vs_M2
1,50E-12	-0,4564584	0,046	0,223	4,98E-08	ARHGEF12	DWWIDA_macrophage_vs_M2

1,75E-12	-0,5216556	0,014	0,159	5,78E-08	LAG3	DWW IDA_macrophage_vs_M2
2,02E-12	-0,625434	0,008	0,146	6,68E-08	VCAM1	DWW IDA_macrophage_vs_M2
2,18E-12	-0,3444484	0,008	0,146	7,22E-08	CRHBP	DWW IDA_macrophage_vs_M2
3,06E-12	-0,8876539	0,321	0,536	1,01E-07	C1orf54	DWW IDA_macrophage_vs_M2
3,07E-12	-0,7448214	0,266	0,491	1,02E-07	PTMS	DWW IDA_macrophage_vs_M2
3,46E-12	-0,6897273	0,13	0,337	1,15E-07	GFRA2	DWW IDA_macrophage_vs_M2
3,62E-12	-0,7487883	0,264	0,48	1,20E-07	SGPL1	DWW IDA_macrophage_vs_M2
3,69E-12	-0,4295726	0,041	0,21	1,22E-07	MFHAS1	DWW IDA_macrophage_vs_M2
3,92E-12	-1,1337316	0,027	0,183	1,30E-07	CCL2	DWW IDA_macrophage_vs_M2
4,03E-12	-0,78678	0,16	0,377	1,34E-07	NRP2	DWW IDA_macrophage_vs_M2
6,01E-12	-0,3649729	0,014	0,154	1,99E-07	RAB3IL1	DWW IDA_macrophage_vs_M2
6,96E-12	-0,8667623	0,223	0,438	2,31E-07	SPRED1	DWW IDA_macrophage_vs_M2
7,39E-12	-0,3599373	0,027	0,18	2,45E-07	CTTNBP2	DWW IDA_macrophage_vs_M2
8,94E-12	-0,8904418	0,503	0,626	2,96E-07	SNX6	DWW IDA_macrophage_vs_M2
1,11E-11	-0,8647763	0,728	0,804	3,67E-07	CD14	DWW IDA_macrophage_vs_M2
1,42E-11	-0,6010071	0,171	0,379	4,71E-07	NISCH	DWW IDA_macrophage_vs_M2
1,49E-11	-0,7167864	0,636	0,737	4,94E-07	CD4	DWW IDA_macrophage_vs_M2
2,06E-11	-1,0049658	0,408	0,581	6,82E-07	HMOX1	DWW IDA_macrophage_vs_M2
2,32E-11	-0,9945558	0,111	0,3	7,69E-07	PDK4	DWW IDA_macrophage_vs_M2
3,08E-11	-0,5838954	0,122	0,316	1,02E-06	PRKACB	DWW IDA_macrophage_vs_M2
3,84E-11	-0,467463	0,076	0,255	1,27E-06	MMD	DWW IDA_macrophage_vs_M2
4,57E-11	-0,8165321	0,076	0,252	1,51E-06	IL2RA	DWW IDA_macrophage_vs_M2
4,89E-11	-0,473179	0,038	0,191	1,62E-06	EPHB2	DWW IDA_macrophage_vs_M2
5,56E-11	-0,3496423	0,03	0,178	1,84E-06	SLC18B1	DWW IDA_macrophage_vs_M2
6,75E-11	-0,5438618	0,951	0,91	2,24E-06	HLA-E	DWW IDA_macrophage_vs_M2
7,51E-11	-0,8057663	0,432	0,586	2,49E-06	ARHGAP18	DWW IDA_macrophage_vs_M2
1,03E-10	-0,4296081	0,016	0,146	3,42E-06	CCDC152	DWW IDA_macrophage_vs_M2
1,18E-10	-0,7298134	0,291	0,493	3,91E-06	RGL1	DWW IDA_macrophage_vs_M2
1,30E-10	-0,8092852	0,696	0,721	4,31E-06	CD81	DWW IDA_macrophage_vs_M2
1,42E-10	-0,5426445	0,103	0,286	4,71E-06	MGLL	DWW IDA_macrophage_vs_M2
1,48E-10	-0,6046827	0,147	0,334	4,89E-06	TGFBR1	DWW IDA_macrophage_vs_M2
1,87E-10	-0,2859704	0,005	0,119	6,19E-06	BCAM	DWW IDA_macrophage_vs_M2
2,19E-10	-0,6536355	0,883	0,87	7,25E-06	CALM2	DWW IDA_macrophage_vs_M2
2,97E-10	-0,5510467	0,133	0,321	9,83E-06	EPB41L2	DWW IDA_macrophage_vs_M2
3,44E-10	-0,397631	0,997	0,987	1,14E-05	MT-CO3	DWW IDA_macrophage_vs_M2
3,72E-10	-0,8171552	0,522	0,679	1,23E-05	CTSL	DWW IDA_macrophage_vs_M2
3,73E-10	-0,7590887	0,497	0,618	1,24E-05	CTSA	DWW IDA_macrophage_vs_M2
4,24E-10	-0,4070937	0,062	0,225	1,40E-05	SCARB1	DWW IDA_macrophage_vs_M2
4,30E-10	-0,7163182	0,296	0,485	1,43E-05	LPAR6	DWW IDA_macrophage_vs_M2
4,52E-10	-0,3186705	0,011	0,127	1,50E-05	SCN1B	DWW IDA_macrophage_vs_M2
5,27E-10	-0,4958591	0,894	0,83	1,75E-05	TMBIM6	DWW IDA_macrophage_vs_M2
6,73E-10	-0,751696	0,364	0,528	2,23E-05	HEXA	DWW IDA_macrophage_vs_M2
6,80E-10	-0,4536213	0,062	0,218	2,25E-05	PDGFC	DWW IDA_macrophage_vs_M2
7,96E-10	-0,6444256	0,033	0,164	2,64E-05	TMIGD3	DWW IDA_macrophage_vs_M2
8,16E-10	-0,4648791	0,019	0,141	2,70E-05	RBP1	DWW IDA_macrophage_vs_M2
8,55E-10	-0,3136127	0,005	0,111	2,83E-05	SLC12A5	DWW IDA_macrophage_vs_M2

8,94E-10	-0,5444521	0,198	0,395	2,96E-05	ZFHX3	DWW	IDA_macrophage_vs_M2
1,11E-09	-0,579107	0,133	0,313	3,69E-05	GLMP	DWW	IDA_macrophage_vs_M2
1,15E-09	-0,5181838	0,103	0,276	3,81E-05	ETV5	DWW	IDA_macrophage_vs_M2
1,72E-09	-0,5640291	0,935	0,92	5,70E-05	CD63	DWW	IDA_macrophage_vs_M2
1,89E-09	-0,4766675	0,179	0,374	6,26E-05	ADAM28	DWW	IDA_macrophage_vs_M2
2,04E-09	-0,3204586	0,033	0,164	6,75E-05	FOXRED2	DWW	IDA_macrophage_vs_M2
2,20E-09	-0,4527729	0,13	0,305	7,29E-05	DST	DWW	IDA_macrophage_vs_M2
2,67E-09	-0,4022509	0,057	0,204	8,85E-05	APPL2	DWW	IDA_macrophage_vs_M2
2,99E-09	-0,4404677	0,114	0,289	9,89E-05	DHRS3	DWW	IDA_macrophage_vs_M2
3,05E-09	-0,9218523	0,302	0,469	0,0001	SERPING1	DWW	IDA_macrophage_vs_M2
3,08E-09	-0,4303443	0,041	0,175	0,0001	STON2	DWW	IDA_macrophage_vs_M2
3,93E-09	-0,6455488	0,351	0,52	0,00013	CYFIP1	DWW	IDA_macrophage_vs_M2
4,49E-09	-0,5580125	0,185	0,371	0,00015	VEGFB	DWW	IDA_macrophage_vs_M2
4,51E-09	-0,5030357	0,938	0,947	0,00015	HLA-DQA1	DWW	IDA_macrophage_vs_M2
4,58E-09	-0,2886106	0,019	0,133	0,00015	CNRIP1	DWW	IDA_macrophage_vs_M2
4,68E-09	-0,4885048	0,967	0,971	0,00016	CST3	DWW	IDA_macrophage_vs_M2
5,22E-09	-0,6755336	0,217	0,393	0,00017	ABHD12	DWW	IDA_macrophage_vs_M2
5,40E-09	-0,7411799	0,152	0,326	0,00018	TCN2	DWW	IDA_macrophage_vs_M2
5,68E-09	-0,5071484	0,179	0,355	0,00019	OSBPL1A	DWW	IDA_macrophage_vs_M2
6,00E-09	-0,7096447	0,484	0,61	0,0002	RB1	DWW	IDA_macrophage_vs_M2
6,28E-09	-0,484596	0,079	0,228	0,00021	SLC38A6	DWW	IDA_macrophage_vs_M2
7,09E-09	-0,3587594	0,033	0,156	0,00023	PER3	DWW	IDA_macrophage_vs_M2
7,75E-09	-0,3173456	0,046	0,178	0,00026	SH3PXD2A	DWW	IDA_macrophage_vs_M2
8,73E-09	-0,425219	0,068	0,212	0,00029	S100A13	DWW	IDA_macrophage_vs_M2
9,43E-09	-0,9070634	0,168	0,334	0,00031	MAN1A1	DWW	IDA_macrophage_vs_M2
9,51E-09	-0,5161515	0,902	0,875	0,00031	HLA-DMA	DWW	IDA_macrophage_vs_M2
9,67E-09	-0,4005475	0,128	0,3	0,00032	CLIC2	DWW	IDA_macrophage_vs_M2
1,05E-08	-0,6016817	0,981	0,987	0,00035	HLA-A	DWW	IDA_macrophage_vs_M2
1,11E-08	-1,174247	0,527	0,623	0,00037	LMNA	DWW	IDA_macrophage_vs_M2
1,11E-08	-0,6186191	0,264	0,446	0,00037	MGAT4A	DWW	IDA_macrophage_vs_M2
1,30E-08	-0,5948096	0,272	0,446	0,00043	CD84	DWW	IDA_macrophage_vs_M2
1,42E-08	-0,3974376	0,136	0,305	0,00047	MERTK	DWW	IDA_macrophage_vs_M2
2,65E-08	-0,323839	0,06	0,199	0,00088	SRGAP3	DWW	IDA_macrophage_vs_M2
3,11E-08	-0,6020942	0,378	0,549	0,00103	HLA-DOA	DWW	IDA_macrophage_vs_M2
3,16E-08	-0,513997	0,139	0,302	0,00105	NTAN1	DWW	IDA_macrophage_vs_M2
3,40E-08	-0,4932439	0,188	0,361	0,00113	FCHO2	DWW	IDA_macrophage_vs_M2
3,48E-08	-0,626664	0,234	0,398	0,00115	ITGAV	DWW	IDA_macrophage_vs_M2
3,77E-08	-0,6827544	0,258	0,435	0,00125	MMP9	DWW	IDA_macrophage_vs_M2
3,83E-08	-0,5207711	0,114	0,273	0,00127	GCLC	DWW	IDA_macrophage_vs_M2
4,60E-08	-0,7709114	0,595	0,663	0,00152	BLVRB	DWW	IDA_macrophage_vs_M2
4,74E-08	-0,2976995	0,022	0,127	0,00157	MYO7A	DWW	IDA_macrophage_vs_M2
5,04E-08	-0,7737348	0,565	0,634	0,00167	CLTC	DWW	IDA_macrophage_vs_M2
5,25E-08	-0,3827607	0,054	0,186	0,00174	PCYOX1	DWW	IDA_macrophage_vs_M2
5,38E-08	-0,4606006	0,071	0,204	0,00178	EPAS1	DWW	IDA_macrophage_vs_M2
6,80E-08	-0,2715812	0,019	0,119	0,00225	RAB42	DWW	IDA_macrophage_vs_M2
6,88E-08	-0,4828698	0,149	0,308	0,00228	LINC00996	DWW	IDA_macrophage_vs_M2

6,99E-08	-0,3099037	0,052	0,178	0,00231	RHOBTB3	DWW IDA_macrophage_vs_M2
7,62E-08	-0,6086112	0,601	0,7	0,00253	FPR3	DWW IDA_macrophage_vs_M2
8,28E-08	-0,5424746	0,234	0,401	0,00274	TRIM14	DWW IDA_macrophage_vs_M2
9,45E-08	-0,6512772	0,81	0,793	0,00313	MTRNR2L8	DWW IDA_macrophage_vs_M2
1,14E-07	-0,6636866	0,163	0,326	0,00377	PLA2G16	DWW IDA_macrophage_vs_M2
1,23E-07	-0,4190984	0,16	0,321	0,00408	DPP9	DWW IDA_macrophage_vs_M2
1,29E-07	-0,4059945	0,995	0,984	0,00427	MT-CYB	DWW IDA_macrophage_vs_M2
1,71E-07	-0,3196186	0,03	0,135	0,00566	FGFR1	DWW IDA_macrophage_vs_M2
1,77E-07	-0,3499173	0,997	0,989	0,00586	MT-ATP6	DWW IDA_macrophage_vs_M2
2,15E-07	-0,534923	0,163	0,313	0,00712	GYPC	DWW IDA_macrophage_vs_M2
2,16E-07	-0,3255894	0,079	0,212	0,00716	FMNL2	DWW IDA_macrophage_vs_M2
2,18E-07	-0,544579	0,182	0,342	0,00722	EMB	DWW IDA_macrophage_vs_M2
2,49E-07	-0,4158214	0,046	0,162	0,00823	KLHDC8B	DWW IDA_macrophage_vs_M2
2,53E-07	-0,41199	0,082	0,215	0,00837	NFATC2	DWW IDA_macrophage_vs_M2
2,98E-07	-0,6950708	0,53	0,629	0,00986	LAMP2	DWW IDA_macrophage_vs_M2
3,06E-07	-0,6430535	0,679	0,7	0,01012	TPP1	DWW IDA_macrophage_vs_M2
3,11E-07	-0,3753701	0,12	0,268	0,01031	sep-11	DWW IDA_macrophage_vs_M2
3,48E-07	-0,4996164	0,87	0,836	0,01153	HLA-DMB	DWW IDA_macrophage_vs_M2
3,74E-07	-0,4886297	0,076	0,204	0,01237	TCEAL3	DWW IDA_macrophage_vs_M2
3,79E-07	-0,315985	0,092	0,228	0,01255	SPIN1	DWW IDA_macrophage_vs_M2
4,17E-07	-0,4964616	0,867	0,836	0,01383	ASAH1	DWW IDA_macrophage_vs_M2
4,18E-07	-0,657951	0,785	0,764	0,01386	RAC1	DWW IDA_macrophage_vs_M2
4,74E-07	-0,7367222	0,519	0,605	0,0157	LAMP1	DWW IDA_macrophage_vs_M2
5,56E-07	-0,5466568	0,212	0,369	0,01841	PARP1	DWW IDA_macrophage_vs_M2
6,38E-07	-0,8135757	0,796	0,732	0,02112	ARL4C	DWW IDA_macrophage_vs_M2
6,50E-07	-0,3675547	0,073	0,196	0,02154	ABCD4	DWW IDA_macrophage_vs_M2
6,91E-07	-0,5578734	0,408	0,525	0,0229	IQGAP2	DWW IDA_macrophage_vs_M2
7,08E-07	-0,3811556	0,095	0,225	0,02346	CYBRD1	DWW IDA_macrophage_vs_M2
7,25E-07	-0,2709529	0,057	0,172	0,02402	SNX24	DWW IDA_macrophage_vs_M2
7,54E-07	-0,5093737	0,057	0,175	0,02499	CXCL1	DWW IDA_macrophage_vs_M2
7,56E-07	-0,4443196	0,198	0,353	0,02505	ACP2	DWW IDA_macrophage_vs_M2
7,67E-07	-0,4397194	0,149	0,3	0,0254	GDE1	DWW IDA_macrophage_vs_M2
8,08E-07	-0,2699766	0,046	0,154	0,02677	PLEKHA1	DWW IDA_macrophage_vs_M2
8,19E-07	-0,3296039	0,087	0,215	0,02713	FAM13A	DWW IDA_macrophage_vs_M2
9,50E-07	-0,4419497	0,149	0,289	0,03146	SGMS1	DWW IDA_macrophage_vs_M2
1,04E-06	-0,603619	0,253	0,398	0,03454	CMKLR1	DWW IDA_macrophage_vs_M2
1,06E-06	-0,4367308	0,071	0,191	0,03518	TCEAL9	DWW IDA_macrophage_vs_M2
1,10E-06	-0,594577	0,473	0,576	0,03635	TIMP2	DWW IDA_macrophage_vs_M2
1,16E-06	-0,5572113	0,236	0,382	0,03833	ADAM9	DWW IDA_macrophage_vs_M2
1,19E-06	-0,3036507	0,054	0,164	0,03927	ZDHHC14	DWW IDA_macrophage_vs_M2
1,19E-06	-0,3723962	0,092	0,223	0,03932	EBI3	DWW IDA_macrophage_vs_M2
1,34E-06	-0,6623091	0,397	0,538	0,04434	TTYH3	DWW IDA_macrophage_vs_M2

p_val	avg_log2FC	pct.1	pct.2	p_val_adj	gene	sign	comp
4,12E-66	1,73522695	0,804	0,213	1,36E-61	JAML	UPP	IDA_macrophage_vs_M1
5,29E-65	1,65066761	0,938	0,514	1,75E-60	FGL2	UPP	IDA_macrophage_vs_M1
1,16E-57	1,40664352	0,992	0,866	3,83E-53	HLA-DPB1	UPP	IDA_macrophage_vs_M1
5,03E-56	1,12692359	1	0,992	1,67E-51	TMSB4X	UPP	IDA_macrophage_vs_M1
1,36E-55	1,40758896	0,87	0,471	4,52E-51	HLA-DMB	UPP	IDA_macrophage_vs_M1
1,07E-54	1,27686126	0,905	0,516	3,55E-50	VAMP8	UPP	IDA_macrophage_vs_M1
5,84E-54	1,17124291	0,976	0,706	1,94E-49	LST1	UPP	IDA_macrophage_vs_M1
2,60E-53	1,21002393	0,965	0,861	8,60E-49	RPL27A	UPP	IDA_macrophage_vs_M1
3,31E-53	1,75722185	0,875	0,397	1,10E-48	RGS2	UPP	IDA_macrophage_vs_M1
9,19E-53	0,989276	0,995	0,932	3,04E-48	RPS14	UPP	IDA_macrophage_vs_M1
1,26E-52	1,62350333	0,796	0,281	4,17E-48	ARL4C	UPP	IDA_macrophage_vs_M1
2,72E-52	1,16361262	0,962	0,856	9,01E-48	RPL13A	UPP	IDA_macrophage_vs_M1
1,29E-50	1,39151737	0,992	0,927	4,26E-46	HLA-DRB1	UPP	IDA_macrophage_vs_M1
1,72E-50	1,44166512	0,845	0,359	5,70E-46	MS4A6A	UPP	IDA_macrophage_vs_M1
2,96E-50	0,99244638	0,989	0,924	9,81E-46	AIF1	UPP	IDA_macrophage_vs_M1
6,97E-50	1,2799003	0,995	0,889	2,31E-45	HLA-DPA1	UPP	IDA_macrophage_vs_M1
1,43E-49	1,86971599	0,967	0,808	4,75E-45	CST3	UPP	IDA_macrophage_vs_M1
2,55E-49	1,31181519	0,976	0,861	8,44E-45	RPS2	UPP	IDA_macrophage_vs_M1
1,08E-48	1,67966073	0,592	0,111	3,56E-44	CLEC10A	UPP	IDA_macrophage_vs_M1
2,39E-48	1,07709513	1	0,949	7,91E-44	CD74	UPP	IDA_macrophage_vs_M1
3,67E-47	0,9630273	0,984	0,896	1,21E-42	RPS6	UPP	IDA_macrophage_vs_M1
1,04E-46	1,74118969	0,728	0,253	3,43E-42	ZNF331	UPP	IDA_macrophage_vs_M1
1,84E-46	0,84970643	0,995	0,967	6,08E-42	RPS19	UPP	IDA_macrophage_vs_M1
2,53E-45	0,91332933	0,981	0,906	8,37E-41	RPS11	UPP	IDA_macrophage_vs_M1
2,95E-45	1,29307229	0,823	0,377	9,78E-41	MPEG1	UPP	IDA_macrophage_vs_M1
9,21E-45	1,15229499	0,929	0,691	3,05E-40	RPSA	UPP	IDA_macrophage_vs_M1
1,08E-44	0,74008101	0,997	0,952	3,57E-40	PTMA	UPP	IDA_macrophage_vs_M1
1,70E-44	1,49419974	0,668	0,19	5,63E-40	CEBPD	UPP	IDA_macrophage_vs_M1
6,97E-44	0,8312679	0,986	0,873	2,31E-39	RPL35	UPP	IDA_macrophage_vs_M1
3,45E-42	0,97025885	0,965	0,81	1,14E-37	RPL10A	UPP	IDA_macrophage_vs_M1
1,08E-41	2,74387791	0,595	0,159	3,56E-37	C1QA	UPP	IDA_macrophage_vs_M1
2,34E-41	1,58986381	0,802	0,365	7,76E-37	CXCR4	UPP	IDA_macrophage_vs_M1
3,10E-41	1,47987877	0,87	0,496	1,03E-36	NR4A2	UPP	IDA_macrophage_vs_M1
3,46E-41	1,28117939	0,883	0,557	1,15E-36	TUBA1B	UPP	IDA_macrophage_vs_M1
1,69E-40	1,19974616	0,97	0,858	5,60E-36	RPL13	UPP	IDA_macrophage_vs_M1
2,84E-40	1,57502865	0,938	0,699	9,40E-36	HLA-DQA1	UPP	IDA_macrophage_vs_M1
1,13E-39	1,34115487	0,948	0,795	3,73E-35	HLA-DQB1	UPP	IDA_macrophage_vs_M1
1,84E-39	1,46572777	0,823	0,489	6,10E-35	HERPUD1	UPP	IDA_macrophage_vs_M1
3,95E-39	0,81175787	0,981	0,906	1,31E-34	RPL18A	UPP	IDA_macrophage_vs_M1
1,22E-38	0,83043623	0,976	0,884	4,05E-34	RPL23	UPP	IDA_macrophage_vs_M1
5,26E-38	0,88530747	0,973	0,899	1,74E-33	RPL3	UPP	IDA_macrophage_vs_M1
1,18E-37	0,96160795	0,899	0,686	3,90E-33	RPL4	UPP	IDA_macrophage_vs_M1
1,22E-37	0,85535508	0,981	0,866	4,03E-33	RPL23A	UPP	IDA_macrophage_vs_M1
2,57E-37	1,35759192	0,943	0,671	8,50E-33	FOSB	UPP	IDA_macrophage_vs_M1
8,60E-37	1,06779187	0,929	0,767	2,85E-32	ITM2B	UPP	IDA_macrophage_vs_M1
1,53E-36	1,44628289	0,821	0,491	5,07E-32	CHMP1B	UPP	IDA_macrophage_vs_M1
1,55E-36	2,40634293	0,557	0,152	5,13E-32	C1QB	UPP	IDA_macrophage_vs_M1
3,51E-35	0,91268816	0,679	0,251	1,16E-30	AOAH	UPP	IDA_macrophage_vs_M1
5,01E-35	1,45923603	0,658	0,241	1,66E-30	ZFP36L2	UPP	IDA_macrophage_vs_M1

5,38E-35	0,9923123	0,793	0,332	1,78E-30	MNDA	UPP	IDA_macrophage_vs_M1
8,81E-35	0,80849668	0,992	0,944	2,92E-30	RPS8	UPP	IDA_macrophage_vs_M1
1,25E-34	1,42136053	0,742	0,337	4,13E-30	KLF4	UPP	IDA_macrophage_vs_M1
1,66E-34	0,95820534	0,902	0,666	5,49E-30	HLA-DMA	UPP	IDA_macrophage_vs_M1
1,70E-34	0,82010861	0,973	0,838	5,63E-30	RPL7	UPP	IDA_macrophage_vs_M1
2,10E-34	1,94665092	0,535	0,132	6,95E-30	C1QC	UPP	IDA_macrophage_vs_M1
2,39E-34	0,67887794	0,989	0,954	7,91E-30	RPS9	UPP	IDA_macrophage_vs_M1
1,61E-33	0,84417259	0,943	0,759	5,32E-29	EEF1B2	UPP	IDA_macrophage_vs_M1
1,95E-33	0,65246034	0,992	0,952	6,45E-29	RPL9	UPP	IDA_macrophage_vs_M1
2,06E-33	1,35699353	0,375	0,028	6,82E-29	SLC40A1	UPP	IDA_macrophage_vs_M1
2,48E-33	0,85843932	0,723	0,319	8,21E-29	ERP29	UPP	IDA_macrophage_vs_M1
4,78E-33	0,97575894	0,731	0,316	1,58E-28	C1orf162	UPP	IDA_macrophage_vs_M1
6,07E-33	0,78006742	0,954	0,815	2,01E-28	NPC2	UPP	IDA_macrophage_vs_M1
6,49E-33	1,30423736	0,938	0,762	2,15E-28	JUNB	UPP	IDA_macrophage_vs_M1
7,91E-33	0,81109094	0,935	0,658	2,62E-28	ATP5MC2	UPP	IDA_macrophage_vs_M1
8,79E-33	0,8858496	0,875	0,575	2,91E-28	SLC25A5	UPP	IDA_macrophage_vs_M1
9,74E-33	0,85491498	0,443	0,068	3,23E-28	P2RY13	UPP	IDA_macrophage_vs_M1
1,86E-32	0,72384812	0,978	0,916	6,16E-28	RPL37A	UPP	IDA_macrophage_vs_M1
2,85E-32	1,85445008	0,804	0,443	9,45E-28	FOS	UPP	IDA_macrophage_vs_M1
1,80E-31	0,66766579	0,984	0,947	5,95E-27	RPL18	UPP	IDA_macrophage_vs_M1
2,34E-31	1,0081471	0,954	0,744	7,76E-27	COTL1	UPP	IDA_macrophage_vs_M1
4,97E-31	0,70718884	0,978	0,942	1,64E-26	RPL15	UPP	IDA_macrophage_vs_M1
2,40E-30	0,57281261	0,992	0,957	7,94E-26	UBA52	UPP	IDA_macrophage_vs_M1
2,41E-30	0,76017846	0,965	0,924	7,98E-26	PSAP	UPP	IDA_macrophage_vs_M1
2,62E-30	1,06427947	0,864	0,559	8,67E-26	CTSC	UPP	IDA_macrophage_vs_M1
4,59E-30	1,7318332	0,538	0,165	1,52E-25	RGS1	UPP	IDA_macrophage_vs_M1
5,63E-30	1,04906508	0,935	0,661	1,86E-25	GPR183	UPP	IDA_macrophage_vs_M1
7,34E-30	1,1357578	0,758	0,408	2,43E-25	LGALS2	UPP	IDA_macrophage_vs_M1
1,35E-29	0,82976859	0,875	0,608	4,47E-25	ARHGDIB	UPP	IDA_macrophage_vs_M1
1,93E-29	0,66877888	0,989	0,929	6,38E-25	RPS3A	UPP	IDA_macrophage_vs_M1
4,10E-29	0,57736935	0,997	0,982	1,36E-24	EEF1A1	UPP	IDA_macrophage_vs_M1
4,37E-29	1,36233963	0,745	0,392	1,45E-24	TXNIP	UPP	IDA_macrophage_vs_M1
5,58E-29	0,51541485	0,995	0,977	1,85E-24	RPL28	UPP	IDA_macrophage_vs_M1
6,01E-29	0,72210334	0,978	0,922	1,99E-24	RPL7A	UPP	IDA_macrophage_vs_M1
7,16E-29	0,78911131	0,938	0,734	2,37E-24	RPLP0	UPP	IDA_macrophage_vs_M1
7,39E-29	0,67125326	0,976	0,866	2,45E-24	RPL27	UPP	IDA_macrophage_vs_M1
3,64E-28	0,53399175	0,997	0,977	1,20E-23	RPLP1	UPP	IDA_macrophage_vs_M1
4,41E-28	0,63143797	0,995	0,97	1,46E-23	RPL11	UPP	IDA_macrophage_vs_M1
1,43E-27	0,60183652	0,997	0,972	4,73E-23	RPS12	UPP	IDA_macrophage_vs_M1
1,46E-27	0,62986684	0,984	0,949	4,84E-23	RPS23	UPP	IDA_macrophage_vs_M1
2,37E-27	0,5615566	0,989	0,954	7,84E-23	RPL19	UPP	IDA_macrophage_vs_M1
3,12E-27	0,59297709	0,986	0,929	1,03E-22	RPL21	UPP	IDA_macrophage_vs_M1
3,38E-27	0,67778746	0,978	0,927	1,12E-22	RPS3	UPP	IDA_macrophage_vs_M1
4,98E-27	1,34669429	0,416	0,084	1,65E-22	SDS	UPP	IDA_macrophage_vs_M1
9,78E-27	0,77806889	0,755	0,405	3,24E-22	VSIR	UPP	IDA_macrophage_vs_M1
1,35E-26	0,82808331	0,951	0,772	4,46E-22	ZFP36	UPP	IDA_macrophage_vs_M1
1,80E-26	0,63679974	0,459	0,111	5,95E-22	SCIMP	UPP	IDA_macrophage_vs_M1
3,02E-26	0,63959256	0,348	0,048	9,99E-22	CD1D	UPP	IDA_macrophage_vs_M1
3,02E-26	0,72489741	0,938	0,803	1,00E-21	RPL5	UPP	IDA_macrophage_vs_M1
5,35E-26	1,35664011	0,658	0,327	1,77E-21	TUBA1A	UPP	IDA_macrophage_vs_M1

7,12E-26	0,61080498	0,959	0,947	2,36E-21	RPS15	UPP	IDA_macrophage_vs_M1
1,88E-25	0,89121536	0,495	0,154	6,24E-21	STAB1	UPP	IDA_macrophage_vs_M1
2,25E-25	0,65136273	0,932	0,795	7,45E-21	PPIA	UPP	IDA_macrophage_vs_M1
2,84E-25	0,76158808	0,984	0,914	9,41E-21	RPS18	UPP	IDA_macrophage_vs_M1
4,90E-25	0,58419026	0,989	0,962	1,62E-20	RPL32	UPP	IDA_macrophage_vs_M1
5,04E-25	0,87880831	0,84	0,554	1,67E-20	SAMHD1	UPP	IDA_macrophage_vs_M1
6,72E-25	0,83678829	0,552	0,21	2,23E-20	PER1	UPP	IDA_macrophage_vs_M1
8,64E-25	0,72459828	0,576	0,22	2,86E-20	PAK1	UPP	IDA_macrophage_vs_M1
8,73E-25	0,67925472	0,549	0,19	2,89E-20	NCF4	UPP	IDA_macrophage_vs_M1
9,56E-25	0,52381324	0,995	0,985	3,17E-20	RPL10	UPP	IDA_macrophage_vs_M1
1,21E-24	0,77109626	0,633	0,286	4,00E-20	SH2B3	UPP	IDA_macrophage_vs_M1
1,25E-24	0,57997234	0,981	0,924	4,13E-20	RPS25	UPP	IDA_macrophage_vs_M1
1,57E-24	0,78997096	0,837	0,623	5,19E-20	SLC25A6	UPP	IDA_macrophage_vs_M1
2,00E-24	0,61288386	0,386	0,073	6,62E-20	CAMK2D	UPP	IDA_macrophage_vs_M1
3,19E-24	0,66568747	0,489	0,159	1,06E-19	APEX1	UPP	IDA_macrophage_vs_M1
3,27E-24	0,62318936	0,962	0,894	1,08E-19	RPL14	UPP	IDA_macrophage_vs_M1
3,79E-24	0,89163617	0,709	0,413	1,26E-19	PYCARD	UPP	IDA_macrophage_vs_M1
4,61E-24	0,74454197	0,908	0,815	1,53E-19	RPS5	UPP	IDA_macrophage_vs_M1
4,92E-24	0,78169877	0,84	0,532	1,63E-19	FYB1	UPP	IDA_macrophage_vs_M1
7,15E-24	0,62610453	0,951	0,866	2,37E-19	RPL38	UPP	IDA_macrophage_vs_M1
1,06E-23	0,73062861	0,777	0,471	3,52E-19	HMGN2	UPP	IDA_macrophage_vs_M1
1,42E-23	0,87148708	0,807	0,476	4,71E-19	PPA1	UPP	IDA_macrophage_vs_M1
2,09E-23	0,80393643	0,378	0,089	6,93E-19	HLA-DOA	UPP	IDA_macrophage_vs_M1
2,49E-23	0,6437318	0,299	0,035	8,26E-19	HIC1	UPP	IDA_macrophage_vs_M1
3,00E-23	0,81581852	0,788	0,494	9,93E-19	RNASET2	UPP	IDA_macrophage_vs_M1
3,64E-23	0,62776009	0,454	0,124	1,21E-18	SIGLEC10	UPP	IDA_macrophage_vs_M1
4,99E-23	0,60554667	0,997	0,985	1,65E-18	HLA-DRA	UPP	IDA_macrophage_vs_M1
5,69E-23	1,0359832	0,682	0,352	1,89E-18	TSC22D3	UPP	IDA_macrophage_vs_M1
6,30E-23	0,70960863	0,435	0,124	2,09E-18	ABI3	UPP	IDA_macrophage_vs_M1
7,45E-23	0,68389822	0,883	0,608	2,47E-18	CALM2	UPP	IDA_macrophage_vs_M1
8,02E-23	0,57564895	0,978	0,916	2,66E-18	RPL6	UPP	IDA_macrophage_vs_M1
9,31E-23	0,88414823	0,764	0,43	3,08E-18	NR4A1	UPP	IDA_macrophage_vs_M1
1,59E-22	0,72704969	0,476	0,154	5,28E-18	GIMAP4	UPP	IDA_macrophage_vs_M1
2,42E-22	0,70816014	0,72	0,395	8,01E-18	WIPF1	UPP	IDA_macrophage_vs_M1
2,61E-22	0,55379309	0,981	0,876	8,63E-18	RPL22	UPP	IDA_macrophage_vs_M1
2,63E-22	0,68268179	0,671	0,314	8,73E-18	UTRN	UPP	IDA_macrophage_vs_M1
4,05E-22	0,64492386	0,954	0,835	1,34E-17	RPS20	UPP	IDA_macrophage_vs_M1
5,22E-22	0,53885385	1	0,954	1,73E-17	RPL12	UPP	IDA_macrophage_vs_M1
6,01E-22	0,82036284	0,481	0,162	1,99E-17	RHOB	UPP	IDA_macrophage_vs_M1
6,11E-22	0,8798231	0,707	0,413	2,02E-17	YPEL5	UPP	IDA_macrophage_vs_M1
8,57E-22	0,57415536	0,978	0,889	2,84E-17	PABPC1	UPP	IDA_macrophage_vs_M1
9,11E-22	0,62497265	0,457	0,147	3,02E-17	PRCP	UPP	IDA_macrophage_vs_M1
1,97E-21	0,68064016	0,927	0,795	6,53E-17	YBX1	UPP	IDA_macrophage_vs_M1
2,68E-21	0,70075215	0,679	0,329	8,88E-17	IRF2BP2	UPP	IDA_macrophage_vs_M1
3,77E-21	0,72135543	0,663	0,349	1,25E-16	AKR1A1	UPP	IDA_macrophage_vs_M1
7,61E-21	0,5636611	0,973	0,878	2,52E-16	RACK1	UPP	IDA_macrophage_vs_M1
1,81E-20	0,60563849	0,261	0,028	6,01E-16	GIMAP7	UPP	IDA_macrophage_vs_M1
4,73E-20	0,50662929	0,992	0,959	1,57E-15	RPL37	UPP	IDA_macrophage_vs_M1
4,94E-20	0,48417045	0,995	0,957	1,64E-15	RPLP2	UPP	IDA_macrophage_vs_M1
4,94E-20	0,64613435	0,916	0,709	1,64E-15	GSTP1	UPP	IDA_macrophage_vs_M1

5,43E-20	0,64254427	0,427	0,134	1,80E-15	TENT4B	UPP	IDA_macrophage_vs_M1
6,15E-20	0,58699645	0,351	0,081	2,04E-15	DAPK1	UPP	IDA_macrophage_vs_M1
6,32E-20	0,58696609	0,429	0,139	2,09E-15	GPRIN3	UPP	IDA_macrophage_vs_M1
6,56E-20	1,29637617	0,764	0,468	2,17E-15	ATF3	UPP	IDA_macrophage_vs_M1
7,03E-20	0,60607662	0,476	0,167	2,33E-15	ITGA4	UPP	IDA_macrophage_vs_M1
7,33E-20	0,58601127	0,378	0,104	2,43E-15	OTULINL	UPP	IDA_macrophage_vs_M1
7,64E-20	0,61045616	0,731	0,43	2,53E-15	CSF1R	UPP	IDA_macrophage_vs_M1
8,23E-20	0,5839424	0,465	0,172	2,73E-15	PEBP1	UPP	IDA_macrophage_vs_M1
1,04E-19	0,54659691	0,981	0,919	3,44E-15	RPL29	UPP	IDA_macrophage_vs_M1
1,15E-19	0,6226052	0,845	0,63	3,80E-15	LAMTOR4	UPP	IDA_macrophage_vs_M1
1,23E-19	0,57977933	0,97	0,919	4,06E-15	RPS4X	UPP	IDA_macrophage_vs_M1
1,81E-19	0,54463419	0,353	0,084	6,00E-15	AKNA	UPP	IDA_macrophage_vs_M1
1,84E-19	0,66126518	0,443	0,147	6,11E-15	USP36	UPP	IDA_macrophage_vs_M1
1,93E-19	0,79416094	0,753	0,471	6,38E-15	SRSF7	UPP	IDA_macrophage_vs_M1
2,41E-19	0,90076741	0,698	0,385	7,99E-15	HBEGF	UPP	IDA_macrophage_vs_M1
2,52E-19	0,49468366	0,986	0,954	8,36E-15	RPS16	UPP	IDA_macrophage_vs_M1
3,78E-19	0,57146976	0,592	0,281	1,25E-14	CNPY3	UPP	IDA_macrophage_vs_M1
4,13E-19	0,50731115	0,326	0,076	1,37E-14	MIS18BP1	UPP	IDA_macrophage_vs_M1
4,37E-19	0,47192366	0,288	0,048	1,45E-14	GZF1	UPP	IDA_macrophage_vs_M1
4,42E-19	0,69698135	0,772	0,514	1,46E-14	HIGD2A	UPP	IDA_macrophage_vs_M1
5,02E-19	0,55383037	0,916	0,749	1,66E-14	HMGB1	UPP	IDA_macrophage_vs_M1
5,55E-19	0,63571339	0,84	0,6	1,84E-14	HNRNPA1	UPP	IDA_macrophage_vs_M1
8,98E-19	0,58902483	0,663	0,365	2,98E-14	NAGK	UPP	IDA_macrophage_vs_M1
1,36E-18	0,62968263	0,405	0,129	4,50E-14	CDKN1B	UPP	IDA_macrophage_vs_M1
1,40E-18	0,62947527	0,595	0,291	4,64E-14	GSTK1	UPP	IDA_macrophage_vs_M1
1,79E-18	0,72393158	0,663	0,413	5,92E-14	ADA2	UPP	IDA_macrophage_vs_M1
1,81E-18	0,69390881	0,639	0,385	6,01E-14	EIF3F	UPP	IDA_macrophage_vs_M1
1,88E-18	0,52850145	0,986	0,949	6,21E-14	RPS7	UPP	IDA_macrophage_vs_M1
2,05E-18	0,9707098	0,704	0,478	6,79E-14	EZR	UPP	IDA_macrophage_vs_M1
4,16E-18	0,42039668	0,989	0,954	1,38E-13	RPS27A	UPP	IDA_macrophage_vs_M1
5,81E-18	0,7360538	0,78	0,562	1,92E-13	PTP4A2	UPP	IDA_macrophage_vs_M1
5,86E-18	0,65056234	0,747	0,476	1,94E-13	JPT1	UPP	IDA_macrophage_vs_M1
6,74E-18	0,51973303	0,53	0,23	2,23E-13	HMGN3	UPP	IDA_macrophage_vs_M1
9,12E-18	0,50235978	0,234	0,028	3,02E-13	AXL	UPP	IDA_macrophage_vs_M1
9,52E-18	0,49365461	0,943	0,841	3,15E-13	COX4I1	UPP	IDA_macrophage_vs_M1
9,92E-18	0,51624378	0,291	0,061	3,29E-13	RGS18	UPP	IDA_macrophage_vs_M1
1,07E-17	0,54028843	0,378	0,122	3,55E-13	PTPN18	UPP	IDA_macrophage_vs_M1
1,23E-17	0,58029386	0,476	0,19	4,07E-13	NAAA	UPP	IDA_macrophage_vs_M1
1,82E-17	0,63692887	0,582	0,291	6,03E-13	COX14	UPP	IDA_macrophage_vs_M1
2,03E-17	0,57257099	0,435	0,159	6,74E-13	KIAA0930	UPP	IDA_macrophage_vs_M1
2,45E-17	0,66312347	0,524	0,235	8,13E-13	SPINT2	UPP	IDA_macrophage_vs_M1
2,55E-17	0,74758818	0,693	0,425	8,46E-13	RAB20	UPP	IDA_macrophage_vs_M1
2,56E-17	0,54742221	0,541	0,228	8,48E-13	CD300A	UPP	IDA_macrophage_vs_M1
3,27E-17	0,52658361	0,293	0,063	1,08E-12	CEACAM4	UPP	IDA_macrophage_vs_M1
3,31E-17	0,43648664	0,992	0,975	1,10E-12	RPS24	UPP	IDA_macrophage_vs_M1
5,31E-17	0,59239706	0,514	0,238	1,76E-12	NDUFS8	UPP	IDA_macrophage_vs_M1
6,14E-17	0,71716493	0,562	0,286	2,03E-12	RPS4Y1	UPP	IDA_macrophage_vs_M1
6,57E-17	0,6145519	0,679	0,418	2,18E-12	LAMTOR1	UPP	IDA_macrophage_vs_M1
7,44E-17	0,50263662	0,946	0,785	2,46E-12	ATP5MG	UPP	IDA_macrophage_vs_M1
1,01E-16	0,38697439	0,247	0,038	3,33E-12	PLCB2	UPP	IDA_macrophage_vs_M1

1,20E-16	0,78515227	0,867	0,689	3,98E-12	SGK1	UPP	IDA_macrophage_vs_M1
1,28E-16	0,47058748	0,258	0,048	4,24E-12	MIF4GD	UPP	IDA_macrophage_vs_M1
1,42E-16	0,66490913	0,872	0,638	4,72E-12	CYBB	UPP	IDA_macrophage_vs_M1
1,44E-16	0,55588426	0,658	0,372	4,76E-12	LAMTOR2	UPP	IDA_macrophage_vs_M1
1,54E-16	0,65125954	0,764	0,504	5,09E-12	STK17B	UPP	IDA_macrophage_vs_M1
1,80E-16	0,71460842	0,796	0,539	5,96E-12	CPVL	UPP	IDA_macrophage_vs_M1
1,89E-16	0,7842734	0,293	0,071	6,27E-12	ID3	UPP	IDA_macrophage_vs_M1
1,93E-16	0,35275478	0,997	0,987	6,41E-12	RPL41	UPP	IDA_macrophage_vs_M1
1,97E-16	0,79409679	0,668	0,415	6,54E-12	IFI30	UPP	IDA_macrophage_vs_M1
2,01E-16	0,49613569	0,647	0,344	6,66E-12	SNU13	UPP	IDA_macrophage_vs_M1
2,19E-16	0,57353769	0,951	0,805	7,24E-12	JUND	UPP	IDA_macrophage_vs_M1
2,19E-16	0,60066963	0,546	0,261	7,26E-12	NAIP	UPP	IDA_macrophage_vs_M1
2,19E-16	0,52406572	0,443	0,172	7,26E-12	CAT	UPP	IDA_macrophage_vs_M1
4,48E-16	0,54265359	0,424	0,162	1,49E-11	ASGR1	UPP	IDA_macrophage_vs_M1
4,66E-16	0,57172801	0,829	0,643	1,54E-11	FCGRT	UPP	IDA_macrophage_vs_M1
5,04E-16	0,43459324	0,245	0,043	1,67E-11	P2RY6	UPP	IDA_macrophage_vs_M1
6,44E-16	0,48269558	0,288	0,066	2,13E-11	DDHD1	UPP	IDA_macrophage_vs_M1
7,30E-16	0,60600904	0,647	0,365	2,42E-11	PLEKHO1	UPP	IDA_macrophage_vs_M1
7,53E-16	0,50127957	0,685	0,42	2,49E-11	SF3B5	UPP	IDA_macrophage_vs_M1
8,69E-16	0,56257748	0,889	0,691	2,88E-11	RPL31	UPP	IDA_macrophage_vs_M1
9,30E-16	0,51283016	0,973	0,942	3,08E-11	RPL26	UPP	IDA_macrophage_vs_M1
9,65E-16	0,59039985	0,389	0,139	3,20E-11	GPR65	UPP	IDA_macrophage_vs_M1
1,07E-15	0,55318179	0,611	0,342	3,55E-11	EIF4B	UPP	IDA_macrophage_vs_M1
1,14E-15	0,580229	0,323	0,099	3,78E-11	AVPI1	UPP	IDA_macrophage_vs_M1
1,26E-15	0,46196996	0,304	0,084	4,17E-11	FRMD4B	UPP	IDA_macrophage_vs_M1
1,36E-15	0,60097064	0,459	0,197	4,51E-11	IDH2	UPP	IDA_macrophage_vs_M1
1,60E-15	0,52772893	0,573	0,278	5,30E-11	ATP5F1A	UPP	IDA_macrophage_vs_M1
1,75E-15	0,81387968	0,633	0,39	5,79E-11	MAT2A	UPP	IDA_macrophage_vs_M1
2,03E-15	0,60641474	0,723	0,453	6,74E-11	CORO1A	UPP	IDA_macrophage_vs_M1
2,05E-15	0,5293264	0,696	0,425	6,80E-11	THEMIS2	UPP	IDA_macrophage_vs_M1
2,28E-15	0,4665106	0,709	0,425	7,54E-11	RNF130	UPP	IDA_macrophage_vs_M1
2,39E-15	0,39132348	0,247	0,048	7,91E-11	CYSLTR1	UPP	IDA_macrophage_vs_M1
2,56E-15	0,9030903	0,81	0,539	8,48E-11	ZFP36L1	UPP	IDA_macrophage_vs_M1
2,57E-15	0,50971942	0,995	0,952	8,52E-11	RPS29	UPP	IDA_macrophage_vs_M1
2,88E-15	0,50047744	0,421	0,177	9,54E-11	PHB	UPP	IDA_macrophage_vs_M1
2,96E-15	0,43304276	0,421	0,165	9,81E-11	CLNS1A	UPP	IDA_macrophage_vs_M1
3,20E-15	0,53755648	0,342	0,111	1,06E-10	ADAP2	UPP	IDA_macrophage_vs_M1
3,66E-15	0,87390117	0,356	0,116	1,21E-10	HES1	UPP	IDA_macrophage_vs_M1
3,88E-15	0,43540438	0,367	0,119	1,28E-10	EVL	UPP	IDA_macrophage_vs_M1
5,31E-15	0,48252161	0,989	0,934	1,76E-10	RPS13	UPP	IDA_macrophage_vs_M1
7,30E-15	0,52895152	0,614	0,332	2,42E-10	TMEM219	UPP	IDA_macrophage_vs_M1
7,71E-15	0,55037039	0,212	0,033	2,56E-10	VSIG4	UPP	IDA_macrophage_vs_M1
8,94E-15	0,57410584	0,739	0,491	2,96E-10	ZFAND5	UPP	IDA_macrophage_vs_M1
9,07E-15	0,48861614	0,429	0,182	3,00E-10	NAGA	UPP	IDA_macrophage_vs_M1
1,09E-14	0,53847962	0,541	0,289	3,60E-10	NDUFS7	UPP	IDA_macrophage_vs_M1
1,09E-14	0,53192021	0,261	0,061	3,62E-10	PRMT9	UPP	IDA_macrophage_vs_M1
1,12E-14	0,45733835	0,505	0,22	3,72E-10	LY86	UPP	IDA_macrophage_vs_M1
1,24E-14	0,42734438	0,307	0,089	4,10E-10	RCSD1	UPP	IDA_macrophage_vs_M1
1,30E-14	0,43129214	0,31	0,094	4,30E-10	CCDC85B	UPP	IDA_macrophage_vs_M1
1,53E-14	0,4284778	0,367	0,122	5,08E-10	CD302	UPP	IDA_macrophage_vs_M1

1,56E-14	0,50666994	0,889	0,737	5,15E-10	EIF3K	UPP	IDA_macrophage_vs_M1
1,83E-14	0,56977687	0,592	0,324	6,05E-10	CELF2	UPP	IDA_macrophage_vs_M1
2,10E-14	0,40442256	0,995	0,99	6,94E-10	RPS27	UPP	IDA_macrophage_vs_M1
2,26E-14	0,30046741	0,188	0,02	7,48E-10	PALLD	UPP	IDA_macrophage_vs_M1
2,27E-14	0,46273011	0,91	0,777	7,52E-10	COX7C	UPP	IDA_macrophage_vs_M1
2,38E-14	0,36655913	0,986	0,982	7,89E-10	RPL30	UPP	IDA_macrophage_vs_M1
2,49E-14	0,46852373	0,875	0,696	8,24E-10	AP2S1	UPP	IDA_macrophage_vs_M1
2,66E-14	0,45656331	0,367	0,134	8,80E-10	C20orf27	UPP	IDA_macrophage_vs_M1
3,00E-14	0,50873677	0,255	0,061	9,93E-10	CD209	UPP	IDA_macrophage_vs_M1
3,34E-14	0,54639009	0,821	0,628	1,11E-09	ZFAS1	UPP	IDA_macrophage_vs_M1
4,00E-14	1,22531995	0,193	0,025	1,33E-09	DNASE1L3	UPP	IDA_macrophage_vs_M1
4,29E-14	0,46951284	0,486	0,238	1,42E-09	TMEM14C	UPP	IDA_macrophage_vs_M1
4,97E-14	0,688393	0,413	0,165	1,65E-09	KLF2	UPP	IDA_macrophage_vs_M1
5,04E-14	0,39880799	0,973	0,937	1,67E-09	NACA	UPP	IDA_macrophage_vs_M1
6,27E-14	0,56488992	0,641	0,392	2,08E-09	TMEM123	UPP	IDA_macrophage_vs_M1
6,54E-14	0,49983524	0,829	0,633	2,17E-09	ATP5MC3	UPP	IDA_macrophage_vs_M1
6,63E-14	0,53936515	0,296	0,089	2,20E-09	LPAR6	UPP	IDA_macrophage_vs_M1
6,89E-14	0,72487612	0,832	0,666	2,28E-09	CYCS	UPP	IDA_macrophage_vs_M1
7,00E-14	0,39441042	0,34	0,116	2,32E-09	MGST2	UPP	IDA_macrophage_vs_M1
7,02E-14	0,51199743	0,345	0,122	2,32E-09	NXT1	UPP	IDA_macrophage_vs_M1
7,33E-14	0,45402099	0,473	0,205	2,43E-09	ENTPD1	UPP	IDA_macrophage_vs_M1
7,34E-14	0,46931841	0,663	0,403	2,43E-09	NDUFA2	UPP	IDA_macrophage_vs_M1
7,68E-14	0,42384563	0,59	0,319	2,54E-09	SYNGR2	UPP	IDA_macrophage_vs_M1
7,77E-14	0,59901575	0,636	0,4	2,57E-09	CD4	UPP	IDA_macrophage_vs_M1
7,82E-14	0,3950108	0,296	0,089	2,59E-09	PMVK	UPP	IDA_macrophage_vs_M1
7,88E-14	0,42510023	0,978	0,934	2,61E-09	RPL36	UPP	IDA_macrophage_vs_M1
8,80E-14	0,77857812	0,859	0,8	2,92E-09	HLA-DRB5	UPP	IDA_macrophage_vs_M1
9,08E-14	0,5452815	0,677	0,451	3,01E-09	EIF3L	UPP	IDA_macrophage_vs_M1
1,13E-13	0,3927816	0,997	0,995	3,76E-09	MT-ATP6	UPP	IDA_macrophage_vs_M1
1,17E-13	0,55489533	0,538	0,311	3,89E-09	UFC1	UPP	IDA_macrophage_vs_M1
1,28E-13	0,59300014	0,296	0,094	4,25E-09	FAM110A	UPP	IDA_macrophage_vs_M1
1,32E-13	0,50785499	0,905	0,775	4,36E-09	GNAI2	UPP	IDA_macrophage_vs_M1
1,34E-13	0,46689261	0,467	0,228	4,44E-09	ALKBH7	UPP	IDA_macrophage_vs_M1
1,40E-13	0,51463558	0,812	0,646	4,64E-09	CAPZB	UPP	IDA_macrophage_vs_M1
1,41E-13	0,42858294	0,91	0,747	4,65E-09	HSPA8	UPP	IDA_macrophage_vs_M1
1,55E-13	0,45864958	0,978	0,919	5,13E-09	CTSS	UPP	IDA_macrophage_vs_M1
1,62E-13	0,49942113	0,321	0,106	5,37E-09	KCNK6	UPP	IDA_macrophage_vs_M1
1,83E-13	0,43804774	0,299	0,091	6,07E-09	INPP5D	UPP	IDA_macrophage_vs_M1
1,84E-13	0,41534159	0,293	0,089	6,10E-09	HVCN1	UPP	IDA_macrophage_vs_M1
1,97E-13	0,84479051	0,81	0,592	6,54E-09	CTSD	UPP	IDA_macrophage_vs_M1
2,25E-13	0,44444289	0,345	0,122	7,46E-09	GAS7	UPP	IDA_macrophage_vs_M1
2,25E-13	0,80765163	0,861	0,727	7,46E-09	SELENOK	UPP	IDA_macrophage_vs_M1
2,34E-13	0,48499353	0,481	0,246	7,74E-09	HNRNPUL1	UPP	IDA_macrophage_vs_M1
2,50E-13	0,60561205	0,851	0,668	8,29E-09	C5AR1	UPP	IDA_macrophage_vs_M1
2,56E-13	0,53126897	0,304	0,096	8,46E-09	CHCHD10	UPP	IDA_macrophage_vs_M1
2,56E-13	0,47851152	0,698	0,441	8,47E-09	ATP5F1C	UPP	IDA_macrophage_vs_M1
2,85E-13	0,54072469	0,495	0,253	9,43E-09	MYCBP2	UPP	IDA_macrophage_vs_M1
3,82E-13	1,02002573	0,715	0,496	1,26E-08	IER2	UPP	IDA_macrophage_vs_M1
3,85E-13	0,56180436	0,668	0,441	1,28E-08	NDUFA12	UPP	IDA_macrophage_vs_M1
4,43E-13	0,52585807	0,625	0,387	1,47E-08	ARL6IP4	UPP	IDA_macrophage_vs_M1

4,86E-13	0,38405403	0,22	0,046	1,61E-08	CYSLTR2	UPP	IDA_macrophage_vs_M1
5,01E-13	0,55126659	0,524	0,284	1,66E-08	YY1	UPP	IDA_macrophage_vs_M1
5,64E-13	0,51935762	0,579	0,327	1,87E-08	MFSD1	UPP	IDA_macrophage_vs_M1
5,65E-13	0,44944906	0,516	0,263	1,87E-08	NUCKS1	UPP	IDA_macrophage_vs_M1
5,79E-13	0,49842314	0,568	0,316	1,92E-08	PGLS	UPP	IDA_macrophage_vs_M1
6,17E-13	0,43971749	0,266	0,071	2,04E-08	TESC	UPP	IDA_macrophage_vs_M1
6,66E-13	0,51893461	0,698	0,468	2,21E-08	EIF3E	UPP	IDA_macrophage_vs_M1
6,79E-13	0,57868372	0,804	0,608	2,25E-08	CLEC7A	UPP	IDA_macrophage_vs_M1
7,38E-13	0,49106855	0,465	0,241	2,44E-08	SCP2	UPP	IDA_macrophage_vs_M1
8,01E-13	0,35885776	0,22	0,046	2,65E-08	LGALS3BP	UPP	IDA_macrophage_vs_M1
8,18E-13	0,56320397	0,736	0,529	2,71E-08	GNA13	UPP	IDA_macrophage_vs_M1
8,30E-13	0,56006421	0,723	0,476	2,75E-08	AHR	UPP	IDA_macrophage_vs_M1
8,60E-13	0,46765605	0,402	0,172	2,85E-08	PHACTR2	UPP	IDA_macrophage_vs_M1
9,07E-13	0,34026167	0,215	0,043	3,00E-08	FADS1	UPP	IDA_macrophage_vs_M1
9,47E-13	0,4670758	0,663	0,38	3,14E-08	COX5A	UPP	IDA_macrophage_vs_M1
9,96E-13	0,47248313	0,405	0,177	3,30E-08	SHTN1	UPP	IDA_macrophage_vs_M1
1,04E-12	0,49246189	0,609	0,357	3,44E-08	LSP1	UPP	IDA_macrophage_vs_M1
1,04E-12	0,56111707	0,595	0,362	3,46E-08	ALDH2	UPP	IDA_macrophage_vs_M1
1,10E-12	0,50545957	0,484	0,241	3,64E-08	VOPP1	UPP	IDA_macrophage_vs_M1
1,17E-12	0,5354584	0,546	0,306	3,88E-08	PRKCB	UPP	IDA_macrophage_vs_M1
1,23E-12	0,75210021	0,946	0,83	4,09E-08	DUSP1	UPP	IDA_macrophage_vs_M1
1,27E-12	0,49736215	0,867	0,729	4,22E-08	ASAH1	UPP	IDA_macrophage_vs_M1
1,32E-12	0,45425215	0,321	0,116	4,37E-08	TOB2	UPP	IDA_macrophage_vs_M1
1,66E-12	0,44953134	0,269	0,078	5,51E-08	SLCO2B1	UPP	IDA_macrophage_vs_M1
1,72E-12	0,56782459	0,582	0,352	5,69E-08	ITPR2	UPP	IDA_macrophage_vs_M1
1,82E-12	0,54814841	0,804	0,61	6,01E-08	NAP1L1	UPP	IDA_macrophage_vs_M1
1,85E-12	0,33340117	0,274	0,078	6,14E-08	SIPA1	UPP	IDA_macrophage_vs_M1
1,89E-12	0,45841799	0,769	0,501	6,25E-08	TNFSF13B	UPP	IDA_macrophage_vs_M1
2,03E-12	0,57781197	0,44	0,22	6,71E-08	ATP5F1D	UPP	IDA_macrophage_vs_M1
2,05E-12	0,39210935	0,326	0,114	6,80E-08	THAP9-AS1	UPP	IDA_macrophage_vs_M1
2,16E-12	0,49172134	0,56	0,316	7,15E-08	ATP5IF1	UPP	IDA_macrophage_vs_M1
2,24E-12	0,45174482	0,307	0,106	7,40E-08	DUT	UPP	IDA_macrophage_vs_M1
2,53E-12	0,50222449	0,899	0,709	8,39E-08	SRSF3	UPP	IDA_macrophage_vs_M1
2,63E-12	0,47913019	0,682	0,405	8,72E-08	PHACTR1	UPP	IDA_macrophage_vs_M1
2,97E-12	0,47440739	0,37	0,154	9,85E-08	TRABD	UPP	IDA_macrophage_vs_M1
3,28E-12	0,49413961	0,47	0,238	1,09E-07	AKAP9	UPP	IDA_macrophage_vs_M1
3,30E-12	0,51976543	0,649	0,4	1,09E-07	TACC1	UPP	IDA_macrophage_vs_M1
3,51E-12	0,483929	0,535	0,273	1,16E-07	CD36	UPP	IDA_macrophage_vs_M1
4,15E-12	0,4503149	0,552	0,314	1,38E-07	DOK2	UPP	IDA_macrophage_vs_M1
4,40E-12	0,4239387	0,386	0,165	1,46E-07	RTCB	UPP	IDA_macrophage_vs_M1
4,60E-12	0,32089558	0,204	0,041	1,52E-07	PRAM1	UPP	IDA_macrophage_vs_M1
5,47E-12	0,31403849	0,234	0,058	1,81E-07	SPECC1	UPP	IDA_macrophage_vs_M1
5,82E-12	0,37998532	0,394	0,167	1,93E-07	APBB1IP	UPP	IDA_macrophage_vs_M1
6,57E-12	0,55278947	0,56	0,324	2,17E-07	GABARAPL:	UPP	IDA_macrophage_vs_M1
6,58E-12	0,36395369	0,329	0,119	2,18E-07	PAXX	UPP	IDA_macrophage_vs_M1
6,98E-12	0,36411365	0,28	0,091	2,31E-07	TRAPPC6A	UPP	IDA_macrophage_vs_M1
7,18E-12	0,27374003	0,149	0,015	2,38E-07	LINC00996	UPP	IDA_macrophage_vs_M1
7,92E-12	0,30360436	1	0,992	2,62E-07	TPT1	UPP	IDA_macrophage_vs_M1
8,45E-12	0,44253138	0,318	0,122	2,80E-07	SASH1	UPP	IDA_macrophage_vs_M1
8,63E-12	0,37255704	0,37	0,154	2,86E-07	ARHGAP4	UPP	IDA_macrophage_vs_M1

8,80E-12	0,50822297	0,87	0,747	2,91E-07	GRN	UPP	IDA_macrophage_vs_M1
8,82E-12	0,53011297	0,318	0,119	2,92E-07	RRP12	UPP	IDA_macrophage_vs_M1
8,97E-12	0,42223464	0,519	0,278	2,97E-07	TIMM13	UPP	IDA_macrophage_vs_M1
9,11E-12	0,35642152	1	0,997	3,02E-07	TMSB10	UPP	IDA_macrophage_vs_M1
9,22E-12	0,450083	0,337	0,137	3,06E-07	COMMD9	UPP	IDA_macrophage_vs_M1
9,48E-12	0,66653679	0,524	0,294	3,14E-07	TAGAP	UPP	IDA_macrophage_vs_M1
9,64E-12	0,27276442	0,236	0,061	3,19E-07	FDFT1	UPP	IDA_macrophage_vs_M1
1,00E-11	0,29313242	0,223	0,053	3,32E-07	MAPRE2	UPP	IDA_macrophage_vs_M1
1,03E-11	0,40308148	0,217	0,051	3,42E-07	JMY	UPP	IDA_macrophage_vs_M1
1,04E-11	0,39986938	0,973	0,947	3,46E-07	HLA-C	UPP	IDA_macrophage_vs_M1
1,24E-11	0,29548814	0,201	0,041	4,10E-07	AIG1	UPP	IDA_macrophage_vs_M1
1,39E-11	0,46061572	0,378	0,159	4,62E-07	GRASP	UPP	IDA_macrophage_vs_M1
1,48E-11	0,3200488	0,215	0,051	4,91E-07	RNF166	UPP	IDA_macrophage_vs_M1
1,58E-11	0,84147182	0,671	0,425	5,25E-07	DUSP2	UPP	IDA_macrophage_vs_M1
1,68E-11	0,44583983	0,353	0,149	5,57E-07	C1QBP	UPP	IDA_macrophage_vs_M1
1,71E-11	0,66574152	0,609	0,362	5,67E-07	TFRC	UPP	IDA_macrophage_vs_M1
1,80E-11	0,3491588	0,193	0,041	5,96E-07	GIMAP8	UPP	IDA_macrophage_vs_M1
1,82E-11	0,41553363	0,503	0,266	6,03E-07	FUOM	UPP	IDA_macrophage_vs_M1
1,85E-11	0,35480757	0,209	0,051	6,13E-07	GIMAP1	UPP	IDA_macrophage_vs_M1
2,01E-11	0,45489329	0,19	0,038	6,67E-07	FUCA1	UPP	IDA_macrophage_vs_M1
2,05E-11	0,57563606	0,603	0,375	6,81E-07	RUNX3	UPP	IDA_macrophage_vs_M1
2,15E-11	0,39855163	0,345	0,139	7,12E-07	ATM	UPP	IDA_macrophage_vs_M1
2,16E-11	0,41216966	0,356	0,147	7,14E-07	MAP3K1	UPP	IDA_macrophage_vs_M1
2,19E-11	0,48629451	0,486	0,258	7,24E-07	NAA38	UPP	IDA_macrophage_vs_M1
2,25E-11	0,37703951	0,231	0,061	7,45E-07	CD72	UPP	IDA_macrophage_vs_M1
2,32E-11	0,28860267	0,231	0,063	7,70E-07	TNFAIP8L2	UPP	IDA_macrophage_vs_M1
2,40E-11	0,31366822	0,28	0,091	7,96E-07	TGFBR2	UPP	IDA_macrophage_vs_M1
2,45E-11	0,33489416	0,989	0,911	8,12E-07	RPL24	UPP	IDA_macrophage_vs_M1
2,53E-11	0,3772635	0,832	0,597	8,40E-07	TYMP	UPP	IDA_macrophage_vs_M1
2,92E-11	0,3156399	0,234	0,061	9,68E-07	C3orf58	UPP	IDA_macrophage_vs_M1
2,93E-11	0,45731694	0,734	0,496	9,70E-07	TKT	UPP	IDA_macrophage_vs_M1
2,94E-11	0,42041417	0,378	0,167	9,72E-07	UQCC2	UPP	IDA_macrophage_vs_M1
2,94E-11	0,37416756	0,978	0,942	9,75E-07	RPS15A	UPP	IDA_macrophage_vs_M1
3,28E-11	0,38927894	0,465	0,23	1,09E-06	PPP1CC	UPP	IDA_macrophage_vs_M1
3,40E-11	0,32609847	0,992	0,965	1,13E-06	UBC	UPP	IDA_macrophage_vs_M1
3,57E-11	1,64855502	0,147	0,018	1,18E-06	SELENOP	UPP	IDA_macrophage_vs_M1
3,80E-11	0,39523527	0,47	0,246	1,26E-06	CD46	UPP	IDA_macrophage_vs_M1
3,92E-11	0,43184867	0,413	0,2	1,30E-06	PLBD1	UPP	IDA_macrophage_vs_M1
4,39E-11	0,5659789	0,519	0,291	1,45E-06	TIPARP	UPP	IDA_macrophage_vs_M1
4,59E-11	0,43877353	0,557	0,337	1,52E-06	NDUFB5	UPP	IDA_macrophage_vs_M1
4,74E-11	0,43772532	0,372	0,167	1,57E-06	IL18BP	UPP	IDA_macrophage_vs_M1
5,14E-11	0,33008158	0,266	0,089	1,70E-06	DNPEP	UPP	IDA_macrophage_vs_M1
5,37E-11	0,48477981	0,788	0,58	1,78E-06	GDI2	UPP	IDA_macrophage_vs_M1
5,49E-11	0,48809822	0,878	0,714	1,82E-06	CTSZ	UPP	IDA_macrophage_vs_M1
5,69E-11	0,31526274	0,239	0,068	1,88E-06	BTN3A2	UPP	IDA_macrophage_vs_M1
5,86E-11	0,41672102	0,424	0,208	1,94E-06	CORO1B	UPP	IDA_macrophage_vs_M1
5,90E-11	0,38880102	0,44	0,223	1,95E-06	SNRPC	UPP	IDA_macrophage_vs_M1
6,13E-11	0,42123456	0,413	0,203	2,03E-06	TXN2	UPP	IDA_macrophage_vs_M1
6,29E-11	0,44909238	0,418	0,203	2,08E-06	DNMT1	UPP	IDA_macrophage_vs_M1
6,32E-11	0,43857883	0,424	0,2	2,09E-06	SEC11C	UPP	IDA_macrophage_vs_M1

6,34E-11	0,7729555	0,696	0,539	2,10E-06	STAT1	UPP	IDA_macrophage_vs_M1
6,45E-11	0,2758207	0,149	0,02	2,14E-06	KCNMA1	UPP	IDA_macrophage_vs_M1
6,66E-11	0,3747262	0,399	0,187	2,21E-06	MLEC	UPP	IDA_macrophage_vs_M1
6,88E-11	0,35648384	0,997	0,99	2,28E-06	MT-CO3	UPP	IDA_macrophage_vs_M1
7,39E-11	0,4312843	0,606	0,375	2,45E-06	PRDX5	UPP	IDA_macrophage_vs_M1
7,85E-11	0,2962492	0,228	0,063	2,60E-06	CASS4	UPP	IDA_macrophage_vs_M1
8,03E-11	0,45069461	0,679	0,478	2,66E-06	ATP5PO	UPP	IDA_macrophage_vs_M1
8,55E-11	0,54054329	0,465	0,261	2,83E-06	IDI1	UPP	IDA_macrophage_vs_M1
8,67E-11	0,44855454	0,66	0,42	2,87E-06	GLIPR1	UPP	IDA_macrophage_vs_M1
8,76E-11	0,39929661	0,399	0,18	2,90E-06	ARID1A	UPP	IDA_macrophage_vs_M1
8,83E-11	0,42003748	0,486	0,251	2,93E-06	LIPA	UPP	IDA_macrophage_vs_M1
8,93E-11	0,49329441	0,386	0,187	2,96E-06	NOP53	UPP	IDA_macrophage_vs_M1
9,11E-11	0,43573889	0,821	0,643	3,02E-06	PARK7	UPP	IDA_macrophage_vs_M1
9,48E-11	0,46460802	0,641	0,423	3,14E-06	MIDN	UPP	IDA_macrophage_vs_M1
9,65E-11	0,54787407	0,44	0,228	3,20E-06	DDIT3	UPP	IDA_macrophage_vs_M1
1,03E-10	0,32233476	0,462	0,225	3,41E-06	PLAC8	UPP	IDA_macrophage_vs_M1
1,07E-10	0,31865403	0,302	0,109	3,54E-06	IL18	UPP	IDA_macrophage_vs_M1
1,07E-10	0,33167122	0,288	0,101	3,55E-06	LMO2	UPP	IDA_macrophage_vs_M1
1,15E-10	0,38114457	0,399	0,187	3,80E-06	SNX5	UPP	IDA_macrophage_vs_M1
1,18E-10	0,39441362	0,967	0,914	3,91E-06	RPS21	UPP	IDA_macrophage_vs_M1
1,22E-10	0,4039978	0,424	0,215	4,03E-06	TMEM147	UPP	IDA_macrophage_vs_M1
1,26E-10	0,47719329	0,867	0,734	4,19E-06	NDUFA4	UPP	IDA_macrophage_vs_M1
1,29E-10	0,35279658	0,166	0,03	4,28E-06	SDC3	UPP	IDA_macrophage_vs_M1
1,30E-10	0,41544938	0,47	0,253	4,31E-06	REX1BD	UPP	IDA_macrophage_vs_M1
1,31E-10	0,43402451	0,745	0,537	4,33E-06	NCL	UPP	IDA_macrophage_vs_M1
1,36E-10	0,45492246	0,364	0,165	4,50E-06	HEXA	UPP	IDA_macrophage_vs_M1
1,47E-10	0,44832236	0,636	0,423	4,88E-06	SNHG8	UPP	IDA_macrophage_vs_M1
1,53E-10	0,40922415	0,549	0,327	5,07E-06	NCKAP1L	UPP	IDA_macrophage_vs_M1
1,54E-10	0,42236087	0,391	0,182	5,10E-06	LIMD2	UPP	IDA_macrophage_vs_M1
1,57E-10	0,40539128	0,598	0,359	5,21E-06	WAS	UPP	IDA_macrophage_vs_M1
1,69E-10	0,45457159	0,668	0,438	5,59E-06	RPL22L1	UPP	IDA_macrophage_vs_M1
1,74E-10	0,69281163	0,753	0,587	5,77E-06	NR4A3	UPP	IDA_macrophage_vs_M1
1,75E-10	0,31059931	0,253	0,081	5,80E-06	TMED3	UPP	IDA_macrophage_vs_M1
1,79E-10	0,43488485	0,446	0,233	5,92E-06	NDEL1	UPP	IDA_macrophage_vs_M1
1,81E-10	0,31795853	0,976	0,97	6,01E-06	RPL35A	UPP	IDA_macrophage_vs_M1
1,82E-10	0,37397131	0,28	0,104	6,03E-06	MGMT	UPP	IDA_macrophage_vs_M1
1,82E-10	0,47118337	0,576	0,324	6,04E-06	RARRES3	UPP	IDA_macrophage_vs_M1
1,92E-10	0,32866075	0,318	0,127	6,36E-06	ATXN10	UPP	IDA_macrophage_vs_M1
1,96E-10	0,42675659	0,766	0,565	6,49E-06	EIF3A	UPP	IDA_macrophage_vs_M1
2,00E-10	0,38598934	0,484	0,263	6,64E-06	RSL1D1	UPP	IDA_macrophage_vs_M1
2,03E-10	0,38915042	0,584	0,354	6,71E-06	NDUFB10	UPP	IDA_macrophage_vs_M1
2,04E-10	0,42361325	0,53	0,319	6,76E-06	ATP5MC1	UPP	IDA_macrophage_vs_M1
2,11E-10	0,33403209	0,274	0,099	7,00E-06	CBR1	UPP	IDA_macrophage_vs_M1
2,17E-10	0,46985072	0,457	0,238	7,18E-06	RAB11FIP1	UPP	IDA_macrophage_vs_M1
2,27E-10	0,37564848	0,454	0,243	7,52E-06	ETFB	UPP	IDA_macrophage_vs_M1
2,32E-10	0,37081434	0,81	0,615	7,69E-06	BST2	UPP	IDA_macrophage_vs_M1
2,32E-10	0,32256607	0,304	0,114	7,70E-06	WBP1L	UPP	IDA_macrophage_vs_M1
2,33E-10	0,27002266	0,19	0,043	7,71E-06	LRMP	UPP	IDA_macrophage_vs_M1
2,39E-10	0,38548087	0,353	0,154	7,90E-06	NUDT1	UPP	IDA_macrophage_vs_M1
2,40E-10	0,56531914	0,543	0,329	7,95E-06	ARL4A	UPP	IDA_macrophage_vs_M1

2,51E-10	0,47485731	0,408	0,2	8,31E-06	LYST	UPP	IDA_macrophage_vs_M1
2,61E-10	0,40842215	0,329	0,142	8,64E-06	SDSL	UPP	IDA_macrophage_vs_M1
2,64E-10	0,41150932	0,856	0,711	8,75E-06	HCST	UPP	IDA_macrophage_vs_M1
3,07E-10	0,30389257	0,196	0,048	1,02E-05	BORCS5	UPP	IDA_macrophage_vs_M1
3,28E-10	0,30345297	0,995	0,982	1,09E-05	RPS28	UPP	IDA_macrophage_vs_M1
3,30E-10	0,3832367	0,823	0,653	1,09E-05	UQCR10	UPP	IDA_macrophage_vs_M1
3,42E-10	0,4537432	0,891	0,805	1,13E-05	HNRNPU	UPP	IDA_macrophage_vs_M1
3,46E-10	0,46370233	0,579	0,354	1,15E-05	ACP5	UPP	IDA_macrophage_vs_M1
3,61E-10	0,42853161	0,606	0,392	1,19E-05	JTB	UPP	IDA_macrophage_vs_M1
3,68E-10	0,27877396	0,228	0,068	1,22E-05	AHCY	UPP	IDA_macrophage_vs_M1
3,75E-10	0,56048314	0,707	0,491	1,24E-05	UCP2	UPP	IDA_macrophage_vs_M1
3,88E-10	0,37607634	0,323	0,132	1,29E-05	GUSB	UPP	IDA_macrophage_vs_M1
3,91E-10	0,39567085	0,467	0,266	1,29E-05	RNASE6	UPP	IDA_macrophage_vs_M1
4,17E-10	0,40939662	0,413	0,208	1,38E-05	LDHB	UPP	IDA_macrophage_vs_M1
4,30E-10	0,90204109	0,717	0,539	1,43E-05	PLIN2	UPP	IDA_macrophage_vs_M1
4,36E-10	0,35688941	0,207	0,056	1,44E-05	TMEM170E	UPP	IDA_macrophage_vs_M1
4,40E-10	0,37262618	0,5	0,278	1,46E-05	SCPEP1	UPP	IDA_macrophage_vs_M1
4,72E-10	0,41881917	0,557	0,339	1,56E-05	MDH2	UPP	IDA_macrophage_vs_M1
5,02E-10	0,36234853	0,25	0,086	1,66E-05	NUDT16	UPP	IDA_macrophage_vs_M1
5,02E-10	0,39385555	0,484	0,261	1,66E-05	MRPS21	UPP	IDA_macrophage_vs_M1
5,03E-10	0,58352272	0,421	0,22	1,67E-05	ATP1B1	UPP	IDA_macrophage_vs_M1
5,25E-10	0,47530663	0,519	0,329	1,74E-05	ANP32B	UPP	IDA_macrophage_vs_M1
5,36E-10	0,27033173	0,163	0,03	1,78E-05	PLA2G16	UPP	IDA_macrophage_vs_M1
6,29E-10	0,35662742	0,625	0,375	2,08E-05	sep-09	UPP	IDA_macrophage_vs_M1
6,51E-10	0,46048909	0,568	0,38	2,16E-05	BANF1	UPP	IDA_macrophage_vs_M1
6,66E-10	0,37488147	0,742	0,519	2,21E-05	JMJD1C	UPP	IDA_macrophage_vs_M1
6,75E-10	0,36909918	0,44	0,233	2,23E-05	SPG21	UPP	IDA_macrophage_vs_M1
6,83E-10	0,36328587	0,299	0,122	2,26E-05	CCDC12	UPP	IDA_macrophage_vs_M1
7,71E-10	0,32174861	0,495	0,276	2,55E-05	SUPT4H1	UPP	IDA_macrophage_vs_M1
7,76E-10	0,27926074	0,391	0,17	2,57E-05	SORL1	UPP	IDA_macrophage_vs_M1
8,16E-10	0,37395049	0,859	0,722	2,70E-05	FOSL2	UPP	IDA_macrophage_vs_M1
8,19E-10	0,39002835	0,549	0,324	2,71E-05	POLE4	UPP	IDA_macrophage_vs_M1
8,34E-10	0,33140117	0,361	0,165	2,76E-05	PEPD	UPP	IDA_macrophage_vs_M1
9,94E-10	0,26730375	0,133	0,018	3,29E-05	GPR155	UPP	IDA_macrophage_vs_M1
1,05E-09	0,35368241	0,321	0,129	3,49E-05	CALHM6	UPP	IDA_macrophage_vs_M1
1,09E-09	0,41499145	0,481	0,258	3,61E-05	BCAS2	UPP	IDA_macrophage_vs_M1
1,12E-09	0,41179037	0,802	0,651	3,71E-05	FAM49B	UPP	IDA_macrophage_vs_M1
1,13E-09	0,32467745	0,261	0,094	3,75E-05	CORO7	UPP	IDA_macrophage_vs_M1
1,29E-09	0,40796923	0,59	0,37	4,27E-05	SNX2	UPP	IDA_macrophage_vs_M1
1,31E-09	0,38604392	0,856	0,734	4,33E-05	TOMM7	UPP	IDA_macrophage_vs_M1
1,44E-09	0,35984655	0,598	0,362	4,78E-05	FIS1	UPP	IDA_macrophage_vs_M1
1,59E-09	0,31247095	0,261	0,096	5,25E-05	ARRDC1	UPP	IDA_macrophage_vs_M1
1,64E-09	0,33243941	0,264	0,099	5,43E-05	ARRDC2	UPP	IDA_macrophage_vs_M1
1,65E-09	0,40492137	0,367	0,177	5,45E-05	FAM53C	UPP	IDA_macrophage_vs_M1
1,71E-09	0,43870832	0,709	0,499	5,67E-05	SET	UPP	IDA_macrophage_vs_M1
1,77E-09	0,33350701	0,304	0,129	5,85E-05	DUSP23	UPP	IDA_macrophage_vs_M1
1,94E-09	0,38872708	0,334	0,154	6,42E-05	SCLT1	UPP	IDA_macrophage_vs_M1
1,97E-09	0,28064489	0,207	0,061	6,52E-05	RPS6KA4	UPP	IDA_macrophage_vs_M1
2,05E-09	0,43011386	0,508	0,311	6,80E-05	H2AFV	UPP	IDA_macrophage_vs_M1
2,14E-09	0,30847831	0,247	0,091	7,09E-05	POP5	UPP	IDA_macrophage_vs_M1

2,16E-09	0,26806211	0,372	0,182	7,17E-05	LSM4	UPP	IDA_macrophage_vs_M1
2,21E-09	0,41167261	0,486	0,301	7,32E-05	PARVG	UPP	IDA_macrophage_vs_M1
2,22E-09	0,35988103	0,421	0,213	7,36E-05	MEF2C	UPP	IDA_macrophage_vs_M1
2,27E-09	0,36850227	0,386	0,187	7,52E-05	LRRC25	UPP	IDA_macrophage_vs_M1
2,33E-09	0,37620095	0,364	0,18	7,72E-05	MAF1	UPP	IDA_macrophage_vs_M1
2,42E-09	0,38903553	0,253	0,096	8,01E-05	CMKLR1	UPP	IDA_macrophage_vs_M1
2,43E-09	0,31397632	0,508	0,273	8,06E-05	C19orf70	UPP	IDA_macrophage_vs_M1
2,58E-09	0,46180705	0,734	0,57	8,54E-05	PRR13	UPP	IDA_macrophage_vs_M1
2,66E-09	0,35653232	0,897	0,747	8,81E-05	PSME2	UPP	IDA_macrophage_vs_M1
2,71E-09	0,29716418	0,739	0,519	8,97E-05	GLRX	UPP	IDA_macrophage_vs_M1
2,91E-09	0,29090459	0,215	0,068	9,64E-05	EIF2AK4	UPP	IDA_macrophage_vs_M1
2,99E-09	0,40426001	0,807	0,613	9,92E-05	MBNL1	UPP	IDA_macrophage_vs_M1
3,18E-09	0,31409293	0,307	0,134	0,000105	NME3	UPP	IDA_macrophage_vs_M1
3,24E-09	0,39995787	0,399	0,215	0,000107	ECHS1	UPP	IDA_macrophage_vs_M1
3,33E-09	0,392968	0,731	0,519	0,00011	SEC11A	UPP	IDA_macrophage_vs_M1
3,60E-09	0,35384402	0,351	0,162	0,000119	RASSF2	UPP	IDA_macrophage_vs_M1
3,66E-09	0,33285893	0,334	0,157	0,000121	LNPEP	UPP	IDA_macrophage_vs_M1
4,00E-09	0,3303823	0,467	0,256	0,000132	CIITA	UPP	IDA_macrophage_vs_M1
4,02E-09	0,3185985	0,307	0,132	0,000133	AKR1B1	UPP	IDA_macrophage_vs_M1
4,32E-09	0,41236588	0,552	0,38	0,000143	SF1	UPP	IDA_macrophage_vs_M1
4,35E-09	0,30753366	0,234	0,078	0,000144	CAMK1D	UPP	IDA_macrophage_vs_M1
4,49E-09	0,35547057	0,465	0,258	0,000149	HNRNPAB	UPP	IDA_macrophage_vs_M1
4,65E-09	0,39613244	0,334	0,154	0,000154	NGLY1	UPP	IDA_macrophage_vs_M1
4,76E-09	0,32538615	0,302	0,134	0,000158	NDUFS3	UPP	IDA_macrophage_vs_M1
4,77E-09	0,42148399	0,37	0,187	0,000158	PLEKHA2	UPP	IDA_macrophage_vs_M1
4,83E-09	0,39169386	0,47	0,266	0,00016	PHB2	UPP	IDA_macrophage_vs_M1
4,85E-09	0,44437635	0,742	0,539	0,000161	UQCRQ	UPP	IDA_macrophage_vs_M1
4,89E-09	0,35869831	0,707	0,506	0,000162	SRRM1	UPP	IDA_macrophage_vs_M1
4,93E-09	0,36164438	0,359	0,175	0,000163	NEMF	UPP	IDA_macrophage_vs_M1
4,93E-09	0,33475585	0,318	0,142	0,000163	DNPH1	UPP	IDA_macrophage_vs_M1
5,02E-09	0,46693799	0,565	0,365	0,000166	NDUFA3	UPP	IDA_macrophage_vs_M1
5,10E-09	0,44914543	0,636	0,43	0,000169	EID1	UPP	IDA_macrophage_vs_M1
5,47E-09	0,39777759	0,438	0,235	0,000181	GTF3A	UPP	IDA_macrophage_vs_M1
5,75E-09	0,36626169	0,516	0,314	0,000191	TUFM	UPP	IDA_macrophage_vs_M1
5,79E-09	0,39052182	0,495	0,289	0,000192	TAF1D	UPP	IDA_macrophage_vs_M1
6,08E-09	0,4192511	0,505	0,281	0,000201	TNFSF10	UPP	IDA_macrophage_vs_M1
6,20E-09	0,39884275	0,484	0,284	0,000205	RB1	UPP	IDA_macrophage_vs_M1
6,28E-09	0,34208739	0,198	0,061	0,000208	OFD1	UPP	IDA_macrophage_vs_M1
6,48E-09	0,34225304	0,242	0,091	0,000215	TMEM160	UPP	IDA_macrophage_vs_M1
6,54E-09	0,34852464	0,856	0,701	0,000217	SSR4	UPP	IDA_macrophage_vs_M1
6,62E-09	0,28956707	0,312	0,139	0,000219	CENPX	UPP	IDA_macrophage_vs_M1
6,77E-09	0,38272464	0,299	0,134	0,000224	CD33	UPP	IDA_macrophage_vs_M1
6,84E-09	0,34648275	0,402	0,213	0,000227	DDX27	UPP	IDA_macrophage_vs_M1
7,09E-09	0,44747689	0,658	0,461	0,000235	CYTIP	UPP	IDA_macrophage_vs_M1
7,16E-09	0,36231124	0,274	0,111	0,000237	GNL3	UPP	IDA_macrophage_vs_M1
7,60E-09	0,36088873	0,291	0,129	0,000252	DNAJC10	UPP	IDA_macrophage_vs_M1
8,11E-09	0,28250887	0,307	0,132	0,000269	FES	UPP	IDA_macrophage_vs_M1
8,26E-09	0,3264484	0,337	0,157	0,000274	NIN	UPP	IDA_macrophage_vs_M1
8,44E-09	0,34041525	0,345	0,165	0,00028	FGD2	UPP	IDA_macrophage_vs_M1
8,51E-09	0,38906963	0,37	0,187	0,000282	AP1B1	UPP	IDA_macrophage_vs_M1

8,53E-09	0,38094236	0,562	0,362	0,000283	PPIG	UPP	IDA_macrophage_vs_M1
8,65E-09	0,29397384	0,329	0,149	0,000287	PCBD1	UPP	IDA_macrophage_vs_M1
8,70E-09	0,34572436	0,37	0,182	0,000288	CDK2AP2	UPP	IDA_macrophage_vs_M1
8,78E-09	0,26502109	0,163	0,038	0,000291	SIGLEC1	UPP	IDA_macrophage_vs_M1
9,18E-09	0,314153	0,416	0,22	0,000304	NDUFV3	UPP	IDA_macrophage_vs_M1
9,27E-09	0,28169112	0,152	0,033	0,000307	CACNA2D3	UPP	IDA_macrophage_vs_M1
9,81E-09	0,28972826	0,353	0,172	0,000325	COMMD1	UPP	IDA_macrophage_vs_M1
1,00E-08	0,43903107	0,562	0,382	0,000332	GNAQ	UPP	IDA_macrophage_vs_M1
1,01E-08	0,54357377	0,56	0,349	0,000335	FCGR3A	UPP	IDA_macrophage_vs_M1
1,02E-08	0,26754627	0,481	0,256	0,000339	NCF1	UPP	IDA_macrophage_vs_M1
1,03E-08	0,39614211	0,383	0,2	0,000342	SULF2	UPP	IDA_macrophage_vs_M1
1,04E-08	0,39451163	0,421	0,235	0,000343	TTC3	UPP	IDA_macrophage_vs_M1
1,08E-08	0,41071996	0,611	0,403	0,000356	ATP5PB	UPP	IDA_macrophage_vs_M1
1,14E-08	0,3668238	0,72	0,509	0,000379	APRT	UPP	IDA_macrophage_vs_M1
1,15E-08	0,29076433	0,304	0,139	0,000381	GRHPR	UPP	IDA_macrophage_vs_M1
1,15E-08	0,35151582	0,329	0,157	0,000382	NDUFV1	UPP	IDA_macrophage_vs_M1
1,18E-08	0,42217578	0,644	0,415	0,000391	PHPT1	UPP	IDA_macrophage_vs_M1
1,19E-08	0,3005637	0,253	0,099	0,000395	RNASEH2B	UPP	IDA_macrophage_vs_M1
1,25E-08	0,48723706	0,329	0,162	0,000413	NRG1	UPP	IDA_macrophage_vs_M1
1,25E-08	0,39354062	0,446	0,258	0,000414	PNN	UPP	IDA_macrophage_vs_M1
1,32E-08	0,45232857	0,986	0,959	0,000439	LYZ	UPP	IDA_macrophage_vs_M1
1,34E-08	0,40562531	0,329	0,154	0,000442	SLA	UPP	IDA_macrophage_vs_M1
1,45E-08	0,36286002	0,421	0,233	0,000479	PFDN1	UPP	IDA_macrophage_vs_M1
1,49E-08	0,27476095	0,239	0,091	0,000492	ARL2	UPP	IDA_macrophage_vs_M1
1,57E-08	0,35898092	0,747	0,585	0,000522	SERBP1	UPP	IDA_macrophage_vs_M1
1,63E-08	0,39351255	0,601	0,387	0,000539	NDUFA6	UPP	IDA_macrophage_vs_M1
1,64E-08	0,28977259	0,318	0,144	0,000543	PQBP1	UPP	IDA_macrophage_vs_M1
1,65E-08	0,39248004	0,435	0,248	0,000548	TPR	UPP	IDA_macrophage_vs_M1
1,66E-08	0,26615705	0,177	0,048	0,000549	BAZ1B	UPP	IDA_macrophage_vs_M1
1,72E-08	0,36344395	0,823	0,716	0,000569	PSME1	UPP	IDA_macrophage_vs_M1
1,79E-08	0,35855824	0,459	0,261	0,000595	DDX3Y	UPP	IDA_macrophage_vs_M1
1,81E-08	0,30037733	0,242	0,089	0,000599	CD38	UPP	IDA_macrophage_vs_M1
1,91E-08	0,38167781	0,807	0,643	0,000632	COMMD6	UPP	IDA_macrophage_vs_M1
1,96E-08	0,34934075	0,378	0,197	0,000651	BAG1	UPP	IDA_macrophage_vs_M1
2,13E-08	0,33062972	0,443	0,241	0,000705	RWDD1	UPP	IDA_macrophage_vs_M1
2,14E-08	0,32346882	0,677	0,501	0,000708	PPT1	UPP	IDA_macrophage_vs_M1
2,15E-08	0,3195154	0,345	0,17	0,000711	MRPL11	UPP	IDA_macrophage_vs_M1
2,26E-08	0,31497155	0,992	0,977	0,000749	RPL34	UPP	IDA_macrophage_vs_M1
2,31E-08	0,38322984	0,649	0,471	0,000766	ATP5PD	UPP	IDA_macrophage_vs_M1
2,40E-08	0,41079872	0,402	0,225	0,000795	RASSF3	UPP	IDA_macrophage_vs_M1
2,41E-08	0,31095971	0,226	0,081	0,000799	ZNF672	UPP	IDA_macrophage_vs_M1
2,43E-08	0,28769479	0,25	0,096	0,000806	SDE2	UPP	IDA_macrophage_vs_M1
2,46E-08	0,34356325	0,497	0,286	0,000815	EVI2B	UPP	IDA_macrophage_vs_M1
2,59E-08	0,28361655	0,198	0,063	0,000858	TOR2A	UPP	IDA_macrophage_vs_M1
2,63E-08	0,28527035	0,274	0,116	0,00087	SCNM1	UPP	IDA_macrophage_vs_M1
2,67E-08	0,44709153	0,361	0,187	0,000884	YWHAH	UPP	IDA_macrophage_vs_M1
2,75E-08	0,31307369	0,193	0,061	0,000912	METTL7A	UPP	IDA_macrophage_vs_M1
2,84E-08	0,38042717	0,584	0,395	0,00094	SNRPD2	UPP	IDA_macrophage_vs_M1
2,88E-08	0,31008883	0,519	0,314	0,000953	DPP7	UPP	IDA_macrophage_vs_M1
2,93E-08	0,40367781	0,234	0,089	0,000972	CCND2	UPP	IDA_macrophage_vs_M1

2,96E-08	0,34673086	0,261	0,106	0,000979	TTC19	UPP	IDA_macrophage_vs_M1
3,09E-08	0,36297793	0,337	0,162	0,001022	BDP1	UPP	IDA_macrophage_vs_M1
3,17E-08	0,29672077	0,356	0,175	0,001051	ZCRB1	UPP	IDA_macrophage_vs_M1
3,19E-08	0,33752282	0,41	0,235	0,001058	NDUFS2	UPP	IDA_macrophage_vs_M1
3,21E-08	0,30277166	0,255	0,106	0,001062	ELMO1	UPP	IDA_macrophage_vs_M1
3,29E-08	0,32331319	0,47	0,289	0,001091	SDHB	UPP	IDA_macrophage_vs_M1
3,30E-08	0,34969954	0,454	0,268	0,001092	RGS19	UPP	IDA_macrophage_vs_M1
3,45E-08	0,44666114	0,595	0,397	0,001142	BLVRB	UPP	IDA_macrophage_vs_M1
3,56E-08	0,36176183	0,538	0,344	0,001178	MRPL54	UPP	IDA_macrophage_vs_M1
3,56E-08	0,38548854	0,709	0,549	0,00118	ELF1	UPP	IDA_macrophage_vs_M1
3,60E-08	0,35326823	0,443	0,268	0,001192	MRPL57	UPP	IDA_macrophage_vs_M1
3,70E-08	0,34211961	0,552	0,354	0,001225	RAB8A	UPP	IDA_macrophage_vs_M1
3,71E-08	0,26607918	0,204	0,068	0,001227	DELE1	UPP	IDA_macrophage_vs_M1
3,78E-08	0,31523234	0,418	0,225	0,001252	MRPL21	UPP	IDA_macrophage_vs_M1
3,83E-08	0,50304576	0,321	0,154	0,001268	ABHD5	UPP	IDA_macrophage_vs_M1
4,31E-08	0,32794092	0,334	0,167	0,001426	COA4	UPP	IDA_macrophage_vs_M1
4,47E-08	0,32746057	0,367	0,197	0,001481	C9orf78	UPP	IDA_macrophage_vs_M1
4,49E-08	0,64109266	0,372	0,203	0,001488	SNAI1	UPP	IDA_macrophage_vs_M1
4,51E-08	0,27363922	0,196	0,066	0,001494	ANKRD13D	UPP	IDA_macrophage_vs_M1
4,62E-08	0,40593965	0,288	0,127	0,00153	CXorf21	UPP	IDA_macrophage_vs_M1
4,62E-08	0,31635339	0,291	0,124	0,00153	LRRK2	UPP	IDA_macrophage_vs_M1
4,84E-08	0,36115917	0,285	0,132	0,001603	EPRS	UPP	IDA_macrophage_vs_M1
4,95E-08	0,29118306	0,255	0,104	0,001638	SF3A3	UPP	IDA_macrophage_vs_M1
5,01E-08	0,3019991	0,978	0,906	0,001659	MCL1	UPP	IDA_macrophage_vs_M1
5,09E-08	0,30868003	0,476	0,289	0,001685	PRPF38B	UPP	IDA_macrophage_vs_M1
5,40E-08	0,36807331	0,595	0,397	0,001789	TBXAS1	UPP	IDA_macrophage_vs_M1
5,42E-08	0,31940751	0,644	0,425	0,001795	NDUFB8	UPP	IDA_macrophage_vs_M1
5,49E-08	0,35270326	0,582	0,39	0,001819	EIF3M	UPP	IDA_macrophage_vs_M1
5,50E-08	0,27743793	0,245	0,096	0,001821	EXOC3	UPP	IDA_macrophage_vs_M1
5,77E-08	0,30345417	0,609	0,405	0,001911	NDUFB7	UPP	IDA_macrophage_vs_M1
6,09E-08	0,35721281	0,535	0,352	0,002016	PSMC5	UPP	IDA_macrophage_vs_M1
6,15E-08	0,33744252	0,576	0,352	0,002036	UBE2L6	UPP	IDA_macrophage_vs_M1
6,26E-08	0,33324603	0,34	0,165	0,002074	IKZF1	UPP	IDA_macrophage_vs_M1
6,27E-08	0,32764058	0,359	0,185	0,002076	BIN2	UPP	IDA_macrophage_vs_M1
6,47E-08	0,32030003	0,546	0,365	0,002142	SNRPF	UPP	IDA_macrophage_vs_M1
6,48E-08	0,33423818	0,448	0,261	0,002146	ATRAID	UPP	IDA_macrophage_vs_M1
6,56E-08	0,28735171	0,239	0,091	0,002174	KLF3	UPP	IDA_macrophage_vs_M1
6,57E-08	0,31289095	0,253	0,106	0,002176	PLPBP	UPP	IDA_macrophage_vs_M1
6,58E-08	0,27719358	0,538	0,327	0,00218	SNRPD1	UPP	IDA_macrophage_vs_M1
6,61E-08	0,35789297	0,522	0,334	0,00219	CPPED1	UPP	IDA_macrophage_vs_M1
6,73E-08	0,30170151	0,671	0,42	0,002229	VAMP5	UPP	IDA_macrophage_vs_M1
6,85E-08	0,34001615	0,378	0,203	0,002269	CCAR1	UPP	IDA_macrophage_vs_M1
6,89E-08	0,34672514	0,361	0,197	0,002281	SUCLG1	UPP	IDA_macrophage_vs_M1
7,07E-08	0,4815323	0,785	0,651	0,002342	INSIG1	UPP	IDA_macrophage_vs_M1
7,14E-08	0,37523301	0,546	0,347	0,002365	SLC8A1	UPP	IDA_macrophage_vs_M1
7,21E-08	0,30562614	0,296	0,134	0,00239	C15orf39	UPP	IDA_macrophage_vs_M1
7,25E-08	0,40414266	0,514	0,316	0,002401	LDLR	UPP	IDA_macrophage_vs_M1
7,27E-08	0,35640524	0,296	0,134	0,002408	SATB1	UPP	IDA_macrophage_vs_M1
7,35E-08	0,40854622	0,326	0,167	0,002434	DCXR	UPP	IDA_macrophage_vs_M1
8,00E-08	0,3569249	0,375	0,203	0,002648	SNRNP70	UPP	IDA_macrophage_vs_M1

8,12E-08	0,31654744	0,399	0,22	0,002689	NHP2	UPP	IDA_macrophage_vs_M1
8,38E-08	0,30453314	0,25	0,106	0,002776	NAPRT	UPP	IDA_macrophage_vs_M1
8,42E-08	0,32975983	0,296	0,139	0,00279	CRTAP	UPP	IDA_macrophage_vs_M1
8,45E-08	0,33652833	0,405	0,23	0,002799	SCAMP2	UPP	IDA_macrophage_vs_M1
8,47E-08	0,28104407	0,212	0,078	0,002807	SP140L	UPP	IDA_macrophage_vs_M1
8,73E-08	0,31263188	0,421	0,241	0,002893	APH1A	UPP	IDA_macrophage_vs_M1
8,88E-08	0,31271598	0,359	0,185	0,002942	GRK3	UPP	IDA_macrophage_vs_M1
9,46E-08	0,28011297	0,166	0,048	0,003134	ZBTB20	UPP	IDA_macrophage_vs_M1
9,77E-08	0,35955346	0,943	0,863	0,003238	PFN1	UPP	IDA_macrophage_vs_M1
9,80E-08	0,27488923	0,745	0,57	0,003246	NDUFS5	UPP	IDA_macrophage_vs_M1
9,92E-08	0,35756952	0,283	0,132	0,003286	CLTB	UPP	IDA_macrophage_vs_M1
1,00E-07	0,3667973	0,446	0,263	0,003318	CTDNEP1	UPP	IDA_macrophage_vs_M1
1,01E-07	0,27691999	0,196	0,068	0,003336	RTN1	UPP	IDA_macrophage_vs_M1
1,03E-07	0,48004509	0,554	0,365	0,003421	RANBP2	UPP	IDA_macrophage_vs_M1
1,04E-07	0,29714362	0,307	0,147	0,003452	FAM96A	UPP	IDA_macrophage_vs_M1
1,09E-07	0,32860105	0,389	0,215	0,003605	TMEM14B	UPP	IDA_macrophage_vs_M1
1,11E-07	0,29476484	0,682	0,499	0,003676	CHD1	UPP	IDA_macrophage_vs_M1
1,14E-07	0,40235481	0,378	0,21	0,003765	CCDC107	UPP	IDA_macrophage_vs_M1
1,15E-07	0,32937251	0,383	0,213	0,003809	TIMM10	UPP	IDA_macrophage_vs_M1
1,21E-07	0,33353734	0,867	0,742	0,004004	EEF2	UPP	IDA_macrophage_vs_M1
1,23E-07	0,32942008	0,582	0,382	0,004069	LSM7	UPP	IDA_macrophage_vs_M1
1,24E-07	0,278253	0,628	0,435	0,004108	PFDN2	UPP	IDA_macrophage_vs_M1
1,24E-07	0,28368547	0,293	0,137	0,004115	CMTM7	UPP	IDA_macrophage_vs_M1
1,30E-07	0,35481317	0,329	0,167	0,004305	ACAA1	UPP	IDA_macrophage_vs_M1
1,34E-07	0,28758852	0,386	0,213	0,004447	ANXA6	UPP	IDA_macrophage_vs_M1
1,38E-07	0,29833257	0,296	0,142	0,004565	RCC2	UPP	IDA_macrophage_vs_M1
1,38E-07	0,36152808	0,755	0,603	0,004567	ATP5F1B	UPP	IDA_macrophage_vs_M1
1,40E-07	0,48623389	0,614	0,418	0,004654	PRDM1	UPP	IDA_macrophage_vs_M1
1,46E-07	0,3142728	0,288	0,137	0,004851	HACD4	UPP	IDA_macrophage_vs_M1
1,49E-07	0,38199373	0,546	0,365	0,004939	PTEN	UPP	IDA_macrophage_vs_M1
1,51E-07	0,40450626	0,543	0,349	0,004996	MRC1	UPP	IDA_macrophage_vs_M1
1,51E-07	0,2687215	0,304	0,147	0,005011	IDH3B	UPP	IDA_macrophage_vs_M1
1,56E-07	0,32658331	0,37	0,195	0,005181	GRK2	UPP	IDA_macrophage_vs_M1
1,59E-07	0,33174492	0,421	0,246	0,005261	RNF114	UPP	IDA_macrophage_vs_M1
1,60E-07	0,36200423	0,427	0,241	0,0053	IFRD1	UPP	IDA_macrophage_vs_M1
1,61E-07	0,2965317	0,236	0,099	0,005334	APOBEC3C	UPP	IDA_macrophage_vs_M1
1,63E-07	0,29090369	0,299	0,139	0,005409	DHRS4L2	UPP	IDA_macrophage_vs_M1
1,68E-07	0,30730058	0,397	0,218	0,005577	DCAF7	UPP	IDA_macrophage_vs_M1
1,76E-07	0,38947475	0,435	0,266	0,005845	NFIL3	UPP	IDA_macrophage_vs_M1
1,82E-07	0,2666245	0,236	0,096	0,006016	WDR46	UPP	IDA_macrophage_vs_M1
1,82E-07	0,38031823	0,533	0,347	0,006024	DBNL	UPP	IDA_macrophage_vs_M1
1,83E-07	0,28379405	0,234	0,096	0,006049	TRIM14	UPP	IDA_macrophage_vs_M1
2,01E-07	0,31641925	0,826	0,671	0,006673	COX6C	UPP	IDA_macrophage_vs_M1
2,02E-07	0,2890002	0,408	0,233	0,006678	MPC1	UPP	IDA_macrophage_vs_M1
2,04E-07	0,29936447	0,391	0,23	0,006763	POLR2G	UPP	IDA_macrophage_vs_M1
2,09E-07	0,32173878	0,435	0,251	0,006937	TMEM256	UPP	IDA_macrophage_vs_M1
2,12E-07	0,27596409	0,228	0,094	0,007036	ISOC2	UPP	IDA_macrophage_vs_M1
2,14E-07	0,2932396	0,772	0,562	0,007087	PCBP2	UPP	IDA_macrophage_vs_M1
2,15E-07	0,27948592	0,601	0,38	0,007122	FPR3	UPP	IDA_macrophage_vs_M1
2,24E-07	0,32945848	0,443	0,266	0,00743	C9orf16	UPP	IDA_macrophage_vs_M1

2,25E-07	0,36858966	0,312	0,162	0,007449	SLC25A19	UPP	IDA_macrophage_vs_M1
2,33E-07	0,32979056	0,302	0,149	0,007726	UPF2	UPP	IDA_macrophage_vs_M1
2,34E-07	0,28901199	0,342	0,175	0,007758	ECH1	UPP	IDA_macrophage_vs_M1
2,37E-07	0,33663784	0,549	0,337	0,007837	ABRACL	UPP	IDA_macrophage_vs_M1
2,44E-07	0,36885756	0,348	0,192	0,008088	METTL26	UPP	IDA_macrophage_vs_M1
2,55E-07	0,29832255	0,323	0,159	0,008432	TMEM273	UPP	IDA_macrophage_vs_M1
2,59E-07	0,26652145	0,302	0,149	0,00857	PHF3	UPP	IDA_macrophage_vs_M1
2,67E-07	0,28970351	0,264	0,119	0,008855	MRPS12	UPP	IDA_macrophage_vs_M1
2,77E-07	0,28450177	0,981	0,937	0,009189	CFL1	UPP	IDA_macrophage_vs_M1
2,81E-07	0,372939	0,655	0,494	0,009311	EIF3H	UPP	IDA_macrophage_vs_M1
2,86E-07	0,37152455	0,603	0,425	0,009478	SSR1	UPP	IDA_macrophage_vs_M1
2,99E-07	0,33654464	0,421	0,241	0,00989	U2SURP	UPP	IDA_macrophage_vs_M1
3,07E-07	0,36029451	0,867	0,757	0,010169	CTSH	UPP	IDA_macrophage_vs_M1
3,15E-07	0,2912871	0,236	0,101	0,010434	C6orf48	UPP	IDA_macrophage_vs_M1
3,18E-07	0,29853889	0,386	0,22	0,010528	TBCB	UPP	IDA_macrophage_vs_M1
3,33E-07	0,26975837	0,495	0,314	0,01103	ANP32A	UPP	IDA_macrophage_vs_M1
3,34E-07	0,30237004	0,579	0,4	0,011076	HSPA9	UPP	IDA_macrophage_vs_M1
3,45E-07	0,33325112	0,446	0,268	0,011416	CCNH	UPP	IDA_macrophage_vs_M1
3,46E-07	0,31726398	0,571	0,385	0,011468	EIF4A2	UPP	IDA_macrophage_vs_M1
3,57E-07	0,27232563	0,315	0,157	0,011839	SIL1	UPP	IDA_macrophage_vs_M1
3,71E-07	0,31284595	0,459	0,281	0,012287	LSM8	UPP	IDA_macrophage_vs_M1
3,75E-07	0,26621811	0,296	0,139	0,012422	RNPEPL1	UPP	IDA_macrophage_vs_M1
3,76E-07	0,32978481	0,53	0,354	0,012444	CHMP4A	UPP	IDA_macrophage_vs_M1
3,78E-07	0,30210654	0,932	0,868	0,012505	EEF1D	UPP	IDA_macrophage_vs_M1
3,81E-07	0,32838589	0,5	0,319	0,012617	FOXN3	UPP	IDA_macrophage_vs_M1
3,94E-07	0,27741628	0,375	0,21	0,013047	PRMT1	UPP	IDA_macrophage_vs_M1
3,97E-07	0,28501838	0,372	0,208	0,013163	DRAM2	UPP	IDA_macrophage_vs_M1
4,03E-07	0,27179854	0,527	0,339	0,013354	FGD4	UPP	IDA_macrophage_vs_M1
4,06E-07	0,27731839	0,323	0,165	0,01344	TMPO	UPP	IDA_macrophage_vs_M1
4,15E-07	0,34369125	0,75	0,577	0,013746	SARAF	UPP	IDA_macrophage_vs_M1
4,20E-07	0,30559468	0,342	0,187	0,013909	ABHD2	UPP	IDA_macrophage_vs_M1
4,23E-07	0,28539202	0,283	0,137	0,014028	ARID4A	UPP	IDA_macrophage_vs_M1
4,33E-07	0,34123007	0,476	0,304	0,014354	EIF3D	UPP	IDA_macrophage_vs_M1
4,88E-07	0,60331346	0,552	0,382	0,016149	CD52	UPP	IDA_macrophage_vs_M1
5,07E-07	0,32895814	0,821	0,704	0,01679	COX5B	UPP	IDA_macrophage_vs_M1
5,35E-07	0,33539106	0,652	0,481	0,017739	WDR83OS	UPP	IDA_macrophage_vs_M1
5,74E-07	0,28987809	0,16	0,051	0,019021	MSR1	UPP	IDA_macrophage_vs_M1
5,76E-07	0,28902898	0,166	0,053	0,019089	EPB41	UPP	IDA_macrophage_vs_M1
5,80E-07	0,34617434	0,592	0,423	0,019216	OAZ2	UPP	IDA_macrophage_vs_M1
5,80E-07	0,32760632	0,546	0,377	0,019222	AURKAIP1	UPP	IDA_macrophage_vs_M1
6,33E-07	0,32516819	0,329	0,18	0,020972	MRPS15	UPP	IDA_macrophage_vs_M1
6,80E-07	0,295524	0,288	0,139	0,022536	PPIP5K2	UPP	IDA_macrophage_vs_M1
6,82E-07	0,33187595	0,361	0,197	0,022585	SECTM1	UPP	IDA_macrophage_vs_M1
7,02E-07	0,35555733	0,587	0,441	0,023255	EIF3G	UPP	IDA_macrophage_vs_M1
7,24E-07	0,30784598	0,329	0,175	0,023977	GAA	UPP	IDA_macrophage_vs_M1
7,34E-07	0,27442666	0,304	0,159	0,024305	PIN1	UPP	IDA_macrophage_vs_M1
7,50E-07	0,28615808	0,397	0,23	0,024852	TTYH3	UPP	IDA_macrophage_vs_M1
8,08E-07	0,29417282	0,943	0,83	0,026772	CD68	UPP	IDA_macrophage_vs_M1
8,36E-07	0,26825583	0,201	0,078	0,027709	SSBP4	UPP	IDA_macrophage_vs_M1
8,58E-07	0,31798999	0,747	0,565	0,02842	NEDD8	UPP	IDA_macrophage_vs_M1

8,68E-07	0,29229658	0,429	0,258	0,028737	XPO1	UPP	IDA_macrophage_vs_M1
8,79E-07	0,40466492	0,543	0,387	0,02913	WASF2	UPP	IDA_macrophage_vs_M1
9,21E-07	0,27495771	0,264	0,124	0,030496	C19orf24	UPP	IDA_macrophage_vs_M1
9,47E-07	0,31015383	0,383	0,22	0,031358	AES	UPP	IDA_macrophage_vs_M1
9,63E-07	0,32969014	0,408	0,243	0,0319	IQGAP2	UPP	IDA_macrophage_vs_M1
9,95E-07	0,26427291	0,266	0,127	0,032964	TOR3A	UPP	IDA_macrophage_vs_M1
1,01E-06	0,37466175	0,47	0,309	0,033353	MARCH1	UPP	IDA_macrophage_vs_M1
1,04E-06	0,30791586	0,247	0,114	0,034591	HPS3	UPP	IDA_macrophage_vs_M1
1,05E-06	0,27877671	0,179	0,066	0,034905	CD2AP	UPP	IDA_macrophage_vs_M1
1,12E-06	0,28631812	0,478	0,289	0,037149	JOSD1	UPP	IDA_macrophage_vs_M1
1,12E-06	0,3090126	0,332	0,18	0,037159	FBL	UPP	IDA_macrophage_vs_M1
1,15E-06	0,36731613	0,375	0,218	0,038105	PDCD4	UPP	IDA_macrophage_vs_M1
1,22E-06	0,29191524	0,264	0,129	0,04056	PQLC3	UPP	IDA_macrophage_vs_M1
1,25E-06	0,31392564	0,296	0,154	0,041356	ARHGAP45	UPP	IDA_macrophage_vs_M1
1,29E-06	0,32818602	0,413	0,251	0,042622	RNPS1	UPP	IDA_macrophage_vs_M1
1,31E-06	0,38364077	0,356	0,213	0,043306	MED29	UPP	IDA_macrophage_vs_M1
1,31E-06	0,28226772	0,413	0,248	0,043419	CREBL2	UPP	IDA_macrophage_vs_M1
1,33E-06	0,27756473	0,198	0,078	0,044103	TCOF1	UPP	IDA_macrophage_vs_M1
1,39E-06	0,29728402	0,462	0,296	0,045911	OCIAD1	UPP	IDA_macrophage_vs_M1
1,44E-06	0,3708203	0,495	0,327	0,047573	LRPAP1	UPP	IDA_macrophage_vs_M1
1,44E-06	0,30155799	0,37	0,208	0,047683	LPCAT2	UPP	IDA_macrophage_vs_M1
1,77E-65	-1,60761284	0,889	0,975	5,88E-61	CD44	DWW	IDA_macrophage_vs_M1
1,03E-62	-2,2134662	0,647	0,944	3,40E-58	C15orf48	DWW	IDA_macrophage_vs_M1
8,40E-61	-1,33191417	0,962	0,995	2,78E-56	VIM	DWW	IDA_macrophage_vs_M1
1,20E-60	-1,32340909	0,976	0,995	3,98E-56	SOD2	DWW	IDA_macrophage_vs_M1
7,79E-59	-2,58583401	0,508	0,927	2,58E-54	CCL3	DWW	IDA_macrophage_vs_M1
2,89E-55	-2,82961085	0,334	0,835	9,56E-51	CCL4	DWW	IDA_macrophage_vs_M1
3,94E-55	-1,7618128	0,823	0,944	1,31E-50	S100A10	DWW	IDA_macrophage_vs_M1
4,85E-51	-2,36833853	0,576	0,911	1,61E-46	IL1RN	DWW	IDA_macrophage_vs_M1
3,76E-48	-2,24708258	0,522	0,871	1,24E-43	CTSL	DWW	IDA_macrophage_vs_M1
7,05E-47	-1,67824885	0,853	0,952	2,34E-42	BCL2A1	DWW	IDA_macrophage_vs_M1
3,00E-46	-2,13818014	0,707	0,922	9,95E-42	IL1B	DWW	IDA_macrophage_vs_M1
5,90E-44	-1,24966529	0,918	0,97	1,96E-39	S100A6	DWW	IDA_macrophage_vs_M1
7,74E-41	-1,72651118	0,505	0,896	2,56E-36	S100A8	DWW	IDA_macrophage_vs_M1
1,35E-40	-1,57066159	0,288	0,737	4,48E-36	AQP9	DWW	IDA_macrophage_vs_M1
1,67E-40	-1,91455261	0,579	0,904	5,53E-36	CXCL8	DWW	IDA_macrophage_vs_M1
1,24E-39	-0,99485737	0,916	0,957	4,11E-35	ANXA5	DWW	IDA_macrophage_vs_M1
1,56E-39	-1,43768793	0,568	0,848	5,17E-35	TNFAIP8	DWW	IDA_macrophage_vs_M1
1,92E-38	-1,39552822	0,261	0,671	6,38E-34	PDE4DIP	DWW	IDA_macrophage_vs_M1
6,87E-37	-1,83422789	0,899	0,972	2,28E-32	TIMP1	DWW	IDA_macrophage_vs_M1
2,53E-34	-1,64479817	0,111	0,522	8,37E-30	TNFAIP6	DWW	IDA_macrophage_vs_M1
5,23E-34	-1,81501955	0,109	0,506	1,73E-29	TNIP3	DWW	IDA_macrophage_vs_M1
2,82E-32	-1,11132882	0,535	0,805	9,35E-28	KYNU	DWW	IDA_macrophage_vs_M1
3,39E-32	-2,61447047	0,364	0,722	1,12E-27	CCL3L1	DWW	IDA_macrophage_vs_M1
5,81E-32	-2,56489937	0,198	0,582	1,93E-27	INHBA	DWW	IDA_macrophage_vs_M1
6,44E-32	-1,4133263	0,087	0,453	2,13E-27	SDC2	DWW	IDA_macrophage_vs_M1
1,64E-31	-0,78787009	0,97	0,985	5,44E-27	FCER1G	DWW	IDA_macrophage_vs_M1
2,00E-30	-1,44541232	0,09	0,453	6,64E-26	EMP1	DWW	IDA_macrophage_vs_M1
1,12E-29	-1,14806354	0,867	0,934	3,70E-25	LGALS1	DWW	IDA_macrophage_vs_M1
2,09E-29	-1,34549078	0,685	0,952	6,93E-25	S100A9	DWW	IDA_macrophage_vs_M1

2,16E-29	-2,29275764	0,084	0,43	7,15E-25	IL1A	DWW	IDA_macrophage_vs_M1
2,47E-28	-0,99038728	0,375	0,757	8,19E-24	SLC11A1	DWW	IDA_macrophage_vs_M1
2,67E-28	-0,68901589	0,981	0,987	8,85E-24	S100A11	DWW	IDA_macrophage_vs_M1
1,52E-27	-1,70327525	0,062	0,382	5,03E-23	MIR3945H(	DWW	IDA_macrophage_vs_M1
2,66E-27	-1,0873192	0,212	0,57	8,80E-23	TRAF1	DWW	IDA_macrophage_vs_M1
2,84E-27	-0,97565734	0,97	0,98	9,42E-23	GAPDH	DWW	IDA_macrophage_vs_M1
3,63E-27	-0,86449937	0,804	0,924	1,20E-22	PGK1	DWW	IDA_macrophage_vs_M1
6,13E-27	-1,71854964	0,076	0,405	2,03E-22	SLC7A11	DWW	IDA_macrophage_vs_M1
6,51E-26	-1,70812568	0,875	0,878	2,16E-21	CSTB	DWW	IDA_macrophage_vs_M1
1,31E-25	-1,18347325	0,394	0,739	4,35E-21	VCAN	DWW	IDA_macrophage_vs_M1
2,76E-25	-0,99149615	0,38	0,681	9,14E-21	TNIP1	DWW	IDA_macrophage_vs_M1
3,93E-25	-1,09178916	0,443	0,759	1,30E-20	EHD1	DWW	IDA_macrophage_vs_M1
1,25E-24	-0,93526983	0,109	0,433	4,15E-20	CD274	DWW	IDA_macrophage_vs_M1
1,48E-24	-2,3720694	0,264	0,603	4,89E-20	CCL4L2	DWW	IDA_macrophage_vs_M1
1,73E-24	-1,12876875	0,644	0,851	5,74E-20	GK	DWW	IDA_macrophage_vs_M1
1,84E-24	-1,29830016	0,231	0,582	6,11E-20	S100A12	DWW	IDA_macrophage_vs_M1
4,15E-24	-0,88962962	0,736	0,833	1,37E-19	SEC61G	DWW	IDA_macrophage_vs_M1
5,31E-24	-0,81483739	0,003	0,251	1,76E-19	AK4	DWW	IDA_macrophage_vs_M1
6,09E-24	-3,95182768	0,019	0,281	2,02E-19	SPP1	DWW	IDA_macrophage_vs_M1
9,87E-24	-0,91018438	0,299	0,633	3,27E-19	ABL2	DWW	IDA_macrophage_vs_M1
2,47E-23	-1,56387514	0,16	0,468	8,17E-19	MIR155HG	DWW	IDA_macrophage_vs_M1
3,31E-23	-1,05225074	0,668	0,825	1,10E-18	WTAP	DWW	IDA_macrophage_vs_M1
3,49E-23	-0,93116963	0,693	0,868	1,16E-18	ATP13A3	DWW	IDA_macrophage_vs_M1
3,81E-23	-1,52382674	0,28	0,577	1,26E-18	ACSL1	DWW	IDA_macrophage_vs_M1
4,73E-23	-1,53215335	0,019	0,276	1,57E-18	ACOD1	DWW	IDA_macrophage_vs_M1
5,18E-23	-0,96085979	0,37	0,656	1,72E-18	CLEC4E	DWW	IDA_macrophage_vs_M1
1,34E-22	-0,93172189	0,125	0,425	4,44E-18	ARID5B	DWW	IDA_macrophage_vs_M1
1,66E-22	-1,12036583	0,179	0,509	5,51E-18	SLC39A8	DWW	IDA_macrophage_vs_M1
1,86E-22	-0,98304501	0,747	0,858	6,15E-18	FCGR2A	DWW	IDA_macrophage_vs_M1
1,86E-22	-1,14024473	0,139	0,443	6,17E-18	CLEC5A	DWW	IDA_macrophage_vs_M1
1,86E-22	-0,98624327	0,772	0,876	6,17E-18	NINJ1	DWW	IDA_macrophage_vs_M1
2,23E-22	-0,92673033	0,372	0,673	7,40E-18	TLR2	DWW	IDA_macrophage_vs_M1
6,12E-22	-0,66591305	0,891	0,957	2,03E-17	SDCBP	DWW	IDA_macrophage_vs_M1
1,26E-21	-0,91824173	0,079	0,357	4,17E-17	LUCAT1	DWW	IDA_macrophage_vs_M1
1,81E-21	-1,20537869	0,22	0,565	6,01E-17	TNF	DWW	IDA_macrophage_vs_M1
2,76E-21	-1,00266355	0,122	0,428	9,13E-17	FLT1	DWW	IDA_macrophage_vs_M1
3,68E-21	-0,90139411	0,163	0,491	1,22E-16	SLC16A10	DWW	IDA_macrophage_vs_M1
7,71E-21	-3,00348347	0,057	0,316	2,56E-16	CXCL1	DWW	IDA_macrophage_vs_M1
1,42E-20	-1,29539107	1	1	4,71E-16	FTH1	DWW	IDA_macrophage_vs_M1
2,88E-20	-3,86621489	0,014	0,235	9,54E-16	CXCL5	DWW	IDA_macrophage_vs_M1
4,82E-20	-0,8536658	0,193	0,489	1,60E-15	ANKRD28	DWW	IDA_macrophage_vs_M1
6,56E-20	-1,08859948	0,065	0,316	2,17E-15	ADAM19	DWW	IDA_macrophage_vs_M1
1,36E-19	-0,79144442	0,122	0,395	4,51E-15	CD59	DWW	IDA_macrophage_vs_M1
1,85E-19	-0,56274798	0,005	0,213	6,11E-15	MSC	DWW	IDA_macrophage_vs_M1
2,38E-19	-2,25864136	0,359	0,608	7,88E-15	CXCL3	DWW	IDA_macrophage_vs_M1
3,21E-19	-1,27261126	0,158	0,448	1,06E-14	APOBEC3A	DWW	IDA_macrophage_vs_M1
4,60E-19	-1,58524957	0,204	0,501	1,52E-14	IL7R	DWW	IDA_macrophage_vs_M1
8,03E-19	-0,89298423	0,209	0,486	2,66E-14	BCAT1	DWW	IDA_macrophage_vs_M1
8,78E-19	-0,68573696	0,853	0,916	2,91E-14	ANXA2	DWW	IDA_macrophage_vs_M1
1,65E-18	-0,94356764	0,742	0,861	5,47E-14	MARCKS	DWW	IDA_macrophage_vs_M1

2,17E-18	-0,85529655	0,312	0,595	7,19E-14	ANPEP	DWW	IDA_macrophage_vs_M1
3,23E-18	-0,78888617	0,995	0,99	1,07E-13	MALAT1	DWW	IDA_macrophage_vs_M1
3,17E-17	-2,34308494	0,571	0,714	1,05E-12	MT2A	DWW	IDA_macrophage_vs_M1
4,20E-17	-1,01286271	0,44	0,701	1,39E-12	CD300E	DWW	IDA_macrophage_vs_M1
5,30E-17	-0,79483797	0,649	0,83	1,76E-12	ETS2	DWW	IDA_macrophage_vs_M1
1,07E-16	-0,71948645	0,935	0,962	3,56E-12	CD63	DWW	IDA_macrophage_vs_M1
1,08E-16	-0,72826871	0,804	0,863	3,59E-12	C4orf3	DWW	IDA_macrophage_vs_M1
1,22E-16	-0,75421451	0,397	0,648	4,05E-12	TREM1	DWW	IDA_macrophage_vs_M1
1,59E-16	-0,89516969	0,356	0,605	5,28E-12	RNF19B	DWW	IDA_macrophage_vs_M1
3,20E-16	-0,77598612	0,655	0,8	1,06E-11	BZW1	DWW	IDA_macrophage_vs_M1
3,43E-16	-0,73884246	0,78	0,919	1,14E-11	IER3	DWW	IDA_macrophage_vs_M1
5,08E-16	-0,74997891	0,236	0,486	1,68E-11	IRAK2	DWW	IDA_macrophage_vs_M1
7,11E-16	-1,31729169	0,372	0,562	2,35E-11	ISG20	DWW	IDA_macrophage_vs_M1
1,12E-15	-1,4618152	0,046	0,248	3,73E-11	F3	DWW	IDA_macrophage_vs_M1
1,16E-15	-0,90313577	0,543	0,727	3,83E-11	UPP1	DWW	IDA_macrophage_vs_M1
2,14E-15	-0,78508768	0,389	0,585	7,10E-11	CD99	DWW	IDA_macrophage_vs_M1
3,81E-15	-1,02716305	0,236	0,458	1,26E-10	MMP14	DWW	IDA_macrophage_vs_M1
4,52E-15	-0,57269152	0,022	0,2	1,50E-10	MSANTD3	DWW	IDA_macrophage_vs_M1
4,86E-15	-0,80563897	0,296	0,532	1,61E-10	PNP	DWW	IDA_macrophage_vs_M1
5,07E-15	-1,24329745	0,383	0,587	1,68E-10	SLC25A37	DWW	IDA_macrophage_vs_M1
5,16E-15	-1,04944226	0,027	0,213	1,71E-10	TNFSF14	DWW	IDA_macrophage_vs_M1
5,32E-15	-1,03732145	0,245	0,481	1,76E-10	CDC42EP3	DWW	IDA_macrophage_vs_M1
6,14E-15	-0,60871969	0,755	0,899	2,03E-10	EMP3	DWW	IDA_macrophage_vs_M1
6,79E-15	-0,62318898	0,141	0,38	2,25E-10	SLC11A2	DWW	IDA_macrophage_vs_M1
8,76E-15	-0,94138922	0,443	0,635	2,90E-10	FNDC3B	DWW	IDA_macrophage_vs_M1
9,82E-15	-0,58708303	0,03	0,215	3,25E-10	ITGB8	DWW	IDA_macrophage_vs_M1
1,13E-14	-0,58423335	0,791	0,843	3,74E-10	PKM	DWW	IDA_macrophage_vs_M1
1,21E-14	-1,74204536	0,06	0,261	4,02E-10	IL6	DWW	IDA_macrophage_vs_M1
1,28E-14	-1,57270098	0,598	0,727	4,24E-10	PTGS2	DWW	IDA_macrophage_vs_M1
1,35E-14	-0,84235895	0,033	0,215	4,48E-10	SLAMF9	DWW	IDA_macrophage_vs_M1
1,51E-14	-0,55067975	0,022	0,197	5,01E-10	MIR3142H(	DWW	IDA_macrophage_vs_M1
3,95E-14	-1,42428342	0	0,144	1,31E-09	SERPINB2	DWW	IDA_macrophage_vs_M1
5,11E-14	-0,86497233	0,226	0,451	1,69E-09	C1orf122	DWW	IDA_macrophage_vs_M1
6,01E-14	-0,90321079	0,359	0,557	1,99E-09	RAB13	DWW	IDA_macrophage_vs_M1
6,26E-14	-1,57943239	0,527	0,691	2,07E-09	CXCL2	DWW	IDA_macrophage_vs_M1
6,37E-14	-0,7962963	0,079	0,281	2,11E-09	BNIP3	DWW	IDA_macrophage_vs_M1
8,53E-14	-1,908193	0,03	0,2	2,82E-09	RNASE1	DWW	IDA_macrophage_vs_M1
1,33E-13	-0,82071256	0,25	0,473	4,41E-09	IL3RA	DWW	IDA_macrophage_vs_M1
1,95E-13	-0,65666993	0,397	0,61	6,46E-09	ACTN1	DWW	IDA_macrophage_vs_M1
2,24E-13	-0,64546949	0,016	0,172	7,42E-09	SLCO4A1	DWW	IDA_macrophage_vs_M1
2,86E-13	-0,78646256	0,101	0,309	9,46E-09	MARCO	DWW	IDA_macrophage_vs_M1
3,54E-13	-0,53242804	0,098	0,304	1,17E-08	KCNN4	DWW	IDA_macrophage_vs_M1
4,35E-13	-0,83544702	0,212	0,428	1,44E-08	MAP3K20	DWW	IDA_macrophage_vs_M1
6,09E-13	-0,8643446	0,84	0,878	2,02E-08	PLEK	DWW	IDA_macrophage_vs_M1
7,31E-13	-0,35540936	0,011	0,157	2,42E-08	HTRA1	DWW	IDA_macrophage_vs_M1
1,29E-12	-0,73612635	0,429	0,625	4,27E-08	NUMB	DWW	IDA_macrophage_vs_M1
1,61E-12	-0,74968573	0,734	0,823	5,32E-08	LDHA	DWW	IDA_macrophage_vs_M1
1,82E-12	-0,62745674	0,179	0,408	6,03E-08	SLC2A6	DWW	IDA_macrophage_vs_M1
3,55E-12	-0,74360183	0,329	0,532	1,18E-07	NBN	DWW	IDA_macrophage_vs_M1
3,60E-12	-0,37129596	0,043	0,213	1,19E-07	SLC41A2	DWW	IDA_macrophage_vs_M1

3,66E-12	-0,65619411	0,182	0,385	1,21E-07	GRAMD1A	DWW	IDA_macrophage_vs_M1
4,66E-12	-0,61030367	0,111	0,309	1,54E-07	MAP4K4	DWW	IDA_macrophage_vs_M1
4,85E-12	-0,4338392	0,815	0,889	1,61E-07	ACTR3	DWW	IDA_macrophage_vs_M1
4,92E-12	-0,49704125	0,796	0,916	1,63E-07	SERPINA1	DWW	IDA_macrophage_vs_M1
5,00E-12	-1,55582375	0,323	0,473	1,66E-07	MIF	DWW	IDA_macrophage_vs_M1
5,11E-12	-1,97609134	0,147	0,354	1,69E-07	CCL20	DWW	IDA_macrophage_vs_M1
5,21E-12	-0,61586981	0,264	0,489	1,72E-07	ITGA5	DWW	IDA_macrophage_vs_M1
5,85E-12	-0,76308141	0,935	0,975	1,94E-07	PLAUR	DWW	IDA_macrophage_vs_M1
6,54E-12	-0,56102959	0,071	0,248	2,17E-07	OPTN	DWW	IDA_macrophage_vs_M1
6,67E-12	-0,63215132	0,155	0,352	2,21E-07	ALCAM	DWW	IDA_macrophage_vs_M1
7,35E-12	-0,61290632	0,867	0,919	2,44E-07	NFKBIA	DWW	IDA_macrophage_vs_M1
7,94E-12	-0,56971333	0,31	0,539	2,63E-07	SMIM25	DWW	IDA_macrophage_vs_M1
8,07E-12	-0,55142623	0,802	0,863	2,67E-07	CD53	DWW	IDA_macrophage_vs_M1
8,28E-12	-0,91056465	0,5	0,722	2,74E-07	G0S2	DWW	IDA_macrophage_vs_M1
8,84E-12	-0,75332548	0,821	0,83	2,93E-07	SERPINB9	DWW	IDA_macrophage_vs_M1
9,00E-12	-0,54033636	0,867	0,891	2,98E-07	GSTO1	DWW	IDA_macrophage_vs_M1
1,12E-11	-0,69776371	0,283	0,491	3,71E-07	DRAM1	DWW	IDA_macrophage_vs_M1
1,12E-11	-0,40573843	0,027	0,175	3,72E-07	FSD1L	DWW	IDA_macrophage_vs_M1
1,13E-11	-1,17319656	0,038	0,195	3,73E-07	CCL5	DWW	IDA_macrophage_vs_M1
1,53E-11	-0,58353581	0,818	0,866	5,05E-07	CFLAR	DWW	IDA_macrophage_vs_M1
2,07E-11	-0,78574449	0,812	0,868	6,85E-07	SERPINB1	DWW	IDA_macrophage_vs_M1
2,30E-11	-0,80429485	0,171	0,354	7,62E-07	DDX60L	DWW	IDA_macrophage_vs_M1
4,01E-11	-0,48761701	0,136	0,327	1,33E-06	FAM129B	DWW	IDA_macrophage_vs_M1
4,79E-11	-0,57584181	0,207	0,405	1,59E-06	DOCK4	DWW	IDA_macrophage_vs_M1
5,72E-11	-1,23305135	0,114	0,294	1,89E-06	IL1R2	DWW	IDA_macrophage_vs_M1
6,12E-11	-0,36932253	0,014	0,142	2,03E-06	ABHD17C	DWW	IDA_macrophage_vs_M1
6,13E-11	-1,05283076	0,848	0,848	2,03E-06	TXN	DWW	IDA_macrophage_vs_M1
6,29E-11	-0,27833958	0,011	0,137	2,08E-06	TNS1	DWW	IDA_macrophage_vs_M1
7,30E-11	-0,73513227	0,508	0,651	2,42E-06	ARFGAP3	DWW	IDA_macrophage_vs_M1
7,64E-11	-0,57108955	0,177	0,37	2,53E-06	DENND5A	DWW	IDA_macrophage_vs_M1
1,09E-10	-0,50142956	0,766	0,82	3,63E-06	RAP1B	DWW	IDA_macrophage_vs_M1
1,11E-10	-0,78792097	0,446	0,628	3,68E-06	CCRL2	DWW	IDA_macrophage_vs_M1
1,14E-10	-0,75174859	0,103	0,286	3,79E-06	ALOX5AP	DWW	IDA_macrophage_vs_M1
1,16E-10	-0,48629684	0,076	0,246	3,85E-06	ECE1	DWW	IDA_macrophage_vs_M1
1,50E-10	-0,67205925	0,538	0,671	4,97E-06	FLNA	DWW	IDA_macrophage_vs_M1
2,04E-10	-0,586056	0,073	0,235	6,76E-06	OASL	DWW	IDA_macrophage_vs_M1
2,05E-10	-0,5607277	0,332	0,542	6,81E-06	MYO1G	DWW	IDA_macrophage_vs_M1
2,07E-10	-0,61280874	0,285	0,476	6,87E-06	SLC44A1	DWW	IDA_macrophage_vs_M1
2,21E-10	-0,67131437	0	0,104	7,34E-06	CEMIP	DWW	IDA_macrophage_vs_M1
2,76E-10	-0,46727798	0,215	0,423	9,15E-06	ACSL5	DWW	IDA_macrophage_vs_M1
3,01E-10	-0,65164745	0,226	0,42	9,96E-06	GPR84	DWW	IDA_macrophage_vs_M1
3,72E-10	-0,69925516	0,312	0,489	1,23E-05	RAPGEF2	DWW	IDA_macrophage_vs_M1
3,95E-10	-0,58095587	0,043	0,187	1,31E-05	SLAMF1	DWW	IDA_macrophage_vs_M1
3,97E-10	-1,06311246	0,035	0,172	1,32E-05	MT1E	DWW	IDA_macrophage_vs_M1
5,09E-10	-0,93837217	0,073	0,23	1,69E-05	MT1F	DWW	IDA_macrophage_vs_M1
5,41E-10	-0,63805831	0,679	0,795	1,79E-05	NFKB1	DWW	IDA_macrophage_vs_M1
5,83E-10	-0,41184694	0,046	0,19	1,93E-05	MGST1	DWW	IDA_macrophage_vs_M1
5,88E-10	-0,5261822	0,497	0,641	1,95E-05	ELOC	DWW	IDA_macrophage_vs_M1
6,63E-10	-0,53836145	0,307	0,489	2,19E-05	SIPA1L1	DWW	IDA_macrophage_vs_M1
7,05E-10	-0,4879305	0,014	0,129	2,34E-05	LINC02345	DWW	IDA_macrophage_vs_M1

7,67E-10	-0,93573061	0,16	0,339	2,54E-05	SLC5A3	DWW	IDA_macrophage_vs_M1
8,03E-10	-0,68583911	0,571	0,742	2,66E-05	IVNS1ABP	DWW	IDA_macrophage_vs_M1
8,06E-10	-1,0821804	0,402	0,539	2,67E-05	GBP5	DWW	IDA_macrophage_vs_M1
1,10E-09	-0,58096545	0,736	0,787	3,65E-05	CARD16	DWW	IDA_macrophage_vs_M1
1,13E-09	-0,33223553	0,054	0,203	3,74E-05	LRP12	DWW	IDA_macrophage_vs_M1
1,40E-09	-0,55116987	0,234	0,415	4,65E-05	MTF1	DWW	IDA_macrophage_vs_M1
1,84E-09	-0,45713345	0,239	0,433	6,11E-05	SPHK1	DWW	IDA_macrophage_vs_M1
1,91E-09	-0,43703024	0,856	0,886	6,31E-05	ENO1	DWW	IDA_macrophage_vs_M1
2,15E-09	-0,81532556	0,59	0,686	7,13E-05	TNFAIP2	DWW	IDA_macrophage_vs_M1
2,34E-09	-0,37218598	0,995	0,995	7,77E-05	SRGN	DWW	IDA_macrophage_vs_M1
3,20E-09	-0,49338155	0,541	0,704	0,000106	ABCA1	DWW	IDA_macrophage_vs_M1
3,23E-09	-0,75291666	0,302	0,471	0,000107	AC016831.:	DWW	IDA_macrophage_vs_M1
3,43E-09	-0,54483252	0,807	0,868	0,000114	PPP1R15A	DWW	IDA_macrophage_vs_M1
4,12E-09	-0,53345978	0,707	0,772	0,000136	NOP10	DWW	IDA_macrophage_vs_M1
5,01E-09	-0,7212576	0,212	0,37	0,000166	P4HA1	DWW	IDA_macrophage_vs_M1
6,09E-09	-0,40088147	0,88	0,906	0,000202	CLIC1	DWW	IDA_macrophage_vs_M1
6,47E-09	-2,40043781	0,258	0,4	0,000214	MMP9	DWW	IDA_macrophage_vs_M1
6,62E-09	-0,49172793	0,981	0,977	0,000219	NAMPT	DWW	IDA_macrophage_vs_M1
6,62E-09	-0,93864123	0,924	0,937	0,000219	MTRNR2L1	DWW	IDA_macrophage_vs_M1
6,87E-09	-0,59815255	0,883	0,929	0,000227	GLUL	DWW	IDA_macrophage_vs_M1
7,47E-09	-0,39091495	0,06	0,197	0,000248	LAMB3	DWW	IDA_macrophage_vs_M1
7,72E-09	-0,45807629	0,087	0,241	0,000256	AC005224.:	DWW	IDA_macrophage_vs_M1
8,32E-09	-1,92683408	0,293	0,438	0,000276	IDO1	DWW	IDA_macrophage_vs_M1
9,20E-09	-0,73430899	0,41	0,542	0,000305	TAP1	DWW	IDA_macrophage_vs_M1
9,27E-09	-0,34929061	0,978	0,962	0,000307	MYL6	DWW	IDA_macrophage_vs_M1
9,39E-09	-0,56043807	0,639	0,739	0,000311	ZFYVE16	DWW	IDA_macrophage_vs_M1
9,43E-09	-0,55452841	0,386	0,582	0,000312	MMP19	DWW	IDA_macrophage_vs_M1
1,03E-08	-0,48534524	0,13	0,284	0,000343	ALDOA	DWW	IDA_macrophage_vs_M1
1,28E-08	-0,69034034	0,508	0,63	0,000425	FPR1	DWW	IDA_macrophage_vs_M1
1,33E-08	-0,45817112	0,125	0,289	0,00044	NRIP3	DWW	IDA_macrophage_vs_M1
1,39E-08	-0,48077469	0,22	0,392	0,00046	BTG3	DWW	IDA_macrophage_vs_M1
1,41E-08	-2,18916709	0,016	0,122	0,000467	MT1H	DWW	IDA_macrophage_vs_M1
1,55E-08	-0,28215957	0,024	0,134	0,000514	LINC01588	DWW	IDA_macrophage_vs_M1
1,68E-08	-0,48029754	0,228	0,413	0,000555	ADAM8	DWW	IDA_macrophage_vs_M1
1,69E-08	-0,38805753	0,188	0,37	0,000561	SDC4	DWW	IDA_macrophage_vs_M1
1,73E-08	-0,72381901	0,72	0,823	0,000575	PPIF	DWW	IDA_macrophage_vs_M1
1,75E-08	-0,4828768	0,027	0,142	0,00058	PDPN	DWW	IDA_macrophage_vs_M1
1,84E-08	-0,52748349	0,283	0,458	0,000608	SNX9	DWW	IDA_macrophage_vs_M1
2,08E-08	-1,40458654	0,027	0,139	0,00069	CCL2	DWW	IDA_macrophage_vs_M1
2,22E-08	-0,4152778	0,109	0,263	0,000734	KLHL21	DWW	IDA_macrophage_vs_M1
2,22E-08	-0,50294991	0,117	0,271	0,000736	ASPH	DWW	IDA_macrophage_vs_M1
2,96E-08	-0,60307613	0,188	0,352	0,00098	P2RX7	DWW	IDA_macrophage_vs_M1
3,24E-08	-0,43796539	0,207	0,377	0,001072	PLP2	DWW	IDA_macrophage_vs_M1
4,26E-08	-0,40376493	0,989	0,985	0,001411	H3F3A	DWW	IDA_macrophage_vs_M1
4,56E-08	-0,53558932	0,16	0,316	0,001512	LIMK2	DWW	IDA_macrophage_vs_M1
4,63E-08	-0,37685072	0,111	0,263	0,001534	PDLIM7	DWW	IDA_macrophage_vs_M1
4,96E-08	-0,46254686	0,144	0,301	0,001644	USP12	DWW	IDA_macrophage_vs_M1
5,77E-08	-0,55310718	0,633	0,739	0,00191	DSE	DWW	IDA_macrophage_vs_M1
6,44E-08	-0,55937567	0,152	0,304	0,002133	AC005280.:	DWW	IDA_macrophage_vs_M1
6,49E-08	-0,76771301	0,573	0,648	0,002149	GBP2	DWW	IDA_macrophage_vs_M1

7,06E-08	-0,51427181	0,579	0,686	0,002339	ITGB1	DWW	IDA_macrophage_vs_M1
9,09E-08	-0,44783608	0,688	0,456	0,003013	HSPD1	DWW	IDA_macrophage_vs_M1
9,23E-08	-0,51701307	0,073	0,205	0,003058	CA2	DWW	IDA_macrophage_vs_M1
1,02E-07	-0,54363542	0,516	0,641	0,003388	AHNAK	DWW	IDA_macrophage_vs_M1
1,03E-07	-0,58563031	0,467	0,582	0,003422	SLC3A2	DWW	IDA_macrophage_vs_M1
1,15E-07	-0,47753167	0,84	0,871	0,00382	HIF1A	DWW	IDA_macrophage_vs_M1
1,19E-07	-0,44370504	0,09	0,225	0,00393	CLEC4D	DWW	IDA_macrophage_vs_M1
1,20E-07	-1,47139593	0,188	0,334	0,003983	MT1X	DWW	IDA_macrophage_vs_M1
1,24E-07	-0,51492765	0,245	0,403	0,004102	ANKLE2	DWW	IDA_macrophage_vs_M1
1,32E-07	-0,32432516	0,826	0,868	0,004374	LGALS3	DWW	IDA_macrophage_vs_M1
1,38E-07	-0,42076784	0,546	0,668	0,004562	RAB1A	DWW	IDA_macrophage_vs_M1
1,38E-07	-0,42159402	0,497	0,635	0,00457	TANK	DWW	IDA_macrophage_vs_M1
1,38E-07	-0,3087726	0,033	0,139	0,004578	ZNF697	DWW	IDA_macrophage_vs_M1
1,41E-07	-0,41172737	0,133	0,281	0,004671	NBPF19	DWW	IDA_macrophage_vs_M1
1,42E-07	-0,35090077	0,062	0,185	0,004713	TRIP10	DWW	IDA_macrophage_vs_M1
1,44E-07	-0,48113126	0,103	0,246	0,004759	AC015912.:	DWW	IDA_macrophage_vs_M1
1,53E-07	-0,5578759	0,304	0,451	0,005054	P2RX4	DWW	IDA_macrophage_vs_M1
1,62E-07	-0,47852281	0,894	0,899	0,005381	S100A4	DWW	IDA_macrophage_vs_M1
1,77E-07	-0,66046839	0,611	0,699	0,00587	DUSP6	DWW	IDA_macrophage_vs_M1
1,82E-07	-0,47696101	0,557	0,661	0,006042	GLIPR2	DWW	IDA_macrophage_vs_M1
1,84E-07	-0,6524148	0,81	0,858	0,0061	MTRNR2L8	DWW	IDA_macrophage_vs_M1
1,89E-07	-0,69244979	0,476	0,6	0,006271	EIF1B	DWW	IDA_macrophage_vs_M1
2,06E-07	-0,43808958	0,864	0,856	0,006837	SEC61B	DWW	IDA_macrophage_vs_M1
2,23E-07	-0,45834672	0,416	0,544	0,007375	SH3GLB1	DWW	IDA_macrophage_vs_M1
2,35E-07	-0,43324402	0,94	0,909	0,007794	PNRC1	DWW	IDA_macrophage_vs_M1
2,78E-07	-0,86037901	0,022	0,116	0,009214	MT1M	DWW	IDA_macrophage_vs_M1
3,08E-07	-0,5776389	0,438	0,542	0,010198	DNAJC3	DWW	IDA_macrophage_vs_M1
3,41E-07	-0,39453093	0,049	0,159	0,011279	AP003354.:	DWW	IDA_macrophage_vs_M1
3,41E-07	-2,2100203	0,052	0,162	0,011312	MT1G	DWW	IDA_macrophage_vs_M1
3,90E-07	-0,35753468	0,171	0,314	0,012918	CLIC4	DWW	IDA_macrophage_vs_M1
4,04E-07	-0,42502317	0,204	0,357	0,013397	KMO	DWW	IDA_macrophage_vs_M1
4,20E-07	-0,45130953	0,522	0,625	0,013917	SLC16A3	DWW	IDA_macrophage_vs_M1
4,56E-07	-0,56670499	0,128	0,268	0,015112	KCNJ2	DWW	IDA_macrophage_vs_M1
5,84E-07	-0,64076057	0,348	0,473	0,019347	GCH1	DWW	IDA_macrophage_vs_M1
5,86E-07	-0,4413515	0,462	0,595	0,019412	PTPN12	DWW	IDA_macrophage_vs_M1
5,87E-07	-0,62462115	0,603	0,742	0,019432	EREG	DWW	IDA_macrophage_vs_M1
6,32E-07	-0,4321602	0,06	0,175	0,020928	MIR222HG	DWW	IDA_macrophage_vs_M1
6,63E-07	-0,44863146	0,348	0,499	0,021949	SLC43A3	DWW	IDA_macrophage_vs_M1
6,96E-07	-0,46760039	0,024	0,116	0,023044	RRAD	DWW	IDA_macrophage_vs_M1
7,83E-07	-0,4629437	0,443	0,575	0,02595	RBMS1	DWW	IDA_macrophage_vs_M1
8,37E-07	-0,41635185	0,068	0,182	0,02772	CD82	DWW	IDA_macrophage_vs_M1
8,64E-07	-0,48722407	0,84	0,878	0,028628	TNFRSF1B	DWW	IDA_macrophage_vs_M1
8,99E-07	-0,31991583	0,179	0,329	0,029787	N4BP1	DWW	IDA_macrophage_vs_M1
9,50E-07	-0,54753696	0,215	0,349	0,031482	CPM	DWW	IDA_macrophage_vs_M1
9,52E-07	-0,40828324	0,894	0,871	0,031547	POMP	DWW	IDA_macrophage_vs_M1
9,68E-07	-0,35395971	0,734	0,843	0,032073	ICAM1	DWW	IDA_macrophage_vs_M1
1,06E-06	-0,31615873	0,071	0,187	0,03509	LDLRAD3	DWW	IDA_macrophage_vs_M1
1,18E-06	-0,4245307	0,188	0,329	0,038973	FPR2	DWW	IDA_macrophage_vs_M1
1,23E-06	-0,4442284	0,34	0,481	0,04061	MIR22HG	DWW	IDA_macrophage_vs_M1
1,37E-06	-0,45370986	0,16	0,299	0,045535	TGM2	DWW	IDA_macrophage_vs_M1

1,51E-06	-0,39481105	0,16	0,296	0,049889	PSMA6	DWW	IDA_macrophage_vs_M1

Supplementary Table 3. Distances of myeloid cells to intestinal lumen in CosMx SMI analysis of Figure 2e

health	SingleR2	n	min	mean	median	max	sd
CD	N2	1	310,37	310,37	310,37	310,37	NA
CD	N1	3	323,41	1188,9	795,28	2448,1	1115,685
HC	N2	6	561,96	1519,2	1190	2939,5	1029,753
CD	N3	6	213,88	1304,9	1365,8	2481	738,1388
HC	Eosinophils	9	368,32	1645,5	1688,8	3213,4	919,4201
HC	N3	9	548,83	1992,9	1980,6	3308,8	915,3936
HC	Inflammatory monocytes	10	332,55	1747,7	1968,1	3289,4	1130,305
HC	N1	10	395,17	2114,9	2390,1	3313,2	1086,044
HC	M1	11	203,08	1324,8	1244,1	3212,2	1117,851
CD	M1	159	75,459	736,62	533,79	2894,1	607,9152
CD	DCs	80	348,41	1592,4	1310,5	4740,2	976,4851
CD	IDA macrophages	591	106,85	2301,8	2176,9	5601,8	1264,295
CD	Cycling myeloid	15	932,49	2415,3	2186,3	4803,4	1256,462
CD	Mast	39	658,37	2722,2	2800,3	5307,2	1163,089
CD	M2	440	434,78	3005,5	3028,7	5651,1	1264,894
CD	Inflammatory monocytes	62	317,76	2951,9	3089	4789,3	1044,455
CD	M0	39	529,44	3337,7	3479,3	5252,4	1166,793
HC	IDA macrophages	31	200,47	1423,7	677,97	4517,7	1307,693
HC	M2	392	160,56	1441,6	1098,6	4576,1	1095,742
HC	M0	153	148,71	1428,2	1099,4	4540,6	1138,721
HC	Cycling myeloid	64	171,98	1539,5	1519,3	4134,3	1018,461
HC	Mast	109	287,5	1799,8	1853,8	3559,8	870,5829
HC	DCs	46	359,51	2698,7	3056,3	4611,5	1195,639
UC	M1	92	175,34	1457,1	1333,5	5003,9	964,6334
UC	N1	57	68,405	1776,8	1507	4935,1	1318,259
UC	Mast	71	328,12	1862,1	1687,5	4904,5	1151,343
UC	N3	59	61,182	1970,6	1737	4927,3	1374,81
UC	Inflammatory monocytes	133	167,36	1975,2	1747,2	4876,4	1183,358
UC	IDA macrophages	307	43,339	2170,9	1851,8	5329,6	1174,219
UC	M0	132	138,17	2143,4	1956	5373,1	1303,63
UC	Eosinophils	17	70,161	1931,1	2012,5	3599,8	1334,988
UC	N2	50	120,09	2080	2083,7	5335,2	1366,913
UC	Cycling myeloid	88	319,66	2399,7	2232	4961	1214,281
UC	M2	489	88,747	2444,1	2246,7	5293,1	1256,944
UC	DCs	53	343,68	2254,3	2340,5	4155,2	1009,63

Supplementary Table 4. Gene markers used to separate each of the five mail cell types identified by scRNA-seq.

**Cell type**	**Markers**
Epithelial cells	*EPCAM, AQP8, BEST4, MUC2, OLFM4, PLCG2, TRPM5, ZG16*
B and Plasma cells	*CD79A, BANK1, CD19, DERL3, MS4A1, MZB1*
T cells	*CD3D, CD3E, CD3G, CD8A, FOXP3, GZMA, GZMB, IL17A, NKG7, TRBC1*
Stromal cells	*ACTA2, ADAMDEC1, CHI3L1, COL3A1, NRXN1, PVALP, SOX6, VWF*
Myeloid cells	*AIF1, C1QA, C1QB, CD14, CMTM2, FCGR3B, LYZ, MS4A2, TPSAB1, TPSAB2*

Supplementary Table 5. Gene used for annotation of clusters within each of the five main cell types.

Epithelial cell subset (EPCAM+)	Colonocytes	*AQP8, FABP1 and SLC26A2*
	Inflammatory colonocytes	*DUOXA2, DUOX2 and LCN2 (low or none AQP8)*
	PLCG colonocytes	*PLCG2, HES1 and ELF1*
	Laminin colonocytes	*LAMA3 and EPHA2*
	BEST4 colonocytes	*BEST4 and OTOP2*
	Tuft cells	*TRPM5, SH2D6 and POU2F3*
	Goblet cells	*MUC2, TFF3 and SPINK4*
	Mature Goblet cells	*TFF1 and FER1L6*
	Secretory progenitors	*RETNLB, CLCA4 and HEPACAM2*
	Paneth-like cells	*DEFA5, DEFA6 and REG3A*
	Enteroendocrine cells	*CHGA, CHGB and NEUROD1*
	Ribosomal high (Ribhi) Epithelial cells	*RPS19, RPS18, RPL35*
	Cycling transit-Amplifying cells	*MKI67, TOP2A and PCNA*
Stromal cell subset (based on Kinchen et al)	S1 fibroblasts	*ADAMDEC1, CP and FABP4*
	S3 fibroblasts	*OGN, CCDC80 and GREM1*
	Peri-cryptal S2	*SOX6, F3*
	S2a	*VSTM2*
	S2b	*NPY, NRG1*
	Ribhi fibroblasts	*RPL7A, RPL28…*
	MT fibroblasts	*high expression of mitochondrial genes*

	Immediate early-response (IER) fibroblasts	*FOS, FOSB, IRF1*
	Fibroblastic reticular cells (FRCs)	*CCL19 and CCL21*
	Myofibroblasts	*SOSTDC1, ACTG2 and MYH11*
	Inflammatory fibroblasts	*IL11, CXCL5, FAP, INHBA and IL24*
	Endothelial cells	*VWF, PECAM1 and PLVAP*
	Activated endothelium	*ACKR1, SELE and SELP*
	Lymphatic endothelium	*MRC1, MMRN1 and LYVE1*
	Pericytes	*NOTCH3 and RGS5*
	Glial cells	*NRXN1, S100B and CDH19*
B and plasma cell subset	Plasma cells	*DERL3, MZB1, XBP1.*
	plasmablasts, PB	*PRDM1low*
	Fully differentiated plasma cells, PC	*PRDM1high*
	Lambda cluster	*IGLC3 and IGLC2*
	PC IGLL5	*IGLL5, IGLC7 and IGLL1*
	PC IER	*FOS, JUN and FOSB*
	B cells	*CD20 (MS4A1).*
	memory B cells	*(ITGAX, FGR and PDCD1)*
	naive B cells	*IGHD and FCER2*
	germinal center (GC) B cells	*SUGCT, AICDA and TCL1A*
	B cell Ribhi	*RPS27, RPL37*
	Cycling cells (PC and B cells)	*TUBB, TOP2A, MKI67*
T cell subset (CD3D, CD3E and CD3G)	CD8 T cells	*CD8A, CD8B*
	CD4 cells	*CD4low*
	CD4 naïve	*CCR7, LEF1 and SELL*
	CD4 ANXA1 (resident phenotype)	*ANXA1, IL7R and GPR186*
	Tregs	*FOXP3, IL2RA, CXCR6 and BATF*
	T follicular helper cells, ThF	*CXCL13 and MAGEH1*
	CCL20 T cells	*CCL20, RORA, KLRB1, ETPD1*
	S1PR1 T cells. Circulating phenotype (Central Memory Cells)	*CCR4, KLF2 and S1PR1*
	CD8 cytotoxic effector cells	*GZMK and KLRG1*

	CD8 TRM	*GZMK-, KLRB1, SPRY1 and IGTA1*
	CD8 FGFBP2 (Corridoni et al)	*FGFBP2, FCGR3A, S1PR5, GZMH, NKG7*
	MAIT	*CD8A, NRC3*
	DN TNF	*CD8-CD4-, TNF, FOS, JUN and FOSB*
	DN EOMES	*CD8-CD4-, EOMES, GZMK*
	GD IEL	*TRDC, TRGC1, FCER1G, KLRC2, CD160, KLRD1, KLRC1, ITGA1 and KIR2DL4*
	ILC3	*LIF, KIT and PCDH9*
	NK	*KLRF1, NCAM1, and lacks expression of CD3G, CD3D, CD8A and CD4*
Myeloid cell subset	Macrophages	*CD68, CD14 and complement coding-genes (C1QA, C1QB)*
	M2 and M2.2 macrophages	*CD209, CD163L1 and FOLR2*
	M0 macrophages	*lack the expression of M2 markers and expression of CD68, C1QA, C1QB, SELENOP, LYZ, HLA-DPB1, HLA-DPA1, AIF1*
	IDA macrophages	*NRG1 AREG, EREG and HBEGF*
	M1 ACOD1	*ACOD1,TNIP3, IL1B, INHBA, IL6, VCAN, CD300E*
	M1 CXCL5	*CXCL5, TNIP3, IL1B, INHBA, IL6, VCAN, CD300E*
	inflammatory monocytes	*VCAN, CD300E, CD14, FCN1 and S100A9*
	Mast cells	*TPSB2 and TPSAB1*
	Mast cells 2	*LTC4S*
	eosinophils	*CLC, IL4 and IL13*
	Neutrophils	*PROK2, CMTM2, CXCL8, FCRG3B, AQP9, S100A8 and S100A9*
	DCs	*LAMP3*
	DCs CD1c	*CD1C, TRL10 and TCTN3*
	DCs CCL22	*CCL22 and CCL19*
