# Supplementary material for: Macrophage and neutrophil heterogeneity at single-cell spatial resolution in human inflammatory bowel disease
Source: Nat Commun. 2023 Jul 26;14:4506. doi: 10.1038/s41467-023-40156-6 (PMC10372067; doi:10.1038/s41467-023-40156-6)
Supplement: Supplementary file 3 — Reporting Summary [file 41467_2023_40156_MOESM3_ESM.pdf]

nature portfolio

Corresponding author(s): Azucena Salas

Last updated by author(s): Jun 16, 2023

Reporting Summary

Nature Portfolio wishes to improve the reproducibility of the work that we publish. This form provides structure for consistency and transparency in reporting. For further information on Nature Portfolio policies, see our Editorial Policies and the Editorial Policy Checklist.

Statistics

For all statistical analyses, confirm that the following items are present in the figure legend, table legend, main text, or Methods section.

n/a	Confirmed	
☐	☒	The exact sample size (*n*) for each experimental group/condition, given as a discrete number and unit of measurement
☐	☒	A statement on whether measurements were taken from distinct samples or whether the same sample was measured repeatedly
☐	☒	The statistical test(s) used AND whether they are one- or two-sided<br>*Only common tests should be described solely by name; describe more complex techniques in the Methods section.*
☐	☒	A description of all covariates tested
☐	☒	A description of any assumptions or corrections, such as tests of normality and adjustment for multiple comparisons
☐	☒	A full description of the statistical parameters including central tendency (e.g. means) or other basic estimates (e.g. regression coefficient) AND variation (e.g. standard deviation) or associated estimates of uncertainty (e.g. confidence intervals)
☒	☐	For null hypothesis testing, the test statistic (e.g. *F*, *t*, *r*) with confidence intervals, effect sizes, degrees of freedom and *P* value noted<br>*Give P values as exact values whenever suitable.*
☒	☐	For Bayesian analysis, information on the choice of priors and Markov chain Monte Carlo settings
☒	☐	For hierarchical and complex designs, identification of the appropriate level for tests and full reporting of outcomes
☒	☐	Estimates of effect sizes (e.g. Cohen's *d*, Pearson's *r*), indicating how they were calculated

Our web collection on statistics for biologists contains articles on many of the points above.

Software and code

Policy information about availability of computer code

Data collection	Single-cell RNA sequencing: Chromium10x Genomics 3' platform and Illumina HiSeq2500; Bulk RNA sequencing: Illumina HighSeq-3000; Image acquisition: Nikon ELIPSE Ti microscope (Nis-Elements Basic Research Software v.5.30.05); FACS acquisition: BD FACSCanto II flow cytometer (BD).
Data analysis	The following softwares/packages were used CellRanger (v 3.1.0), R (v4.2.0 for scRNA-seq, v3.2.3 for bulk RNA-seq), SeuratObject R package (v 4.0.2), scDblFinder R package (v 1.8.0), Surat (v. 4.1.0), Harmony (v 0.1.1.), Milo package (v 1.4.0), Monocle 2 (v 2.24.1), Matschore (v 0.1.0), Scater (v. 1.22.0), Scran (v 1.22.1), Batchelor (1.10.0), SingleR (v 1.8.1), Hmisc R package (v 4.6-0), corrplot (v 0.92), Skewer (v 2.2.8), STAR aligner (v 2.5.2a), RSEM (v 1.2.31), Graphpad Prism (v 9.0), ImageJ (v 1.53t). Flow cytometry: FlowJO software v.10.6.1 (BD). CosMxTM technology:CosMxTM Human Universal Cell Characterization RNA Panel (1000-plex) + 20 custom genes; Nanostring. Full code for scRNAseq analysis is available at https://github.com/ibd-bcn/ibd-bcn_single_cell and https://servidor2-ciberehd.upc.es/external/garrido/methods2/ . Full code for analysis of CosMx SMI data can be found at https://github.com/HelenaLC/CosMx-SMI-IBD.

For manuscripts utilizing custom algorithms or software that are central to the research but not yet described in published literature, software must be made available to editors and reviewers. We strongly encourage code deposition in a community repository (e.g. GitHub). See the Nature Portfolio guidelines for submitting code & software for further information.

Data

Policy information about availability of data

All manuscripts must include a data availability statement. This statement should provide the following information, where applicable:

- Accession codes, unique identifiers, or web links for publicly available datasets
- A description of any restrictions on data availability
- For clinical datasets or third party data, please ensure that the statement adheres to our policy

Expression profiles and raw single-cell RNA sequencing data of all cell compartments has been deposited in GEO: GSE214695. The CosMx SMI data are available at GSE234713. Bulk RNA-seq data from human colonic biopsies is accessible at GSE235236. Analyzed data can be explored in our interactive webpage:https://servidor2-ciberehd.upc.es/external/garrido/app/.

Human research participants

Policy information about studies involving human research participants and Sex and Gender in Research.

Reporting on sex and gender	Our findings apply to both female and male sex. Sex or gender were not considered in our study design and both male and female were included. Cohort 1, 2 and 3 contain 38.9 %, 55.5% and 44.6% female respectively. In general, sex based analysis was not performed due to limited sample size. Consent was given by participants for sharing of individual-level data. Disaggregated data is provided in the source data files.
Population characteristics	Cohort 1: age range 21-68. We analyzed 6 non-IBD controls, 6 CD and 6 UC patients for Single-cell RNA-seq. Biopsies of IBD patients were taken from active areas of the colon at colonoscopy. IBD patients medications at the time of colonoscopy included 41.6% of corticosteroids, 25% vedolizumab, 8.3% ustekinumab, 8.3% a-TNF and 8,3% aminosalicylates. Cohort 2: age range 38-69. FFPE blocks from surgical pieces or biopsies of 3 non-IBD controls, 3 CD and 3 UC patients were processed for CosMx SMI. Biopsies and surgical piece for IBD patients were taken from active areas of the colon at colonoscopy or resected from surgical piece. IBD patients medications at the time of sample harvesting included 33,33% tofacitinib, 16,66% ustekinumab, 16,66% a-TNF, 16,66% immunosupressor and 16,66% antibiotics. Cohort 3: age range 19-69. 8 non-IBD controls, 22 CD and 26 UC were anlayzed for Bulk RNA-seq. Biopsies were taken from active areas of the colon. TREATMENTS?. Additionally, active colonic biopsies were harvested from UC and CD, and non-IBD patients for flow cytometry analysis, and resections from healthy areas of 13 patients undergoing surgery for colorectal cancer were used for epithelial organoid culture. IBD patients treatment at the time of colonoscopy or surgery included 51,78% immunosupressors, 16% corticosteroids, 5,35% aminosalicylates., 1,78% a-TNF and 10,71% no treatment.
Recruitment	Patients were recruited in the Hospital Clinic de Barcelona or Mutua Terrasa Hospital, Spain. Colon biopsies were collected during routine endoscopies performed as standard of care. Healthy controls were individuals undergoing endoscopy for colorectal cancer screening and presenting no signs of dysplasia or polyps at the time of endoscopy. Surgical colon resections were obtained from patients undergoing surgery for colorectal cancer, or from UC and CD patients (undergoing colonic resective surgery). Blood was harvested from patients before colonoscopy. Selection was based exclusively on and established diagonosis of either UC or CD and disease activity at the time of inclusion. No other selection biased was applied.
Ethics oversight	The study was approved by the Ethics Committee of Hospital Clinic Barcelona (HCB/2018/1062 and HCB/2022/0125) and the Hospital Mutua de Terrassa (CI201901). All patients signed an informed consent at the time of colonoscopy or before surgical intervention. Participants received no compensation for the study.

Note that full information on the approval of the study protocol must also be provided in the manuscript.

Field-specific reporting

Please select the one below that is the best fit for your research. If you are not sure, read the appropriate sections before making your selection.

☒ Life sciences ☐ Behavioural & social sciences ☐ Ecological, evolutionary & environmental sciences

For a reference copy of the document with all sections, see nature.com/documents/nr-reporting-summary-flat.pdf

Life sciences study design

All studies must disclose on these points even when the disclosure is negative.

Sample size	The number of available samples was determined by the number of interventions (surgery or colonoscopy) carried out that meet the standar of active (IBD) or healthy colon (HC). In the manuscript sample size for every analysis is shown with proper statistics.
Data exclusions	Cells from single-cell RNA.-seq analysis from non-IBD controls and IBD patients that did not match our quality control (https://github.com/ibd-bcn/ibd-bcn_single_cell) were removed from the analysis.<br>Bulk RNA-seq from biopsies that failed quality control (methods) were also removed.

Replication	Given the sample nature, technical replications involving human samples were not possible for single-cell RNA-seq, CosMX SMI, Bulk RNAseq, flow cytometry and organoid culture. All replicates in those cases were biological. For immunostaining experiments, when possible, technical replicates were performed in order to validate results. For immunostaining stainings were performed in at least 3 independent donors per group (HC, UC and CD when all groups were included). For identification of CD68/CD209 12 independent HC control donors were analyzed. For MPO and MBP samples from 9HC, 6 UC and 5 CD patients were analyzed. ISH fro NRG1 or OLFM4 was performed on 3 HC and 6 IBD patients. Findings were replicated in all tested samples.
Randomization	Randomization was not relevant for the results of this manuscript. For each experiment, all samples were processed equally (regardless of their patient group). Samples were collected as needed for the different applications based on patient availability at the time of analysis.
Blinding	All human samples used in this manuscript were blinded right after collection by giving them a unique ID. Researchers were unaware of the sample origin (HC, UC or CD) during processing of the sample and raw data analysis (scRNAseq, immunostainings, CosMx and bulk RNAseq). Once data was collected and processed, analysis required group assignment and thus researchers had to be aware of their patient group (HC, UC or CD).

Reporting for specific materials, systems and methods

We require information from authors about some types of materials, experimental systems and methods used in many studies. Here, indicate whether each material, system or method listed is relevant to your study. If you are not sure if a list item applies to your research, read the appropriate section before selecting a response.

Materials & experimental systems

n/a	Involved in the study
☐	☒ Antibodies
☒	☐ Eukaryotic cell lines
☒	☐ Palaeontology and archaeology
☒	☐ Animals and other organisms
☒	☐ Clinical data
☒	☐ Dual use research of concern

Methods

n/a	Involved in the study
☒	☐ ChIP-seq
☐	☒ Flow cytometry
☒	☐ MRI-based neuroimaging

Antibodies

Antibodies used	Antibodies used for IHQ and IHF: rabbit anti-MPO ref HPA021147 (Sigma)@1/3000; mouse anti-MBP clone BMK13 ref CBL419 (Sigma)@1/100; rabbit anti-OLFM4 ref 14369S (Cell Signaling)@1:100; mouse anti-CD209 clone DC28 ref SC-65740 (Santa Cruz Biotechnology)@1:500 for IHQ and @1:200 for IHF; rabbit anti-CD68 ref HPA048982 (Sigma)@1:500 for IHQ and @1:200 for IHF. Secondaries: anti-rabbit ref ZC0908 (Vector)@1/200; anti-mouse ref BA-2000 (Vector)@1:200; anti-rabbit AF488 ref 111-545-144 (Jackson ImmunoResearch)@1:400; anti-mouse Cy3 ref 115-165-205 (Jackson ImmunoResearch)@1:400. Antibodies used for flow cytometry: CD66b PE-Cy7 (#305116 clone G10F5, 5 ul/tube, BioLegend), CD16 PerCP (#302028 clone 3G8, 5 ul/tube, BioLegend), CD62L APC-Cy7 (#304814 clone DREG-56, 5 ul/tube, BioLegend), CD69 BV421 (#310930 clone FN50, 5 ul/tube, BioLegend), CD193 FITC (#310720 clone 5E8, 5 ul/tube, BioLegend), CD63 FITC (#353005 clone H5C6, 5 ul/tube, BioLegend) and CXCR4 PE (# FAB170P clone 12G5, 10 ul/tube, R&D systems) and Zombie Aqua Fixable Viability Kit 1/1000 (#423101, BioLegend) for death cells.
Validation	FACS:<br>Biolegend antibodies have been validated by the manufacturer for flow cytometry use. CXCR4 PE (R&D systems) has been validated by the manufacturer for flow cytometry use.<br><br>IHQ and IHF:<br>rabbit anti-MPO ref HPA021147 (Sigma); mouse anti-MBP clone BMK13 ref CBL419 (Sigma); rabbit anti-OLFM4 ref 14369S (Cell Signaling); mouse anti-CD209 ref SC-65740 (Santa Cruz Biotechnology); rabbit anti-CD68 ref HPA048982 (Sigma) have been all validated for immunostaining use by the manufacturer.

Flow Cytometry

Plots

Confirm that:

☒ The axis labels state the marker and fluorochrome used (e.g. CD4-FITC).

☒ The axis scales are clearly visible. Include numbers along axes only for bottom left plot of group (a 'group' is an analysis of identical markers).

☒ All plots are contour plots with outliers or pseudocolor plots.

☒ A numerical value for number of cells or percentage (with statistics) is provided.

Methodology

Sample preparation

Biopsies (n=4-6 per patient) were taken from involved areas of the colon of UC and CD patients with signs of endoscopic activity, placed immediately in cold Hank's Balanced Salt Solution (HBSS) (Gibco, MA, USA) and kept at 4ºC until processing (<1 h). Colonic biopsies from non-IBD controls were collected from the sigmoid colon and processed in the same way. Freshly collected biopsies were washed with 5mM DTT (Roche, Spain) in HBSS for 15 min and then washed in complete medium (CM) (RPMI 1640 medium (Lonza, MD, USA) supplemented with 10% heat-inactivated fetal bovine serum (FBS) (Biosera, France), 100U/ml penicillin, 100 U/ml streptomycin and 250 ng/ml amphotericin B (Lonza), 10µg/ml gentamicin sulfate (Lonza) and 1,5mM Hepes (Lonza)) for 10 minutes. Both incubations were performed at room temperature in a platform rocker. Biopsies were chopped with a scalpel and placed into tubes containing 500 µl of Digestion Solution (CM + Liberase TM (0.5 Wünsch units/ml) (Roche, Spain) + DNase I (10 µg/mL) (Roche, Spain)) and incubated on a shaking platform for 1h at 250 RPM and 37ºC. After incubation biopsies were filtered through a 50-µm cell strainer (CellTrics, Sysmex, USA), washed with Dulbecco's Phosphate Buffered Saline (PBS; Gibco, USA) and resuspended in FACS buffer (PBS + 2% inactivated FBS (fetal bovine serum) + NaN3 0.1%) for flow cytometry analysis. Human granulocytes were isolated from blood by a double density gradient. In brief, diluted blood in PBS (1/2) was layered over Lymphoprep™ (1.077 g/ml) that was layered over denser Polymorphprep™ (1.113 g/ml). The double gradient was then centrifuged at 500g for 30 min obtaining 2 separated cell layers. The lower layer containing the granulocytes was collected, washed and red blood cells were lysed using a commercial lysis buffer (BioLegend). Purity of granulocytes achieved with this method is >95%

Instrument

FACS analysis was done on a BD FACSCanto II (BD)

Software

FACS data was analyzed in FlowJO software (BD) v.10.6.1

Cell population abundance

FACS was used only for analysis. Cells were not sorted. The abundance of neutrophils and eosinophils is shown in Extended Figure 11a.

Gating strategy

Cells were selected in FSC/SSC and from there doublets were removed by using FSC-A/FSC-H. Live cells were selected using Aqua Zombie L/D (Biolegend). Eosinophils and neutrophils were gated from live cells using CD66b and CD16. CD66b+CD16+ (neutrophils) and CD66b+CD16- (eosinophils)

☒ Tick this box to confirm that a figure exemplifying the gating strategy is provided in the Supplementary Information.
